# Gene selection and classification of microarray data using random forest

**DOI:** 10.1186/1471-2105-7-3

**Published:** 2006-01-06

**Authors:** Ramón Díaz-Uriarte, Sara Alvarez de Andrés

**Affiliations:** 1 Bioinformatics Unit, Biotechnology Programme, Spanish National Cancer Centre (CNIO), Melchor Fernandez Almagro 3, Madrid, 28029, Spain; 2 Cytogenetics Unit, Biotechnology Programme, Spanish National Cancer Centre (CNIO), Melchor Fernández Almagro 3, Madrid, 28029, Spain

## Abstract

**Background:**

Selection of relevant genes for sample classification is a common task in most gene expression studies, where researchers try to identify the smallest possible set of genes that can still achieve good predictive performance (for instance, for future use with diagnostic purposes in clinical practice). Many gene selection approaches use univariate (gene-by-gene) rankings of gene relevance and arbitrary thresholds to select the number of genes, can only be applied to two-class problems, and use gene selection ranking criteria unrelated to the classification algorithm. In contrast, random forest is a classification algorithm well suited for microarray data: it shows excellent performance even when most predictive variables are noise, can be used when the number of variables is much larger than the number of observations and in problems involving more than two classes, and returns measures of variable importance. Thus, it is important to understand the performance of random forest with microarray data and its possible use for gene selection.

**Results:**

We investigate the use of random forest for classification of microarray data (including multi-class problems) and propose a new method of gene selection in classification problems based on random forest. Using simulated and nine microarray data sets we show that random forest has comparable performance to other classification methods, including DLDA, KNN, and SVM, and that the new gene selection procedure yields very small sets of genes (often smaller than alternative methods) while preserving predictive accuracy.

**Conclusion:**

Because of its performance and features, random forest and gene selection using random forest should probably become part of the "standard tool-box" of methods for class prediction and gene selection with microarray data.

## Background

Selection of relevant genes for sample classification (e.g., to differentiate between patients with and without cancer) is a common task in most gene expression studies (e.g., [1-6]). When facing gene selection problems, biomedical researchers often show interest in one of the following objectives:

1. To identify relevant genes for subsequent research; this involves obtaining a (probably large) set of genes that are related to the outcome of interest, and this set should include genes even if they perform similar functions and are highly correlated.

2. To identify small sets of genes that could be used for diagnostic purposes in clinical practice; this involves obtaining the smallest possible set of genes that can still achieve good predictive performance (thus, "redundant" genes should not be selected).

We will focus here on the second objective. Most gene selection approaches in class prediction problems combine ranking genes (e.g., using an *F*-ratio or a Wilcoxon statistic) with a specific classifier (e.g., discriminant analysis, nearest neighbor). Selecting an optimal number of features to use for classification is a complicated task, although some preliminary guidelines, based on simulation studies by [4], are available. Frequently an arbitrary decision as to the number of genes to retain is made (e.g., keep the 50 best ranked genes and use them with a linear discriminant analysis as in [1,7]; keep the best 150 genes as in [8]). This approach, although it can be appropriate when the only objective is to classify samples, is not the most appropriate if the objective is to obtain the smaller possible sets of genes that will allow good predictive performance. Another common approach, with many variants (e.g., [9-11]), is to repeatedly apply the same classifier over progressively smaller sets of genes (where we exclude genes based either on the ranking statistic or on the effect of the elimination of a gene on error rate) until a satisfactory solution is achieved (often the smallest error rate over all sets of genes tried). A potential problem of this second approach, if the elimination is based on univariate rankings, is that the ranking of a gene is computed in isolation from all other genes, or at most in combinations of pairs of genes [12], and without any direct relation to the classification algorithm that will later be used to obtain the class predictions. Finally, the problem of gene selection is generally regarded as much more problematic in multi-class situations (where there are three or more classes to be differentiated), as evidence by recent papers in this area (e.g., [2,8]). Therefore, classification algorithms that directly provide measures of variable importance (related to the relevance of the variable in the classification) are of great interest for gene selection, specially if the classification algorithm itself presents features that make it well suited for the types of problems frequently faced with microarray data. Random forest is one such algorithm.

Random forest is an algorithm for classification developed by Leo Breiman [13] that uses an ensemble of classification trees [14-16]. Each of the classification trees is built using a bootstrap sample of the data, and at each split the candidate set of variables is a random subset of the variables. Thus, random forest uses both bagging (bootstrap aggregation), a successful approach for combining unstable learners [16,17], and random variable selection for tree building. Each tree is unpruned (grown fully), so as to obtain low-bias trees; at the same time, bagging and random variable selection result in low correlation of the individual trees. The algorithm yields an ensemble that can achieve both low bias and low variance (from averaging over a large ensemble of low-bias, high-variance but low correlation trees).

Random forest has excellent performance in classification tasks, comparable to support vector machines. Although random forest is not widely used in the microarray literature (but see [18-23]), it has several characteristics that make it ideal for these data sets:

a) Can be used when there are many more variables than observations.

b) Can be used both for two-class and multi-class problems of more than two classes.

c) Has good predictive performance even when most predictive variables are noise, and therefore it does not require a pre-selection of genes (i.e., "shows strong robustness with respect to large feature sets", *sensu *[4]).

d) Does not overfit.

e) Can handle a mixture of categorical and continuous predictors.

f) Incorporates interactions among predictor variables.

g) The output is invariant to monotone transformations of the predictors.

h) There are high quality and free implementations: the original Fortran code from L. Breiman and A. Cutler, and an R package from A. Liaw and M. Wiener [24].

i) Returns measures of variable (gene) importance.

j) There is little need to fine-tune parameters to achieve excellent performance. The most important parameter to choose is *mtry*, the number of input variables tried at each split, but it has been reported that the default value is often a good choice [24]. In addition, the user needs to decide how many trees to grow for each forest (*ntree*) as well as the minimum size of the terminal nodes (*nodesize*). These three parameters will be thoroughly examined in this paper.

Given these promising features, it is important to understand the performance of random forest compared to alternative state-of-the-art prediction methods with microarray data, as well as the effects of changes in the parameters of random forest. In this paper we present, as necessary background for the main topic of the paper (gene selection), the first through examination of these issues, including evaluating the effects of *mtry*, *ntree *and *nodesize *on error rate using nine real microarray data sets and simulated data.

The main question addressed in this paper is gene selection using random forest. A few authors have previously used variable selection with random forest. [25] and [20] use filtering approaches and, thus, do not take advantage of the measures of variable importance returned by random forest as part of the algorithm. Svetnik, Liaw, Tong and Wang [26] propose a method that is somewhat similar to our approach. The main difference is that [26] first find the "best" dimension (*p*) of the model, and then choose the *p *most important variables. This is a sound strategy when the objective is to build accurate predictors, without any regards for model interpretability. But this might not be the most appropriate for our purposes as it shifts the emphasis away from selection of specific genes, and in genomic studies the identity of the selected genes is relevant (e.g., to understand molecular pathways or to find targets for drug development).

The last issue addressed in this paper is the multiplicity (or lack of uniqueness or lack of stability) problem. Variable selection with microarray data can lead to many solutions that are equally good from the point of view of prediction rates, but that share few common genes. This multiplicity problem has been emphasized by [27] and [28] and recent examples are shown in [29] and [30]. Although multiplicity of results is not a problem when the only objective of our method is prediction, it casts serious doubts on the biological interpretability of the results [27]. Unfortunately most "methods papers" in bioinformatics do not evaluate the stability of the results obtained, leading to a false sense of trust on the biological interpretability of the output obtained. Our paper presents a through and critical evaluation of the stability of the lists of selected genes with the proposed (and two competing) methods.

In this paper we present the first comprehensive evaluation of random forest for classification problems with microarray data, including an assessment of the effects of changes in its parameters and we show it to be an excellent performer even in multi-class problems, and without any need to fine-tune parameters or pre-select relevant genes. We then propose a new method for gene selection in classification problems (for both two-class and multi-class problems) that uses random forest; the main advantage of this method is that it returns very small sets of genes that retain a high predictive accuracy, and is competitive with existing methods of gene selection.

## Results

### Evaluation of performance and comparisons with alternative approaches

We have used both simulated and real microarray data sets to evaluate the variable selection procedure. For the real data sets, original reference paper and main features are shown in Table 1 and further details are provided in the supplementary material [see Additional file 1]. To evaluate if the proposed procedure can recover the signal in the data and can eliminate redundant genes, we need to use simulated data, so that we know exactly which genes are relevant. Details on the simulated data are provided in the methods and in the supplementary material [see Additional file 1].

We have compared the predictive performance of the variable selection approach with: a) random forest without any variable selection (using mtry=number of genes
 MathType@MTEF@5@5@+=feaafiart1ev1aaatCvAUfKttLearuWrP9MDH5MBPbIqV92AaeXatLxBI9gBaebbnrfifHhDYfgasaacH8akY=wiFfYdH8Gipec8Eeeu0xXdbba9frFj0=OqFfea0dXdd9vqai=hGuQ8kuc9pgc9s8qqaq=dirpe0xb9q8qiLsFr0=vr0=vr0dc8meaabaqaciGacaGaaeqabaqabeGadaaakeaacqWGTbqBcqWG0baDcqWGYbGCcqWG5bqEcqGH9aqpdaGcaaqaaiabd6gaUjabdwha1jabd2gaTjabdkgaIjabdwgaLjabdkhaYjaaykW7cqWGVbWBcqWGMbGzcaaMc8Uaem4zaCMaemyzauMaemOBa4MaemyzauMaem4Camhaleqaaaaa@4876@, *ntree *= 5000, *nodesize *= 1); b) three other methods that have shown good performance in reviews of classification methods with microarray data [7,31,32] but that do not include any variable selection; c) three methods that carry out variable selection. For the three methods that do not carry out variable selection, **Diagonal Linear Discriminant Analysis (DLDA)**, **K nearest neighbor (KNN)**, and **Support Vector Machines (SVM) **with linear kernel, we have used, based on [7], the 200 genes with the largest *F*-ratio of between to within groups sums of squares. For **KNN**, the number of neighbors (*K*) was chosen by cross-validation as in [7]. The methods that incorporate variable selection are two different versions of **Shrunken centroids (SC) **[33], **SC.l **and **SC.s**, as well as **Nearest neighbor + variable selection (NN.vs)**; further details are provided in the methods and in the supplementary material [see Additional file 1].

### Estimation of error rates

To estimate the prediction error rate of all methods we have used the .632+ bootstrap method [34,35]. The .632+ bootstrap method uses a weighted average of the resubstitution error (the error when a classifier is applied to the training data) and the error on samples not used to train the predictor (the "leave-one-out" bootstrap error); this average is weighted by a quantity that reflects the amount of overfitting. It must be emphasized that the error rate used when performing variable selection is not what we report in as prediction error rate in Tables 2 or 3. To calculate the prediction error rate as reported, for example, in Table 2, the .632+ bootstrap method is applied to the complete procedure, and thus the samples used to compute the leave-one-out bootstrap error used in the .632+ method are samples that are not used when fitting the random forest, or carrying out variable selection. The .632+ bootstrap method was also used when evaluating the competing methods.

### Effects of parameters of random forest on prediction error rate

Before examining gene selection, we first evaluated the effect of changes in parameters of random forest on its classification performance. Random forest returns a measure of error rate based on the out-of-bag cases for each fitted tree, the OOB error, and this is the measure of error we will use here to assess the effects of parameters. We examined whether the OOB error rate is substantially affected by changes in *mtry*, *ntree*, and *nodesize*.

**Figure 1:**
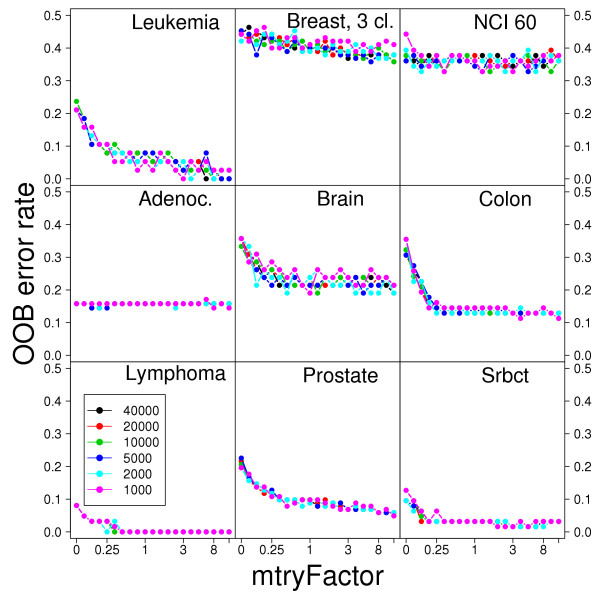
**Out-of-Bag (OOB) vs *mtryFactor *for the nine microarray data sets**. *mtryFactor *is the multiplicative factor of the default *mtry *(number⋅of⋅genes
 MathType@MTEF@5@5@+=feaafiart1ev1aaatCvAUfKttLearuWrP9MDH5MBPbIqV92AaeXatLxBI9gBaebbnrfifHhDYfgasaacH8akY=wiFfYdH8Gipec8Eeeu0xXdbba9frFj0=OqFfea0dXdd9vqai=hGuQ8kuc9pgc9s8qqaq=dirpe0xb9q8qiLsFr0=vr0=vr0dc8meaabaqaciGacaGaaeqabaqabeGadaaakeaadaGcaaqaaiabd6gaUjabdwha1jabd2gaTjabdkgaIjabdwgaLjabdkhaYjabgwSixlabd+gaVjabdAgaMjabgwSixlabdEgaNjabdwgaLjabd6gaUjabdwgaLjabdohaZbWcbeaaaaa@4332@); thus, an *mtryFactor *of 3 means the number of genes tried at each split is 3 * number⋅of⋅genes
 MathType@MTEF@5@5@+=feaafiart1ev1aaatCvAUfKttLearuWrP9MDH5MBPbIqV92AaeXatLxBI9gBaebbnrfifHhDYfgasaacH8akY=wiFfYdH8Gipec8Eeeu0xXdbba9frFj0=OqFfea0dXdd9vqai=hGuQ8kuc9pgc9s8qqaq=dirpe0xb9q8qiLsFr0=vr0=vr0dc8meaabaqaciGacaGaaeqabaqabeGadaaakeaadaGcaaqaaiabd6gaUjabdwha1jabd2gaTjabdkgaIjabdwgaLjabdkhaYjabgwSixlabd+gaVjabdAgaMjabgwSixlabdEgaNjabdwgaLjabd6gaUjabdwgaLjabdohaZbWcbeaaaaa@4332@; an *mtryFactor *= 0 means the number of genes tried was 1; the *mtryFactors *examined were = {0, 0.05, 0.1, 0.17, 0.25, 0.33, 0.5, 0.75, 0.8, 1, 1.15, 1.33, 1.5, 2, 3, 4, 5, 6, 8, 10, 13}. Results shown for six different *ntree *= {1000, 2000, 5000, 10000, 20000, 40000}, *nodesize *= 1.

Figure 1 and the Figure"error.vs.mtry.pdf" in Additional file 2 show that, for both real and simulated data, the relation of OOB error rate with *mtry *is largely independent of *ntree *(for *ntree *between 1000 and 40000) and *nodesize *(nodesizes 1 and 5). In addition, the default setting of *mtry *(*mtryFactor *= 1 in the figures) is often a good choice in terms of OOB error rate. In some cases, increasing *mtry *can lead to small decreases in error rate, and decreases in *mtry *often lead to increases in the error rate. This is specially the case with simulated data with very few relevant genes (with very few relevant genes, small *mtry *results in many trees being built that do not incorporate any of the relevant genes). Since the OOB error and the relation between OOB error and *mtry *do not change whether we use *nodesize *of 1 or 5, and because the increase in computing speed from using *nodesize *of 5 is inconsequential, all further analyses will use only the default *nodesize *= 1. These results show the robustness of random forest to changes in its parameters; nevertheless, to re-examine robustness of gene selection to these parameters, in the rest of the paper we will report results for different settings of *ntree *and *mtry *(and these results will again show the robustness of the gene selection results to changes in *ntree *and *mtry*).

The error rates of random forest (without gene selection) compared with the alternative methods, using the real microarray data, and estimated in all cases using the .632+ bootstrap method, are shown in Table 2. These results clearly show that random forest has a predictive performance comparable to that of the alternative methods, without any need for pre-selection of genes or tuning of its parameters.

### Gene selection using random forest

Random forest returns several measures of variable importance. The most reliable measure is based on the decrease of classification accuracy when values of a variable in a node of a tree are permuted randomly [13,36], and this is the measure of variable importance (in its unscaled version – see Additional file 1) that we will use in the rest of the paper. (In the Supplementary material [see Additional file 1] we show that this measure of variable importance is not the same as a non-parametric statistic of difference between groups, such as could be obtained with a Kruskal-Wallis test). Other measures of variable importance are available, however, and future research should compare the performance of different measures of importance.

To select genes we iteratively fit random forests, at each iteration building a new forest after discarding those variables (genes) with the smallest variable importances; the selected set of genes is the one that yields the smallest OOB error rate. Note that in this section we are using OOB error to choose the final set of genes, not to obtain unbiased estimates of the error rate of this rule. Because of the iterative approach, the OOB error is biased down and cannot be used to asses the overall error rate of the approach, for reasons analogous to those leading to "selection bias" [34,37]. To assess prediction error rates we will use the bootstrap, not OOB error (see above). (Using error rates affected by selection bias to select the optimal number of genes is not necessarily a bad procedure from the point of view of selecting the final number of genes; see [38]).

In our algorithm we examine all forests that result from eliminating, iteratively, a fraction, *fraction.dropped*, of the genes (the least important ones) used in the previous iteration. By default, *fraction.dropped *= 0.2 which allows for relatively fast operation, is coherent with the idea of an "aggressive variable selection" approach, and increases the resolution as the number of genes considered becomes smaller. We do not recalculate variable importances at each step as [26] mention severe overfitting resulting from recalculating variable importances. After fitting all forests, we examine the OOB error rates from all the fitted random forests. We choose the solution with the smallest number of genes whose error rate is within *u *standard errors of the minimum error rate of all forests. Setting *u *= 0 is the same as selecting the set of genes that leads to the smallest error rate. Setting *u *= 1 is similar to the common "1 s.e. rule", used in the classification trees literature [14,15]; this strategy can lead to solutions with fewer genes than selecting the solution with the smallest error rate, while achieving an error rate that is not different, within sampling error, from the "best solution". In this paper we will examine both the "1 s.e. rule" and the "0 s.e. rule".

On the simulated data sets [see Additional file 1, Tables 3 and 4] backwards elimination often leads to very small sets of genes, often much smaller than the set of "true genes". The error rate of the variable selection procedure, estimated using the .632+ bootstrap method, indicates that the variable selection procedure does not lead to overfitting, and can achieve the objective of aggressively reducing the set of selected genes. In contrast, when the simplification procedure is applied to simulated data sets without signal (see Tables 1 and 2 in Additional file 1), the number of genes selected is consistently much larger and, as should be the case, the estimated error rate using the bootstrap corresponds to that achieved by always betting on the most probable class.

Results for the real data sets are shown in Tables 2 and 3 (see also Additional file 1, Tables 5, 6, 7, for additional results using different combinations of *ntree *= {2000, 5000, 20000}, *mtryFactor *= {1, 13}, *se *= {0, 1}, *fraction.dropped *= {0.2, 0.5}). Error rates (see Table 2) when performing variable selection are in most cases comparable (within sampling error) to those from random forest without variable selection, and comparable also to the error rates from competing state-of-the-art prediction methods. The number of genes selected varies by data set, but generally (Table 3) the variable selection procedure leads to small (< 50) sets of predictor genes, often much smaller than those from competing approaches (see also Table 8 in Additional file 1 and discussion). There are no relevant differences in error rate related to differences in *mtry*, *ntree *or whether we use the "s.e. 1" or "s.e. 0" rules. The use of the "s.e. 1" rule, however, tends to result in smaller sets of selected genes.

### Stability (uniqueness) of results

Following [39,40], and [41], we have evaluated the stability of the variable selection procedure using the bootstrap. This allows us to asses how often a given gene, selected when running the variable selection procedure in the original sample, is selected when running the procedure on bootstrap samples.

The results here will focus on the real microarray data sets (results from the simulated data are presented in Additional file 1). Table 3 (see also Additional file 1, Tables 5, 6, 7, for other combinations of *ntree*, *mtryFactor*, *fraction.dropped*, *se*) shows the variation in the number of genes selected in bootstrap samples, and the frequency with which the genes selected in the original sample appear among the genes selected from the bootstrap samples. In most cases, there is a wide range in the number of genes selected; more importantly, the genes selected in the original samples are rarely selected in more than 50% of the bootstrap samples. These results are not strongly affected by variations in *ntree *or *mtry*; using the "s.e. 1" rule can lead, in some cases, to increased stability of the results.

As a comparison, we also show in Table 3 the stability of two alternative approaches for gene selection, the shrunken centroids method, and a filter approach combined with a Nearest Neighbor classifier (see Table 8 in Additional file 1 for results of SC.l). Error rates are comparable, but both alternative methods lead to much larger sets of selected genes than backwards variable selection with random forests. The alternative approaches seem to lead to somewhat more stable results in variable selection (probably a consequence of the large number of genes selected) but in practical applications this increase in stability is probably far out-weighted by the very large number of selected genes.

## Discussion

We have first presented an exhaustive evaluation of the performance of random forest for classification problems with microarray data, and shown it to be competitive with alternative methods, without requiring any fine-tuning of parameters or pre-selection of variables. The performance of random forest without variable selection is also equivalent to that of alternative approaches that fine-tune the variable selection process (see below).

We have then examined the performance of an approach for gene selection using random forest, and compared it to alternative approaches. Our results, using both simulated and real microarray data sets, show that this method of gene selection accomplishes the proposed objectives. Our method returns very small sets of genes compared to alternative variable selection methods, while retaining predictive performance. Our method of gene selection will not return sets of genes that are highly correlated, because they are redundant. This method will be most useful under two scenarios: a) when considering the design of diagnostic tools, where having a small set of probes is often desirable; b) to help understand the results from other gene selection approaches that return many genes, so as to understand which ones of those genes have the largest signal to noise ratio and could be used as surrogates for complex processes involving many correlated genes. A backwards elimination method, precursor to the one used here, has been already used to predict breast tumor type based on chromosomic alterations [18].

We have also thoroughly examined the effects of changes in the parameters of random forest (specifically *mtry*, *ntree*, *nodesize*) and the variable selection algorithm (*se*, *fraction.dropped*). Changes in these parameters have in most cases negligible effects, suggesting that the default values are often good options, but we can make some general recommendations. Time of execution of the code increases ≈ linearly with *ntree*. Larger *ntree *values lead to slightly more stable values of variable importances, but for the data sets examined, *ntree *= 2000 or *ntree *= 5000 seem quite adequate, with further increases having negligible effects. The change in *nodesize *from 1 to 5 has negligible effects, and thus its default setting of 1 is appropriate. For the backwards elimination algorithm, the parameter *fraction.dropped *can be adjusted to modify the resolution of the number of variable selected; smaller values of *fraction.dropped *lead to finer resolution in the examination of number of genes, but to slower execution of the code. Finally, the parameter *se *has also minor effects on the results of the backwards variable selection algorithm but a value of *se *= 1 leads to slightly more stable results and smaller sets of selected genes.

In contrast to other procedures (e.g., [3,8]) our procedure does not require to pre-specify the number of genes to be used, but rather adaptively chooses the number of genes. [3] have conducted an evaluation of several gene selection algorithms, including genetic algorithms and various ranking methods; these authors show results for the Leukemia and NCI60 data sets, but the Leukemia results are not directly comparable since [3] focus on a three-class problem. They report the best results with the NCI60 data set estimated with the .632 bootstrap rule (compared to the .632+ method that we use, the .632 can be downwardly biased specially with highly overfit rules like nearest neighbor that they use – [35]). These best error rates are 0.408 for their evolutionary algorithm with 30 genes and 0.318 for 40 top-ranked genes. Using a number of genes slightly larger than us, these error rates are similar to ours; however, these are the best error rates achieved over a range of ranking methods and error rates, and not the result of a complete procedure that automatically determines the best number of genes and ranking scheme (such as our method provides). [8] conducted a comparative study of feature selection and multi-class classification. Although they use four-fold cross-validation instead of the bootstrap to assess error rates, their results for three data sets common to both studies (Srbct, Lymphoma, NCI60) are similar to, or worse than, ours. In contrast to our method, their approach pre-selects a set of 150 genes for prediction and their best error rates are those over a set of seven different algorithms and eight different rank selection methods, where no algorithm or gene selection was consistently the best. In contrast, our results with one single algorithm and gene selection method (random forest) match or outperform their results.

Recently, several approaches that adaptively select the best number of genes or features have been reported. For the Leukemia data set our method consistently returns sets of two genes, similar to [27] using an exhaustive search method, and lower than the numbers given by [42] of 3 to 25. [2] have proposed a Bayesian model averaging (BMA) approach for gene selection; comparing the results for the two common data sets between our study and theirs, in one case (Leukemia) our procedure returns a much smaller set of genes (2 vs. 15), whereas in another (Breast, 2 class) their BMA procedure returns 8 fewer genes (14 vs. 6); in contrast to BMA, however, our procedure does not require setting a limit in the maximum number of relevant genes to be selected. [43] have developed a method for gene selection and classification, LS Bound, related to least-squares SVMs; their method uses an initial pre-filtering (they choose 1000 initial genes) and is not clear how it could be applied to multi-class problems. The performance of their procedure with the leukemia data set is better than that reported by our method, but they use a total of 72 samples (the original 38 training plus the 34 validation of [44]) thus making these results hard to compare. With the colon data sets, however, their best performing results are not better than ours with a number of features that is similar to ours. [5] proposed two Bayesian classification algorithms that incorporate gene selection (though it is not clear how their algorithms can be used in multi-class problems). The results for the Leukemia data set are not comparable to ours (as they use the validation set of 34 samples), but their results for the colon data set show error rates of 0.167 to 0.242, slightly larger than ours (although these authors used random partitions with 50 training and 12 testing samples instead of the .632+ bootstrap to assess error rate), with between 8 and 15 features selected (somewhat larger than those from random forest). Finally, [31], applied both shrunken centroids and a genetic algorithm + KNN technique to the NCI60 and Srcbt data sets; their results with shrunken centroids are similar to ours with that technique, but the genetic algorithm + KNN technique used larger sets of genes (155 and 72 for the NCI60 and Srbct, respectively) than variable selection with random forest using the suggested parameters. In summary, then, our proposed procedure matches or outperforms alternative approaches for gene selection in terms of error rate and number of genes selected, without any need to fine-tune parameters or preselect genes; in addition, this method is equally applicable to two-class and multi-class problems, and has software readily available. Thus, the newly proposed method is an ideal candidate for gene selection in classification problems with microarray data.

A reviewer has alerted us to the paper by Jiang et al. [45], previously unknown to us. In fact, our approach is virtually the same as the one used by Jiang et al., with the exception that these authors recompute variable importances at each step (we do not do this in this paper, although the option is available in our code) and, more importantly, that their gene selection is based both in the OOB error, as well as the prediction error when the forest trained with one data set is applied to a second, independent, data set; thus, this approach for gene selection is not feasible when we only have one data set. Jiang et al. [45] also show the excellent performance of variable selection using random forest when applied to their data sets. The final issue addressed in this paper is instability or multiplicity of the selected sets of genes. From this point of view, the results are slightly disappointing. But so are the results of the competing methods. And so are the results of most examined methods so far with microarray data, as shown in [29] and [30] and discussed thoroughly by [27] for classification and by [28] for the related problem of the effect of threshold choice in gene selection. However, and except for the above cited papers and [6,46] and [5], this is an issue that still seems largely ignored in the microarray literature. As these papers and the statistical literature on variable selection (e.g., [40,47]) discusses, the causes of the problem are small sample sizes and the extremely small ratio of samples to variables (i.e., number of arrays to number of genes). Thus, we might need to learn to live with the problem, and try to assess the stability and robustness of our results by using a variety of gene selection features, and examining whether there is a subset of features that tends to be repeatedly selected. This concern is explicitly taken into account in our results, and facilities for examining this problem are part of our R code.

The multiplicity problem, however, does not need to result in large prediction errors. This and other papers [7,27,31,32,48,49] (see also above) show that very different classifiers often lead to comparable and successful error rates with a variety of microarray data sets. Thus, although improving prediction rates is important, when trying to address questions of biological mechanism or discover therapeutic targets, probably a more challenging and relevant issue is to identify sets of genes with biological relevance.

Two areas of future research are using random forest for the selection of potentially large sets of genes that include correlated genes, and improving the computational efficiency of these approaches; in the present work, we have used parallelization of the "embarrassingly parallelizable" tasks using MPI with the Rmpi and Snow packages [50,51] for R. In a broader context, further work is warranted on the stability properties and biological relevance of this and other gene-selection approaches, because the multiplicity problem casts doubts on the biological interpretability of most results based on a single run of one gene-selection approach.

## Conclusion

The proposed method can be used for variable selection fulfilling the objectives above: we can obtain very small sets of non-redundant genes while preserving predictive accuracy. These results clearly indicate that the proposed method can be profitably used with microarray data and is competitive with existing methods. Given its performance and availability, random forest and variable selection using random forest should probably become part of the "standard tool-box" of methods for the analysis of microarray data.

## Methods

### Simulated data sets

Data have been simulated using different numbers of classes of patients (2 to 4), number of independent dimensions (1 to 3), and number of genes per dimension (5, 20, 100). In all cases, we have set to 25 the number of subjects per class. Each independent dimension has the same relevance for discrimination of the classes. The data come from a multivariate normal distribution with variance of 1, a (within-class) correlation among genes within dimension of 0.9, and a within-class correlation of 0 between genes from different dimensions, as those are independent. The multivariate means have been set so that the unconditional prediction error rate [52] of a linear discriminant analysis using one gene from each dimension is approximately 5%. To each data set we have added 2000 random normal variates (mean 0, variance 1) and 2000 random uniform [-1,1] variates. In addition, we have generated data sets for 2, 3, and 4 classes where no genes have signal (all 4000 genes are random). For the non-signal data sets we have generated four replicate data sets for each level of number of classes. Further details are provided in the supplementary material [see Additional file 1].

### Competing methods

We have compared the predictive performance of the variable selection approach with: a) random forest without any variable selection (using mtry=number of variables
 MathType@MTEF@5@5@+=feaafiart1ev1aaatCvAUfKttLearuWrP9MDH5MBPbIqV92AaeXatLxBI9gBaebbnrfifHhDYfgasaacH8akY=wiFfYdH8Gipec8Eeeu0xXdbba9frFj0=OqFfea0dXdd9vqai=hGuQ8kuc9pgc9s8qqaq=dirpe0xb9q8qiLsFr0=vr0=vr0dc8meaabaqaciGacaGaaeqabaqabeGadaaakeaacqWGTbqBcqWG0baDcqWGYbGCcqWG5bqEcqGH9aqpdaGcaaqaaiabd6gaUjabdwha1jabd2gaTjabdkgaIjabdwgaLjabdkhaYjaaykW7cqWGVbWBcqWGMbGzcaaMc8ocbiGae8NDayNae8xyaeMae8NCaiNaemyAaKMaemyyaeMaemOyaiMaemiBaWMaemyzauMaem4Camhaleqaaaaa@4DE7@, *ntree *= 5000, *nodesize *= 1); b) three other methods that have shown good performance in reviews of classification methods with microarray data [7,31] but that do not include any variable selection (i.e., they use a number of genes decided before hand); c) two methods that carry out variable selection.

The three methods that do not carry out variable selection are:

• **Diagonal Linear Discriminant Analysis (DLDA) **DLDA is the maximum likelihood discriminant rule, for multivariate normal class densities, when the class densities have the same diagonal variance-covariance matrix (i.e., variables are uncorrelated, and for each variable, its variance is the same in all classes). This yields a simple linear rule, where a sample is assigned to the class *k *which minimizes ∑j=1p(xj−x¯kj)2/σ^j2
 MathType@MTEF@5@5@+=feaafiart1ev1aaatCvAUfKttLearuWrP9MDH5MBPbIqV92AaeXatLxBI9gBaebbnrfifHhDYfgasaacH8akY=wiFfYdH8Gipec8Eeeu0xXdbba9frFj0=OqFfea0dXdd9vqai=hGuQ8kuc9pgc9s8qqaq=dirpe0xb9q8qiLsFr0=vr0=vr0dc8meaabaqaciGacaGaaeqabaqabeGadaaakeaadaaeWaqaaiabcIcaOiabdIha4naaBaaaleaacqWGQbGAaeqaaOGaeyOeI0IafmiEaGNbaebadaWgaaWcbaGaem4AaSMaemOAaOgabeaakiabcMcaPmaaCaaaleqabaGaeGOmaidaaOGaei4la8Iafq4WdmNbaKaadaqhaaWcbaGaemOAaOgabaGaeGOmaidaaaqaaiabdQgaQjabg2da9iabigdaXaqaaiabdchaWbqdcqGHris5aaaa@43ED@, where *p *is the number of variables, *x*_*j *_is the value on variable (gene) *j *of the test sample, x¯kj
 MathType@MTEF@5@5@+=feaafiart1ev1aaatCvAUfKttLearuWrP9MDH5MBPbIqV92AaeXatLxBI9gBaebbnrfifHhDYfgasaacH8akY=wiFfYdH8Gipec8Eeeu0xXdbba9frFj0=OqFfea0dXdd9vqai=hGuQ8kuc9pgc9s8qqaq=dirpe0xb9q8qiLsFr0=vr0=vr0dc8meaabaqaciGacaGaaeqabaqabeGadaaakeaacuWG4baEgaqeamaaBaaaleaacqWGRbWAcqWGQbGAaeqaaaaa@3127@ is the sample mean of class *k *and variable (gene) *j*, and σ^j2
 MathType@MTEF@5@5@+=feaafiart1ev1aaatCvAUfKttLearuWrP9MDH5MBPbIqV92AaeXatLxBI9gBaebbnrfifHhDYfgasaacH8akY=wiFfYdH8Gipec8Eeeu0xXdbba9frFj0=OqFfea0dXdd9vqai=hGuQ8kuc9pgc9s8qqaq=dirpe0xb9q8qiLsFr0=vr0=vr0dc8meaabaqaciGacaGaaeqabaqabeGadaaakeaacuaHdpWCgaqcamaaDaaaleaacqWGQbGAaeaacqaIYaGmaaaaaa@30FD@ is the (pooled) estimate of the variance of gene *j *[7]. In spite of its simplicity and its somewhat unrealistic assumptions (independent multivariate normal class densities), this method has been found to work very well.

• **K nearest neighbor (KNN) **KNN is a non-parametric classification method that predicts the sample of a test case as the majority vote among the k nearest neighbors of the test case [15,16]. To decide on "nearest" we use, as in [7], the Euclidean distance. The number of neighbors used (k) is chosen by cross-validation as in [7]: for a given training set, the performance of the KNN for values of *k *in {1, 3, 5, ..., 21} is determined by cross-validation, and the *k *that produces the smallest error is used.

• **Support Vector Machines (SVM) **SVM are becoming increasingly popular classifiers in many areas, including microarrays [53-55]. SVM (with linear kernel, as used here) try to find an optimal separating hyperplane between the classes. When the classes are linearly separable, the hyperplane is located so that it has maximal margin (i.e., so that there is maximal distance between the hyperplane and the nearest point of any of the classes) which should lead to better performance on data not yet seen by the SVM. When the data are not separable, there is no separating hyperplane; in this case, we still try to maximize the margin but allow some classification errors subject to the constraint that the total error (distance from the hyperplane in the "wrong side") is less than a constant. For problems involving more than two classes there are several possible approaches; the one used here is the "one-against-one" approach, as implemented in "libsvm" [56]. Reviews and introductions to SVM can be found in [16,57].

For each of these three methods we need to decide which of the genes will be used to build the predictor. Based on the results of [7] we have used the 200 genes with the largest *F*-ratio of between to within groups sums of squares. [7] found that, for the methods they considered, 200 genes as predictors tended to perform as well as, or better than, smaller numbers (30, 40, 50 depending on data set). The three methods that include gene selection are:

• **Shrunken centroids (SC) **The method of "nearest shrunken centroids" was originally described in [33]. It uses "de-noised" versions of centroids to classify a new observations to the nearest centroid. The "de-noising" is achieved using soft-thresholding or penalization, so that for each gene, class centroids are shrunken towards the overall centroid. This method is very similar to a DLDA with shrinkage on the centroids. The optimal amount of shrinkage can be found with cross-validation, and used to select the number of genes to retain in the final classifier. We have used two different approaches to determine the best number of features.

     - **SC.l**: we choose the number of genes that minimizes the cross-validated error rate and, in case of several solutions with minimal error rates, we choose the one with largest likelihood.

     - **SC.s**: we choose the number of genes that minimizes the cross-validated error rate and, in case of several solutions with minimal error rates, we choose the one with smallest number of genes (larger penalty).

• **Nearest neighbor + variable selection (NN.vs) **We first rank all genes based on their F-ratio, and then run a Nearest Neighbor classifier (KNN with K = 1; using N = 1 is often a successful rule [15,16]) on all subsets of variables that result from eliminating 20% of the genes (the ones with the smallest F-ratio) used in the previous iteration. The final number of genes is the one that leads to the smallest cross-validated error rate.

The ranking of the genes using the F-ratio is done without using the left-out sample. In other words, for a given data set, we first divide it 10 samples of about the same size; then, we repeat 10 times the following:

a) Exclude sample "i", the "left-out" sample.

b) Using the other 9 samples, rank the genes using the F-ratio

c) Predict the values for the left-out sample at each of the pre-specified numbers of genes (subsets of genes), using the genes as given by the ranking in the previous step.

At the end of the 10 iterations, we average the error rate over the 10 left-out samples, and obtain the average cross-validated error rate at each number of genes. These estimates are not affected by "selection bias" [34,37] as the error rate is obtained from the left-out samples, but the left-out samples are not involved in the ranking of genes. (Note, that using error rates affected by selection bias to select the optimal number of genes is not necessarily a bad procedure from the point of view of selecting the final number of genes; see [38]).

Even if we use, as here, error rates not affected by selection bias, using that cross-validated error rate as the estimated error rate of the rule would lead to a biased-down error rate (for reasons analogous to those leading to selection bias). Thus, we do not use these error rates in the tables, but compute the estimated prediction error rate of the rule using the .632+ bootstrap method.

This type of approach, in its many variants (changing both the classifier and the ordering criterion) is popular in many microarray papers; a recent example is [10], and similar general strategies are implemented in the program Tnasas [58].

### Software and data sets

All simulations and analyses were carried out with R [59], using packages randomForest (from A. Liaw and M. Wiener) for random forest, e1071 (E. Dimitriadou, K. Hornik, F. Leisch, D. Meyer, and A. Weingessel) for SVM, class (B. Ripley and W. Venables) for KNN, PAM [33] for shrunken centroids, and geSignatures (by R.D.-U.) for DLDA.

The microarray and simulated data sets are available from the supplementary material web page [60].

## Availability and requirements

Our procedure is available both as an R package (varSelRF) and as a web-based application (GeneSrF).

### varSelRF

**Project name: **varSelRF.

**Project home page: **

**Operating system(s): **Linux and UNIX, Windows, MacOS.

**Programming language: **R.

**Other requirements: **Linux/UNIX and LAM/MPI for parallelized computations.

**License: **GNU GPL 2.0 or newer.

**Any restrictions to use by non-academics: **None.

### GeneSrF

**Project name: **GeneSrF

**Project home page: **

**Operating system(s): **Platform independent.

**Programming language: **Python and R.

**Other requirements: **A web browser.

**License: **Not applicable. Access non-restricted.

**Any restrictions to use by non-academics: **None.

## List of abbreviations

• DLDA: Diagonal linear discriminant analysis.

• KNN: K-nearest neighbor.

• NN: nearest neighbor (like KNN with *K *= 1).

• NN.vs: Nearest neighbor with variable selection.

• OOB error: Out-of-bag error; error rate from samples not used in the construction of a given tree.

• SC.l: Shrunken centroids with minimization of error and maximization of likelihood if ties.

• SC.s: Shrunken centroids with minimization of error and minimization of features if ties.

• SVM: Support vector machine.

• *mtry*: Number of input variables tried at each split by random forest.

• *mtryFactor*: Multiplicative factor of the default *mtry *(number⋅of⋅genes
 MathType@MTEF@5@5@+=feaafiart1ev1aaatCvAUfKttLearuWrP9MDH5MBPbIqV92AaeXatLxBI9gBaebbnrfifHhDYfgasaacH8akY=wiFfYdH8Gipec8Eeeu0xXdbba9frFj0=OqFfea0dXdd9vqai=hGuQ8kuc9pgc9s8qqaq=dirpe0xb9q8qiLsFr0=vr0=vr0dc8meaabaqaciGacaGaaeqabaqabeGadaaakeaadaGcaaqaaiabd6gaUjabdwha1jabd2gaTjabdkgaIjabdwgaLjabdkhaYjabgwSixlabd+gaVjabdAgaMjabgwSixlabdEgaNjabdwgaLjabd6gaUjabdwgaLjabdohaZbWcbeaaaaa@4332@)

• *nodesize*: Minimum size of the terminal nodes of the trees in a random forest.

• *ntree*: Number of trees used by random forest.

• *s.e. *0 and *s.e. *1: "0 s.e." (respectively "1 s.e.") rule for choosing the best solution for gene selection (how far the selected solution can be from the minimal error solution).

## Authors' contributions

R.D-U developed the gene selection methodology, designed and carried out the comparative study, wrote the code, and drafted the manuscript. S.A.A. brought up the biological problem that prompted the methodological development and verified and provided discussion on the methodology, and co-authored the manuscript. Both authors read and approved the manuscript.

## Supplementary Material

Additional File 1 A PDF file with additional results, showing error rates and stability for simulated data under various parameters, as well as error rates and stabilities for the real microarray data with other parameters, and further details on the data sets, simulations, and alternative methods. Click here for file

Additional File 2 A PDF file with additional plots of OOB error rate vs. *mtry *for both simulated data and real data under other parameters. Click here for file

Additional File 3 Source code for the R package varSelRF. This is a compressed (tar.gz) file ready to be installed with the usual R installation procedure under Linux/UNIX. Additional formats are available from CRAN [68], the Comprehensive R Archive Network. Click here for file

## Figures and Tables

**Table 1:** Main characteristics of the microarray data sets used

Dataset	Original ref.	Genes	Patients	Classes
Leukaemia	[44]	3051	38	2
Breast	[9]	4869	78	2
Breast	[9]	4869	96	3
NCI 60	[61]	5244	61	8
Adenocarcinoma	[62]	9868	76	2
Brain	[63]	5597	42	5
Colon	[64]	2000	62	2
Lymphoma	[65]	4026	62	3
Prostate	[66]	6033	102	2
Srbct	[67]	2308	63	4

**Table 2:** Error rates (estimated using the 0.632+ bootstrap method with 200 bootstrap samples) for the microarray data sets using different methods. The results shown for variable selection with random forest used *ntree *= 2000, *fraction.dropped *= 0.2, *mtryFactor *= 1. Note that the OOB error used for variable selection *is not *the error reported in this table; the error rate reported is obtained using bootstrap on the complete variable selection process. The column "no info" denotes the minimal error we can make if we use no information from the genes (i.e., we always bet on the most frequent class).

Data set	no info	SVM	KNN	DLDA	SC.l	SC.s	NN.vs	random forest	random forest var.sel.	
									s.e. 0	s.e. 1
Leukemia	0.289	0.014	0.029	0.020	0.025	0.062	0.056	0.051	0.087	0. 075
Breast 2 cl.	0.429	0.325	0.337	0.331	0.324	0.326	0.337	0.342	0.337	0. 332
Breast 3 cl.	0.537	0.380	0.449	0.370	0.396	0.401	0.424	0.351	0.346	0. 364
NCI 60	0.852	0.256	0.317	0.286	0.256	0.246	0.237	0.252	0.327	0.353
Adenocar.	0.158	0.203	0.174	0.194	0.177	0.179	0.181	0.125	0.185	0. 207
Brain	0.762	0.138	0.174	0.183	0.163	0.159	0.194	0.154	0.216	0. 216
Colon	0.355	0.147	0.152	0.137	0.123	0.122	0.158	0.127	0.159	0. 177
Lymphoma	0.323	0.010	0.008	0.021	0.028	0.033	0.04	0.009	0.047	0. 042
Prostate	0.490	0.064	0.100	0.149	0.088	0.089	0.081	0.077	0.061	0. 064
Srbct	0.635	0.017	0.023	0.011	0.012	0.025	0.031	0.021	0.039	0.038

**Table 3:** Stability of variable (gene) selection evaluated using 200 bootstrap samples. "# Genes": number of genes selected on the original data set. "# Genes boot.": median (1st quartile, 3rd quartile) of number of genes selected from on the bootstrap samples. "Freq. genes": median (1st quartile, 3rd quartile) of the frequency with which each gene in the original data set appears in the genes selected from the bootstrap samples. Parameters for backwards elimination with random forest: *mtryFactor *= 1, *s.e. *= 0, *ntree *= 2000, *ntreelterat *= 1000, *fraction.dropped *= 0.2.

Data set	Error	# Genes	# Genes boot.	Freq. genes
**Backwards elimination of genes from random forest**				

*s.e. *= 0				

Leukemia	0.087	2	2 (2, 2)	0.38 (0.29, 0.48)^1 ^
Breast 2 cl.	0.337	14	9 (5, 23)	0.15 (0.1, 0.28)
Breast 3 cl.	0.346	110	14 (9, 31)	0.08 (0.04, 0.13)
NCI 60	0.327	230	60 (30, 94)	0.1 (0.06, 0.19)
Adenocar.	0.185	6	3 (2, 8)	0.14 (0.12, 0.15)
Brain	0.216	22	14 (7, 22)	0.18 (0.09, 0.25)
Colon	0.159	14	5 (3, 12)	0.29 (0.19, 0.42)
Lymphoma	0.047	73	14 (4, 58)	0.26 (0.18, 0.38)
Prostate	0.061	18	5 (3, 14)	0.22 (0.17, 0.43)
Srbct	0.039	101	18 (11, 27)	0.1 (0.04, 0.29)

*s.e. *= 1				

Leukemia	0.075	2	2 (2, 2)	0.4 (0.32, 0.5)^1 ^
Breast 2 cl.	0.332	14	4 (2, 7)	0.12 (0.07, 0.17)
Breast 3 cl.	0.364	6	7 (4, 14)	0.27 (0.22, 0.31)
NCI 60	0.353	24	30 (19, 60)	0.26 (0.17, 0.38)
Adenocar.	0.207	8	3 (2, 5)	0.06 (0.03, 0.12)
Brain	0.216	9	14 (7, 22)	0.26 (0.14, 0.46)
Colon	0.177	3	3 (2, 6)	0.36 (0.32, 0.36)
Lymphoma	0.042	58	12 (5, 73)	0.32 (0.24, 0.42)
Prostate	0.064	2	3 (2, 5)	0.9 (0.82, 0.99)^1 ^
Srbct	0.038	22	18 (11, 34)	0.57 (0.4, 0.88)

**Alternative approaches**				

SC.s				

Leukemia	0.062	82^2 ^	46 (14, 504)	0.48 (0.45, 0.59)
Breast 2 cl.	0.326	31	55 (24, 296)	0.54 (0.51, 0.66)
Breast 3 cl.	0.401	2166	4341 (2379, 4804)	0.84 (0.78, 0.88)
NCI 60	0.246	5118^3 ^	4919 (3711, 5243)	0.84 (0.74, 0.92)
Adenocar.	0.179	0	9 (0, 18)	NA (NA, NA)
Brain	0.159	4177	1257 (295, 3483)	0.38 (0.3, 0.5)
Colon	0.122	15	22 (15, 34)	0.8 (0.66, 0.87)
Lymphoma	0.033	2796	2718 (2030, 3269)	0.82 (0.68, 0.86)
Prostate	0.089	4	3 (2, 4)	0.72 (0.49, 0.92)
Srbct	0.025	37^4 ^	18 (12, 40)	0.45 (0.34, 0.61)

NN.vs				

Leukemia	0.056	512	23 (4, 134)	0.17 (0.14, 0.24)
Breast 2 cl.	0.337	88	23 (4, 110)	0.24 (0.2, 0.31)
Breast 3 cl.	0.424	9	45 (6, 214)	0.66 (0.61, 0.72)
NCI 60	0.237	1718	880 (360, 1718)	0.44 (0.34, 0.57)
Adenocar.	0.181	9868	73 (8, 1324)	0.13 (0.1, 0.18)
Brain	0.194	1834	158 (52, 601)	0.16 (0.12, 0.25)
Colon	0.158	8	9 (4, 45)	0.57 (0.45, 0.72)
Lymphoma	0.04	15	15 (5, 39)	0.5 (0.4, 0.6)
Prostate	0.081	7	6 (3, 18)	0.46 (0.39, 0.78)
Srbct	0.031	11	17 (11, 33)	0.7 (0.66, 0.85)

^1 ^Only two genes are selected from the complete data set; the values are the actual frequencies of those two genes.

^2 ^[33] select 21 genes after visually inspecting the plot of cross-validation error rate vs. amount of shrinkage and number of genes. Their procedure is hard to automate and thus it is very difficult to obtain estimates of the error rate of their procedure.

^3 ^[31] report obtaining more than 2000 genes when using shrunken centroids with this data set and show that the minimum error rate is achieved with about 5000 genes.

^4 ^[33] select 43 genes. The difference is likely due to differences in the random partitions for cross-validation. Repeating 100 times the gene selection process with the full data set the median, 1st quartile, and 3rd quartile of the number of selected genes are 13, 8, and 147. For these data, [31] obtain 72 genes with shrunken centroids, which also falls within the above interval.
