# Supplementary material for: Gene selection and classification of microarray data using random forest
Source: BMC Bioinformatics. 2006 Jan 6;7:3. doi: 10.1186/1471-2105-7-3 (PMC1363357; doi:10.1186/1471-2105-7-3)
Supplement: Additional File 1 — A PDF file with additional results, showing error rates and stability for simulated data under various parameters, as well as error rates and stabilities for the real microarray data with other parameters, and further details on the data sets, simulations, and alternative methods. [file 1471-2105-7-3-S1.pdf]

Supplementary material for "Gene selection and classification of microarray data using random forest"

Ramón Díaz-Uriarte, Sara Alvarez de Andrés
Bioinformatics Unit, Cytogenetics Unit
Spanish National Cancer Center (CNIO)
Melchor Fernández Almagro 3
Madrid, 28029
Spain.
rdiaz@cnio.es
http://ligarto.org/rdiaz

1 Variable importance from random forest

Random forest returns several measures of variable importance. The most reliable measure of variable importance is based on the decrease of classification accuracy when values of a variable in a node of a tree are permuted randomly (Breiman, 2001; Bureau *et al.*, 2003; Remlinger, 2004). This measure is sometimes reported as such, and sometimes it is reported after scaling it, or dividing by a quantity somewhat analogous to its standard error ("somewhat analogous" because the data used to obtain that "standard error" are not truly independent, and thus the true standard error can be severely underestimated). We use in this paper the unscaled importance measure, because it allows us to compare directly runs with different settings of *ntree* and *mtry* (in contrast, scaled importances increase monotonically as we increase the value of *ntree*).

2 Microarray data sets

The data sets **Colon**, **Prostate**, **Lymphoma**, **SRBCT** and **Brain** were obtained, as binary R files, from Marcel Dettling's web site `http://stat.ethz.ch/~dettling/bagboost.html`. The data sets and their preprocessing are fully described in Dettling & Bühlmann (2002).

Leukemia dataset From Golub *et al.* (1999). The original data, from an Affymetrix chip, comprises 6817 genes, but after filtering as done by the authors we are left with 3051 genes. Filtering and preprocessing is described in the original paper and in Dudoit *et al.* (2002). We used the training data set of 38 cases (27 ALL and 11 AML) in the original paper (the observations in the "test set" are from a different lab and were collected at different times). This data set is available from [`http://www-genome.wi.mit.edu/cgi-bin/cancer/datasets.cgi`] and also from the Bioconductor package multtest ([`http://www.bioconductor.org`]).

Adenocarcinoma dataset From Ramaswamy *et al.* (2003). We used the data from the 12 metastatic tumors and 64 primary tumors. The original data set included 16063 genes from Affymetrix chips. The data (DatasetA_Tum_vsMet.res), downloaded from [`http://www-genome.wi.mit.edu/cgi-bin/cancer/`], had already been rescaled by the authors. We took the subset of 9376 genes according to the UniGene mapping, thresholded the data, and filtered by variation as explained by the authors. The final data set contains 9868 clones (several genes were represented by more than one clone); of these, 196 had constant values over all individuals.

NCI 60 dataset From Ross *et al.* (2000). The data, from cDNA arrays, can be obtained from [`http://genome-www.stanford.edu/sutech/download/nci60/index.html`]. The raw data we used, which is the same as the data used in Dettling & Bühlmann (2003); Dudoit *et al.* (2002), is the one in the file "figure3.cdt". As in Dettling & Bühlmann (2003); Dudoit *et al.* (2002) we filtered out genes with more than two missing observations and we also eliminated, because of small sample size, the two prostate cell line observations and the unknown observation. After filtering, we were left with a 61 x 5244 matrix, corresponding to eight different tumor types (note that, as done by previous authors, we did not average the two observations with triplicate hybridizations). As in Dudoit *et al.* (2002) we used 5-nearest neighbor imputation of missing data using the program GEPAS (Herrero *et al.*, 2003) (`http://gepas.bioinfo.cnio.es/cgi-bin/preprocess`); unlike Dudoit *et al.* (2002), however, we measured gene similarity using Euclidean distance from the genes with complete data, instead of correlation: Troyanskaya *et al.* (2001) found Euclidean distance to be an appropriate metric. Finally, as in (Dudoit *et al.*, 2002, p. 82) gene expression data were standardized so that arrays had mean 0 and variance 1 across variables (genes).

Breast cancer dataset From van 't Veer *et al.* (2002). The data were downloaded from [`http://www.rii.com/publications/2002/vantveer.htm`] (we used the files ArrayData_less_than_5yr.zip, ArrayData_greater_than_5yr.zip, ArrayData_BRCA1.zip, corresponding to 34 patients that developed distant metastases within 5 years, 44 that remained disease-free for over 5 years, and 18 with BRCA1 germline mutations and 2 with BRCA2 mutations). As did by the authors, we selected only the genes that were "significantly regulated" (see their definition in the paper and supplementary material), which resulted in a total of 4869 clones. Because of the small sample size, we excluded the 2 patients with the BRCA2 mutation. We used 5-nearest neighbor imputation for the missing data, as for the NCI 60 data set. Finally, we excluded from the analyses the 10th subject from the set that developed metastases in less than 5 years (sample 54, IRI000045837, in the original data files), because it had 10896 missing values out of the original 24481 clones, and was an outstanding outlying point both before and after imputation. The breast cancer dataset was used both for two

class comparison (those that developed metastases within 5 years vs. those that remain metastases free after 5 years) and for three group comparisons.

Tab-separated text files for these data sets are available from [`http://ligarto.org/rdiaz/Papers/rfVS/randomForestVarSel.html`].

3 Generation of simulated data

We have simulated data under different number of classes of patients (2, 3, 4), number of independent dimensions (1 to 3), and number of genes per dimension (5, 20, 100). In all cases, the number of subjects per class has been set to 25 (a number which is similar to, or smaller than, that of many microarray studies). The data have been simulated from a multivariate normal distribution. All "genes" have a variance of 1, and the correlation between genes within a dimension is 0.9, whereas the correlation between genes among dimensions is 0. In other words, the variance-covariance matrix is a block-diagonal matrix as:

$$\Sigma = \begin{bmatrix} \mathbf{a} & \mathbf{0} & \mathbf{0} & \dots & \mathbf{0} \\ \mathbf{0} & \mathbf{a} & \mathbf{0} & \dots & \mathbf{0} \\ \vdots & \vdots & \vdots & \vdots & \vdots \\ \mathbf{0} & \mathbf{0} & \mathbf{0} & \dots & \mathbf{a} \end{bmatrix},$$

where

$$\mathbf{a} = \begin{bmatrix} 1 & 0.9 & \dots & 0.9 \\ 0.9 & 1 & \dots & 0.9 \\ \vdots & \vdots & \vdots & \vdots \end{bmatrix}.$$

The class means have been set so that the unconditional prediction error rate (see McLachlan (1992)) of a DLDA using one gene from each dimension is approximately 5%; and each dimension has the same relevance in separation. Specifically, the class means used are:

- One dimension:
  - Two classes: $\mu_1 = -1.65, \mu_2 = 1.65$.
  - Three classes: $\mu_1 = -3.58, \mu_2 = 0, \mu_3 = 3.58$.
  - Four classes: $\mu_1 = -3.7, \mu_2 = 0, \mu_3 = 3.7, \mu_4 = 7.4$.
- Two dimensions:
  - Two classes:$\mu_1 = [-1.18, -1.18], \mu_2 = [1.18, 1.18]$.
  - Three classes: $\mu_1 = [0, 0], \mu_2 = [3.88\cos(15), 3.88\sin(15)]$, $\mu_3 = [3.88\cos(75), 3.88\sin(75)]$.
  - Four classes: $\mu_1 = [1, 1], \mu_2 = [4.95, 1]$, $\mu_3 = [1, 4.95], \mu_4 = [4.95, 4.95]$.
- Three dimensions:
  - Two classes:$\mu_1 = [-0.98, -0.98, -0.98], \mu_2 = [0.98, 0.98, 0.98]$.
  - Three classes: $\mu_1 = [2.76, 0, 0], \mu_2 = [0, 2.76, 0]$, $\mu_3 = [0, 0, 2.76]$.
  - Four classes: $\mu_1 = [2.96, 0, 0], \mu_2 = [0, 2.96, 0]$, $\mu_3 = [0, 0, 2.96], \mu_4 = [2.96, 2.96, 2.96]$

After the genes that belong to the dimensions are generated, we add another 2000 $\mathcal{N}(0, 1)$ variables and another 2000 $\mathcal{U}[-1, 1]$ variables to the matrix of "genes". For each combination of number of dimensions * number of classes * number of genes per dimension we generate 4 data sets.

All simulated data files used are available (in R format)from [`http://ligarto.org/rdiaz/Papers/rfVS/randomForestVarSel.html`].

4 Choosing mtry and ntree

Figure "error.vs.mtry.pdf" shows the OOB error rate plotted against the mtry factor for different ntree and nodesize. The mtry factor = $\{0, 0.05, 0.1, 0.17, 0.25, 0.33, 0.5, 0.75, 0.8, 1, 1.15, 1.33, 1.5, 2, 3, 4, 5, 6, 8, 10, 13\}$, where an mtry factor of 0 means mtry = 1 variable. Values of ntree = $1000, 2000, 5000, 10000, 40000$ for the simulated data (both with and without signal) and ntree = $1000, 2000, 5000, 10000, 20000, 40000$ for the real microarray data sets. The values of nodesize were 1 (the default) and 5.

5 Backwards elimination of variables using OOB error

5.1 Simulated data

Classes	# Vars	Error rate
	94 (10, 24, 75)	0.527
2	148 (15, 38, 94)	0.478
	19 (12, 30, 75)	0.461
	38 (12, 38, 94)	0.502
	118 (19, 48, 94)	0.676
3	94 (24, 48, 75)	0.695
	48 (24, 48, 75)	0.705
	148 (19, 48, 94)	0.67
	75 (28, 60, 94)	0.756
4	94 (30, 48, 94)	0.755
	94 (30, 60, 94)	0.757
	118 (38, 60, 94)	0.745

Table 1: `simplify.no.signal.02` Number of variables selected and error rate (estimated using the .632+ bootstrap method, with 200 bootstrap samples) from simulated data without signal. Results shown for four replicates of each condition. Values in parenthesis are the 25th percentile, median, and 75th percentile of the number of variables selected when running the procedure on the bootstrap samples. The parameters used where $fraction.dropped = 0.2, ntree = 2000, ntreeIterat = 1000, nodesize = 1, mtryFactor = 1, seRule = 1$. The error rates estimated are comparable to the error rates from always betting on the most common class (in this case all are equiprobable, and those error rates correspond to 50% in the 2 class case, 66% in the 3 class case and 75% in the 4 class case).

Classes	# Vars	Error rate
	65 (5, 33, 65)	0.523
2	65 (9, 33, 131)	0.461
	65 (17, 33, 131)	0.454
	33 (17, 65, 131)	0.507
	263 (33, 65, 131)	0.667
3	263 (33, 49, 131)	0.692
	131 (33, 65, 131)	0.701
	131 (33, 65, 131)	0.67
	131 (33, 65, 131)	0.756
4	131 (33, 65, 131)	0.758
	263 (33, 65, 131)	0.754
	263 (33, 65, 131)	0.751

Table 2: `simplify.no.signal.05` Number of variables selected and error rate (estimated using the .632+ bootstrap method, with 200 bootstrap samples) from simulated data without signal. Results shown for four replicates of each condition. Values in parenthesis are the 25th percentile, median, and 75th percentile of the number of variables selected when running the procedure on the bootstrap samples. The parameters used where $fraction.dropped = 0.5, ntree = 5000, nodesize = 1, mtryFactor = 1, seRule = 1$. Recall that, with $fraction.dropped = 0.5$, we eliminate 50% of the variables at each iteration (see text), which explains the set of values 33, 65, 131, 263 (number of variables in step t = number of variables in step $t + 1$ - round (number of variables in step $t + 1$ * 0.5)). The error rates estimated are comparable to the error rates from always betting on the most common class (in this case all are equiprobable, and those error rates correspond to 50% in the 2 class case, 66% in the 3 class case and 75% in the 4 class case).

Classes	Dimensions	Genes/dimension	# Vars	Error rate
2	1	5	141[1] (2, 2, 3)	0.023
2	1	20	2 (2, 2, 3)	0.054
2	1	100	5 (2, 2, 3)	0.032
2	2	5	841[2] (2, 3, 96)	0.052
2	2	20	4 (2, 2, 5)	0.058
2	2	100	3 (2, 2, 3)	0.086
2	3	5	5 (3, 17, 188)	0.121
2	3	20	5 (2, 5, 178)	0.083
2	3	100	3 (2, 2, 3)	0.102
3	1	5	3 (2, 2, 3)	0.014
3	1	20	24[3] (2, 3, 5)	0.067
3	1	100	5 (2, 2, 3)	0.051
3	2	5	24[4] (3, 5, 176)	0.066
3	2	20	434[5] (3, 5, 10)	0.062
3	2	100	30[6] (3, 4, 8)	0.022
3	3	5	1645[7] (6, 42, 276)	0.039
3	3	20	8 (3, 8, 19)	0.064
3	3	100	3 (3, 5, 9)	0.061
4	1	5	840[8] (2, 7, 220)	0.031
4	1	20	2 (2, 2, 4)	0.046
4	1	100	2 (2, 2, 3)	0.04
4	2	5	8 (2, 4, 8)	0.018
4	2	20	6 (2, 3, 5)	0.024
4	2	100	10 (4, 8, 12)	0.04
4	3	5	91[9] (12, 114, 276)	0.058
4	3	20	2 (4, 8, 24)	0.092
4	3	100	50[10] (3, 7, 17)	0.072

[1] The 25th percentile, median, and 75th percentile of number of variables selected when running on another 10 data sets (generated with the same parameters) were: $8.25, 155.00, 261.30$.
[2] $5, 38, 134$.
[3] $2.00, 2.00, 2.75$.
[4] $8.25, 101.50, 241.50$.
[5] $3, 3, 5$.
[6] $3.25, 6.00, 8.00$.
[7] $16.0, 52.0, 591.8$.
[8] $7.0, 46.0, 107.3$.
[9] $5.25, 15.00, 235.50$.
[10] $3.25, 4.50, 6.00$.

Table 3: `simplify.signal.02` Number of variables selected and error rate (estimated using the .632+ bootstrap method, with 200 bootstrap samples) from simulated data with signal. Values in parenthesis are the 25th percentile, median, and 75th percentile of the number of variables selected when running the procedure on the bootstrap samples. The parameters used where $fraction.dropped = 0.2, ntree = 2000, ntreeIterat = 1000, nodesize = 1, mtryFactor = 1, seRule = 1$

Classes	Dimensions	Genes/dimension	# Vars	Error rate
2	1	5	2 (2, 2, 2)	0.023
2	1	20	2 (2, 2, 3)	0.051
2	1	100	17 (2, 2, 3)	0.033
2	2	5	2 (2, 2, 7)	0.048
2	2	20	2 (2, 2, 3)	0.06
2	2	100	3 (2, 2, 3)	0.089
2	3	5	125[1] (2, 7, 125)	0.129
2	3	20	2030[2] (2, 3, 253)	0.088
2	3	100	3 (2, 2, 3)	0.111
3	1	5	2 (2, 2, 3)	0.01
3	1	20	2 (2, 2, 3)	0.066
3	1	100	2 (2, 2, 3)	0.052
3	2	5	3 (3, 7, 15)	0.064
3	2	20	253[3] (3, 7, 15)	0.063
3	2	100	9 (2, 5, 9)	0.024
3	3	5	501[4] (7, 47, 251)	0.036
3	3	20	7 (3, 11, 31)	0.062
3	3	100	5 (3, 5, 9)	0.058
4	1	5	7 (2, 3, 63)	0.032
4	1	20	3 (2, 2, 3)	0.045
4	1	100	2 (2, 2, 2)	0.038
4	2	5	2 (2, 7, 15)	0.017
4	2	20	2 (2, 3, 7)	0.024
4	2	100	9 (9, 17, 33)	0.036
4	3	5	15 (15, 63, 251)	0.059
4	3	20	3 (3, 7, 31)	0.091
4	3	100	17 (3, 9, 33)	0.075

[1] The 25th percentile, median, and 75th percentile of number of variables selected when running on another 10 data sets (generated with the same parameters) were: $15, 47, 204$.
[2] $2, 2, 193$.
[3] $2, 5, 27$.
[4] $15, 47, 251$.

Table 4: `simplify.signal.05` Number of variables selected and error rate (estimated using the .632+ bootstrap method, with 200 bootstrap samples) from simulated data with signal. Values in parenthesis are the 25th percentile, median, and 75th percentile of the number of variables selected when running the procedure on the bootstrap samples. The parameters used where $fraction.dropped = 0.5, ntree = 5000, ntreeIterat = 5000, nodesize = 1, mtryFactor = 1, seRule = 1$

5.2 Real microarray data sets

Data set	Error rate	# Vars	# Vars bootstrap	Freq. vars
mtry factor = 1, s.e. = 0, ntree = 5000				
Leukemia	0.074	2	2 (2, 2)	0.42 (0.32, 0.5)[1]
Breast 2 cl.	0.337	39	9 (3, 19)	0.14 (0.1, 0.19)
Breast 3 cl.	0.339	39	19 (9, 39)	0.2 (0.14, 0.31)
NCI 60	0.315	327	81 (41, 81)	0.1 (0.05, 0.18)
Adenocar.	0.187	5	3 (2, 9)	0.06 (0.06, 0.2)
Brain	0.211	11	21 (11, 43)	0.29 (0.26, 0.4)
Colon	0.155	15	7 (3, 15)	0.3 (0.25, 0.4)
Lymphoma	0.037	63	15 (6, 63)	0.36 (0.29, 0.47)
Prostate	0.059	11	5 (2, 23)	0.41 (0.28, 0.5)
Srbct	0.038	19	19 (19, 37)	0.76 (0.56, 0.92)
mtry factor = 13, s.e. = 0, ntree = 5000				
Leukemia	0.09	2	2 (2, 2)	0.4 (0.29, 0.52)[1]
Breast 2 cl.	0.334	39	9 (4, 39)	0.14 (0.09, 0.22)
Breast 3 cl.	0.364	77	19 (5, 39)	0.12 (0.09, 0.16)
NCI 60	0.353	81	81 (41, 81)	0.28 (0.2, 0.4)
Adenocar.	0.224	9	5 (2, 9)	0.14 (0.12, 0.16)
Brain	0.202	21	21 (11, 43)	0.28 (0.22, 0.46)
Colon	0.171	7	7 (2, 15)	0.45 (0.38, 0.49)
Lymphoma	0.036	63	63 (15, 125)	0.46 (0.35, 0.57)
Prostate	0.066	755	47 (5, 755)	0.18 (0.12, 0.27)
Srbct	0.042	19	37 (37, 73)	0.84 (0.66, 0.98)
mtry factor = 1, s.e. = 1, ntree = 5000				
Leukemia	0.091	2	2 (2, 2)	0.37 (0.29, 0.46)[1]
Breast 2 cl.	0.344	19	3 (2, 6)	0.08 (0.05, 0.13)
Breast 3 cl.	0.376	3	9 (3, 19)	0.31 (0.27, 0.34)
NCI 60	0.35	21	41 (21, 81)	0.34 (0.19, 0.42)
Adenocar.	0.202	2	2 (2, 3)	0.17 (0.16, 0.18)[1]
Brain	0.205	11	21 (11, 43)	0.34 (0.28, 0.48)
Colon	0.172	7	2 (2, 3)	0.29 (0.19, 0.3)
Lymphoma	0.032	125	31 (7, 125)	0.31 (0.24, 0.46)
Prostate	0.061	2	3 (2, 11)	0.9 (0.8, 1)[1]
Srbct	0.03	73	37 (19, 37)	0.36 (0.2, 0.58)
mtry factor = 13, s.e. = 1, ntree = 5000				
Leukemia	0.081	2	2 (2, 2)	0.45 (0.32, 0.6)[1]
Breast 2 cl.	0.353	5	3 (2, 9)	0.28 (0.16, 0.32)
Breast 3 cl.	0.392	39	5 (3, 9)	0.08 (0.05, 0.15)
NCI 60	0.414	81	41 (21, 41)	0.13 (0.08, 0.24)
Adenocar.	0.227	5	2 (2, 3)	0.14 (0.08, 0.14)
Brain	0.201	11	21 (21, 43)	0.33 (0.27, 0.58)
Colon	0.193	3	2 (2, 7)	0.31 (0.25, 0.34)
Lymphoma	0.042	63	31 (7, 125)	0.38 (0.29, 0.5)
Prostate	0.072	2	5 (2, 23)	0.96 (0.92, 1)[1]
Srbct	0.042	19	37 (37, 73)	0.8 (0.68, 0.97)

[1] Since there are only two variables, the values here are the actual frequencies of those two variables, not the 25th and 75th percentiles.

Table 5: `stability-5000` Error rate and stability of results of backwards elimination of variables using OOB error, evaluated using 200 bootstrap samples. Results for $fraction.dropped = 0.5, ntree = 5000, ntreeIterat = 5000$. Error rate is the error rate estimated using 0.632+ bootstrap method. "# Vars" denotes the number of variables selected on the original data set. "# Vars bootstrap" shows the median (1st quartile, 3rd quartile) number of variables selected when the procedure is run on the bootstrap samples. "Freq. vars" is the median (1st quartile, 3rd quartile) of the frequency with which each variable in the original data set appears in the variables selected when the procedure is run on the bootstrap samples.

Data set	Error rate	# Vars	# Vars bootstrap	Freq. vars
mtry factor = 1, s.e. = 0, ntree = 20000				
Leukemia	0.076	2	2 (2, 2)	0.44 (0.28, 0.61)[1]
Breast 2 cl.	0.325	19	9 (3, 19)	0.18 (0.14, 0.24)
Breast 3 cl.	0.342	39	19 (9, 39)	0.21 (0.14, 0.32)
NCI 60	0.325	327	81 (41, 81)	0.12 (0.07, 0.19)
Adenocar.	0.188	9	3 (2, 9)	0.12 (0.1, 0.19)
Brain	0.196	11	21 (11, 43)	0.34 (0.25, 0.54)
Colon	0.174	15	3 (2, 7)	0.23 (0.18, 0.34)
Lymphoma	0.036	125	15 (7, 125)	0.25 (0.18, 0.37)
Prostate	0.059	23	11 (3, 23)	0.28 (0.23, 0.48)
Srbct	0.029	73	19 (19, 37)	0.3 (0.16, 0.52)
mtry factor = 13, s.e. = 0, ntree = 20000				
Leukemia	0.081	2	2 (2, 2)	0.4 (0.22, 0.57)[1]
Breast 2 cl.	0.329	19	9 (3, 39)	0.21 (0.15, 0.29)
Breast 3 cl.	0.351	77	19 (9, 39)	0.12 (0.09, 0.18)
NCI 60	0.348	81	41 (41, 81)	0.28 (0.22, 0.42)
Adenocar.	0.202	5	5 (2, 9)	0.22 (0.2, 0.24)
Brain	0.194	11	43 (21, 43)	0.4 (0.34, 0.63)
Colon	0.173	15	7 (2, 15)	0.26 (0.23, 0.35)
Lymphoma	0.043	125	47 (7, 125)	0.29 (0.13, 0.44)
Prostate	0.069	755	47 (3, 755)	0.19 (0.14, 0.26)
Srbct	0.044	37	37 (37, 73)	0.62 (0.5, 0.84)
mtry factor = 1, s.e. = 1, ntree = 20000				
Leukemia	0.08	2	2 (2, 2)	0.47 (0.32, 0.61)[1]
Breast 2 cl.	0.346	9	3 (2, 9)	0.16 (0.1, 0.26)
Breast 3 cl.	0.362	19	5 (3, 19)	0.16 (0.12, 0.24)
NCI 60	0.346	21	41 (21, 81)	0.38 (0.26, 0.48)
Adenocar.	0.204	5	2 (2, 3)	0.08 (0.07, 0.13)
Brain	0.208	11	21 (11, 43)	0.32 (0.25, 0.56)
Colon	0.177	7	2 (2, 3)	0.28 (0.22, 0.36)
Lymphoma	0.038	63	31 (3, 125)	0.41 (0.33, 0.47)
Prostate	0.06	2	3 (2, 11)	0.94 (0.89, 1)[1]
Srbct	0.03	73	37 (19, 37)	0.32 (0.17, 0.54)
mtry factor = 13, s.e. = 1, ntree = 20000				
Leukemia	0.08	2	2 (2, 2)	0.43 (0.26, 0.61)[1]
Breast 2 cl.	0.352	19	3 (2, 9)	0.13 (0.1, 0.18)
Breast 3 cl.	0.386	19	5 (3, 9)	0.15 (0.08, 0.2)
NCI 60	0.404	81	41 (21, 41)	0.17 (0.11, 0.28)
Adenocar.	0.223	5	2 (2, 5)	0.12 (0.1, 0.16)
Brain	0.199	11	32 (21, 43)	0.46 (0.37, 0.64)
Colon	0.189	3	2 (2, 7)	0.34 (0.31, 0.36)
Lymphoma	0.042	63	31 (7, 125)	0.36 (0.29, 0.48)
Prostate	0.07	2	5 (2, 47)	0.95 (0.91, 1)[1]
Srbct	0.051	19	37 (19, 73)	0.82 (0.63, 0.94)

[1]Since there are only two variables, the values here are the actual frequencies of those two variables, not the 25th and 75th percentiles.

Table 6: `stability-20000` Error rate and stability of results of backwards elimination of variables using OOB error, evaluated using 200 bootstrap samples. Results for $fraction.dropped = 0.5, ntree = 20000, ntreeIterat = 20000$. Error rate is the error rate estimated using 0.632+ bootstrap method. "# Vars" denotes the number of variables selected on the original data set. "# Vars bootstrap" shows the median (1st quartile, 3rd quartile) number of variables selected when the procedure is run on the bootstrap samples. "Freq. vars" is the median (1st quartile, 3rd quartile) of the frequency with which each variable in the original data set appears in the variables selected when the procedure is run on the bootstrap samples.

Data set	Error rate	# Vars	# Vars bootstrap	Freq. vars
mtry factor = 1, s.e. = 0, ntree = 5000, ntreeIterat = 2000				
Leukemia	0.084	2	2 (2, 2)	0.4 (0.3, 0.5)[1]
Breast 2 cl.	0.331	14	9 (5, 23)	0.2 (0.14, 0.29)
Breast 3 cl.	0.345	56	18 (9, 31)	0.14 (0.1, 0.22)
NCI 60	0.319	230	60 (30, 94)	0.12 (0.08, 0.21)
Adenocar.	0.181	6	3 (2, 8)	0.14 (0.13, 0.18)
Brain	0.213	11	14 (8, 22)	0.24 (0.17, 0.44)
Colon	0.167	18	3 (2, 9)	0.19 (0.18, 0.29)
Lymphoma	0.04	73	12 (5, 73)	0.34 (0.24, 0.42)
Prostate	0.061	18	5 (2, 12)	0.21 (0.16, 0.38)
Srbct	0.043	22	18 (11, 27)	0.54 (0.36, 0.88)
mtry factor = 1, s.e. = 1, ntree = 5000, ntreeIterat = 2000				
Leukemia	0.091	2	2 (2, 2)	0.38 (0.26, 0.52)[1]
Breast 2 cl.	0.343	6	4 (3, 7)	0.22 (0.1, 0.26)
Breast 3 cl.	0.367	11	7 (4, 14)	0.2 (0.1, 0.3)
NCI 60	0.355	19	34 (19, 60)	0.32 (0.29, 0.44)
Adenocar.	0.205	8	2 (2, 4)	0.08 (0.06, 0.09)
Brain	0.199	9	14 (7, 22)	0.28 (0.22, 0.46)
Colon	0.181	5	3 (2, 5)	0.3 (0.24, 0.38)
Lymphoma	0.038	91	15 (4, 91)	0.3 (0.21, 0.4)
Prostate	0.06	2	3 (2, 5)	0.93 (0.86, 1)[1]
Srbct	0.045	52	18 (11, 27)	0.27 (0.18, 0.45)
mtry factor = 1, s.e. = 0, ntree = 2000, ntreeIterat = 1000				
Leukemia	0.087	2	2 (2, 2)	0.38 (0.29, 0.48)[1]
Breast 2 cl.	0.337	14	9 (5, 23)	0.15 (0.1, 0.28)
Breast 3 cl.	0.346	110	14 (9, 31)	0.08 (0.04, 0.13)
NCI 60	0.327	230	60 (30, 94)	0.1 (0.06, 0.19)
Adenocar.	0.185	6	3 (2, 8)	0.14 (0.12, 0.15)
Brain	0.216	22	14 (7, 22)	0.18 (0.09, 0.25)
Colon	0.159	14	5 (3, 12)	0.29 (0.19, 0.42)
Lymphoma	0.047	73	14 (4, 58)	0.26 (0.18, 0.38)
Prostate	0.061	18	5 (3, 14)	0.22 (0.17, 0.43)
Srbct	0.039	101	18 (11, 27)	0.1 (0.04, 0.29)
mtry factor = 1, s.e. = 1, ntree = 2000, ntreeIterat = 1000				
Leukemia	0.075	2	2 (2, 2)	0.4 (0.32, 0.5)[1]
Breast 2 cl.	0.332	14	4 (2, 7)	0.12 (0.07, 0.17)
Breast 3 cl.	0.364	6	7 (4, 14)	0.27 (0.22, 0.31)
NCI 60	0.353	24	30 (19, 60)	0.26 (0.17, 0.38)
Adenocar.	0.207	8	3 (2, 5)	0.06 (0.03, 0.12)
Brain	0.216	9	14 (7, 22)	0.26 (0.14, 0.46)
Colon	0.177	3	3 (2, 6)	0.36 (0.32, 0.36)
Lymphoma	0.042	58	12 (5, 73)	0.32 (0.24, 0.42)
Prostate	0.064	2	3 (2, 5)	0.9 (0.82, 0.99)[1]
Srbct	0.038	22	18 (11, 34)	0.57 (0.4, 0.88)

[1] Since there are only two variables, the values here are the actual frequencies of those two variables, not the 25th and 75th percentiles.

Table 7: `stability-02` Error rate and stability of results of backwards elimination of variables using OOB error, evaluated using 200 bootstrap samples. Results for $fraction.dropped = 0.2$. Error rate is the error rate estimated using 0.632+ bootstrap method. "# Vars" denotes the number of variables selected on the original data set. "# Vars bootstrap" shows the median (1st quartile, 3rd quartile) number of variables selected when the procedure is run on the bootstrap samples. "Freq. vars" is the median (1st quartile, 3rd quartile) of the frequency with which each variable in the original data set appears in the variables selected when the procedure is run on the bootstrap samples.

Data set	Error rate	# Vars	# Vars bootstrap	Freq. vars
Shrunken centroids; mimimizing error rate then maximizing log-likelihood				
Leukemia	0.025	3051	3051 (102, 3051)	0.64 (0.6, 0.68)
Breast 2 cl.	0.324	31	71 (33, 340)	0.58 (0.54, 0.65)
Breast 3 cl.	0.396	2166	4562 (3272, 4869)	0.9 (0.86, 0.92)
NCI 60	0.256	4703	4590 (3485, 5232)	0.82 (0.72, 0.92)
Adenocar.	0.177	1	11 (4, 20)	0.66 (0.66, 0.66)[1]
Brain	0.163	5270	2070 (459, 4026)	0.42 (0.32, 0.55)
Colon	0.123	23	26 (20, 70)	0.77 (0.57, 0.89)
Lymphoma	0.028	2796	3336 (2664, 4026)	0.88 (0.82, 0.92)
Prostate	0.088	11	8 (4, 14)	0.57 (0.37, 0.78)
Srbct	0.012	209	206 (130, 470)	0.68 (0.56, 0.86)
Shrunken centroids; mimimizing error rate then minimizing number of genes selected				
Leukemia	0.062	82	46 (14, 504)	0.48 (0.45, 0.59)
Breast 2 cl.	0.326	31	55 (24, 296)	0.54 (0.51, 0.66)
Breast 3 cl.	0.401	2166	4341 (2379, 4804)	0.84 (0.78, 0.88)
NCI 60	0.246	5118	4919 (3711, 5243)	0.84 (0.74, 0.92)
Adenocar.	0.179	0	9 (0, 18)	NA (NA, NA)[2]
Brain	0.159	4177	1257 (295, 3483)	0.38 (0.3, 0.5)
Colon	0.122	15	22 (15, 34)	0.8 (0.66, 0.87)
Lymphoma	0.033	2796	2718 (2030, 3269)	0.82 (0.68, 0.86)
Prostate	0.089	4	3 (2, 4)	0.72 (0.49, 0.92)
Srbct	0.025	37	18 (12, 40)	0.45 (0.34, 0.61)
Nearest Neighbor with variable selection				
Leukemia	0.056	512	23 (4, 134)	0.17 (0.14, 0.24)
Breast 2 cl.	0.337	88	23 (4, 110)	0.24 (0.2, 0.31)
Breast 3 cl.	0.424	9	45 (6, 214)	0.66 (0.61, 0.72)
NCI 60	0.237	1718	880 (360, 1718)	0.44 (0.34, 0.57)
Adenocar.	0.181	9868	73 (8, 1324)	0.13 (0.1, 0.18)
Brain	0.194	1834	158 (52, 601)	0.16 (0.12, 0.25)
Colon	0.158	8	9 (4, 45)	0.57 (0.45, 0.72)
Lymphoma	0.04	15	15 (5, 39)	0.5 (0.4, 0.6)
Prostate	0.081	7	6 (3, 18)	0.46 (0.39, 0.78)
Srbct	0.031	11	17 (11, 33)	0.7 (0.66, 0.85)

[1]Only one variable was selected.
[2]No variables were selected.

Table 8: Stability (and error rates) of results from two alternative approaches for variable selection, evaluated using 200 bootstrap samples. "# Vars" denotes the number of variables selected on the original data set. "# Vars bootstrap" shows the median (1st quartile, 3rd quartile) number of variables selected when the procedure is run on the bootstrap samples. "Freq. vars" is the median (1st quartile, 3rd quartile) of the frequency with which each variable in the original data set appears in the variables selected when the procedure is run on the bootstrap samples. For details on the methods, see text.

6 Variable importance: relation with Kruskal-Wallis and ANOVA rankings

In figure 1 we show the relationship between variable importances from random forest and the p-values obtained from testing, for each gene, the null hypothesis of no differential expression, using both Kruskal-Wallis non-parametric test and an ANOVA (an ANOVA ranking is the same as a t-test ranking in the two-group case). As can be seen from the figure, the relationship between the variable importance from random forest and either of Kruskal-Walli's or ANOVA's is often much weaker than the relationship between the rankings of ANOVA and Kruskal-Wallis. Thus, there is no evidence that the variable importances from random forest are very similar to the rankings of importance we would obtain from doing Kruskal-Wallis tests on each gene.

The above can be observed more clearly if we carry out rank correlation tests (i.e., Spearman's correlation coefficient) between the rankings from each of random forest, Kruskal-Wallis and ANOVA.

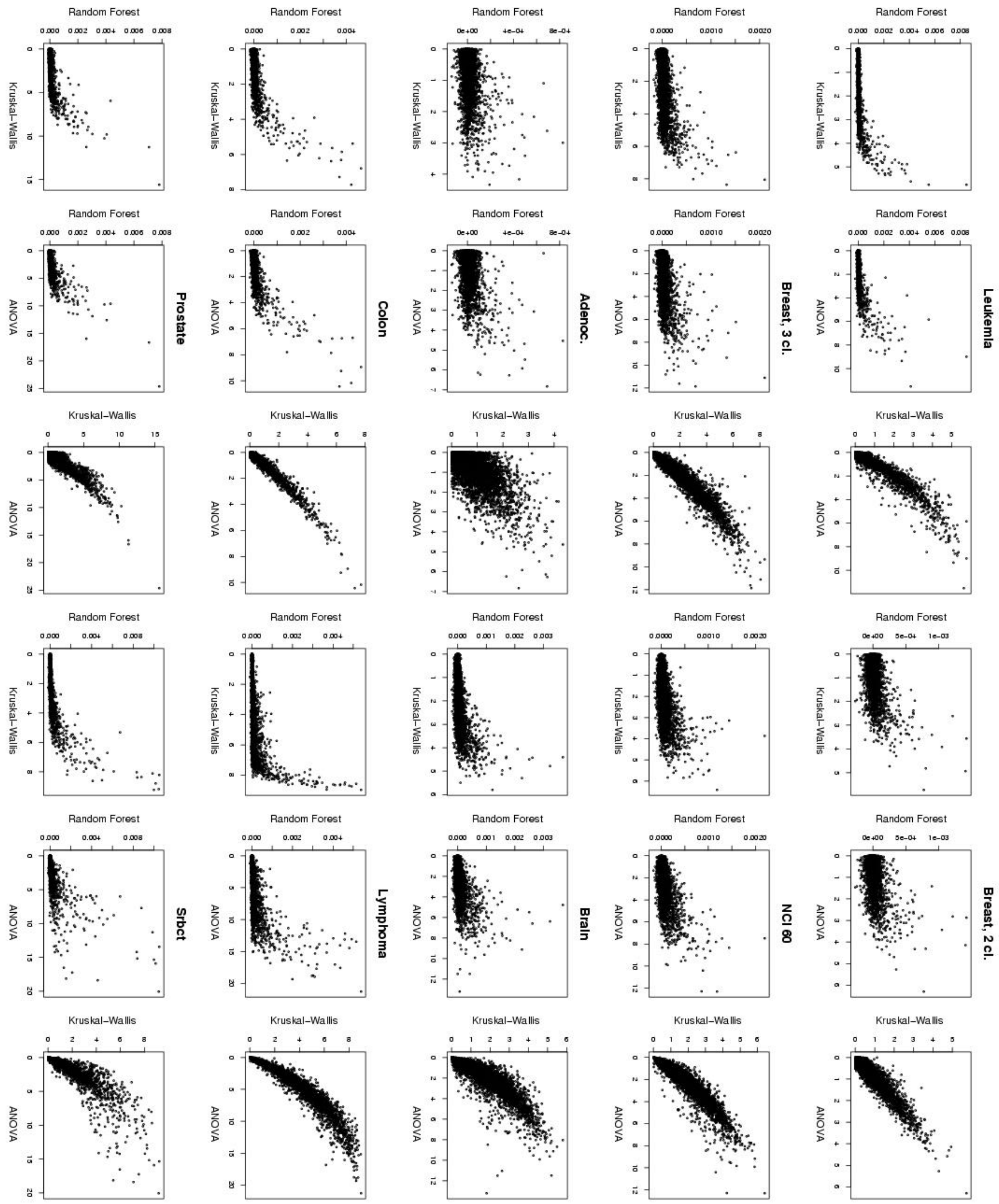

Figure 1: Relationship between random forest's variable importance and p-values from ANOVA and Kruskal-Wallis tests. The p-values from ANOVA and Kruskal-Wallis tests correspond to testing, for each gene, the null hypothesis of no differential expression among classes. For ease of interpretation, we use $-\log_{10} p - value$ when showing the p-values from Kruskal-Wallis and ANOVA. Each three consecutive (along the horizontal dimension) scatterplots corresponds to a data set.

Data set	All genes			100 most important genes from random forest		
	rF - KW	rF-ANOVA	KW-ANOVA	rF - KW	rF-ANOVA	KW-ANOVA
Leukemia	0.401	0.398	0.937	0.48	0.119	0.342
Breast 2 cl.	0.193	0.198	0.903	0.214	0.173	0.683
Breast 3 cl.	0.301	0.294	0.968	0.216	0.034	0.797
NCI 60	0.442	0.439	0.954	-0.295	0.237	0.225
Adenocar.	0.114	0.125	0.539	0.212	-0.084	0.609
Brain	0.408	0.379	0.91	0.242	0.205	0.796
Colon	0.39	0.393	0.955	0.574	0.597	0.78
Lymphoma	0.526	0.528	0.983	0.487	0.176	-0.006
Prostate	0.245	0.252	0.751	0.202	0.248	0.297
Srbct	0.614	0.572	0.922	0.462	0.357	0.373

Table 9: Rank correlation between importances from random forest, $-log_{10}$ p-values from Kruskal-Wallis and $-log_{10}$ p-values from ANOVA. rF: random Forest; KW: Kruskal-Wallis.

These correlation coefficients are shown in table 9. It can be argued that the correlation ought to be considered only for the most important genes according to random forest, since the figures above show that random forests importances are very flat for the least important genes. Thus, we have recomputed the correlation using only the 100 most important genes according to random forests. This table clearly shows that the rankings from random forest and Kruskal-Wallis are less similar than the rankings from Kruskal-Wallis and ANOVA.

References

Breiman, L. (2001) Random forests. *Machine Learning,* **45**, 5–32.

Bureau, A., Dupuis, J., Hayward, B., Falls, K. & Van Eerdewegh, P. (2003) Mapping complex traits using Random Forests. *BMC Genet,* **4 Suppl 1**, S64.

Dettling, M. & Bühlmann, P. (2002) Supervised clustering of genes. *Genome Biology,* **3** (12), 0069.1–0069.15.

Dettling, M. & Bühlmann, P. (2003) Boosting for tumor classification with gene expression data. *Bioinformatics,* **19** (9), 1061–1069.

Dudoit, S., Fridlyand, J. & Speed, T. P. (2002) Comparison of discrimination methods for the classification of tumors suing gene expression data. *J Am Stat Assoc,* **97** (457), 77–87.

Golub, T. R., Slonim, D. K., Tamayo, P., Huard, C., Gaasenbeek, M., Mesirov, J. P., Coller, H., Loh, M. L., Downing, J. R., Caligiuri, M. A., Bloomfield, C. D. & Lander, E. S. (1999) Molecular classification of cancer: class discovery and class prediction by gene expression monitoring. *Science,* **286**, 531–537.

Herrero, J., Díaz-Uriarte, R. & Dopazo, J. (2003) Gene expression data preprocessing. *Bioinformatics,* **19** (5), 655–656.

McLachlan, G. J. (1992) *Discriminant analysis and statistical pattern recognition.* Wiley, New York.

Ramaswamy, S., Ross, K. N., Lander, E. S. & Golub, T. R. (2003) A molecular signature of metastasis in primary solid tumors. *Nature Genetics,* **33**, 49–54.

Remlinger, K. Introduction and application of random forest on high throughput screeing from drug discovery.

Ross, D. T., Scherf, U., Eisen, M. B., Perou, C. M., Rees, C., Spellman, P., Iyer, V., Jeffrey, S. S., de Rijn, M. V., Waltham, M., Pergamenschikov, A., Lee, J. C., Lashkari, D., Shalon, D., Myers, T. G., Weinstein, J. N., Botstein, D. & Brown, P. O. (2000) Systematic variation in gene expression patterns in human cancer cell lines. *Nature Genetics,* **24** (3), 227–235.

Troyanskaya, O., Cantor, M., Sherlock, G., Brown, P., Hastie, T., Tibshirani, R., Botstein, D. & Altman, R. (2001) Missing value estimation methods for dna microarrays. *Bioinformatics,* **17**, 520–525.

van 't Veer, L. J., Dai, H., van de Vijver, M. J., He, Y. D., Hart, A. A. M., Mao, M., Peterse, H. L., van der Kooy, K., Marton, M. J., Witteveen, A. T., Schreiber, G. J., Kerkhoven, R. M., Roberts, C., Linsley, P. S., Bernards, R. & Friend, S. H. (2002) Gene expression profiling predicts clinical outcome of breast cancer. *Nature,* **415**, 530–536.
