# Supplementary material for: Gene selection and classification of microarray data using random forest
Source: BMC Bioinformatics. 2006 Jan 6;7:3. doi: 10.1186/1471-2105-7-3 (PMC1363357; doi:10.1186/1471-2105-7-3)
Supplement: Additional File 2 — A PDF file with additional plots of OOB error rate vs. mtry for both simulated data and real data under other parameters. [file 1471-2105-7-3-S2.pdf]

OOB error rate vs. mtry

x−axis: factor that multiplies default mtry [sqrt(number variables)]

Mtry factors used: {0, 0.05, 0.1, 0.17, 0.25, 0.33, 0.5, 0.75, 0.8, 1, 1.15, 1.33, 1.5, 2, 3, 4, 5, 6, 8, 10, 13}

(mtry factors of 0 means mtry = 1)

I: Simulated data sets:

signal + 4000 noise random variables

(shown first with nodesize = 1, then nodesize = 5)

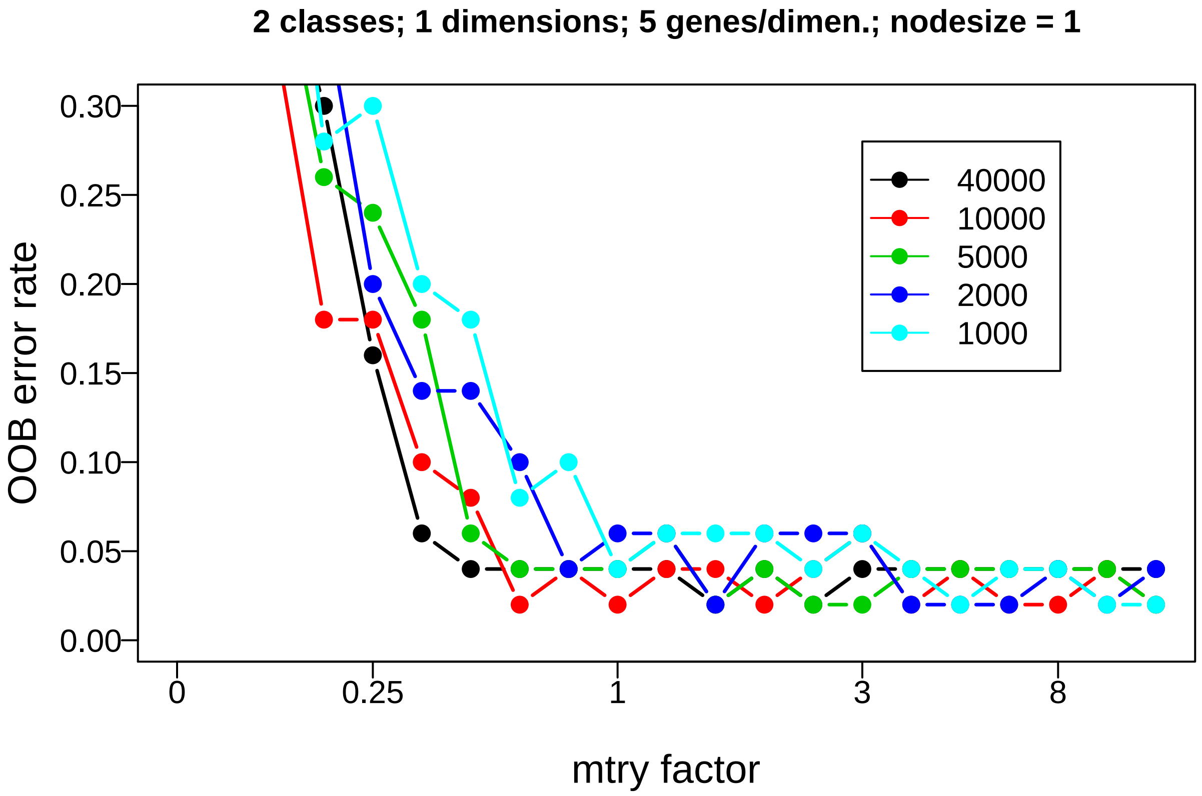
2 classes; 1 dimensions; 5 genes/dimen.; nodesize = 1
OOB error rate
mtry factor
0.30
0.25
0.20
0.15
0.10
0.05
0.00
0
0.25
1
3
8
40000
10000
5000
2000
1000

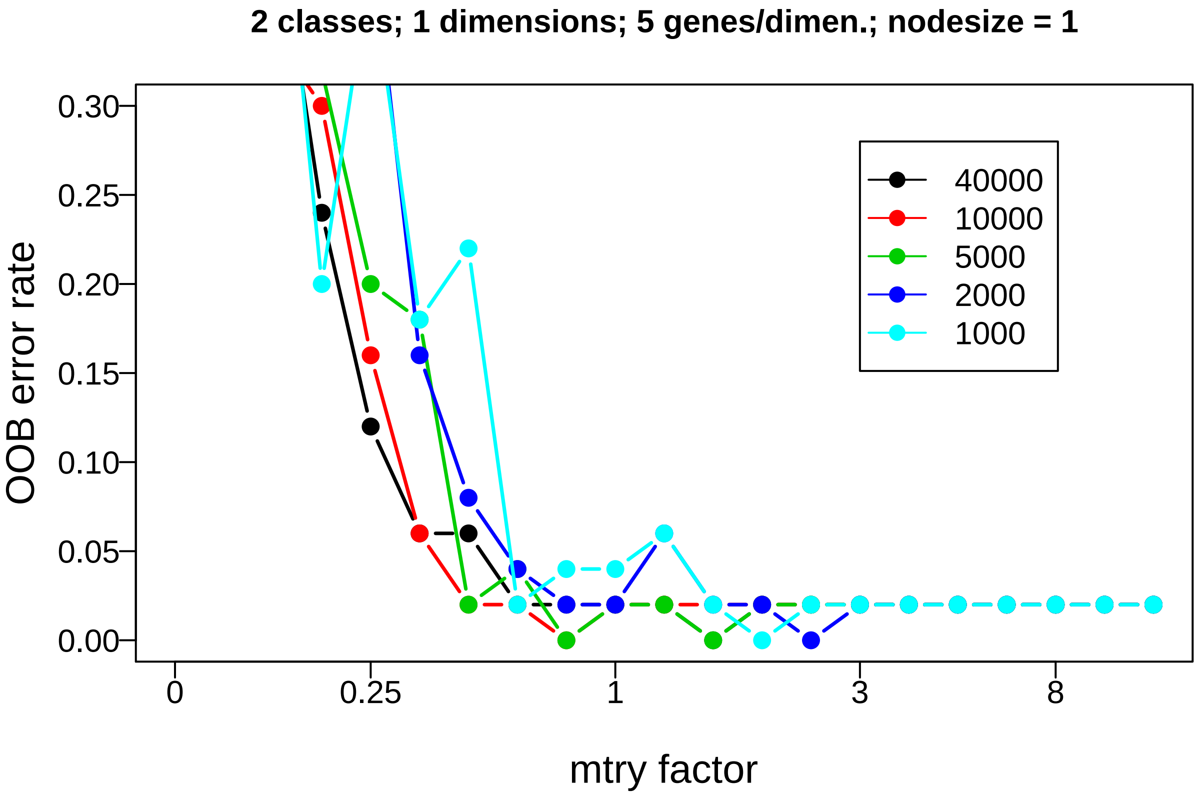
2 classes; 1 dimensions; 5 genes/dimen.; nodesize = 1
OOB error rate
mtry factor
0.30
0.25
0.20
0.15
0.10
0.05
0.00
0
0.25
1
3
8
40000
10000
5000
2000
1000

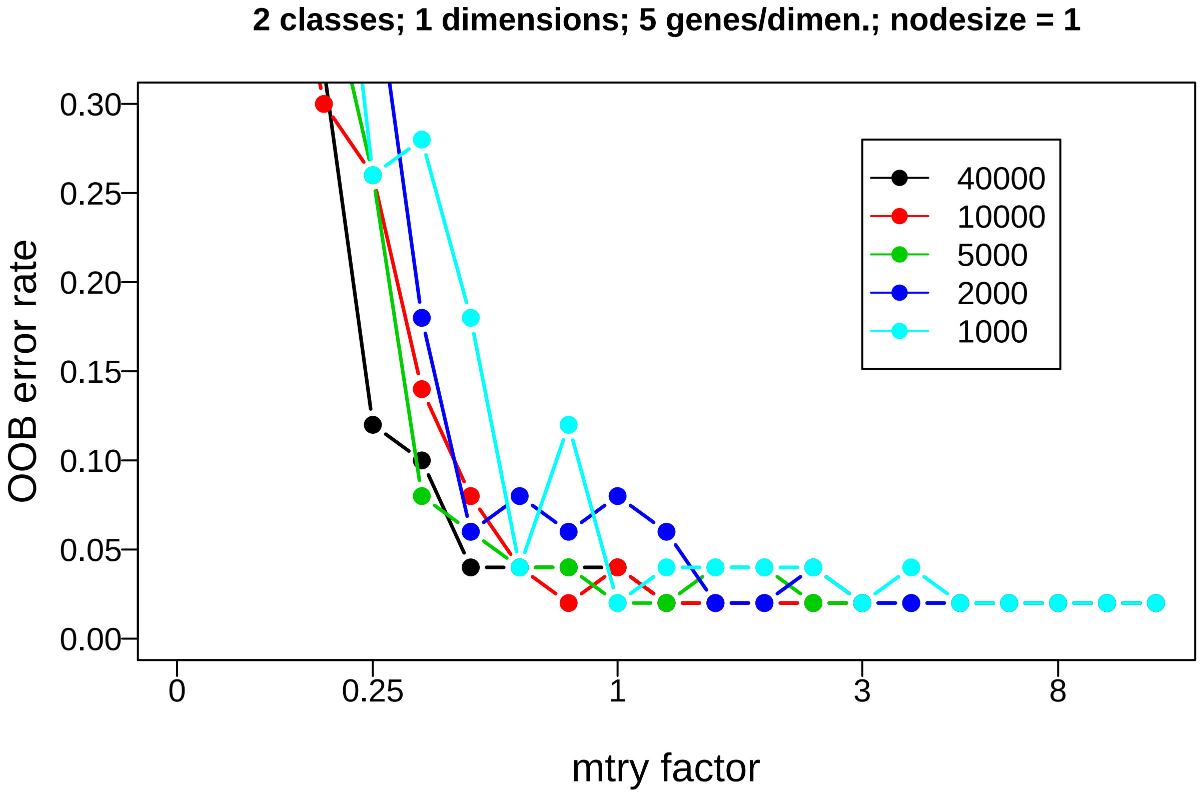
2 classes; 1 dimensions; 5 genes/dimen.; nodesize = 1
OOB error rate
mtry factor
0.30
0.25
0.20
0.15
0.10
0.05
0.00
0
0.25
1
3
8
40000
10000
5000
2000
1000

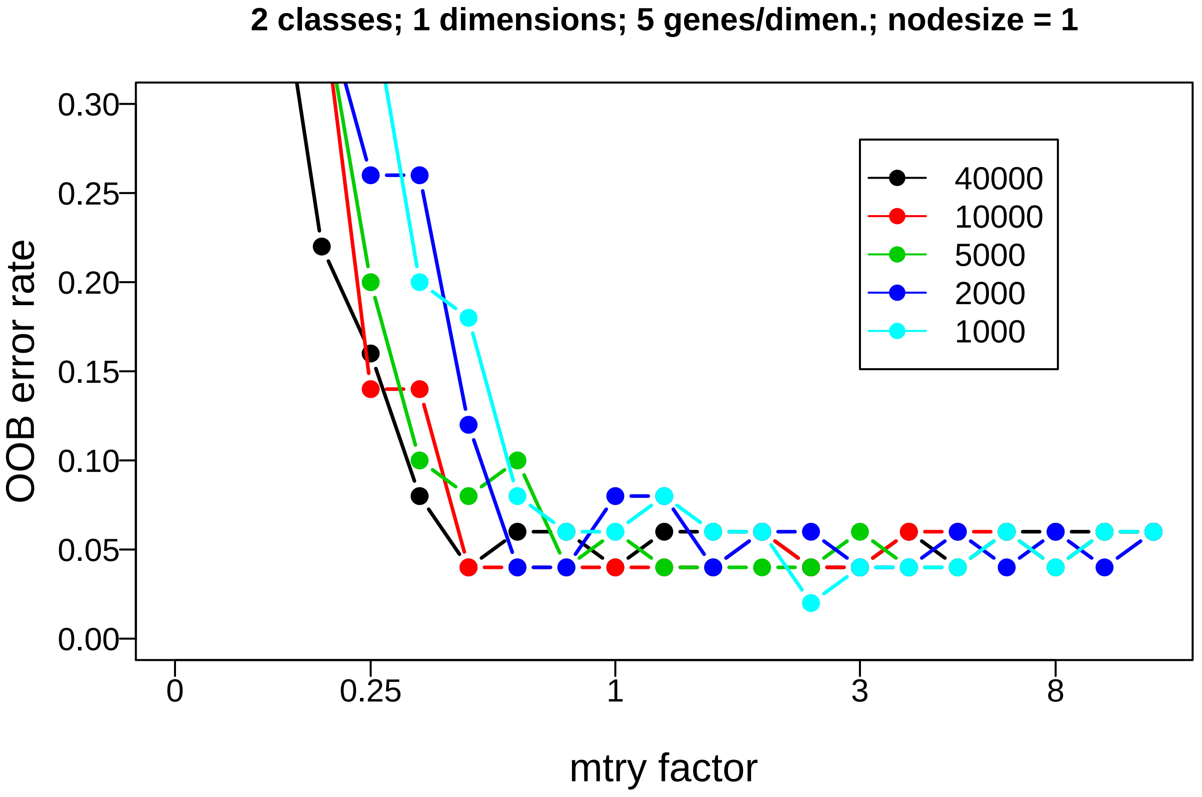
2 classes; 1 dimensions; 5 genes/dimen.; nodesize = 1
OOB error rate
mtry factor
0.30
0.25
0.20
0.15
0.10
0.05
0.00
0
0.25
1
3
8
40000
10000
5000
2000
1000

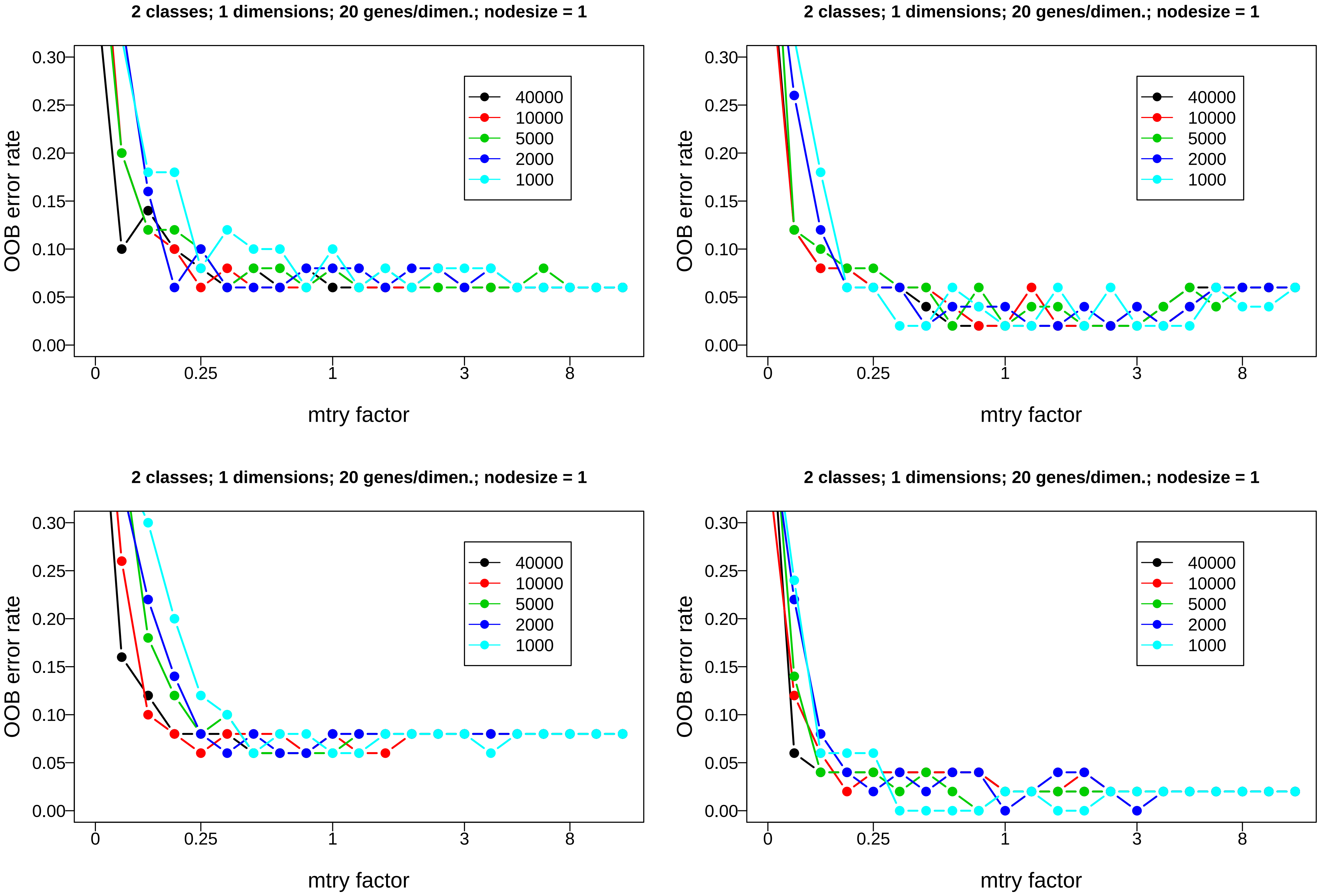

2 classes; 1 dimensions; 20 genes/dimen.; nodesize = 1
OOB error rate
mtry factor
0.30
0.25
0.20
0.15
0.10
0.05
0.00
0
0.25
1
3
8
40000
10000
5000
2000
1000
2 classes; 1 dimensions; 20 genes/dimen.; nodesize = 1
OOB error rate
mtry factor
40000
10000
5000
2000
1000
2 classes; 1 dimensions; 20 genes/dimen.; nodesize = 1
OOB error rate
mtry factor
40000
10000
5000
2000
1000
2 classes; 1 dimensions; 20 genes/dimen.; nodesize = 1
OOB error rate
mtry factor
40000
10000
5000
2000
1000

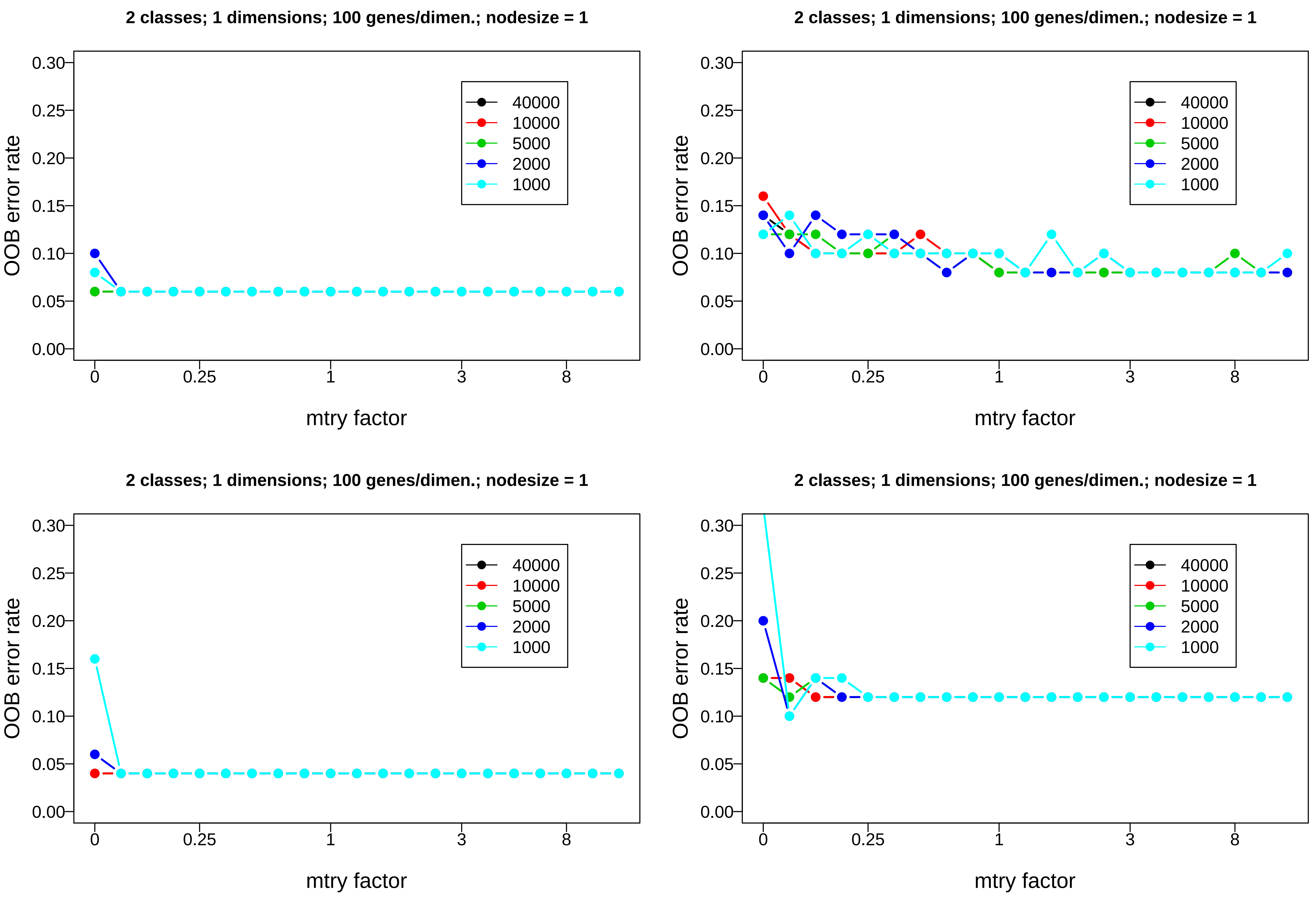
2 classes; 1 dimensions; 100 genes/dimen.; nodesize = 1
OOB error rate
mtry factor
0.00
0.05
0.10
0.15
0.20
0.25
0.30
0
0.25
1
3
8
40000
10000
5000
2000
1000
2 classes; 1 dimensions; 100 genes/dimen.; nodesize = 1
OOB error rate
mtry factor
2 classes; 1 dimensions; 100 genes/dimen.; nodesize = 1
OOB error rate
mtry factor
2 classes; 1 dimensions; 100 genes/dimen.; nodesize = 1
OOB error rate
mtry factor

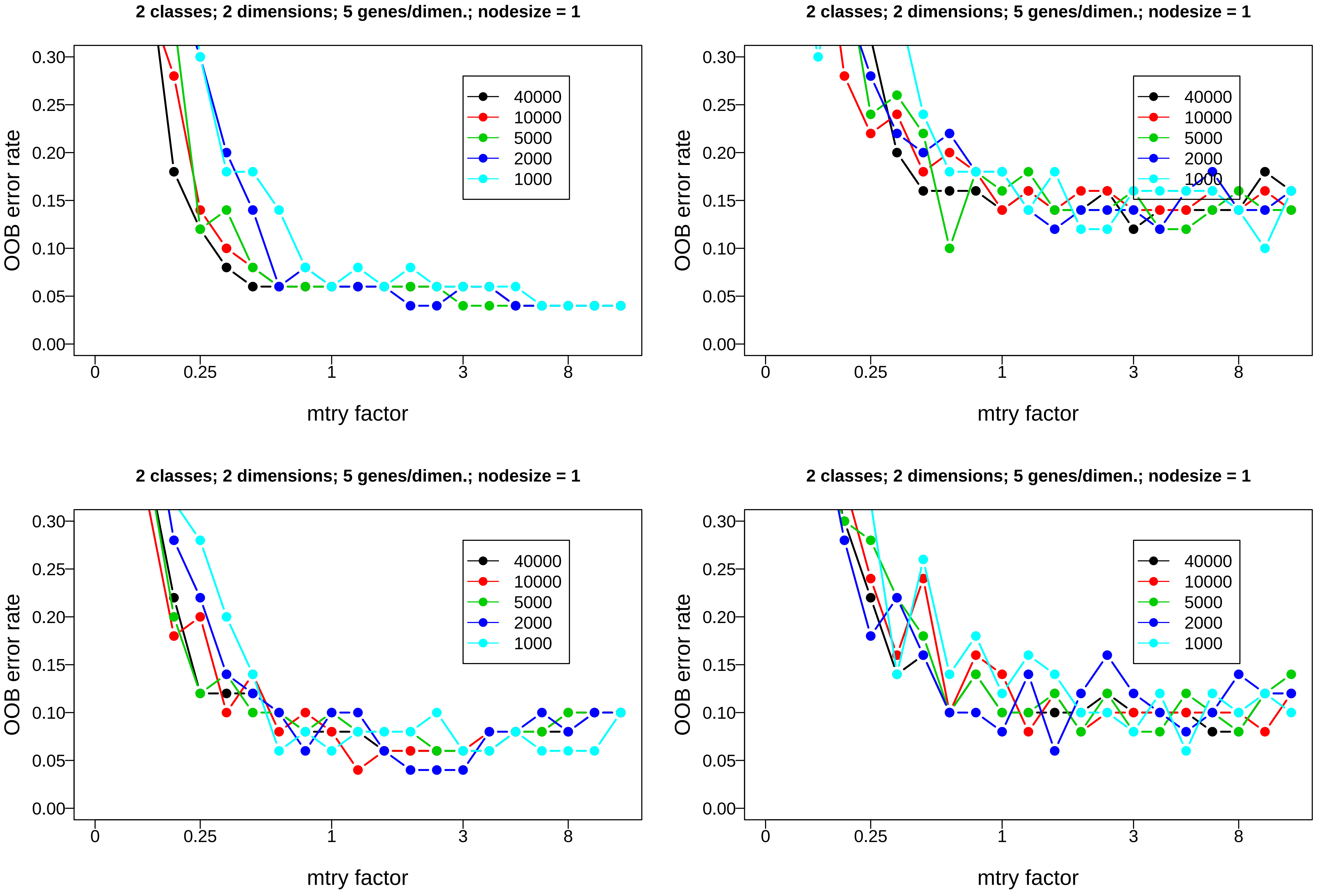
2 classes; 2 dimensions; 5 genes/dimen.; nodesize = 1
OOB error rate
0.00
0.05
0.10
0.15
0.20
0.25
0.30
0
0.25
1
3
8
mtry factor
40000
10000
5000
2000
1000
2 classes; 2 dimensions; 5 genes/dimen.; nodesize = 1
OOB error rate
mtry factor
40000
10000
5000
2000
1000
2 classes; 2 dimensions; 5 genes/dimen.; nodesize = 1
OOB error rate
mtry factor
40000
10000
5000
2000
1000
2 classes; 2 dimensions; 5 genes/dimen.; nodesize = 1
OOB error rate
mtry factor
40000
10000
5000
2000
1000

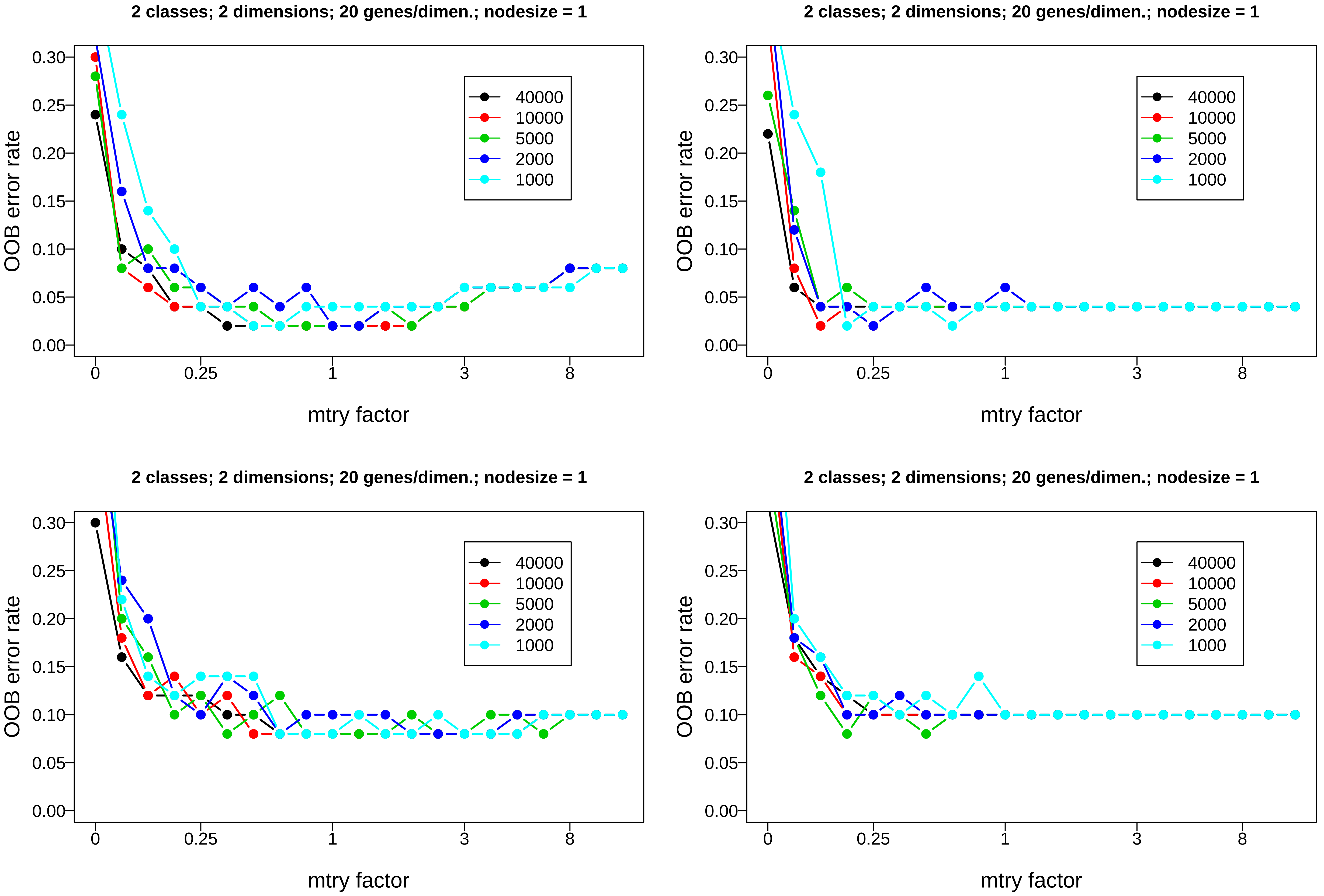

2 classes; 2 dimensions; 20 genes/dimen.; nodesize = 1
OOB error rate
mtry factor
0.00
0.05
0.10
0.15
0.20
0.25
0.30
0
0.25
1
3
8
40000
10000
5000
2000
1000

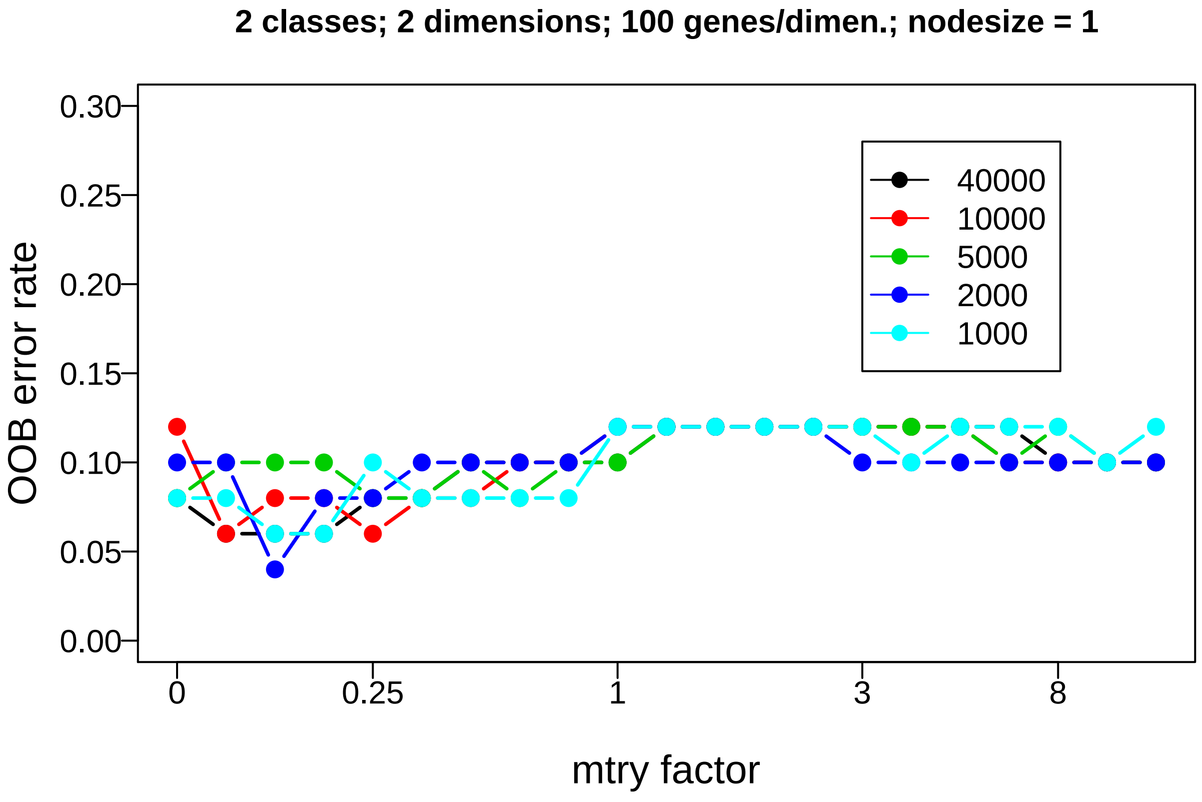
2 classes; 2 dimensions; 100 genes/dimen.; nodesize = 1
OOB error rate
0.30
0.25
0.20
0.15
0.10
0.05
0.00
0
0.25
1
3
8
mtry factor
40000
10000
5000
2000
1000

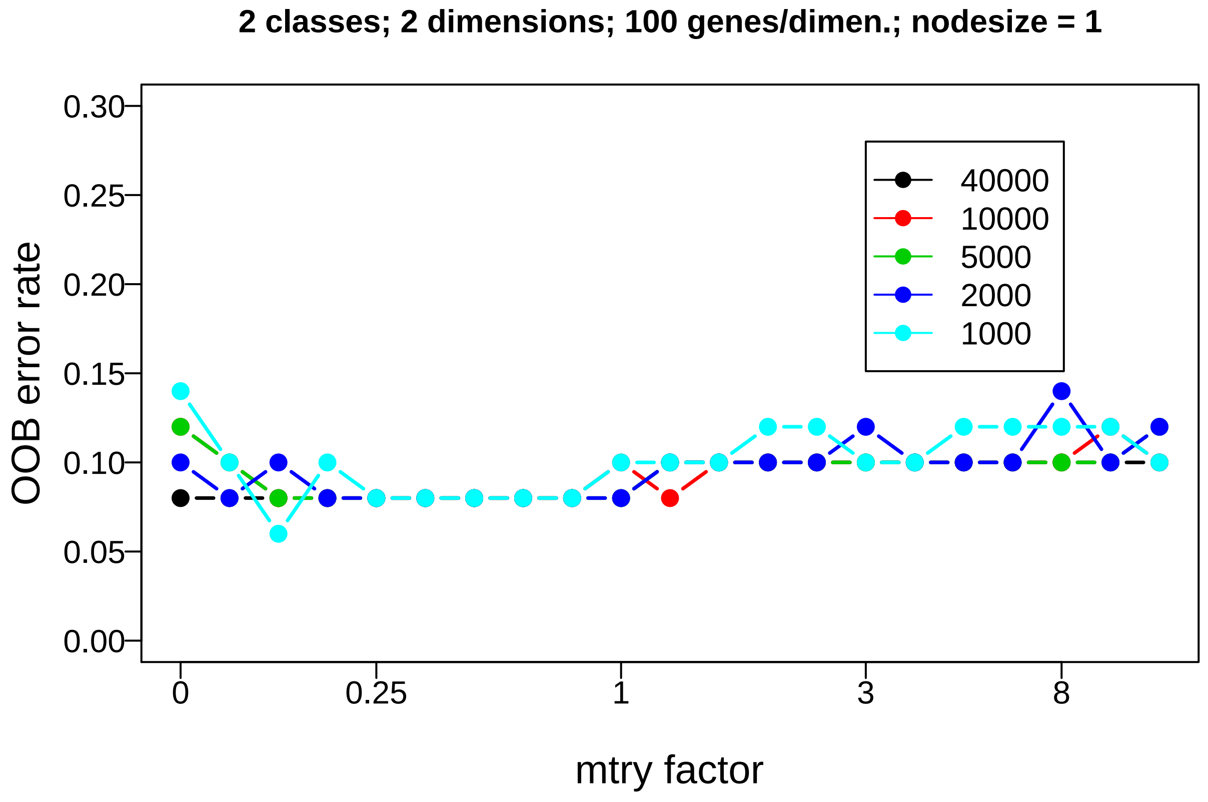
2 classes; 2 dimensions; 100 genes/dimen.; nodesize = 1
OOB error rate
0.30
0.25
0.20
0.15
0.10
0.05
0.00
0
0.25
1
3
8
mtry factor
40000
10000
5000
2000
1000

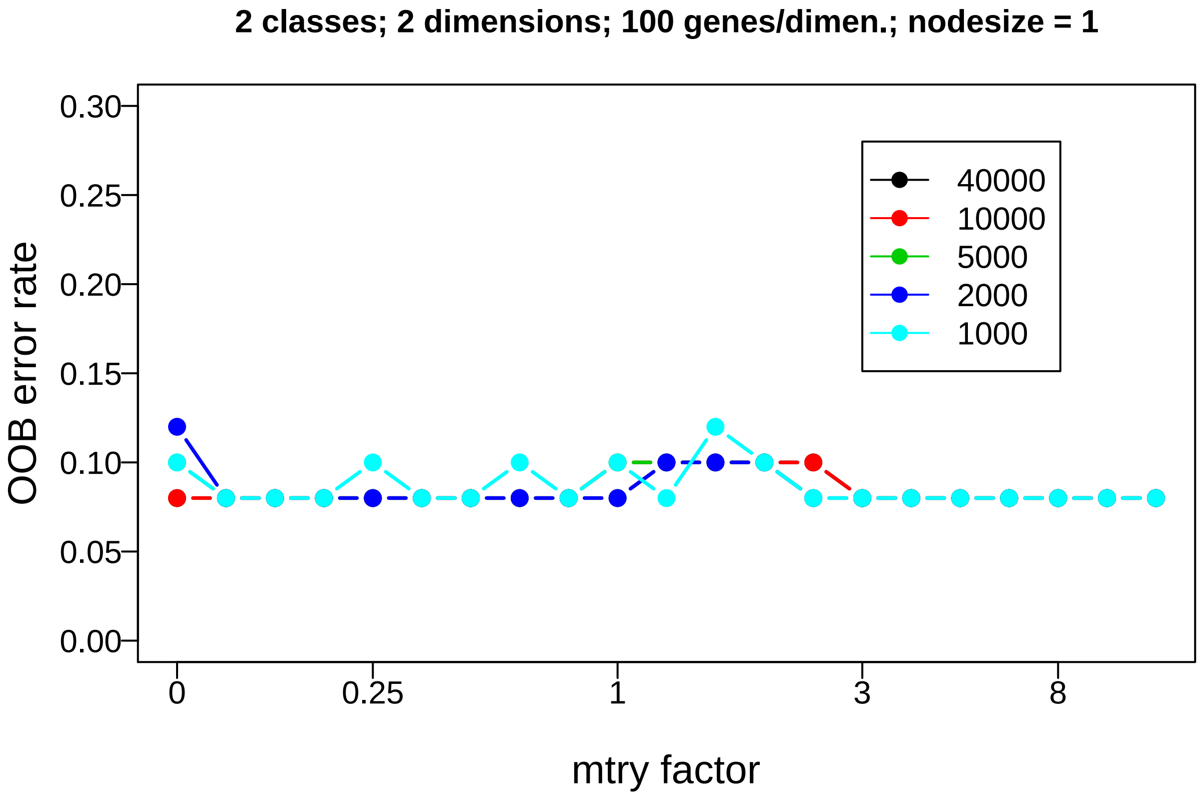
2 classes; 2 dimensions; 100 genes/dimen.; nodesize = 1
OOB error rate
0.30
0.25
0.20
0.15
0.10
0.05
0.00
0
0.25
1
3
8
mtry factor
40000
10000
5000
2000
1000

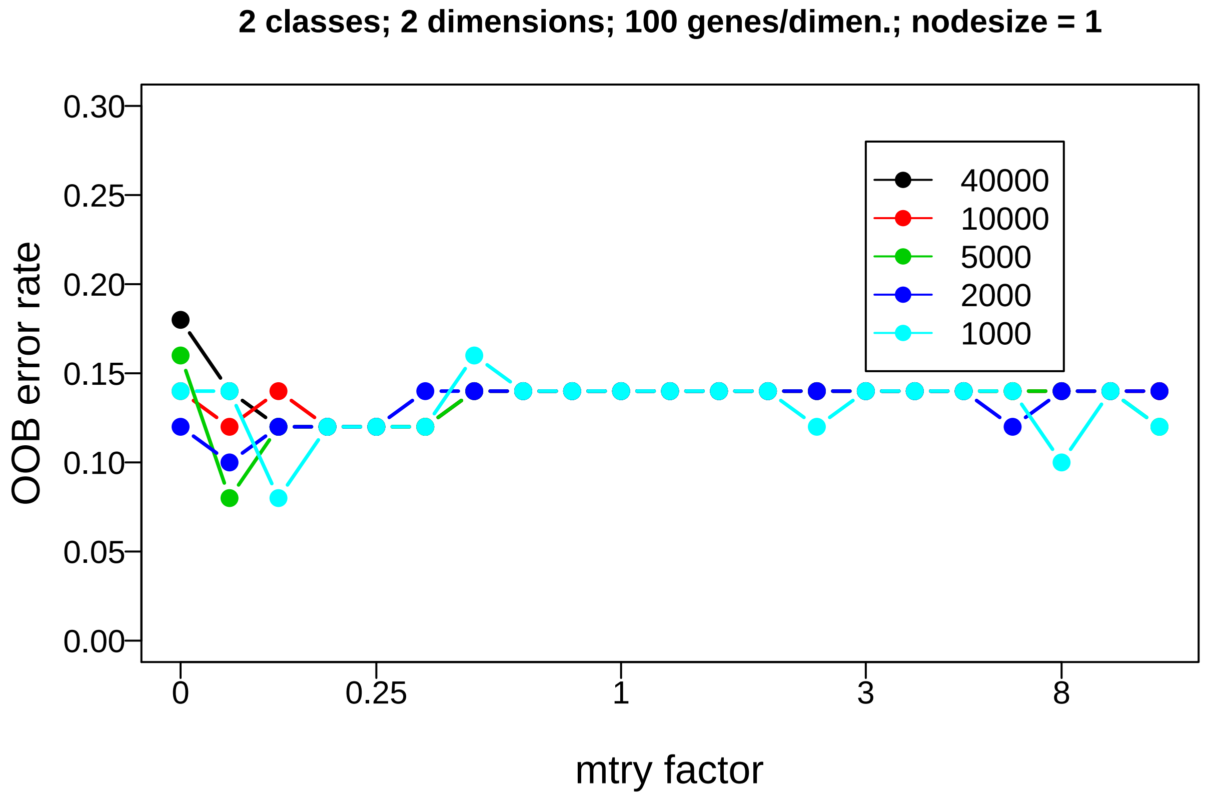
2 classes; 2 dimensions; 100 genes/dimen.; nodesize = 1
OOB error rate
0.30
0.25
0.20
0.15
0.10
0.05
0.00
0
0.25
1
3
8
mtry factor
40000
10000
5000
2000
1000

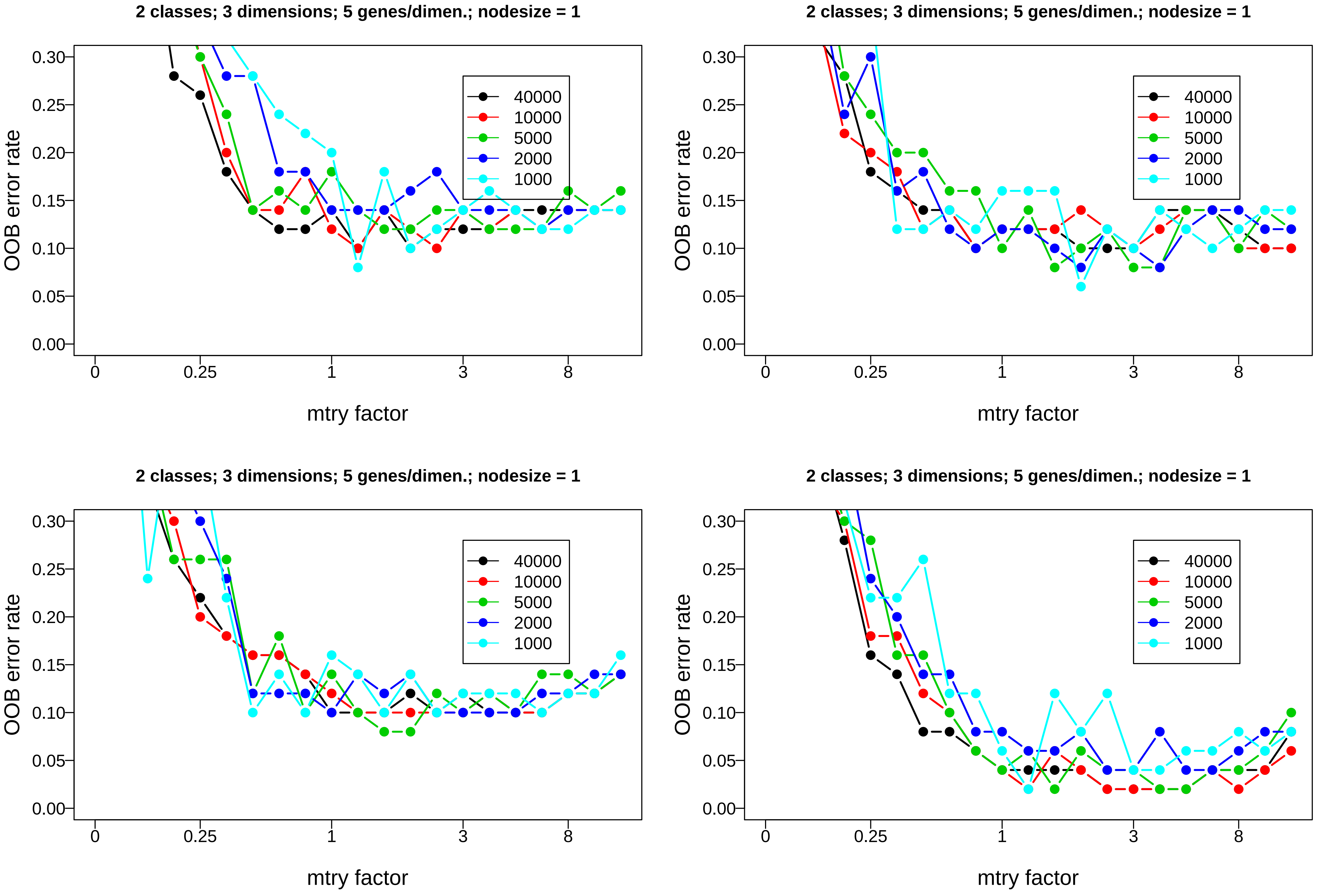
2 classes; 3 dimensions; 5 genes/dimen.; nodesize = 1
OOB error rate
mtry factor
0.30
0.25
0.20
0.15
0.10
0.05
0.00
0
0.25
1
3
8
40000
10000
5000
2000
1000
2 classes; 3 dimensions; 5 genes/dimen.; nodesize = 1
OOB error rate
mtry factor
40000
10000
5000
2000
1000
2 classes; 3 dimensions; 5 genes/dimen.; nodesize = 1
OOB error rate
mtry factor
40000
10000
5000
2000
1000
2 classes; 3 dimensions; 5 genes/dimen.; nodesize = 1
OOB error rate
mtry factor
40000
10000
5000
2000
1000

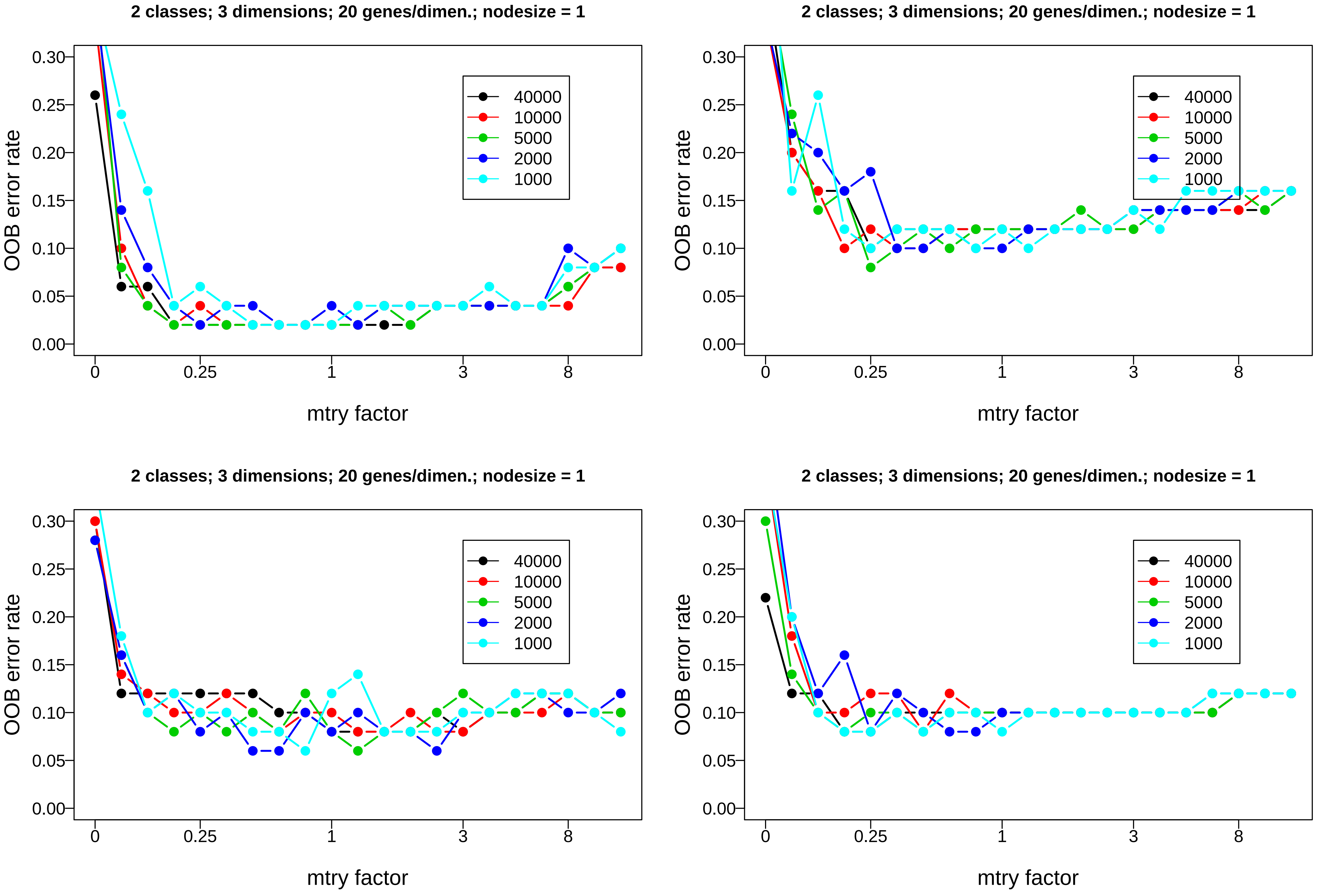
2 classes; 3 dimensions; 20 genes/dimen.; nodesize = 1
OOB error rate
mtry factor
0.00
0.05
0.10
0.15
0.20
0.25
0.30
0
0.25
1
3
8
40000
10000
5000
2000
1000
2 classes; 3 dimensions; 20 genes/dimen.; nodesize = 1
OOB error rate
mtry factor
2 classes; 3 dimensions; 20 genes/dimen.; nodesize = 1
OOB error rate
mtry factor
2 classes; 3 dimensions; 20 genes/dimen.; nodesize = 1
OOB error rate
mtry factor

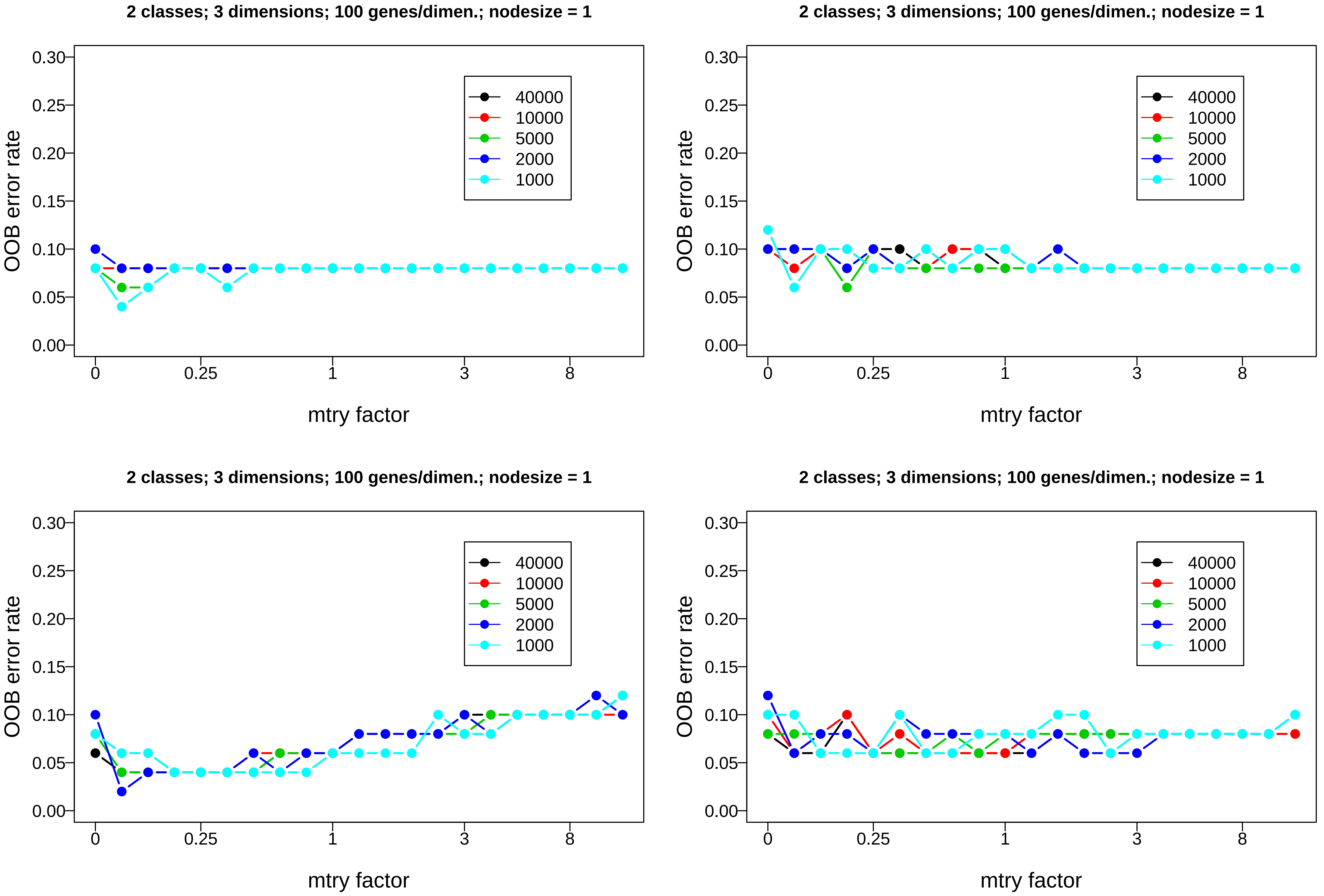
2 classes; 3 dimensions; 100 genes/dimen.; nodesize = 1
OOB error rate
mtry factor
0.00
0.05
0.10
0.15
0.20
0.25
0.30
0
0.25
1
3
8
40000
10000
5000
2000
1000

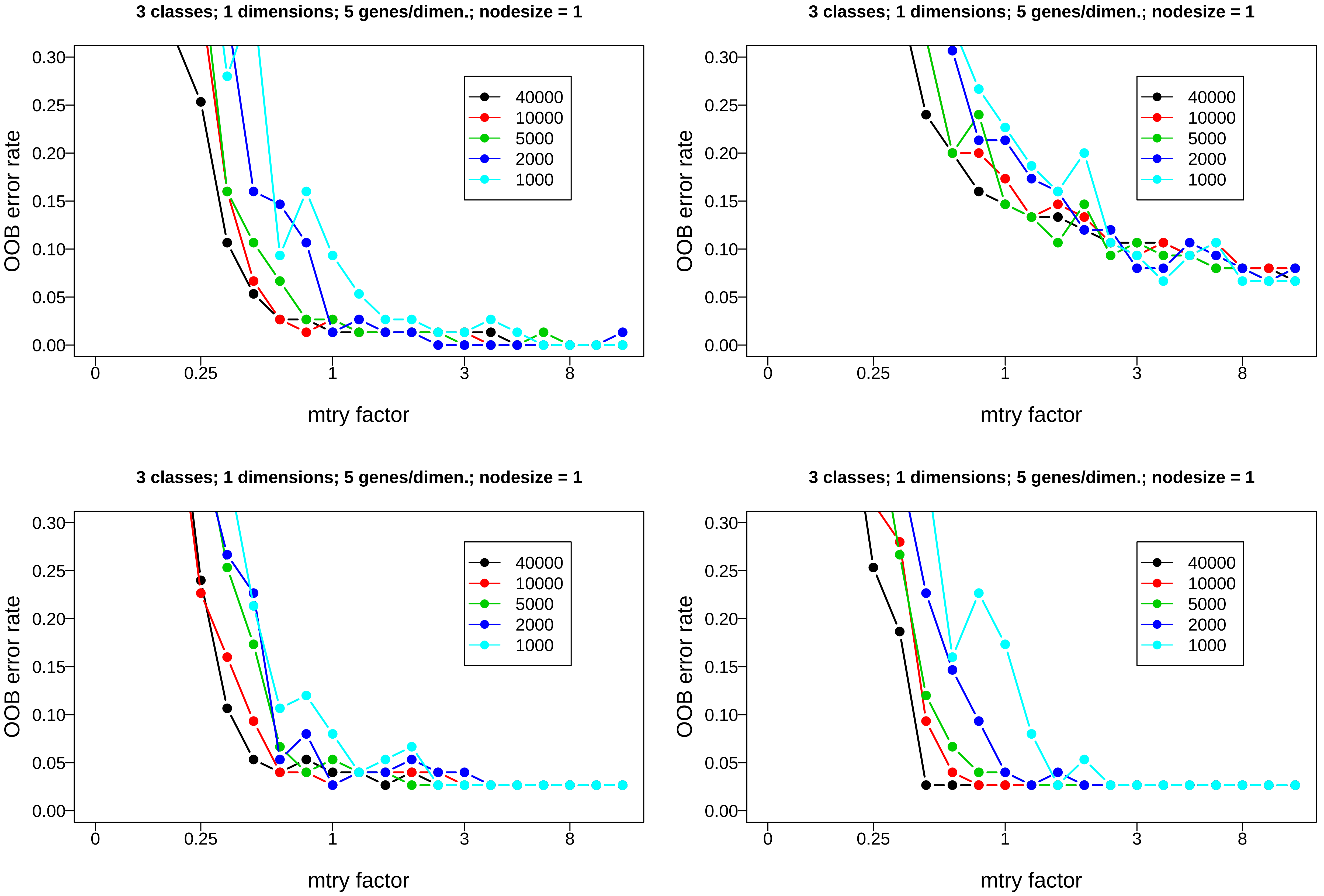
3 classes; 1 dimensions; 5 genes/dimen.; nodesize = 1
OOB error rate
mtry factor
40000
10000
5000
2000
1000
0.00
0.05
0.10
0.15
0.20
0.25
0.30
0
0.25
1
3
8

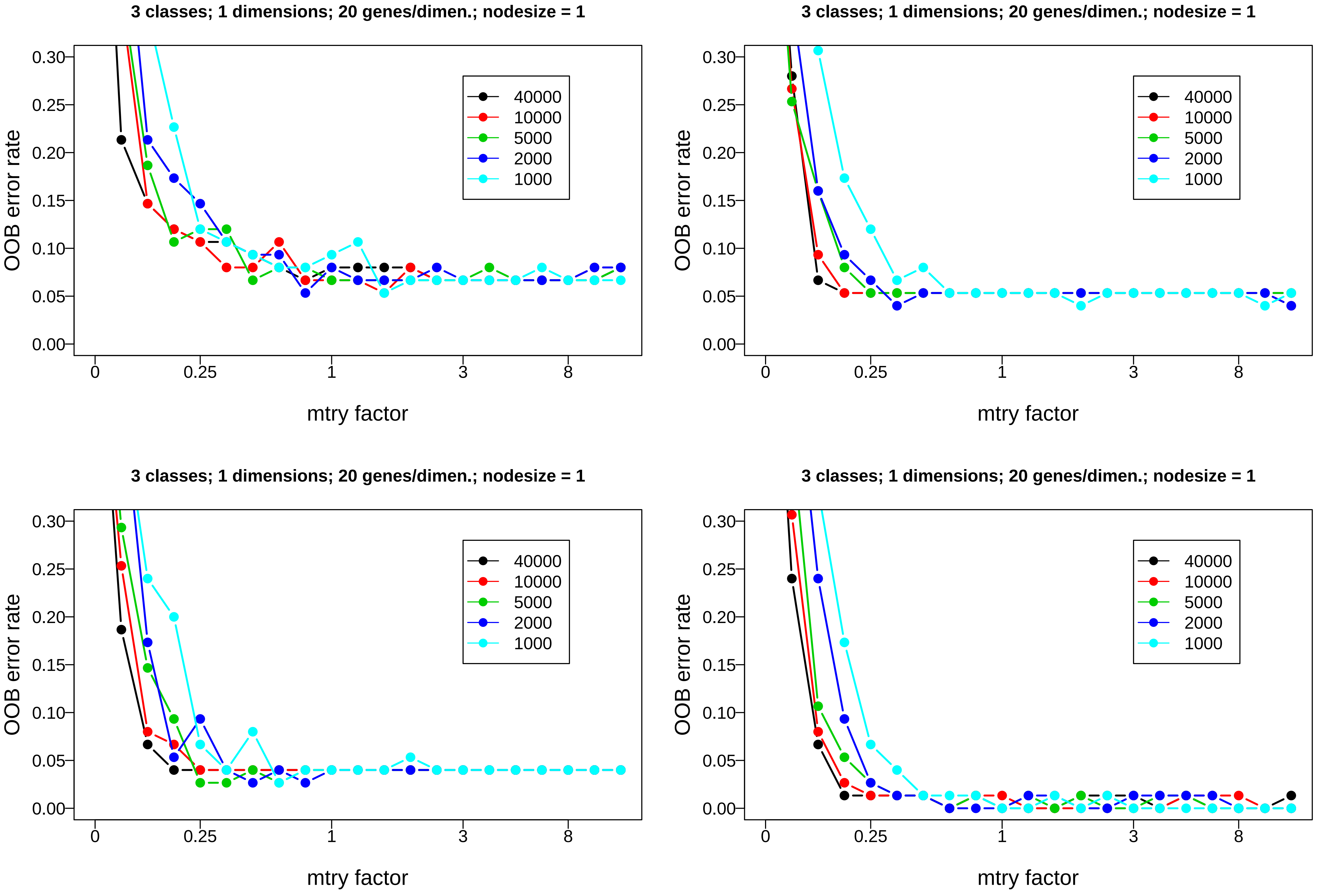

3 classes; 1 dimensions; 20 genes/dimen.; nodesize = 1
OOB error rate
mtry factor
0.30
0.25
0.20
0.15
0.10
0.05
0.00
0
0.25
1
3
8
40000
10000
5000
2000
1000

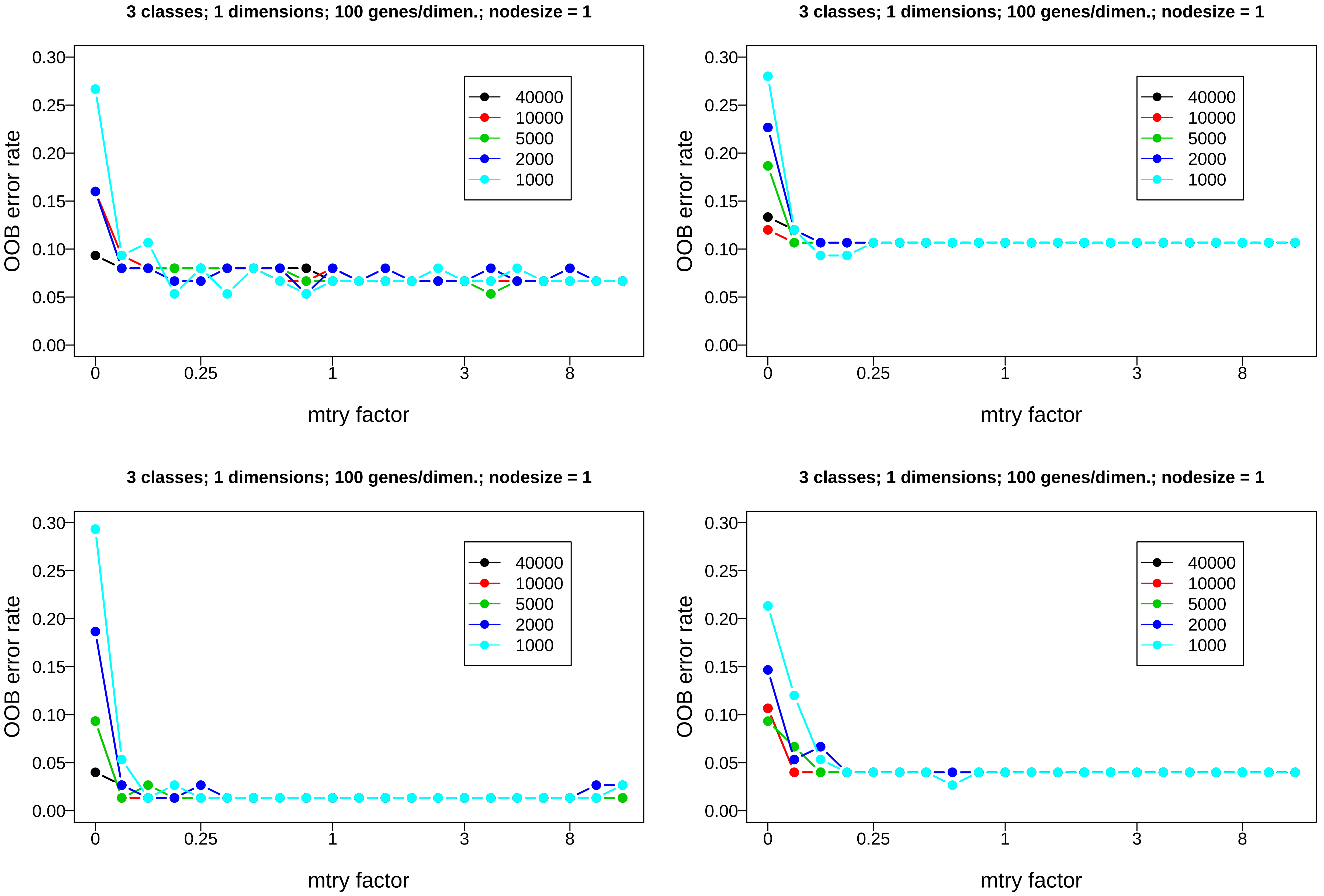

3 classes; 1 dimensions; 100 genes/dimen.; nodesize = 1
OOB error rate
0.30
0.25
0.20
0.15
0.10
0.05
0.00
mtry factor
0
0.25
1
3
8
40000
10000
5000
2000
1000
3 classes; 1 dimensions; 100 genes/dimen.; nodesize = 1
OOB error rate
mtry factor
3 classes; 1 dimensions; 100 genes/dimen.; nodesize = 1
OOB error rate
mtry factor
3 classes; 1 dimensions; 100 genes/dimen.; nodesize = 1
OOB error rate
mtry factor

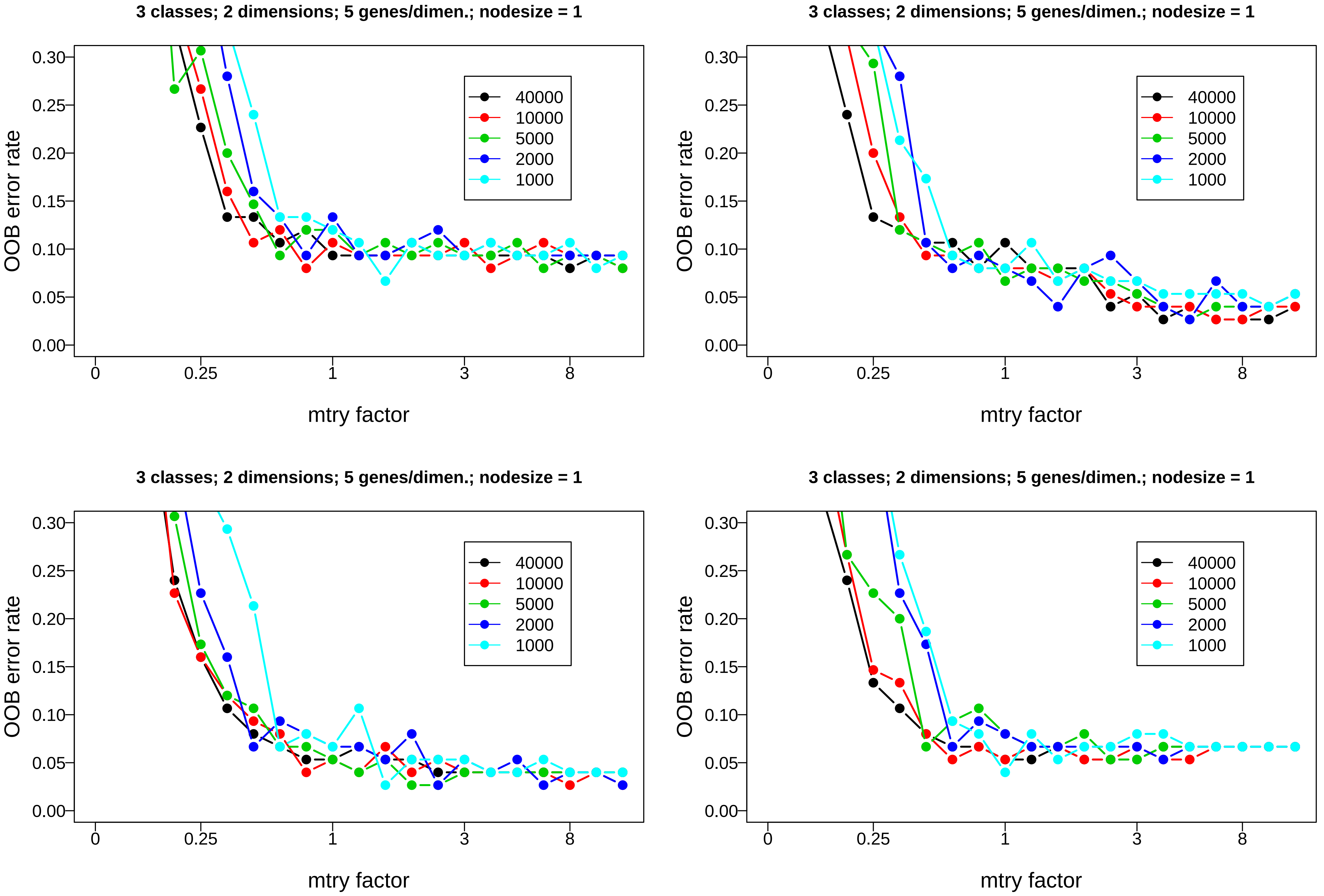
3 classes; 2 dimensions; 5 genes/dimen.; nodesize = 1
OOB error rate
mtry factor
40000
10000
5000
2000
1000
0.00
0.05
0.10
0.15
0.20
0.25
0.30
0
0.25
1
3
8

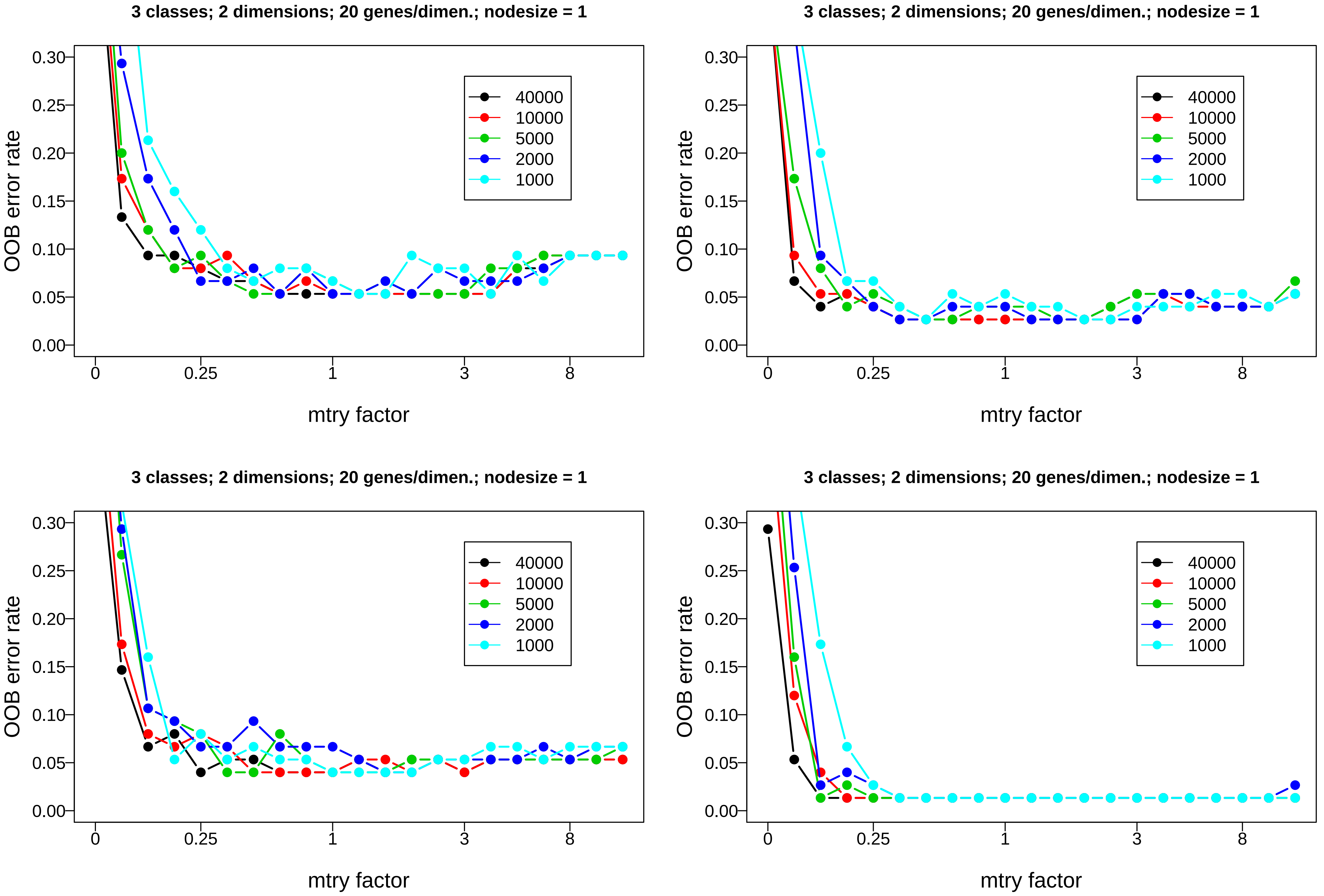

3 classes; 2 dimensions; 20 genes/dimen.; nodesize = 1
OOB error rate
mtry factor
0.00
0.05
0.10
0.15
0.20
0.25
0.30
0
0.25
1
3
8
40000
10000
5000
2000
1000

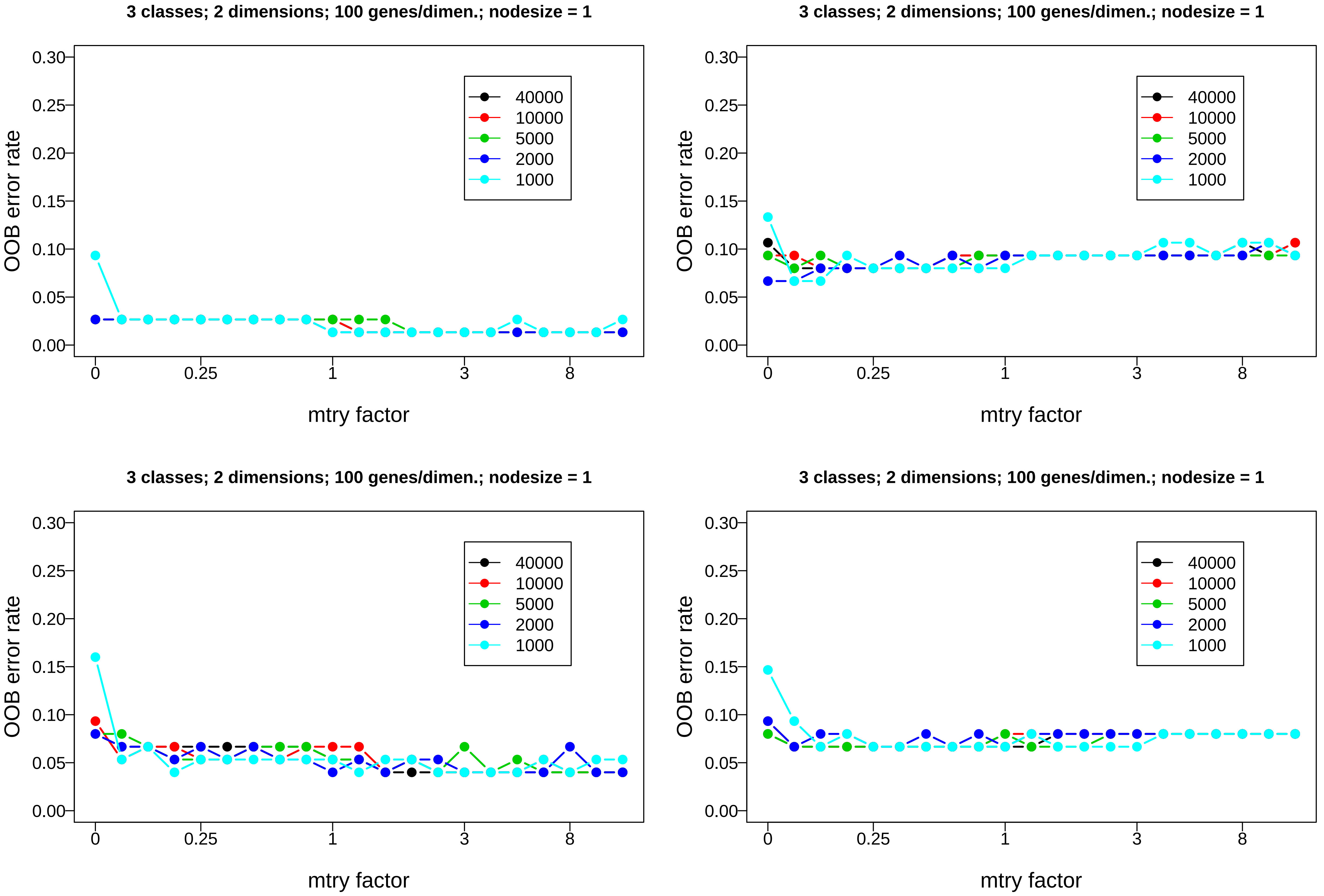
3 classes; 2 dimensions; 100 genes/dimen.; nodesize = 1
OOB error rate
mtry factor
0.00
0.05
0.10
0.15
0.20
0.25
0.30
0
0.25
1
3
8
40000
10000
5000
2000
1000

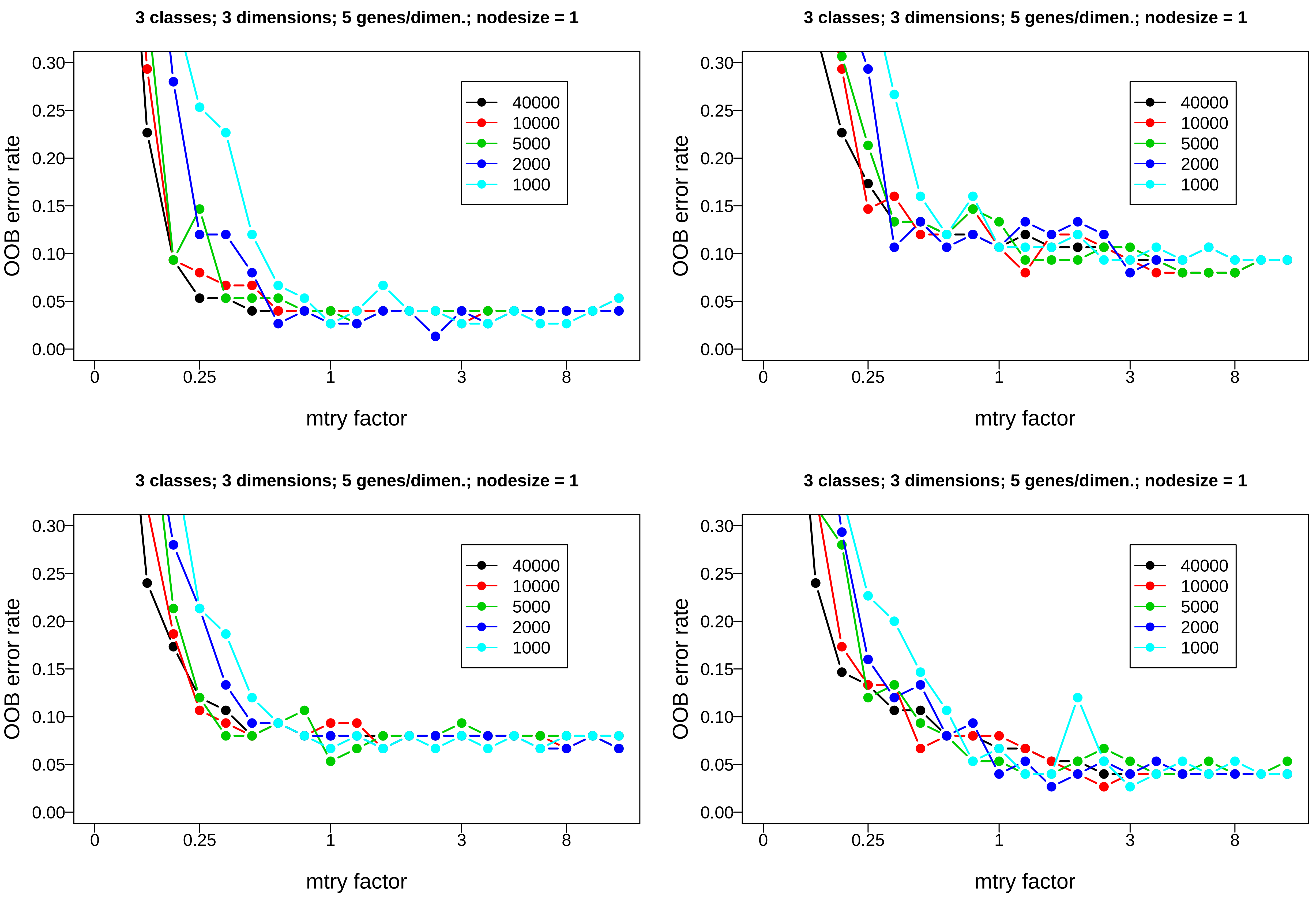
3 classes; 3 dimensions; 5 genes/dimen.; nodesize = 1
OOB error rate
0.00
0.05
0.10
0.15
0.20
0.25
0.30
mtry factor
0
0.25
1
3
8
40000
10000
5000
2000
1000
3 classes; 3 dimensions; 5 genes/dimen.; nodesize = 1
OOB error rate
mtry factor
40000
10000
5000
2000
1000
3 classes; 3 dimensions; 5 genes/dimen.; nodesize = 1
OOB error rate
mtry factor
40000
10000
5000
2000
1000
3 classes; 3 dimensions; 5 genes/dimen.; nodesize = 1
OOB error rate
mtry factor
40000
10000
5000
2000
1000

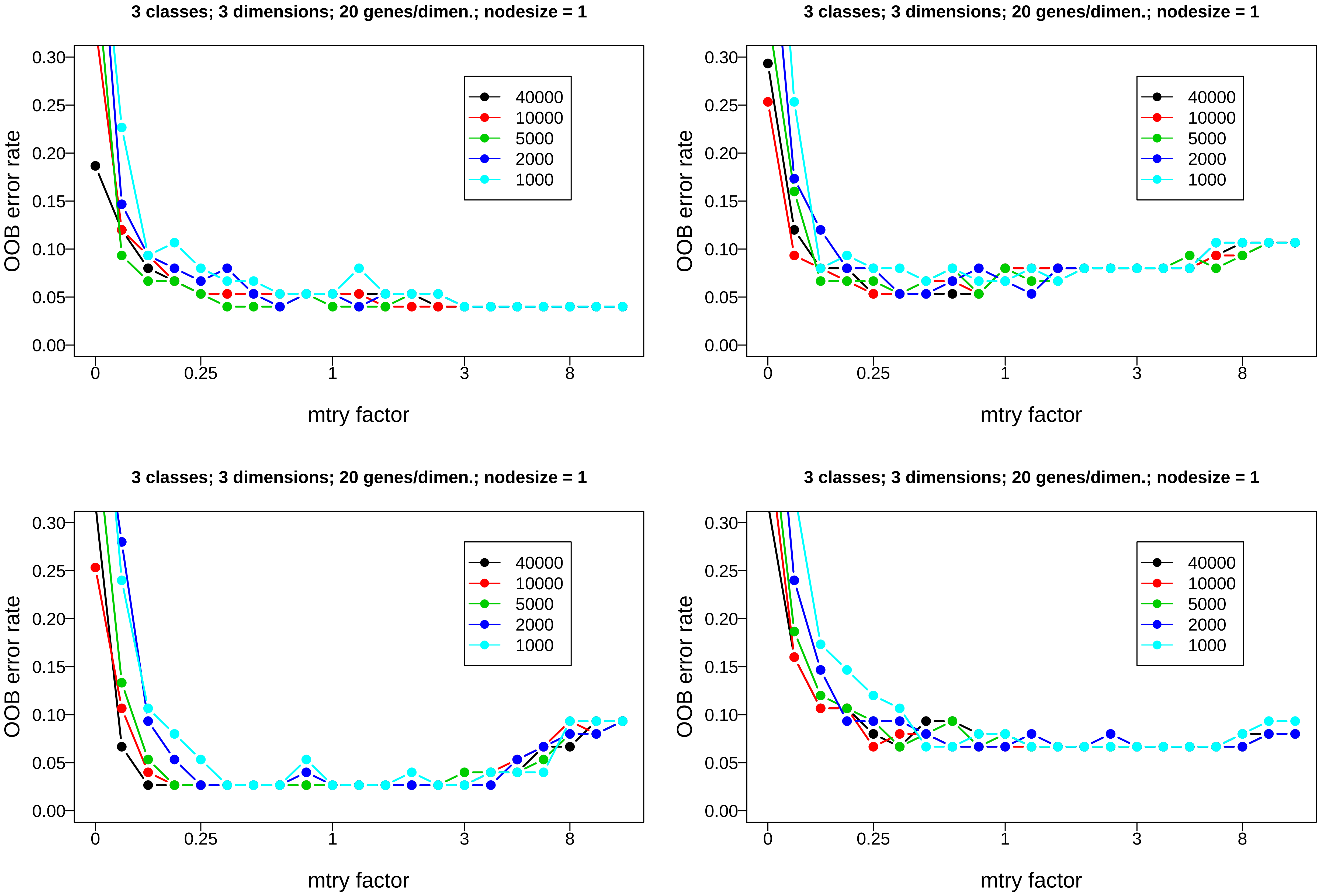

3 classes; 3 dimensions; 20 genes/dimen.; nodesize = 1
OOB error rate
mtry factor
0.30
0.25
0.20
0.15
0.10
0.05
0.00
0
0.25
1
3
8
40000
10000
5000
2000
1000

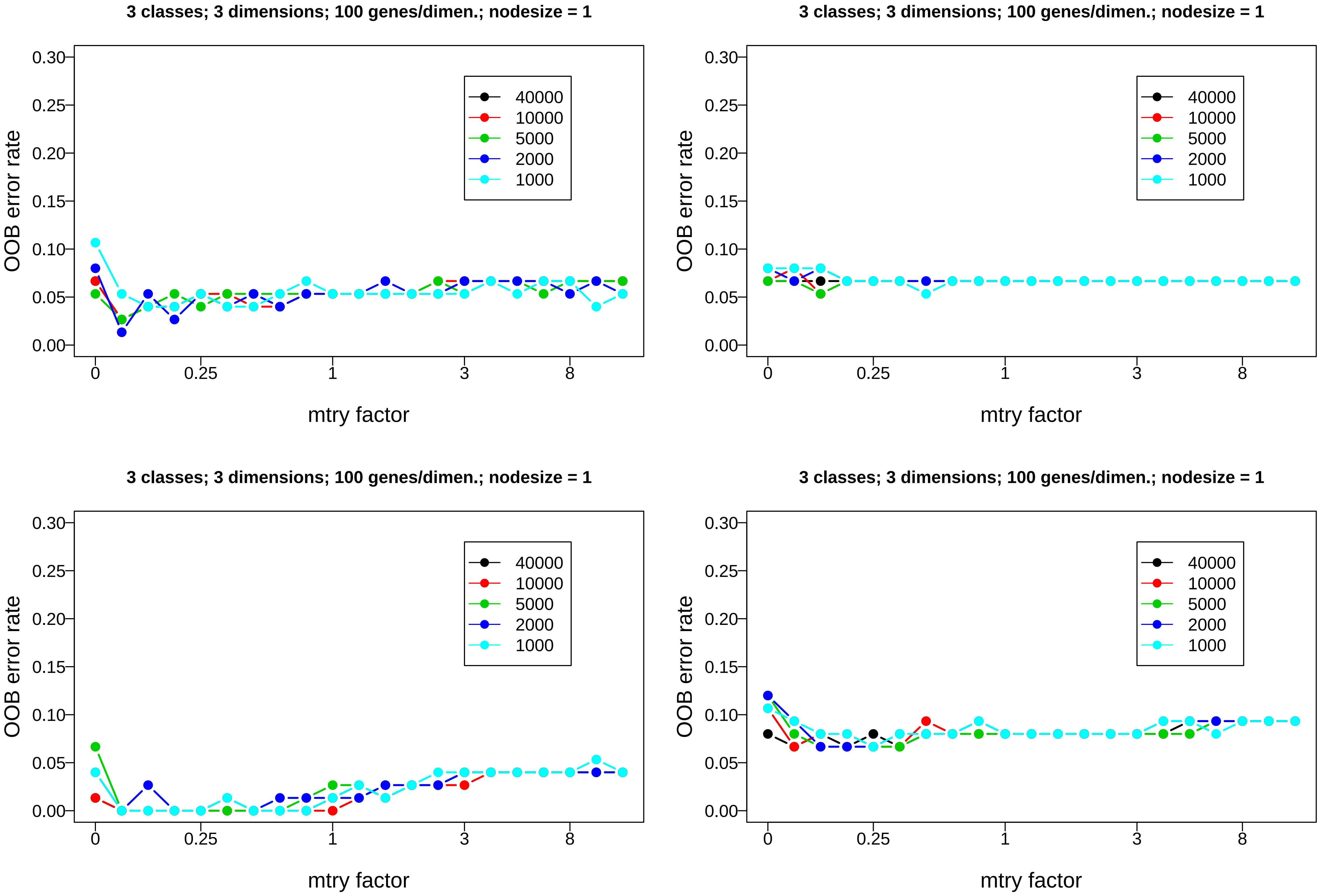

3 classes; 3 dimensions; 100 genes/dimen.; nodesize = 1
OOB error rate
mtry factor
0.30
0.25
0.20
0.15
0.10
0.05
0.00
0
0.25
1
3
8
40000
10000
5000
2000
1000

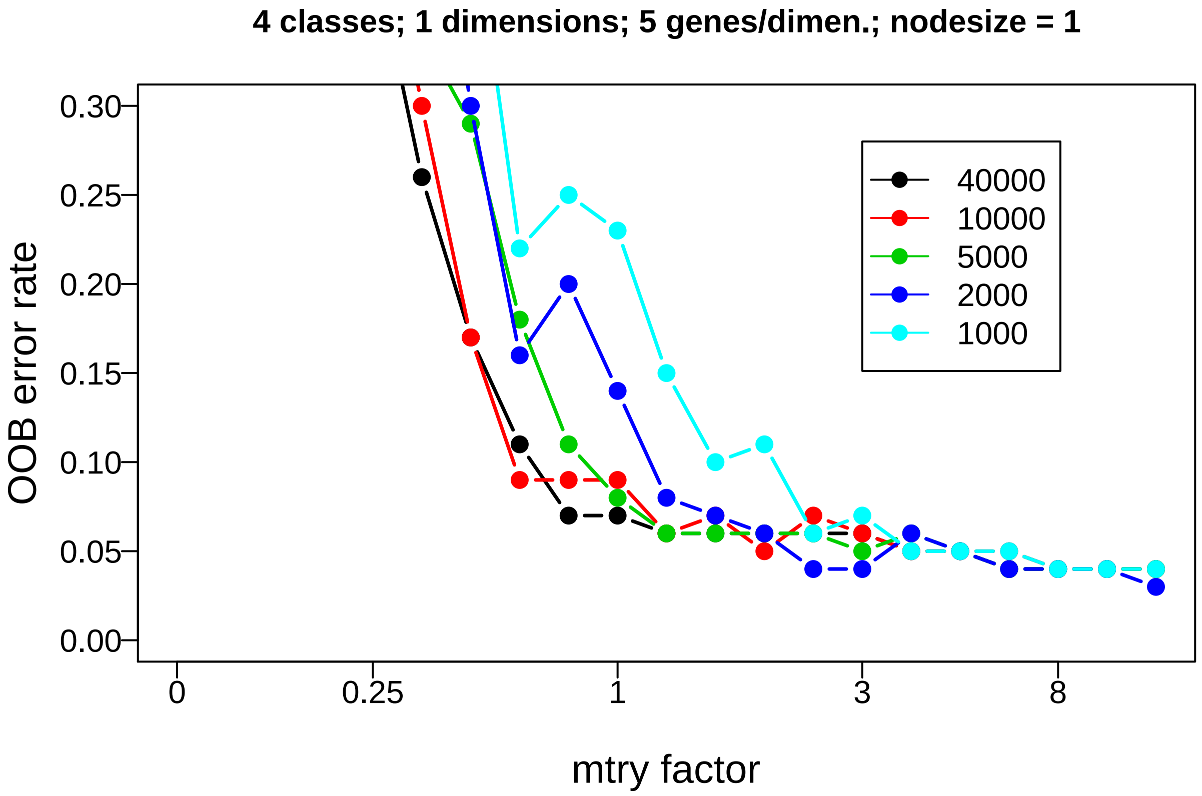
4 classes; 1 dimensions; 5 genes/dimen.; nodesize = 1
OOB error rate
0.30
0.25
0.20
0.15
0.10
0.05
0.00
0
0.25
1
3
8
mtry factor
40000
10000
5000
2000
1000

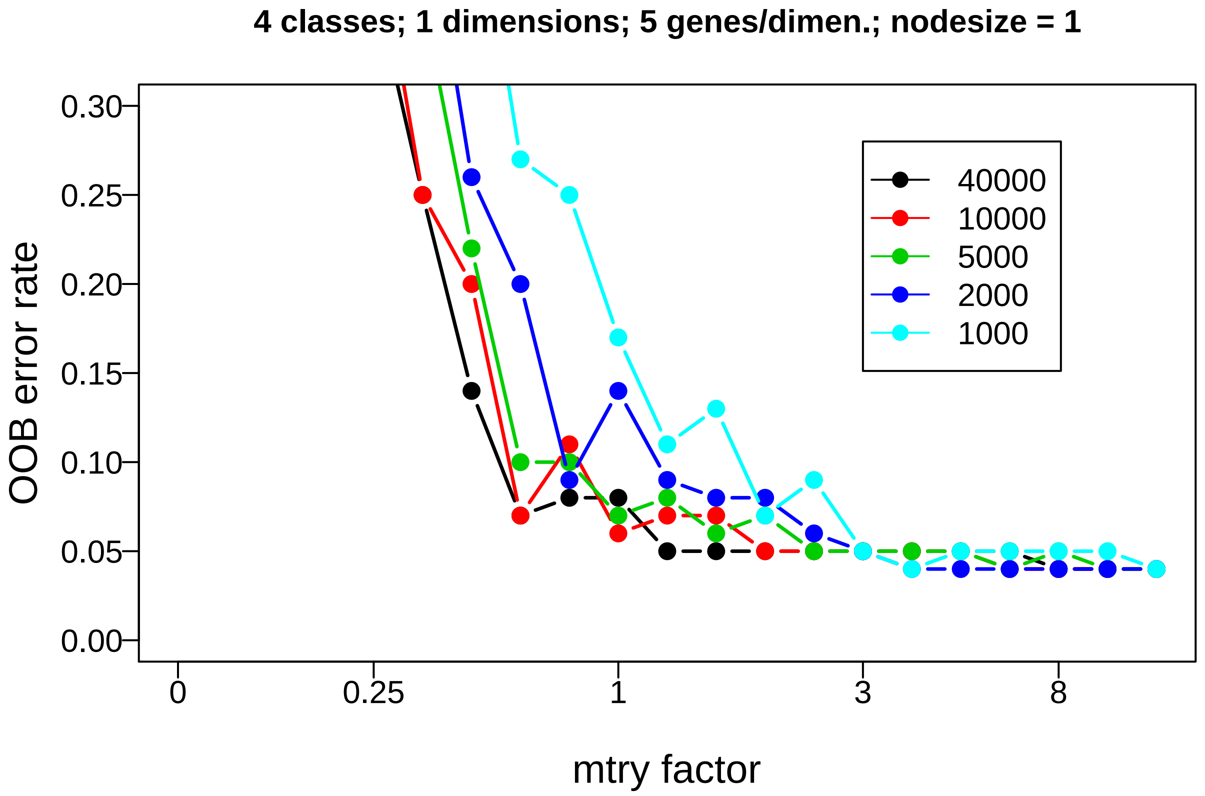
4 classes; 1 dimensions; 5 genes/dimen.; nodesize = 1
OOB error rate
0.30
0.25
0.20
0.15
0.10
0.05
0.00
0
0.25
1
3
8
mtry factor
40000
10000
5000
2000
1000

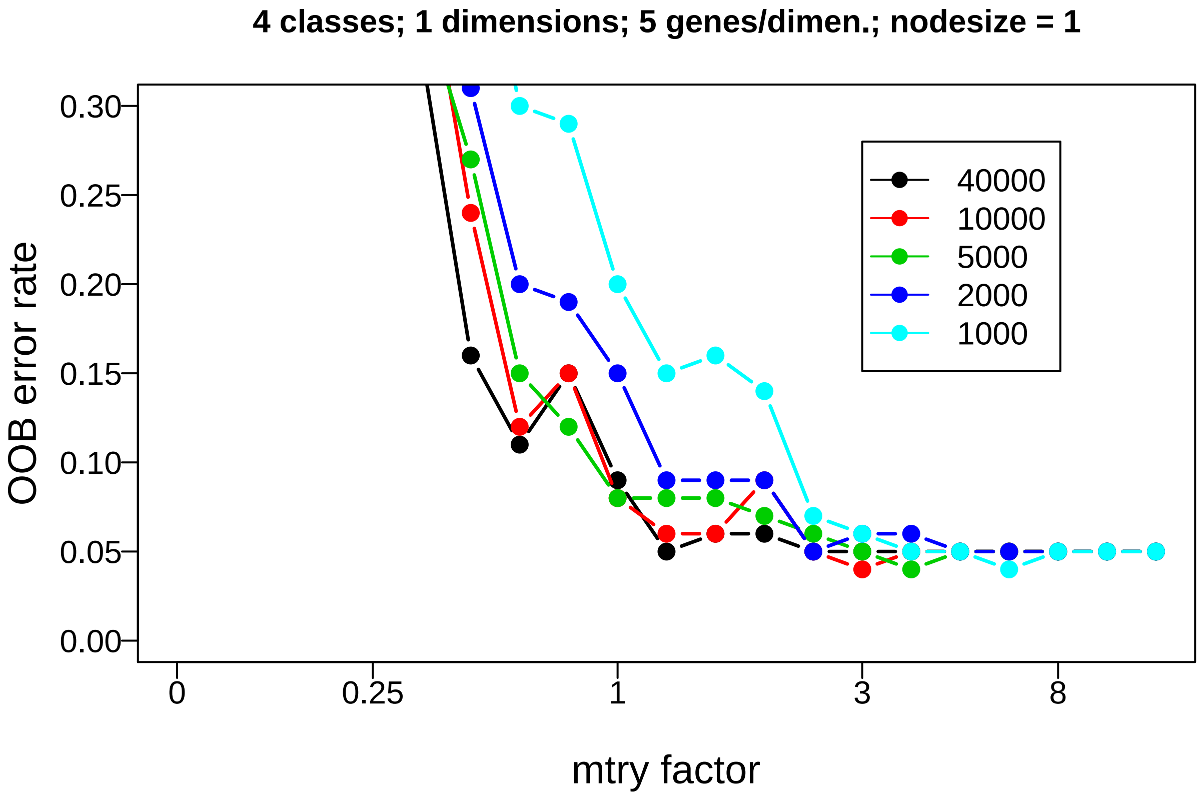
4 classes; 1 dimensions; 5 genes/dimen.; nodesize = 1
OOB error rate
0.30
0.25
0.20
0.15
0.10
0.05
0.00
0
0.25
1
3
8
mtry factor
40000
10000
5000
2000
1000

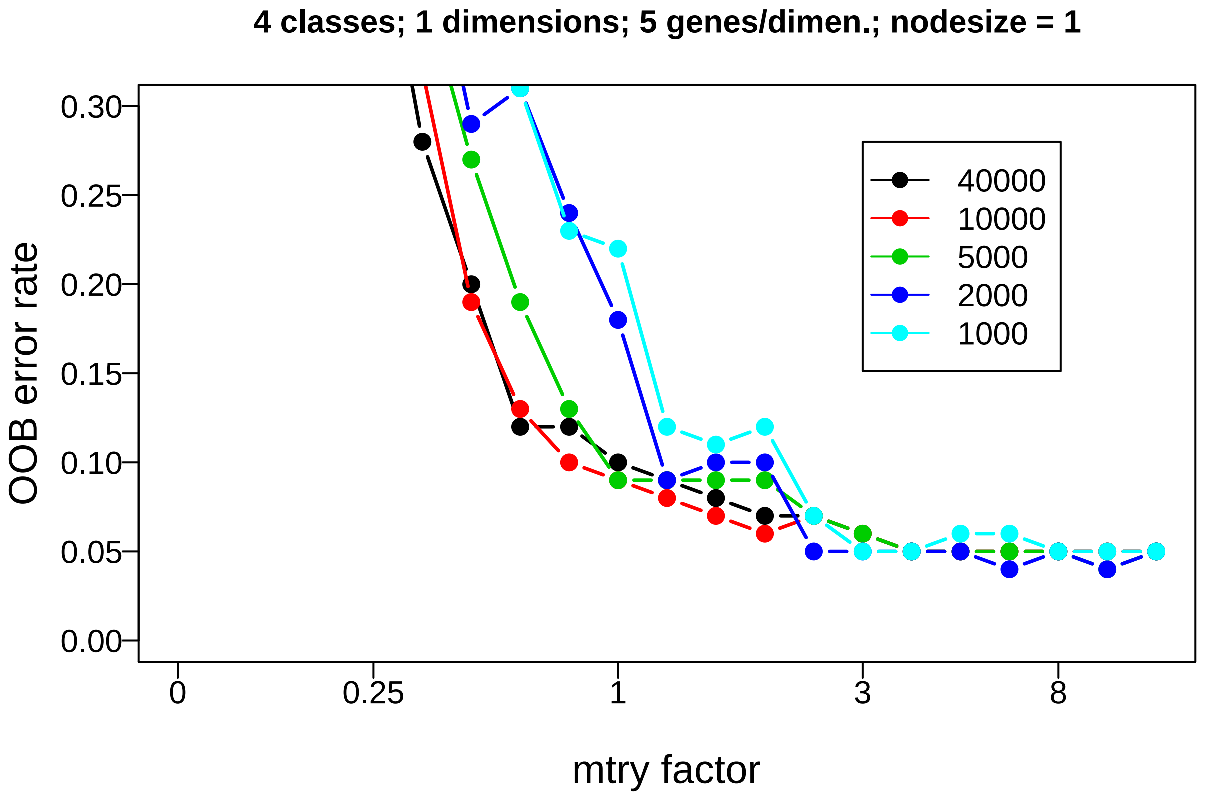
4 classes; 1 dimensions; 5 genes/dimen.; nodesize = 1
OOB error rate
0.30
0.25
0.20
0.15
0.10
0.05
0.00
0
0.25
1
3
8
mtry factor
40000
10000
5000
2000
1000

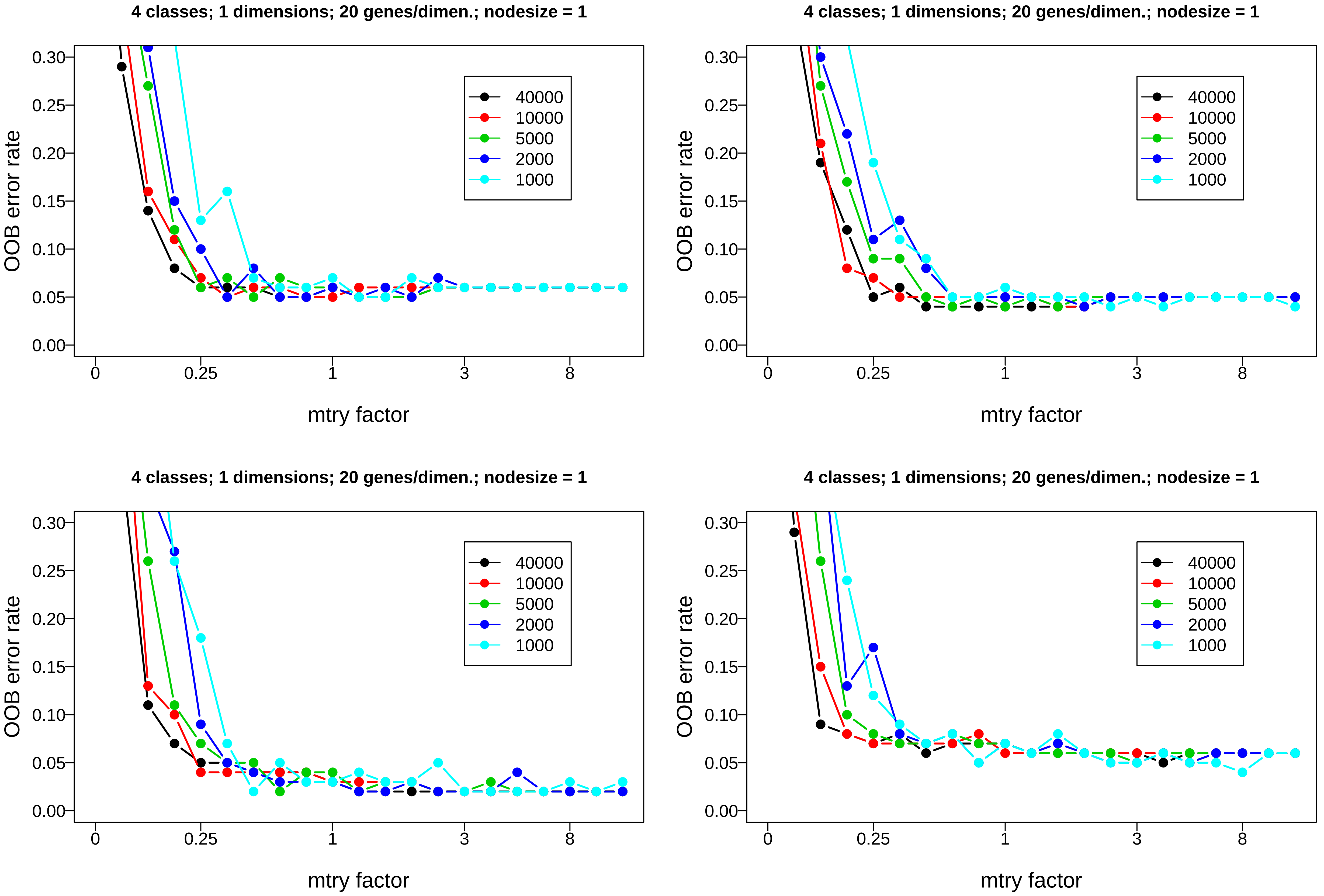
4 classes; 1 dimensions; 20 genes/dimen.; nodesize = 1
OOB error rate
0.00
0.05
0.10
0.15
0.20
0.25
0.30
0
0.25
1
3
8
mtry factor
40000
10000
5000
2000
1000

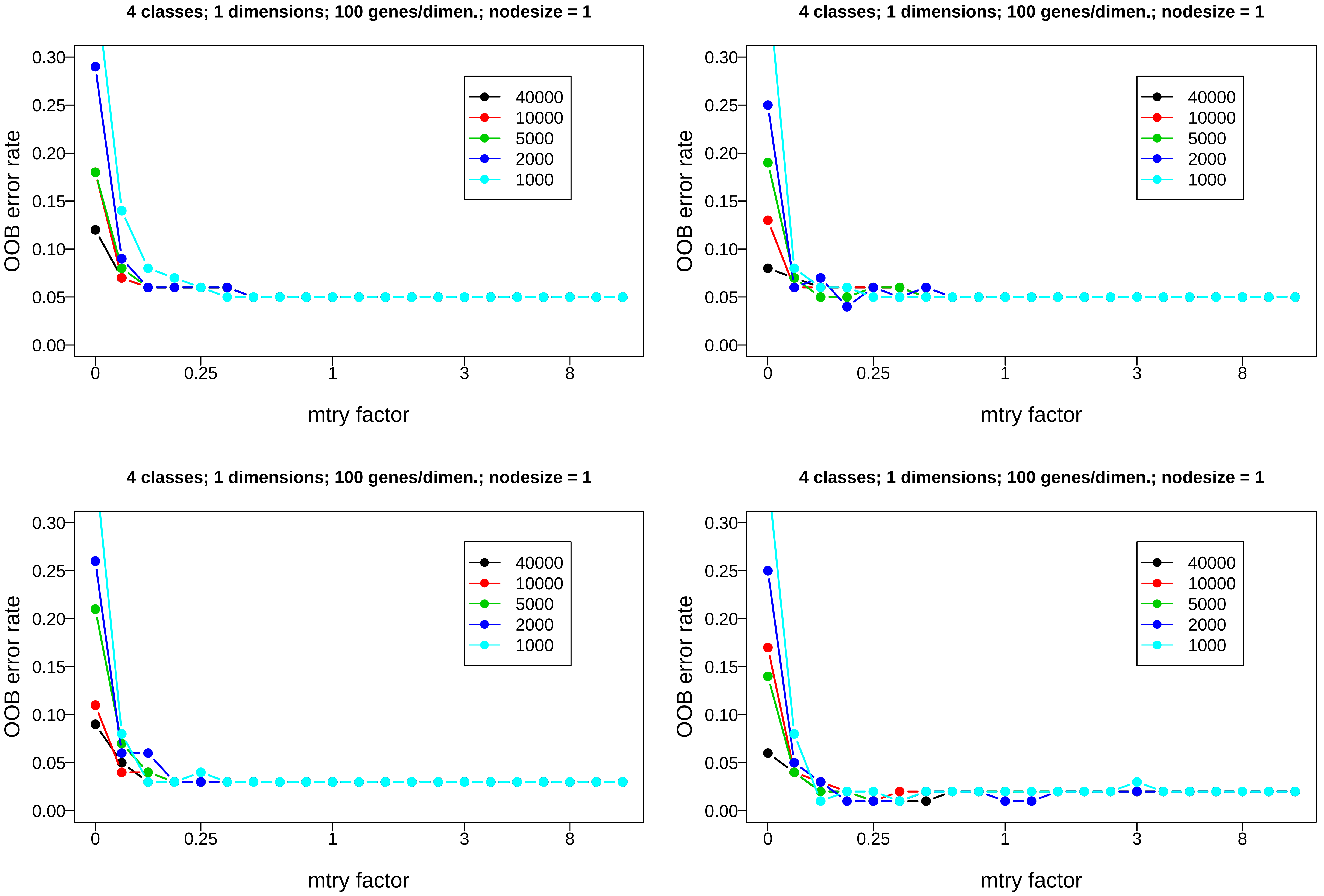
4 classes; 1 dimensions; 100 genes/dimen.; nodesize = 1
OOB error rate
mtry factor
0.00
0.05
0.10
0.15
0.20
0.25
0.30
0
0.25
1
3
8
40000
10000
5000
2000
1000

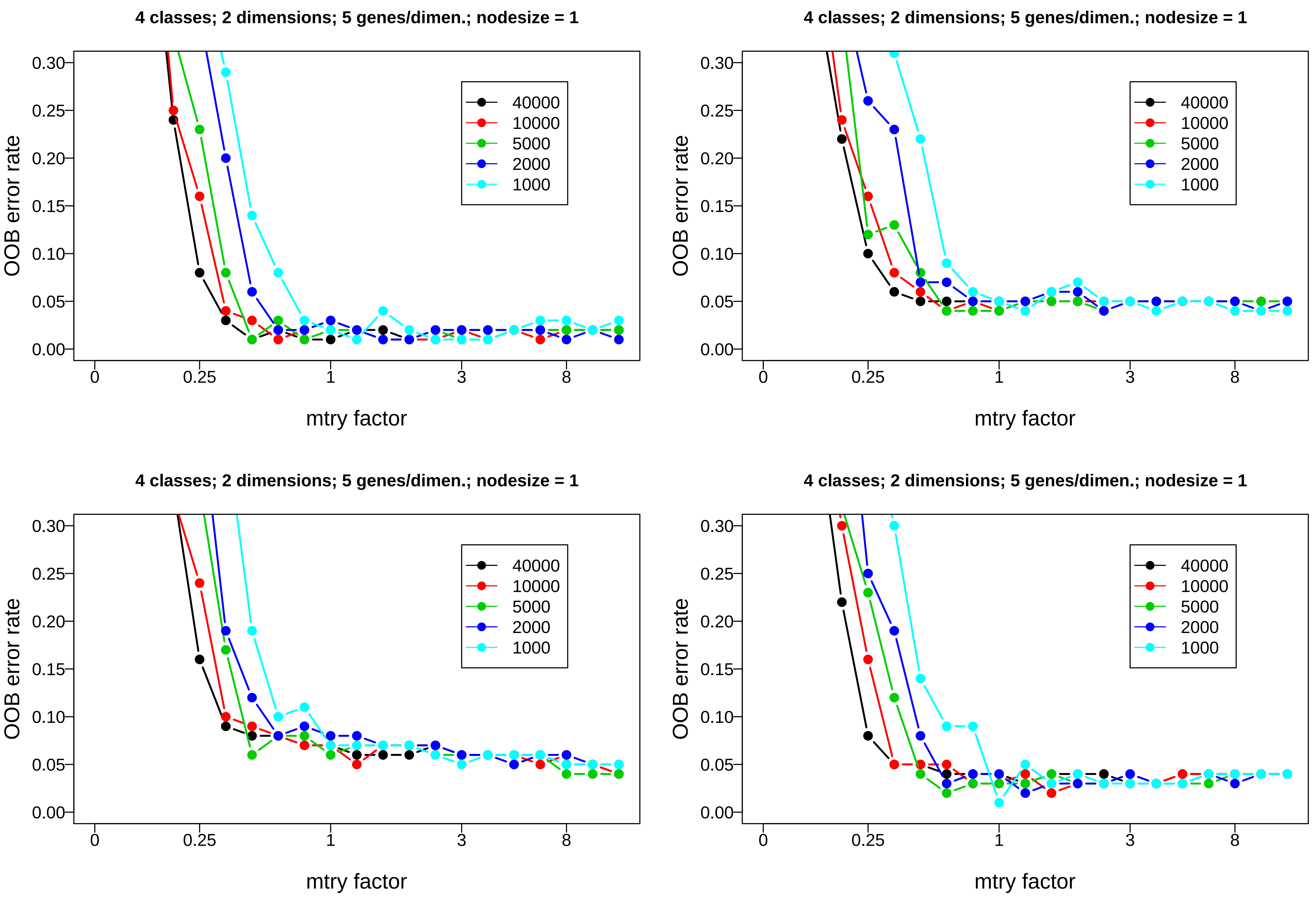
4 classes; 2 dimensions; 5 genes/dimen.; nodesize = 1
OOB error rate
0.00
0.05
0.10
0.15
0.20
0.25
0.30
mtry factor
0
0.25
1
3
8
40000
10000
5000
2000
1000

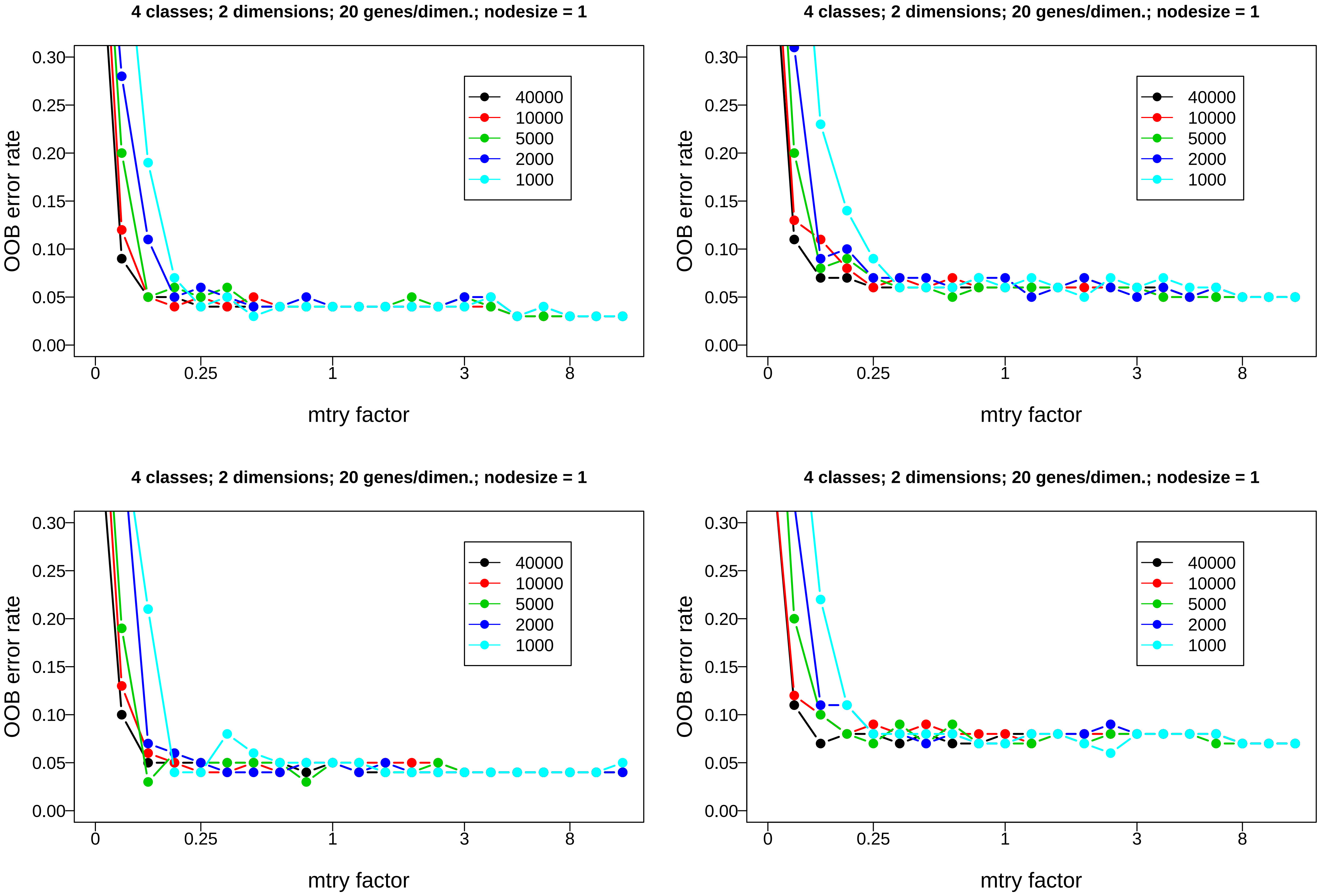
4 classes; 2 dimensions; 20 genes/dimen.; nodesize = 1
OOB error rate
mtry factor
40000
10000
5000
2000
1000
0.00
0.05
0.10
0.15
0.20
0.25
0.30
0
0.25
1
3
8
4 classes; 2 dimensions; 20 genes/dimen.; nodesize = 1
4 classes; 2 dimensions; 20 genes/dimen.; nodesize = 1
4 classes; 2 dimensions; 20 genes/dimen.; nodesize = 1

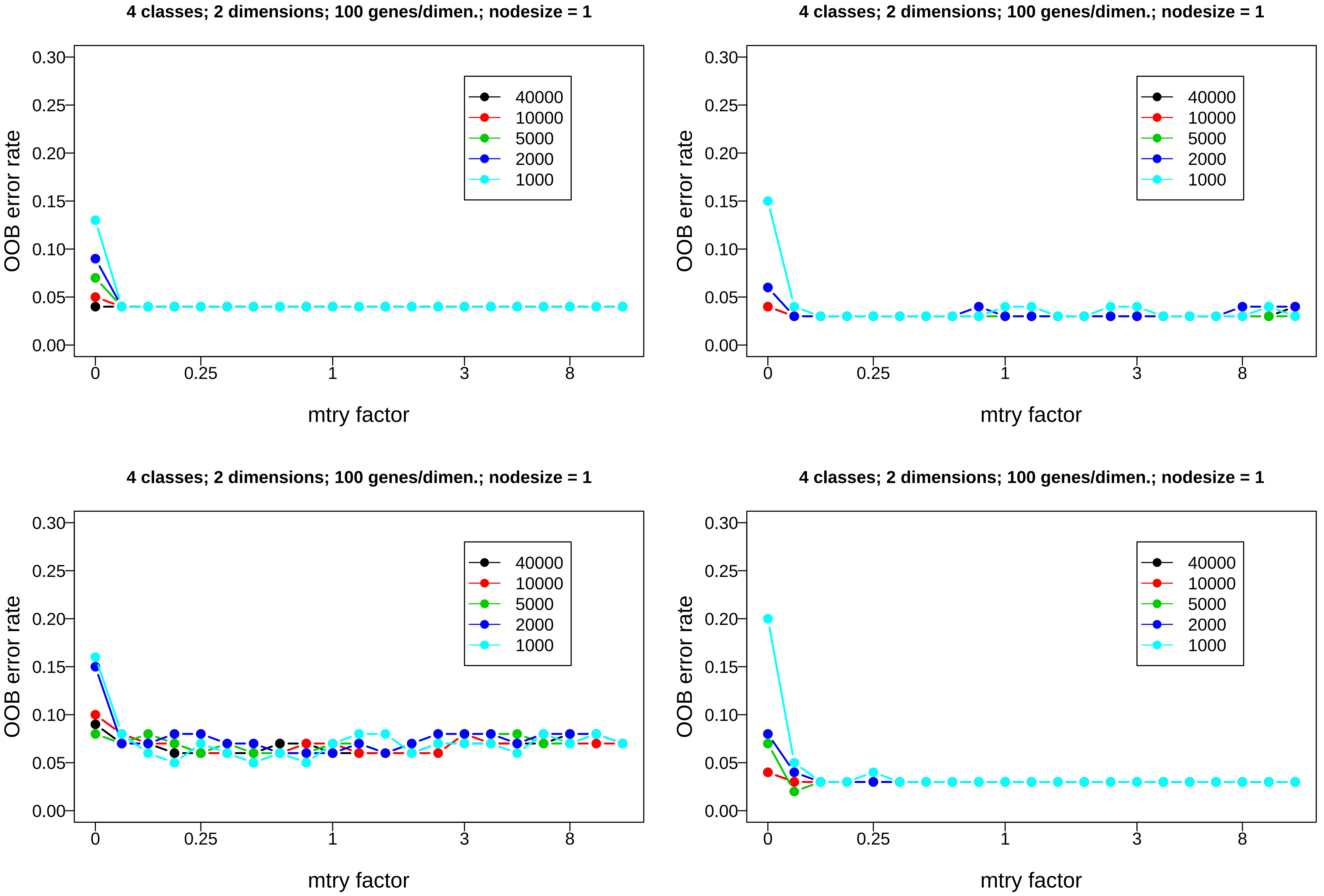
4 classes; 2 dimensions; 100 genes/dimen.; nodesize = 1
OOB error rate
mtry factor
40000
10000
5000
2000
1000
0.00
0.05
0.10
0.15
0.20
0.25
0.30
0
0.25
1
3
8

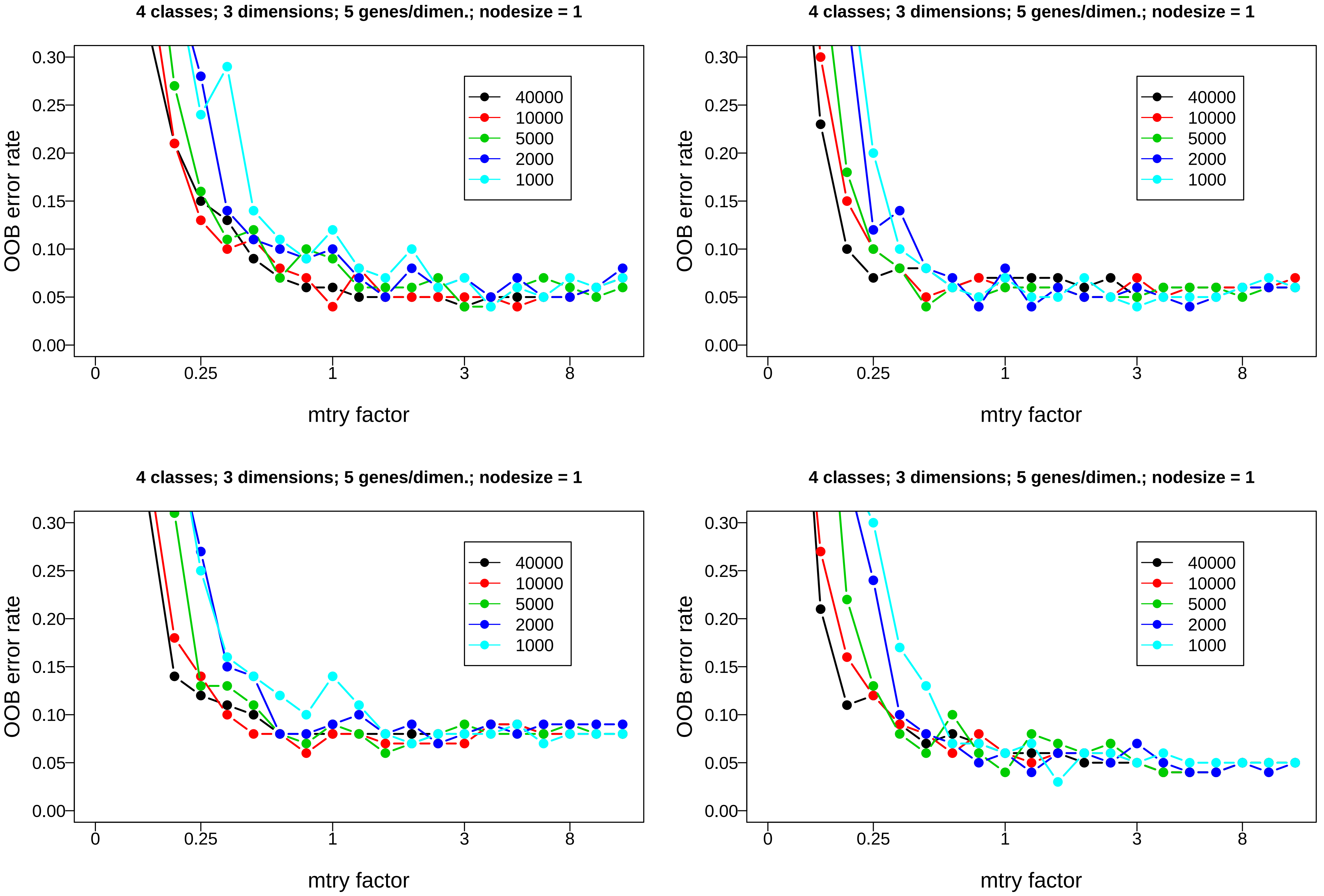
4 classes; 3 dimensions; 5 genes/dimen.; nodesize = 1
OOB error rate
mtry factor
40000
10000
5000
2000
1000
0.00
0.05
0.10
0.15
0.20
0.25
0.30
0
0.25
1
3
8

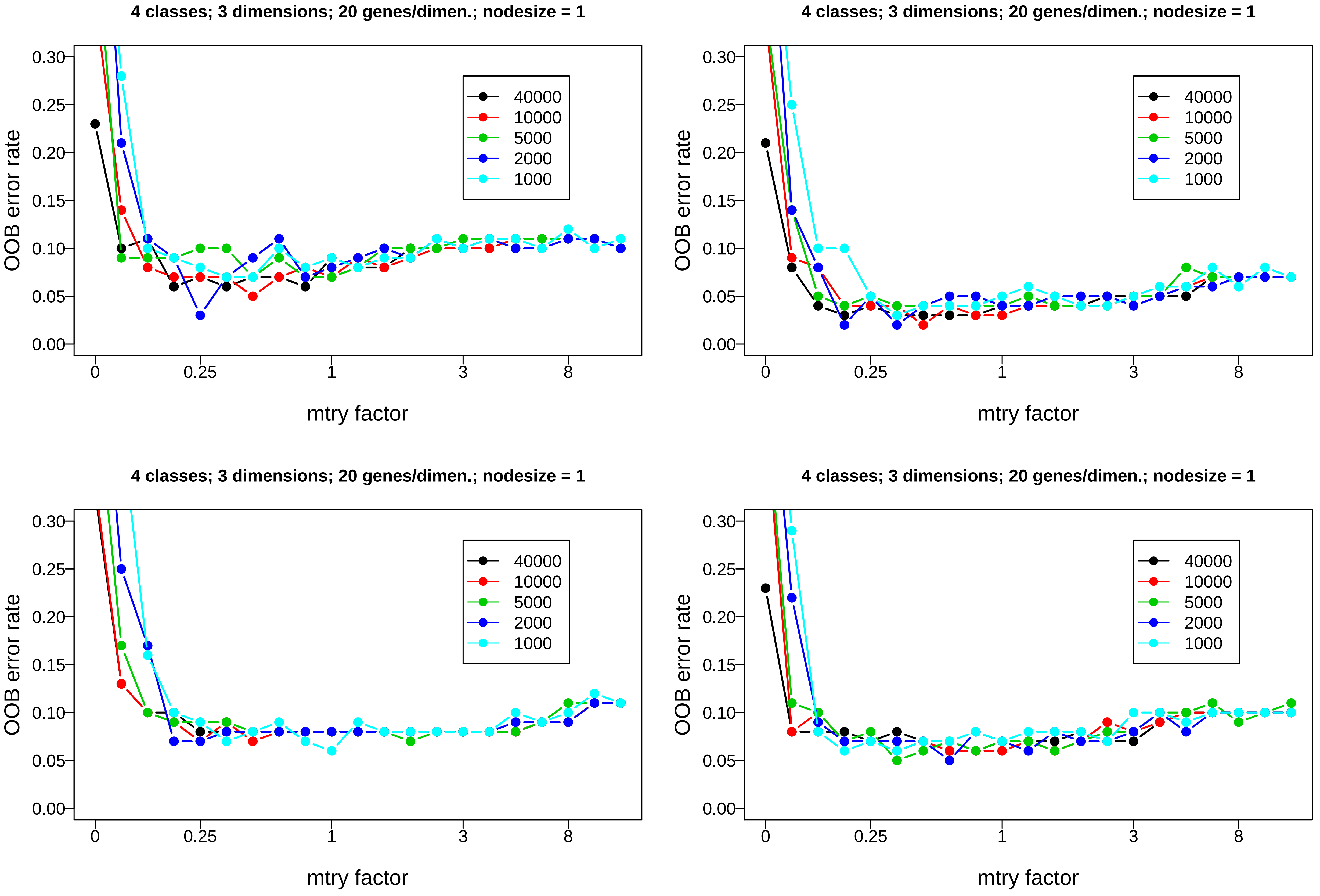

4 classes; 3 dimensions; 20 genes/dimen.; nodesize = 1
OOB error rate
0.00
0.05
0.10
0.15
0.20
0.25
0.30
mtry factor
0
0.25
1
3
8
40000
10000
5000
2000
1000

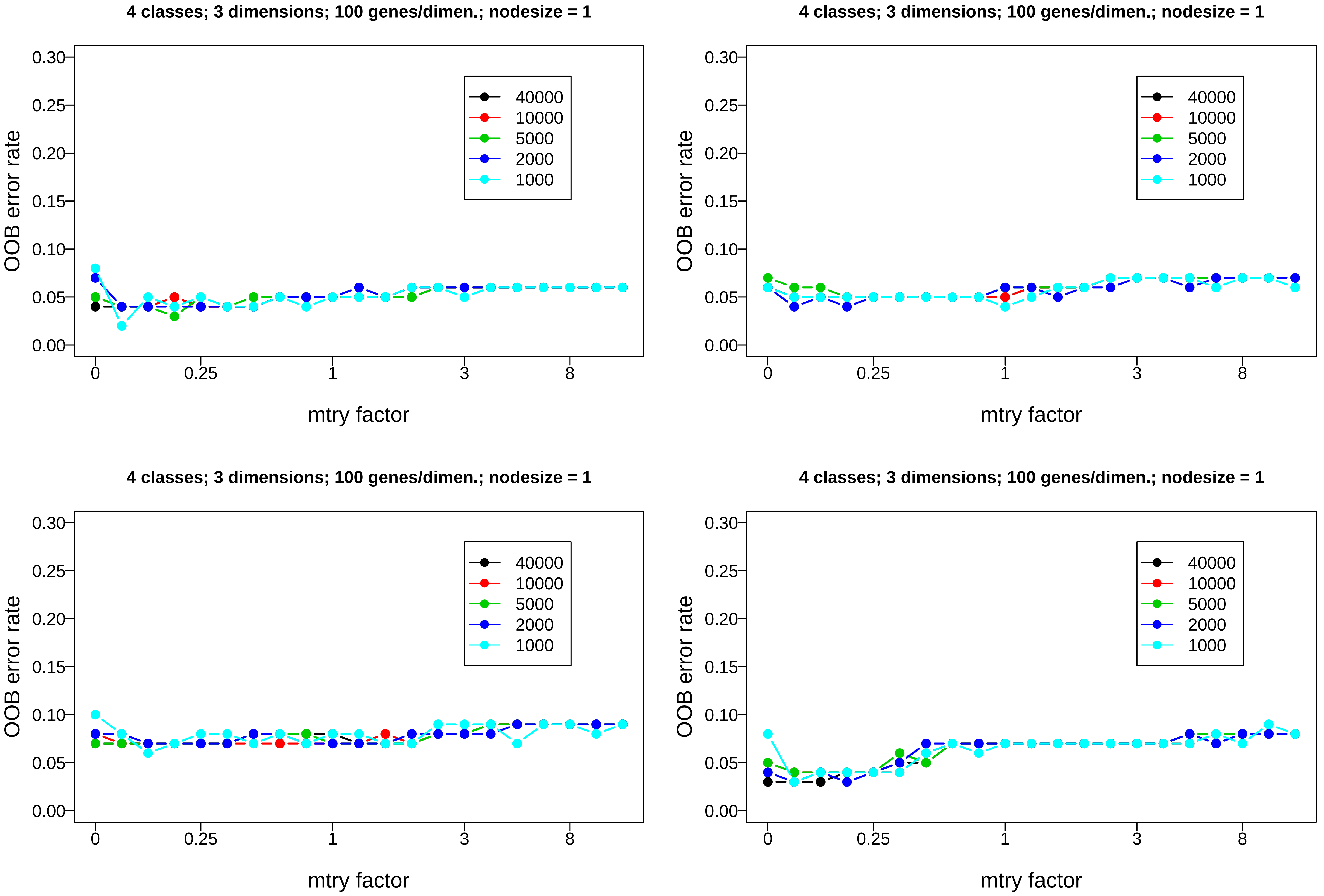

4 classes; 3 dimensions; 100 genes/dimen.; nodesize = 1
OOB error rate
mtry factor
40000
10000
5000
2000
1000
0.00
0.05
0.10
0.15
0.20
0.25
0.30
0
0.25
1
3
8
4 classes; 3 dimensions; 100 genes/dimen.; nodesize = 1
OOB error rate
mtry factor
40000
10000
5000
2000
1000
4 classes; 3 dimensions; 100 genes/dimen.; nodesize = 1
OOB error rate
mtry factor
40000
10000
5000
2000
1000
4 classes; 3 dimensions; 100 genes/dimen.; nodesize = 1
OOB error rate
mtry factor
40000
10000
5000
2000
1000

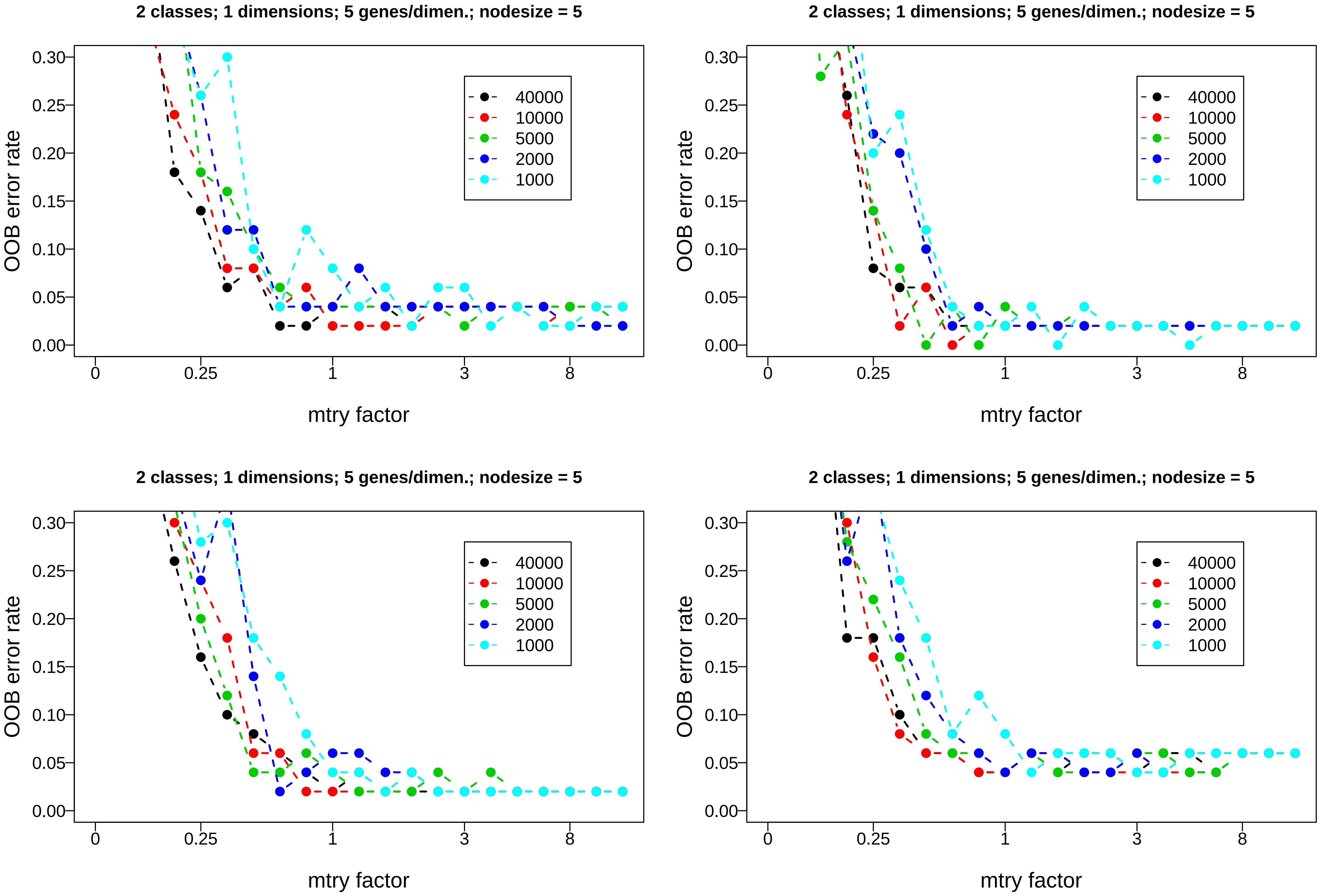
2 classes; 1 dimensions; 5 genes/dimen.; nodesize = 5
OOB error rate
0.00 0.05 0.10 0.15 0.20 0.25 0.30
0 0.25 1 3 8
mtry factor
40000
10000
5000
2000
1000
2 classes; 1 dimensions; 5 genes/dimen.; nodesize = 5
OOB error rate
0.00 0.05 0.10 0.15 0.20 0.25 0.30
0 0.25 1 3 8
mtry factor
40000
10000
5000
2000
1000
2 classes; 1 dimensions; 5 genes/dimen.; nodesize = 5
OOB error rate
0.00 0.05 0.10 0.15 0.20 0.25 0.30
0 0.25 1 3 8
mtry factor
40000
10000
5000
2000
1000
2 classes; 1 dimensions; 5 genes/dimen.; nodesize = 5
OOB error rate
0.00 0.05 0.10 0.15 0.20 0.25 0.30
0 0.25 1 3 8
mtry factor
40000
10000
5000
2000
1000

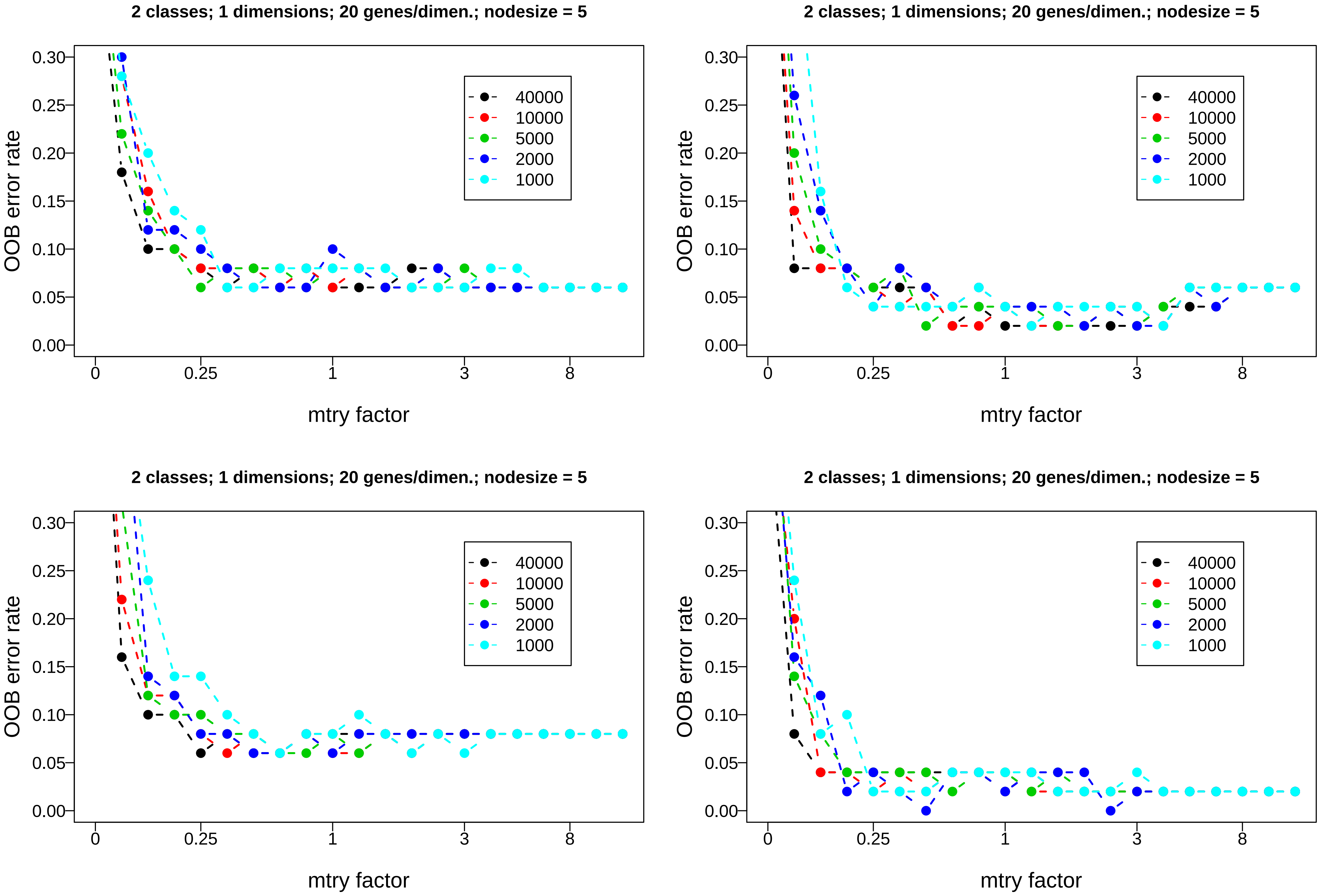
2 classes; 1 dimensions; 20 genes/dimen.; nodesize = 5
OOB error rate
mtry factor
0.00
0.05
0.10
0.15
0.20
0.25
0.30
0
0.25
1
3
8
40000
10000
5000
2000
1000
2 classes; 1 dimensions; 20 genes/dimen.; nodesize = 5
OOB error rate
mtry factor
40000
10000
5000
2000
1000
2 classes; 1 dimensions; 20 genes/dimen.; nodesize = 5
OOB error rate
mtry factor
40000
10000
5000
2000
1000
2 classes; 1 dimensions; 20 genes/dimen.; nodesize = 5
OOB error rate
mtry factor
40000
10000
5000
2000
1000

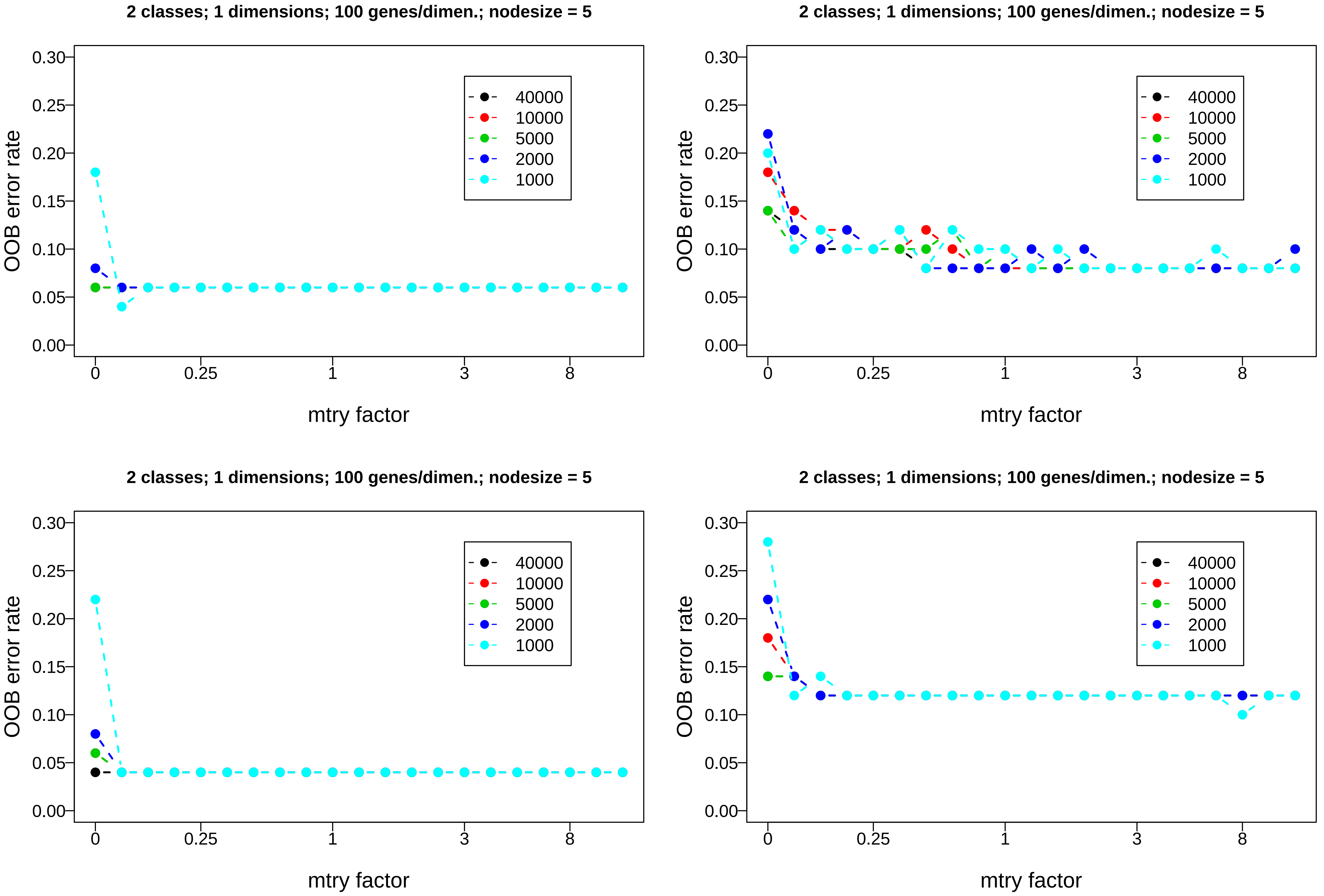

2 classes; 1 dimensions; 100 genes/dimen.; nodesize = 5
OOB error rate
mtry factor
0.00
0.05
0.10
0.15
0.20
0.25
0.30
0
0.25
1
3
8
40000
10000
5000
2000
1000
2 classes; 1 dimensions; 100 genes/dimen.; nodesize = 5
OOB error rate
mtry factor
40000
10000
5000
2000
1000
2 classes; 1 dimensions; 100 genes/dimen.; nodesize = 5
OOB error rate
mtry factor
40000
10000
5000
2000
1000
2 classes; 1 dimensions; 100 genes/dimen.; nodesize = 5
OOB error rate
mtry factor
40000
10000
5000
2000
1000

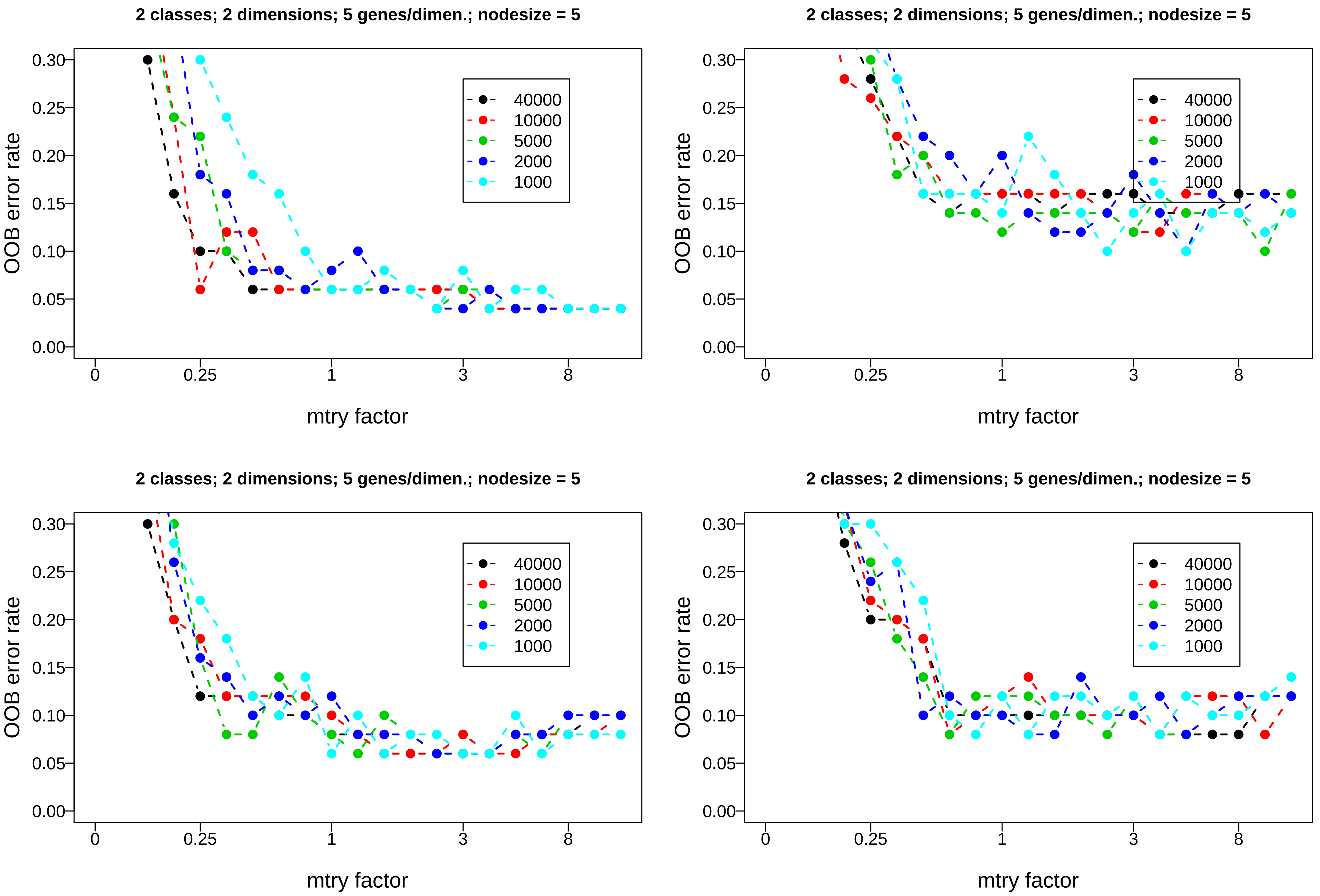
2 classes; 2 dimensions; 5 genes/dimen.; nodesize = 5
OOB error rate
0.00
0.05
0.10
0.15
0.20
0.25
0.30
mtry factor
0
0.25
1
3
8
40000
10000
5000
2000
1000
2 classes; 2 dimensions; 5 genes/dimen.; nodesize = 5
OOB error rate
mtry factor
40000
10000
5000
2000
1000
2 classes; 2 dimensions; 5 genes/dimen.; nodesize = 5
OOB error rate
mtry factor
40000
10000
5000
2000
1000
2 classes; 2 dimensions; 5 genes/dimen.; nodesize = 5
OOB error rate
mtry factor
40000
10000
5000
2000
1000

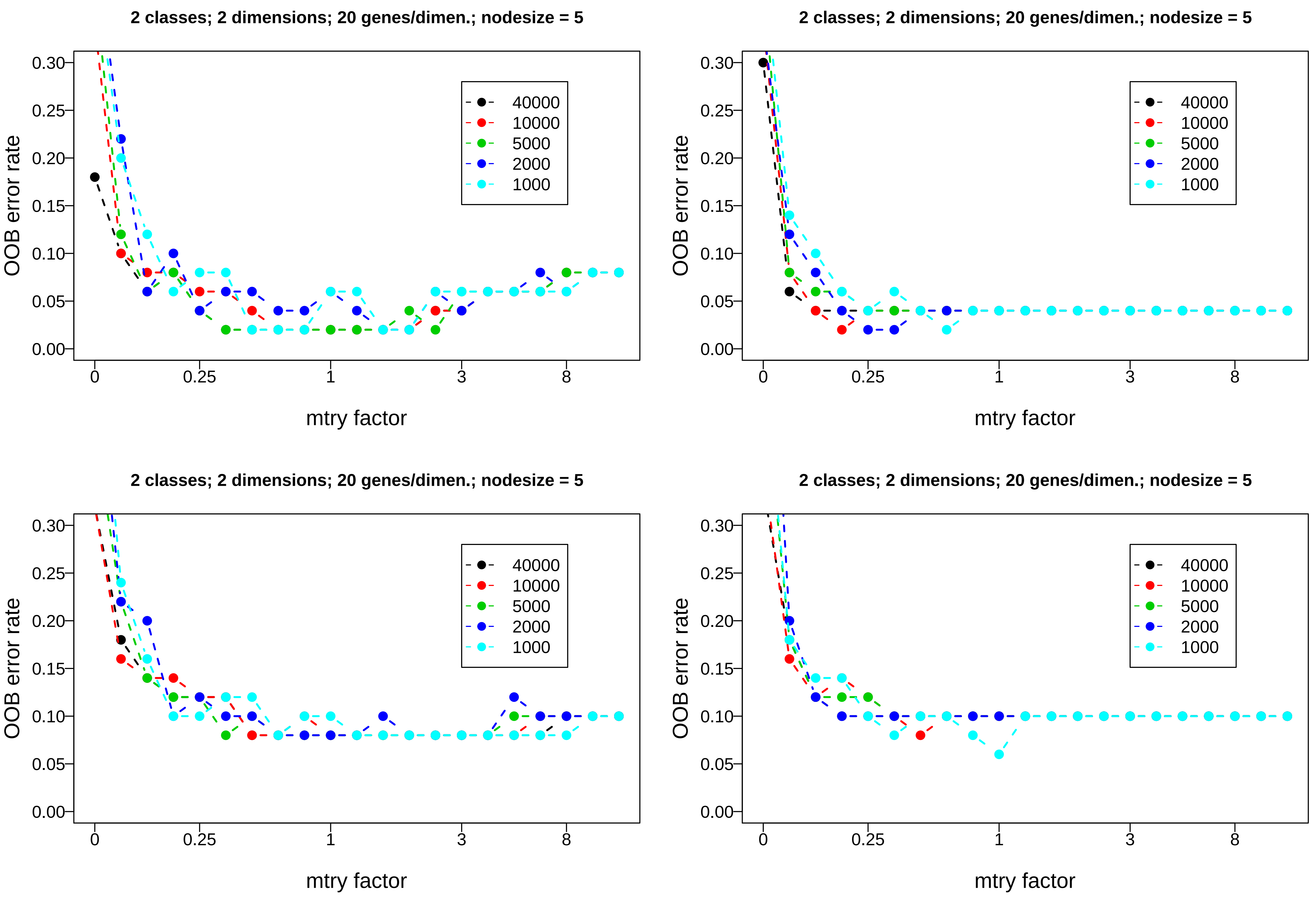
2 classes; 2 dimensions; 20 genes/dimen.; nodesize = 5
OOB error rate
0.00
0.05
0.10
0.15
0.20
0.25
0.30
mtry factor
0
0.25
1
3
8
40000
10000
5000
2000
1000
2 classes; 2 dimensions; 20 genes/dimen.; nodesize = 5
OOB error rate
mtry factor
40000
10000
5000
2000
1000
2 classes; 2 dimensions; 20 genes/dimen.; nodesize = 5
OOB error rate
mtry factor
40000
10000
5000
2000
1000
2 classes; 2 dimensions; 20 genes/dimen.; nodesize = 5
OOB error rate
mtry factor
40000
10000
5000
2000
1000

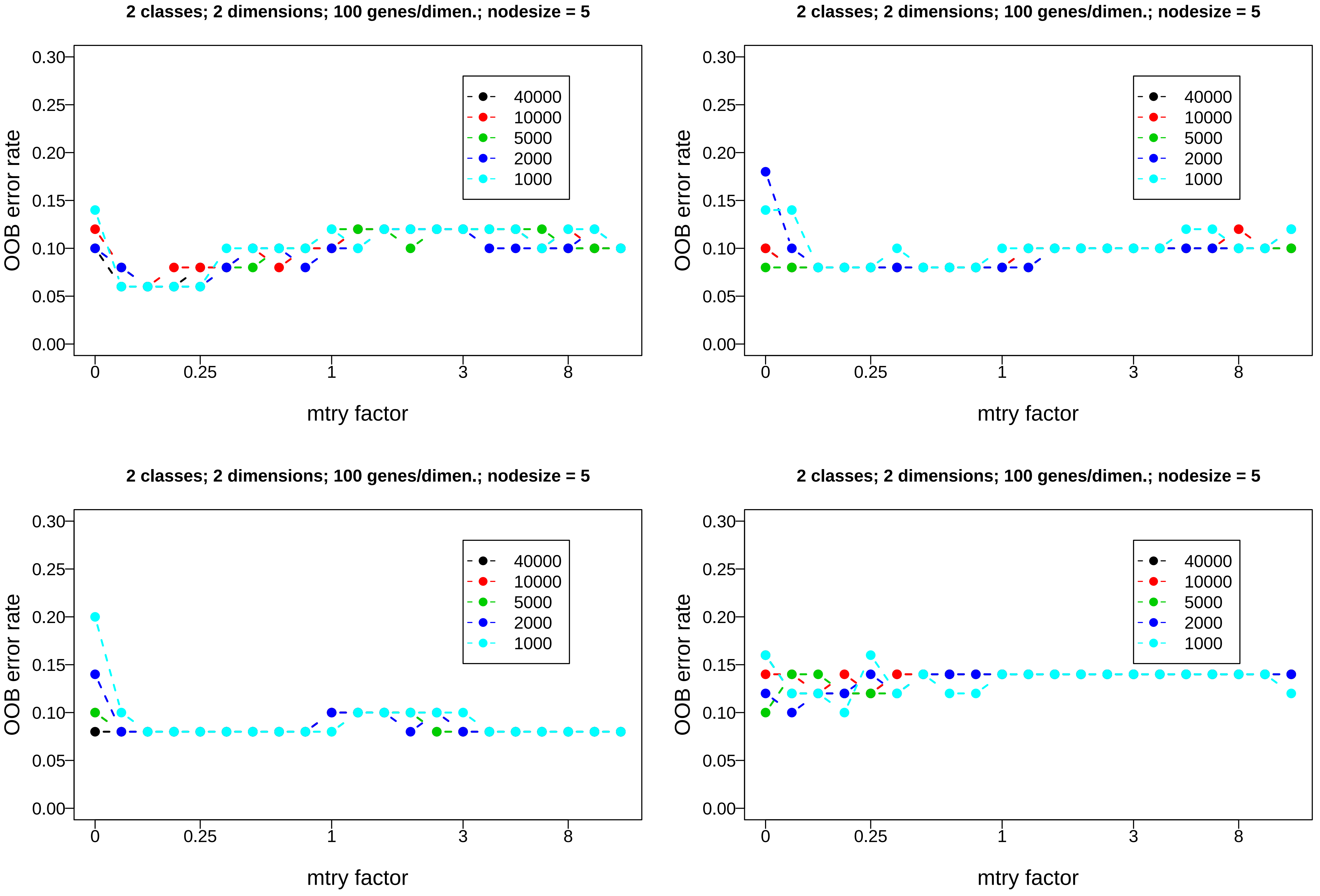

2 classes; 2 dimensions; 100 genes/dimen.; nodesize = 5
OOB error rate
mtry factor
40000
10000
5000
2000
1000
0.00
0.05
0.10
0.15
0.20
0.25
0.30
0
0.25
1
3
8

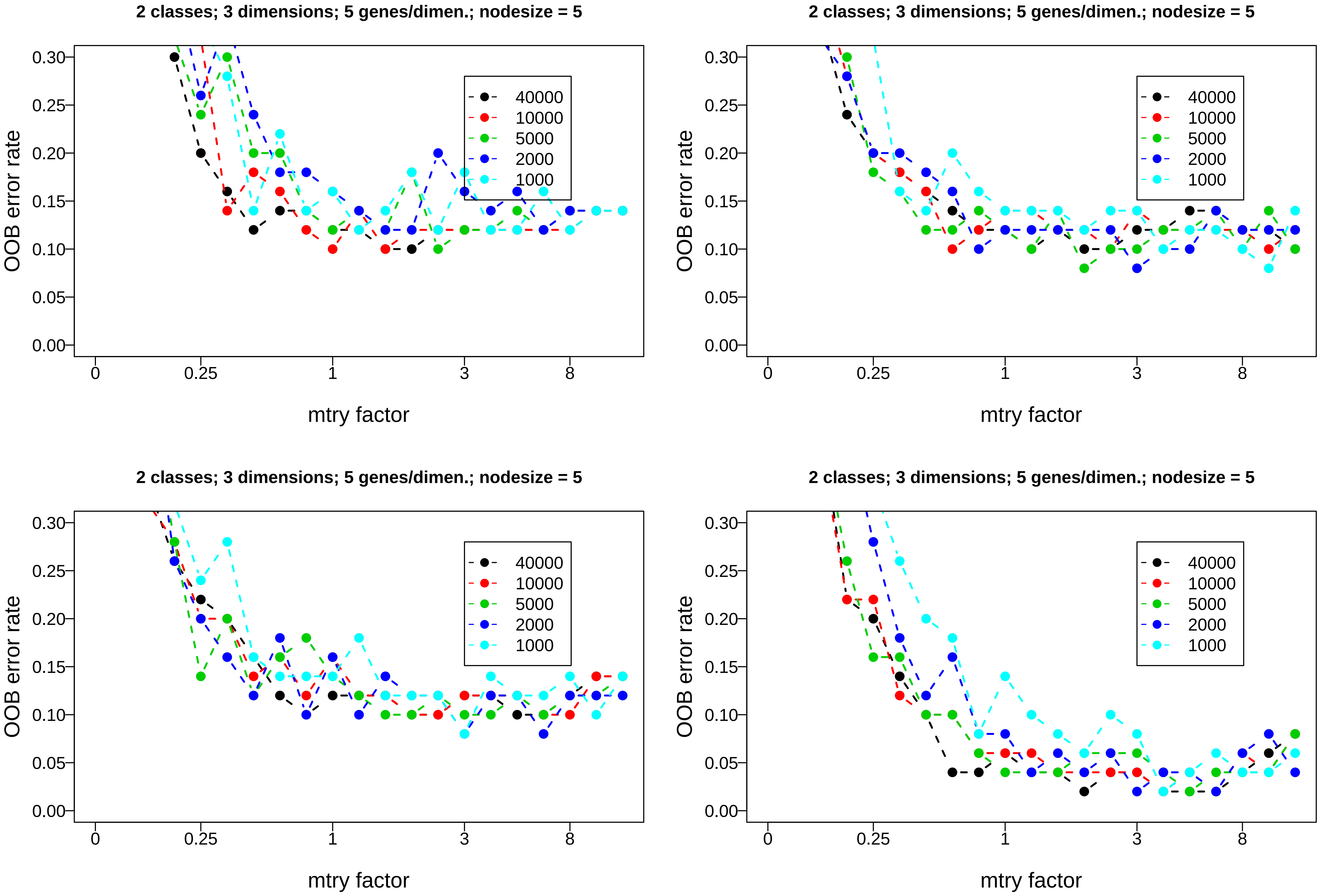
2 classes; 3 dimensions; 5 genes/dimen.; nodesize = 5
OOB error rate
mtry factor
40000
10000
5000
2000
1000
0.00
0.05
0.10
0.15
0.20
0.25
0.30
0
0.25
1
3
8
2 classes; 3 dimensions; 5 genes/dimen.; nodesize = 5
2 classes; 3 dimensions; 5 genes/dimen.; nodesize = 5
2 classes; 3 dimensions; 5 genes/dimen.; nodesize = 5

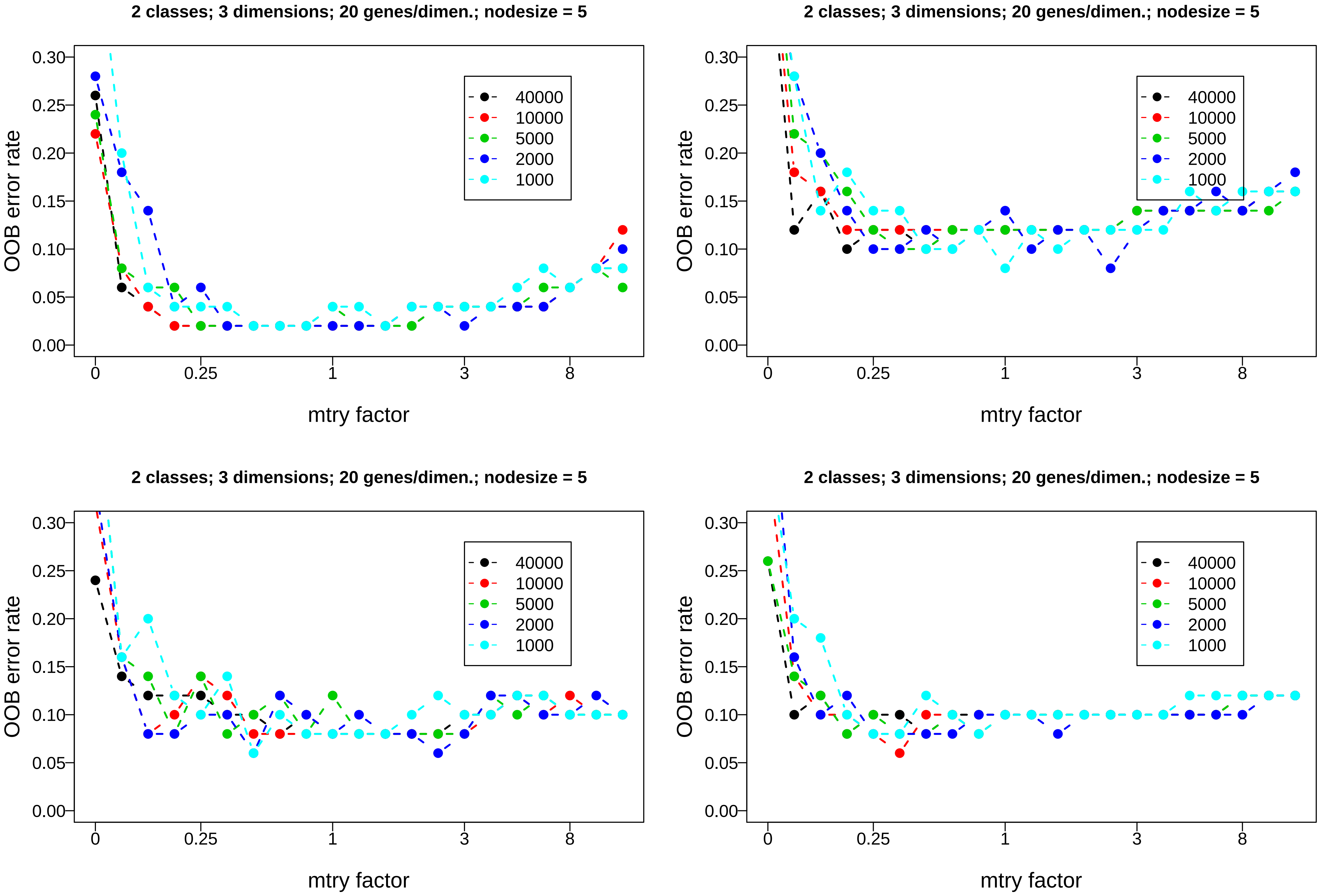
2 classes; 3 dimensions; 20 genes/dimen.; nodesize = 5
OOB error rate
0.00
0.05
0.10
0.15
0.20
0.25
0.30
mtry factor
0
0.25
1
3
8
40000
10000
5000
2000
1000
2 classes; 3 dimensions; 20 genes/dimen.; nodesize = 5
OOB error rate
mtry factor
40000
10000
5000
2000
1000
2 classes; 3 dimensions; 20 genes/dimen.; nodesize = 5
OOB error rate
mtry factor
40000
10000
5000
2000
1000
2 classes; 3 dimensions; 20 genes/dimen.; nodesize = 5
OOB error rate
mtry factor
40000
10000
5000
2000
1000

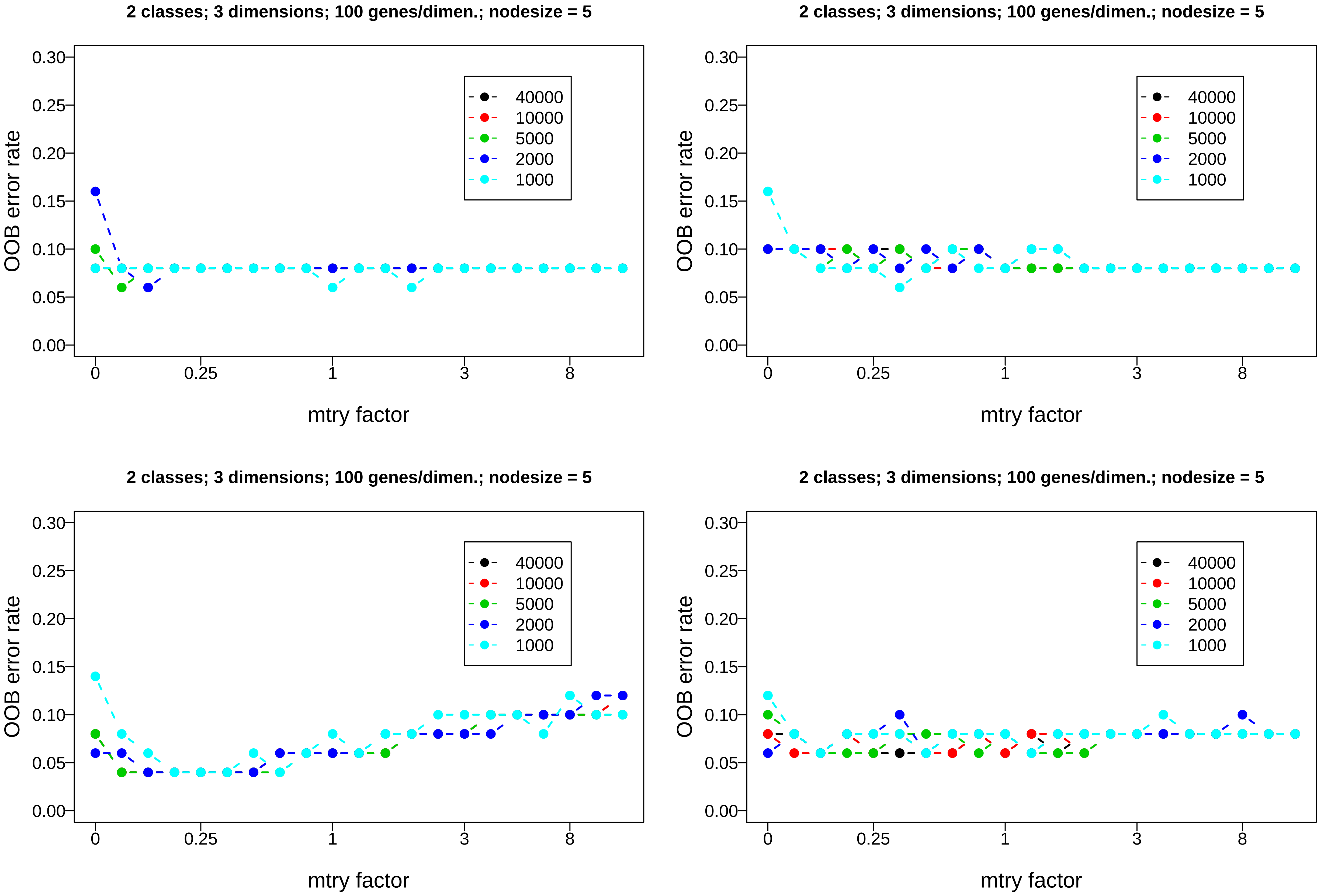
2 classes; 3 dimensions; 100 genes/dimen.; nodesize = 5
OOB error rate
0.00
0.05
0.10
0.15
0.20
0.25
0.30
mtry factor
0
0.25
1
3
8
40000
10000
5000
2000
1000
2 classes; 3 dimensions; 100 genes/dimen.; nodesize = 5
OOB error rate
mtry factor
40000
10000
5000
2000
1000
2 classes; 3 dimensions; 100 genes/dimen.; nodesize = 5
OOB error rate
mtry factor
40000
10000
5000
2000
1000
2 classes; 3 dimensions; 100 genes/dimen.; nodesize = 5
OOB error rate
mtry factor
40000
10000
5000
2000
1000

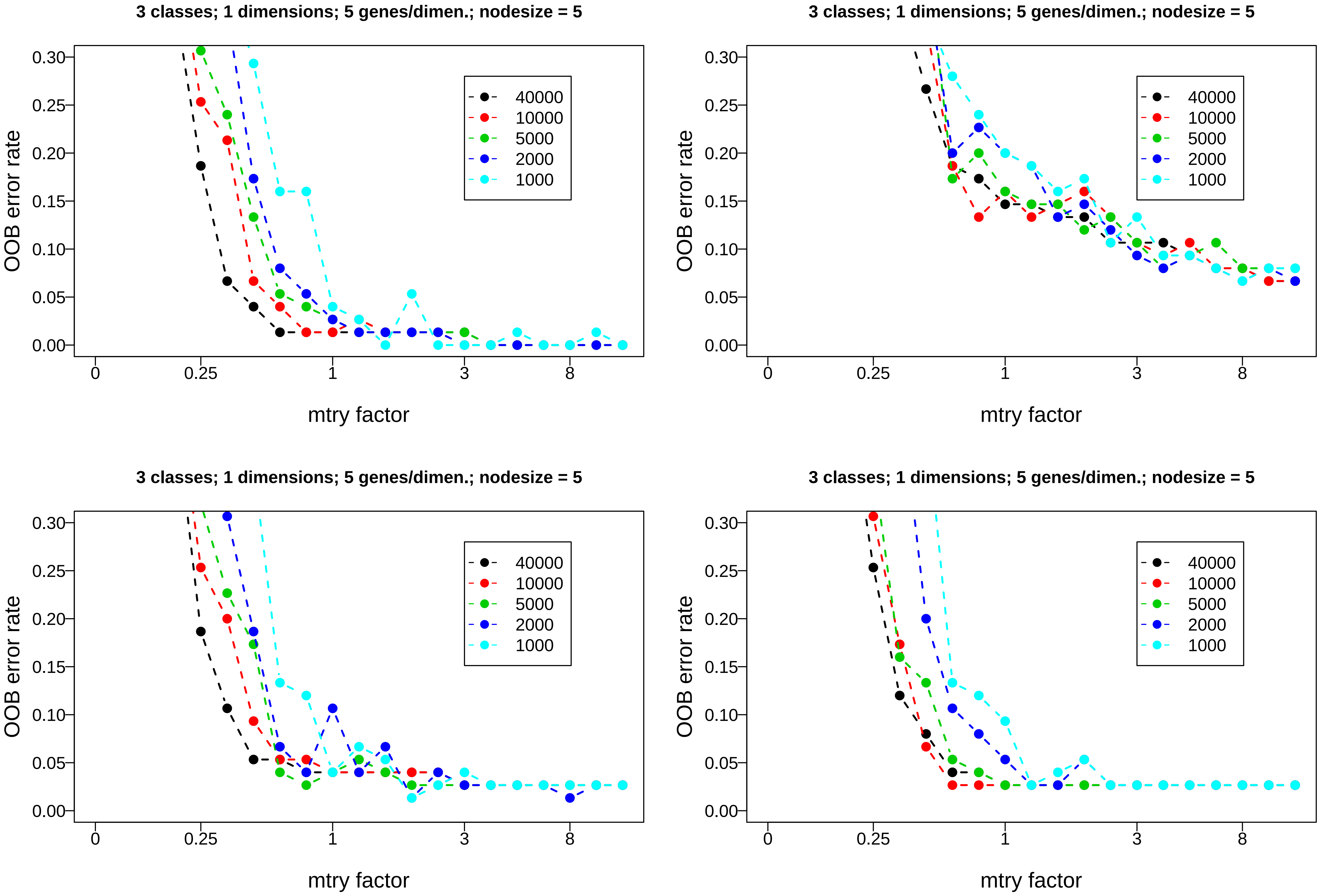
3 classes; 1 dimensions; 5 genes/dimen.; nodesize = 5
OOB error rate
0.00
0.05
0.10
0.15
0.20
0.25
0.30
mtry factor
0
0.25
1
3
8
40000
10000
5000
2000
1000
3 classes; 1 dimensions; 5 genes/dimen.; nodesize = 5
OOB error rate
mtry factor
40000
10000
5000
2000
1000
3 classes; 1 dimensions; 5 genes/dimen.; nodesize = 5
OOB error rate
mtry factor
40000
10000
5000
2000
1000
3 classes; 1 dimensions; 5 genes/dimen.; nodesize = 5
OOB error rate
mtry factor
40000
10000
5000
2000
1000

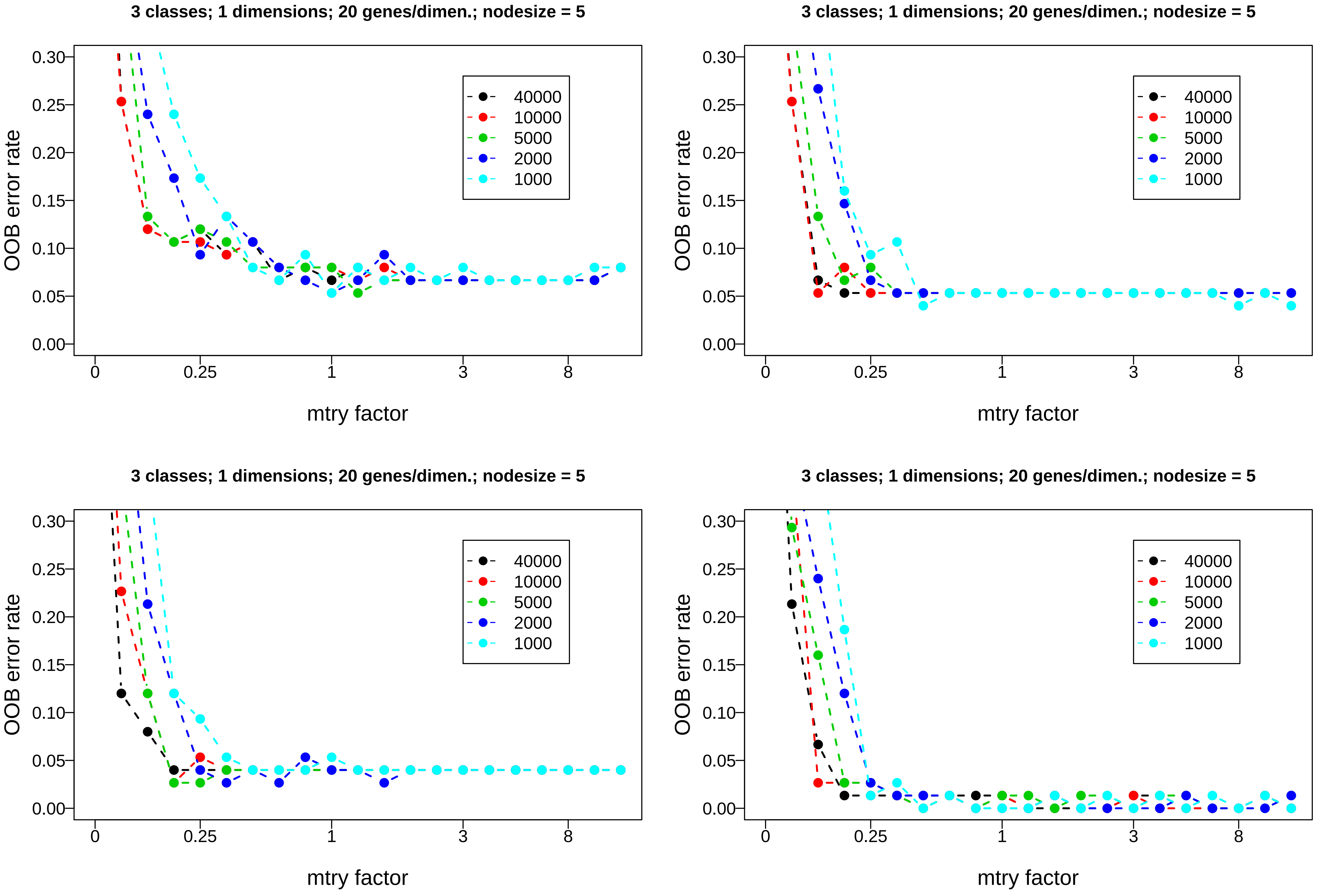
3 classes; 1 dimensions; 20 genes/dimen.; nodesize = 5
OOB error rate
0.00
0.05
0.10
0.15
0.20
0.25
0.30
mtry factor
0
0.25
1
3
8
40000
10000
5000
2000
1000
3 classes; 1 dimensions; 20 genes/dimen.; nodesize = 5
OOB error rate
mtry factor
40000
10000
5000
2000
1000
3 classes; 1 dimensions; 20 genes/dimen.; nodesize = 5
OOB error rate
mtry factor
40000
10000
5000
2000
1000
3 classes; 1 dimensions; 20 genes/dimen.; nodesize = 5
OOB error rate
mtry factor
40000
10000
5000
2000
1000

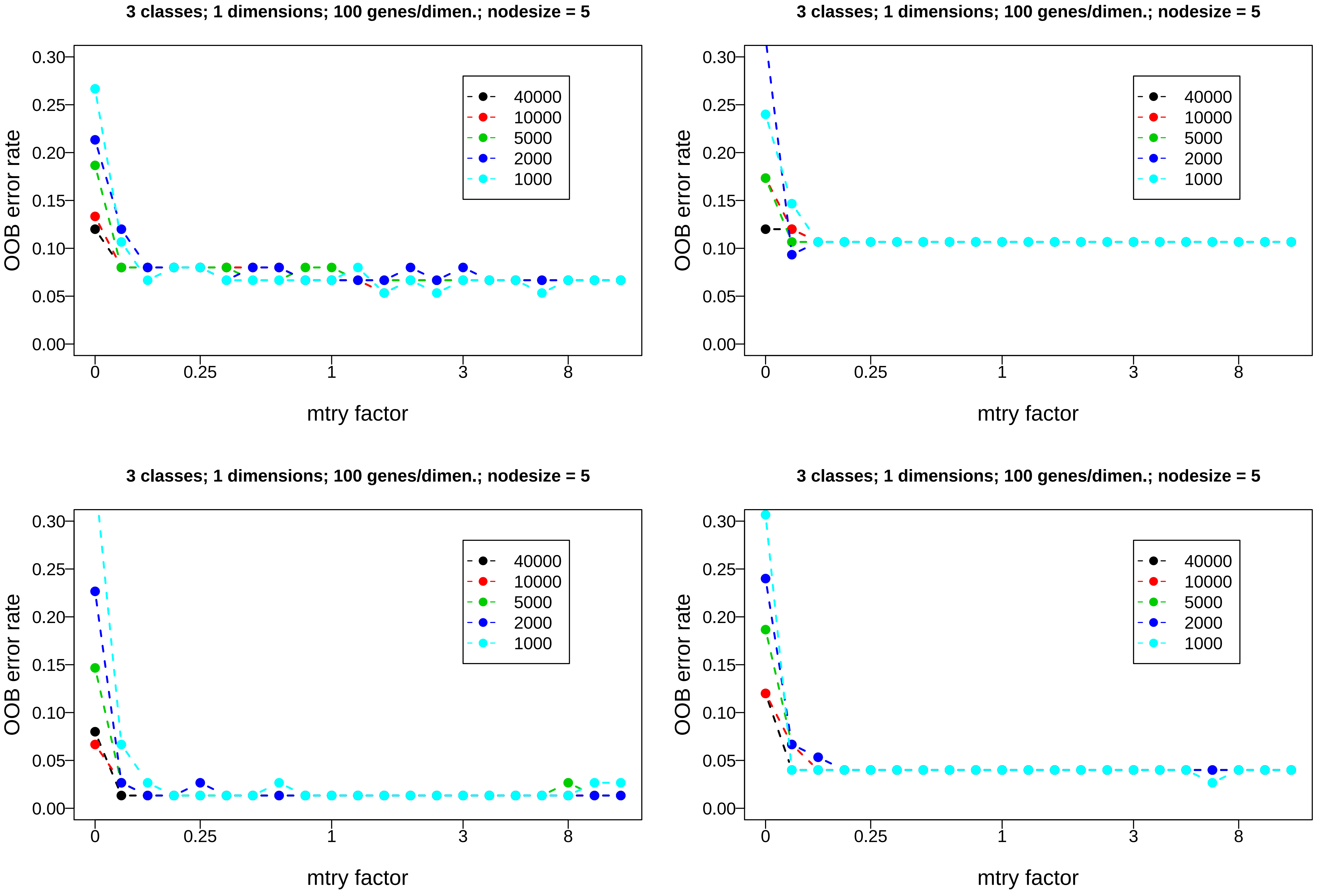
3 classes; 1 dimensions; 100 genes/dimen.; nodesize = 5
OOB error rate
mtry factor
0.00
0.05
0.10
0.15
0.20
0.25
0.30
0
0.25
1
3
8
40000
10000
5000
2000
1000

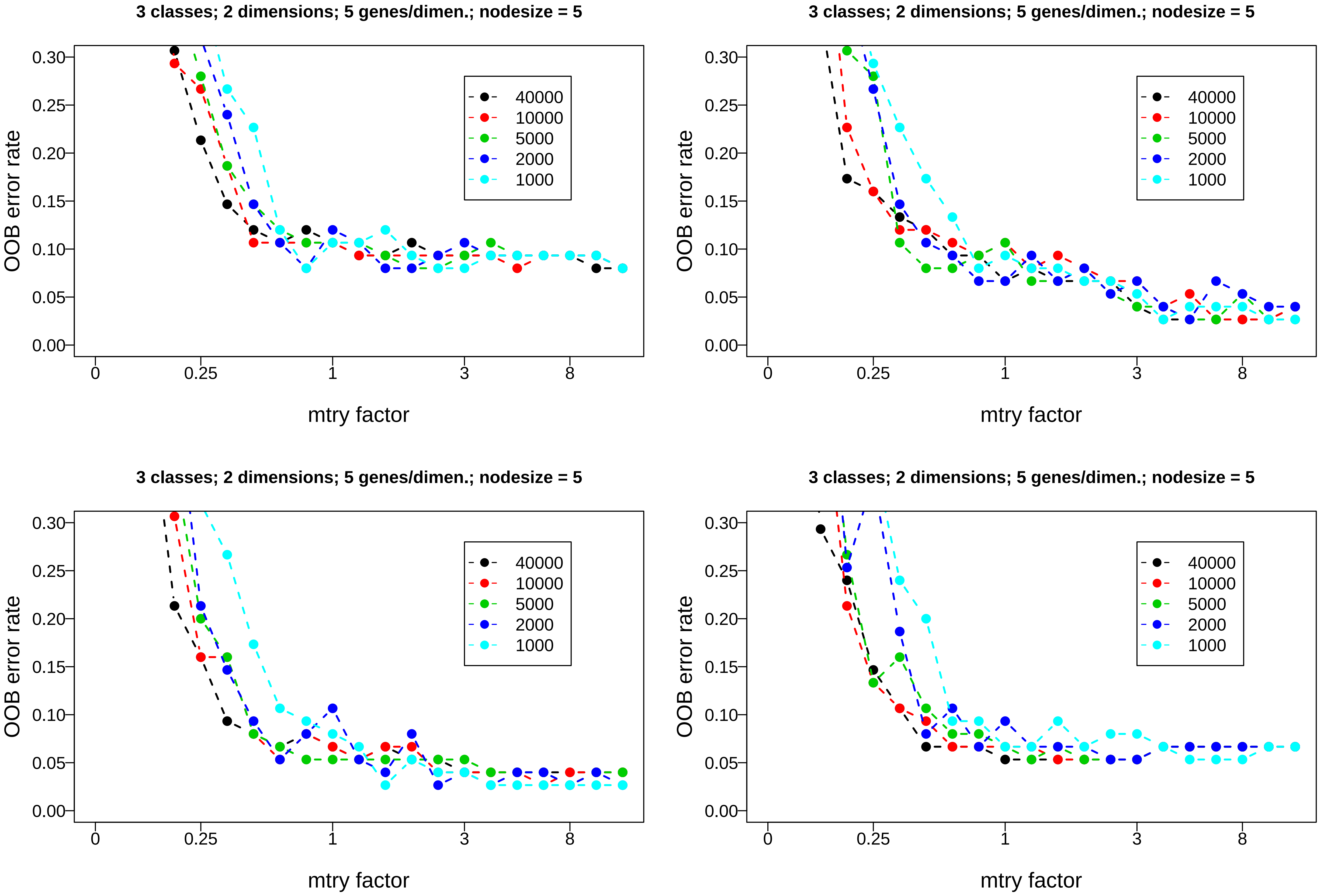

3 classes; 2 dimensions; 5 genes/dimen.; nodesize = 5
OOB error rate
mtry factor
0.00
0.05
0.10
0.15
0.20
0.25
0.30
0
0.25
1
3
8
40000
10000
5000
2000
1000

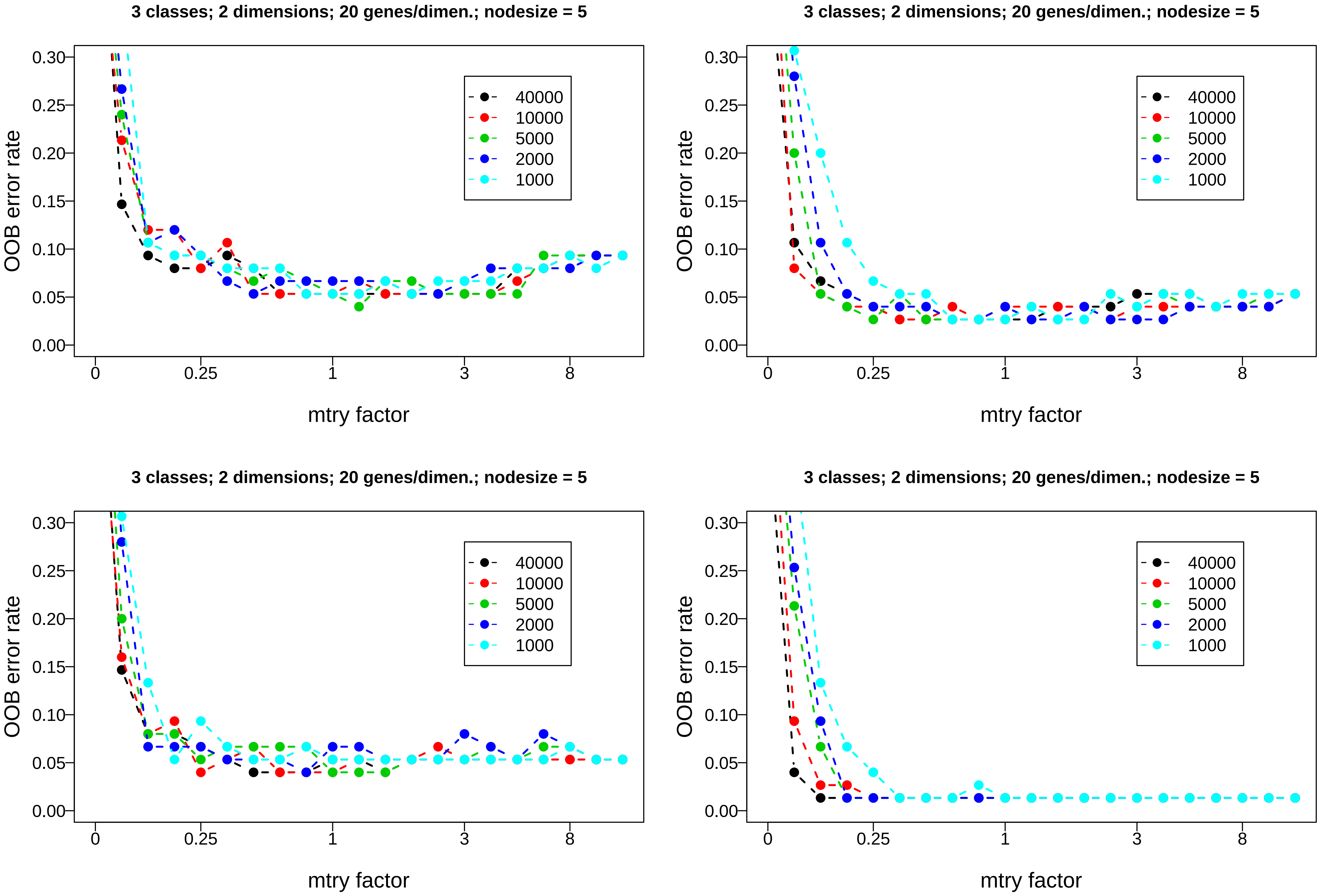
3 classes; 2 dimensions; 20 genes/dimen.; nodesize = 5
OOB error rate
mtry factor
40000
10000
5000
2000
1000
0.30
0.25
0.20
0.15
0.10
0.05
0.00
0
0.25
1
3
8

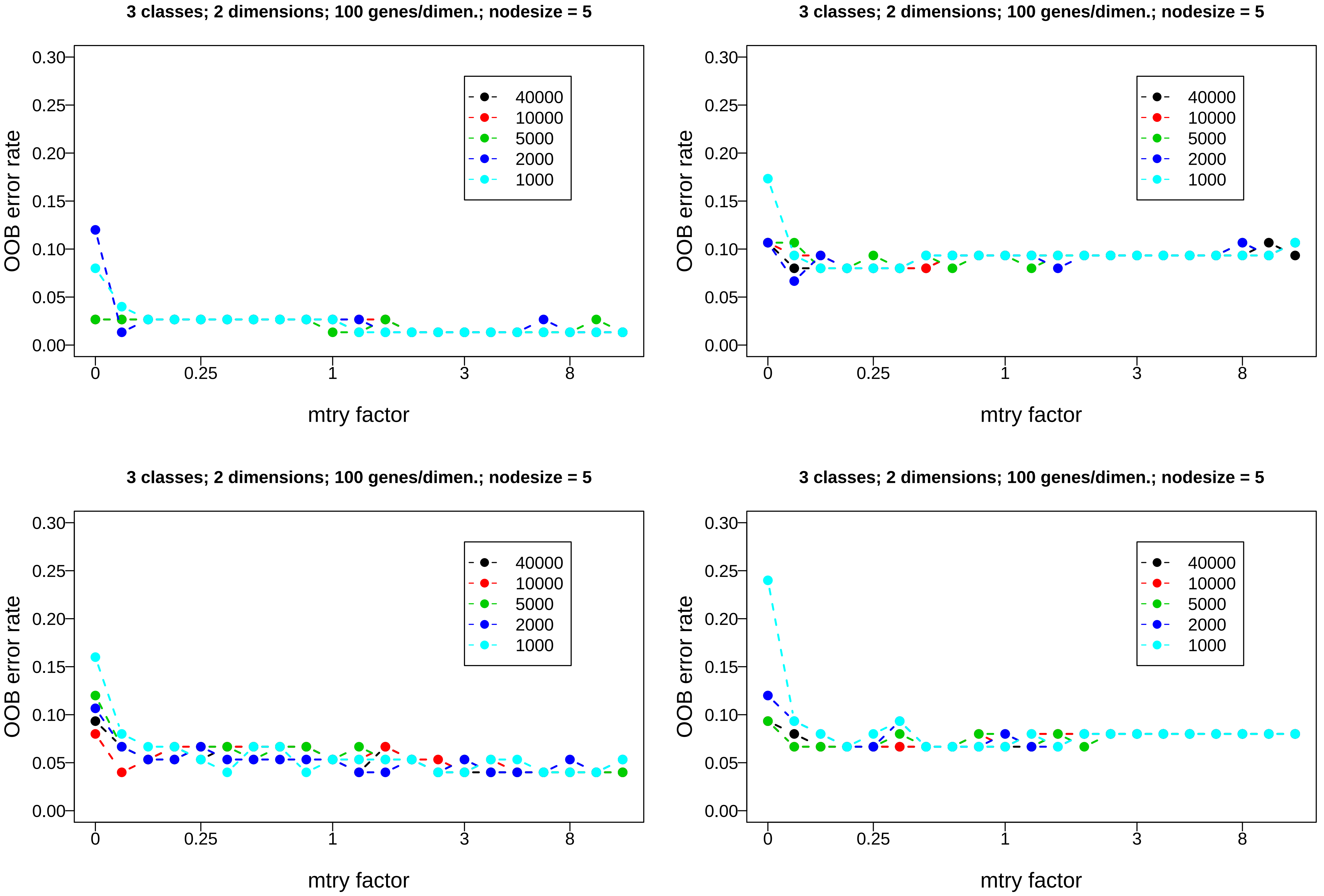

3 classes; 2 dimensions; 100 genes/dimen.; nodesize = 5
OOB error rate
mtry factor
40000
10000
5000
2000
1000
0.00
0.05
0.10
0.15
0.20
0.25
0.30
0
0.25
1
3
8

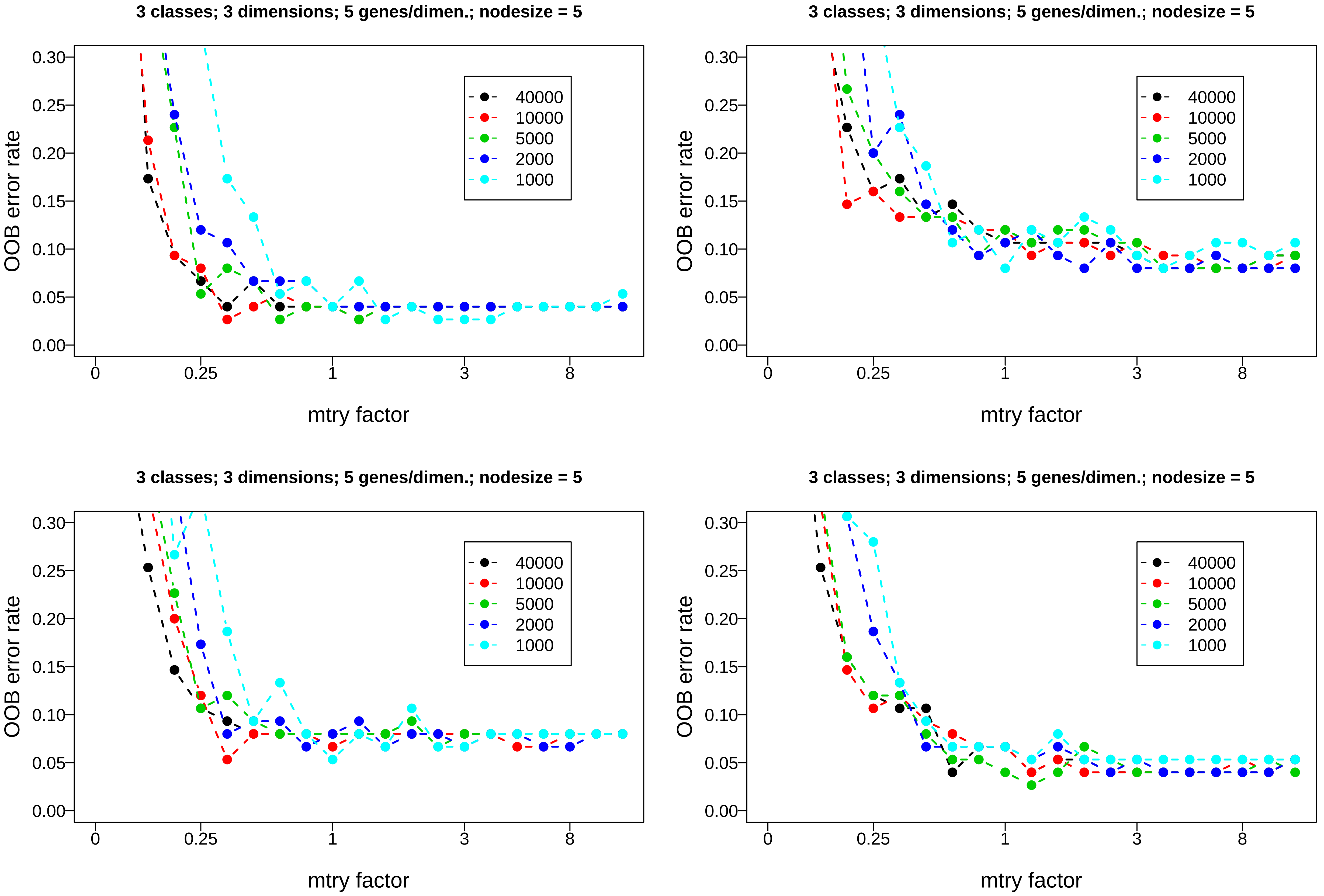
3 classes; 3 dimensions; 5 genes/dimen.; nodesize = 5
OOB error rate
mtry factor
0.00
0.05
0.10
0.15
0.20
0.25
0.30
0
0.25
1
3
8
40000
10000
5000
2000
1000

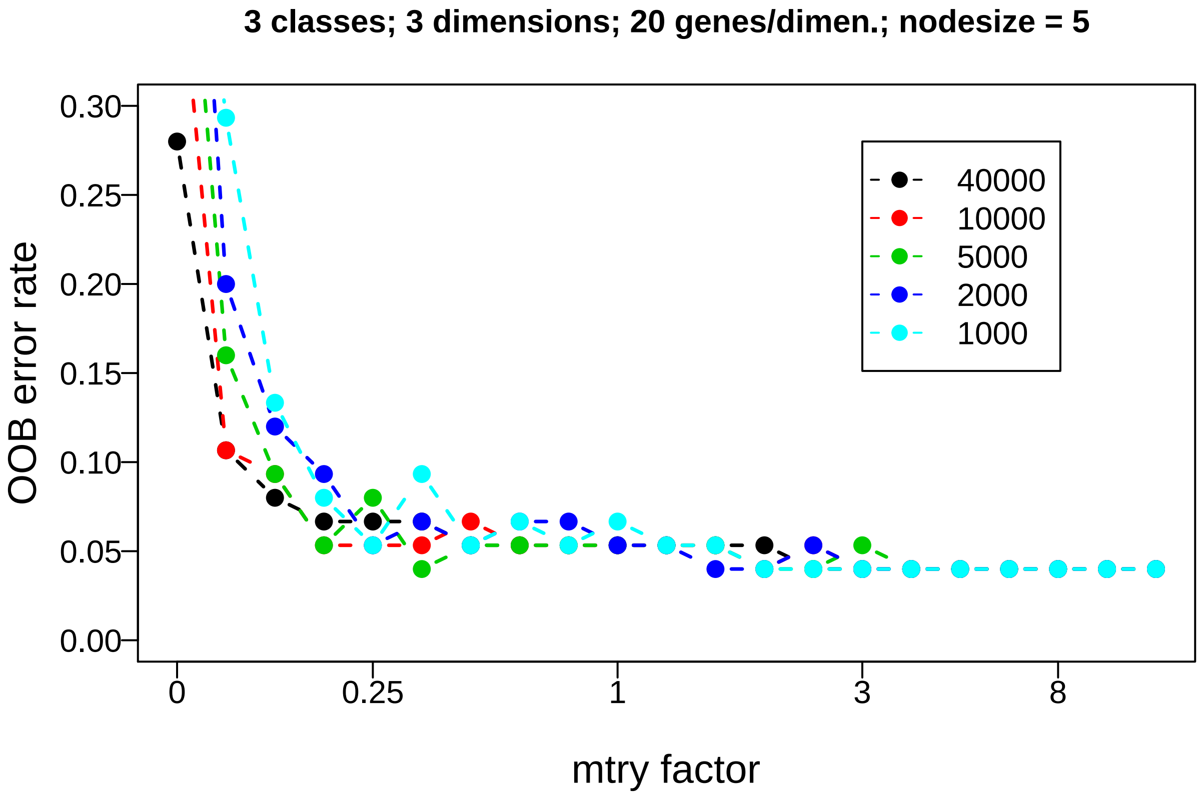
3 classes; 3 dimensions; 20 genes/dimen.; nodesize = 5
OOB error rate
0.00
0.05
0.10
0.15
0.20
0.25
0.30
0
0.25
1
3
8
mtry factor
40000
10000
5000
2000
1000

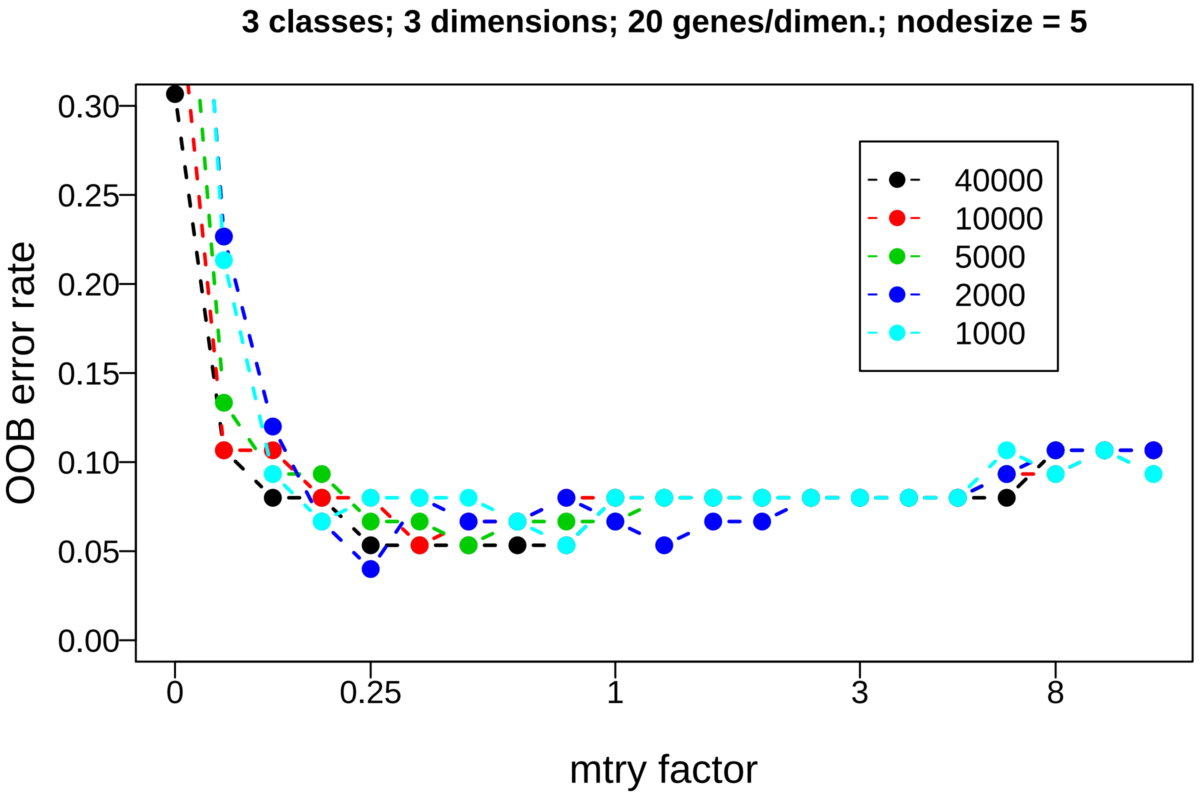
3 classes; 3 dimensions; 20 genes/dimen.; nodesize = 5
OOB error rate
0.00
0.05
0.10
0.15
0.20
0.25
0.30
0
0.25
1
3
8
mtry factor
40000
10000
5000
2000
1000

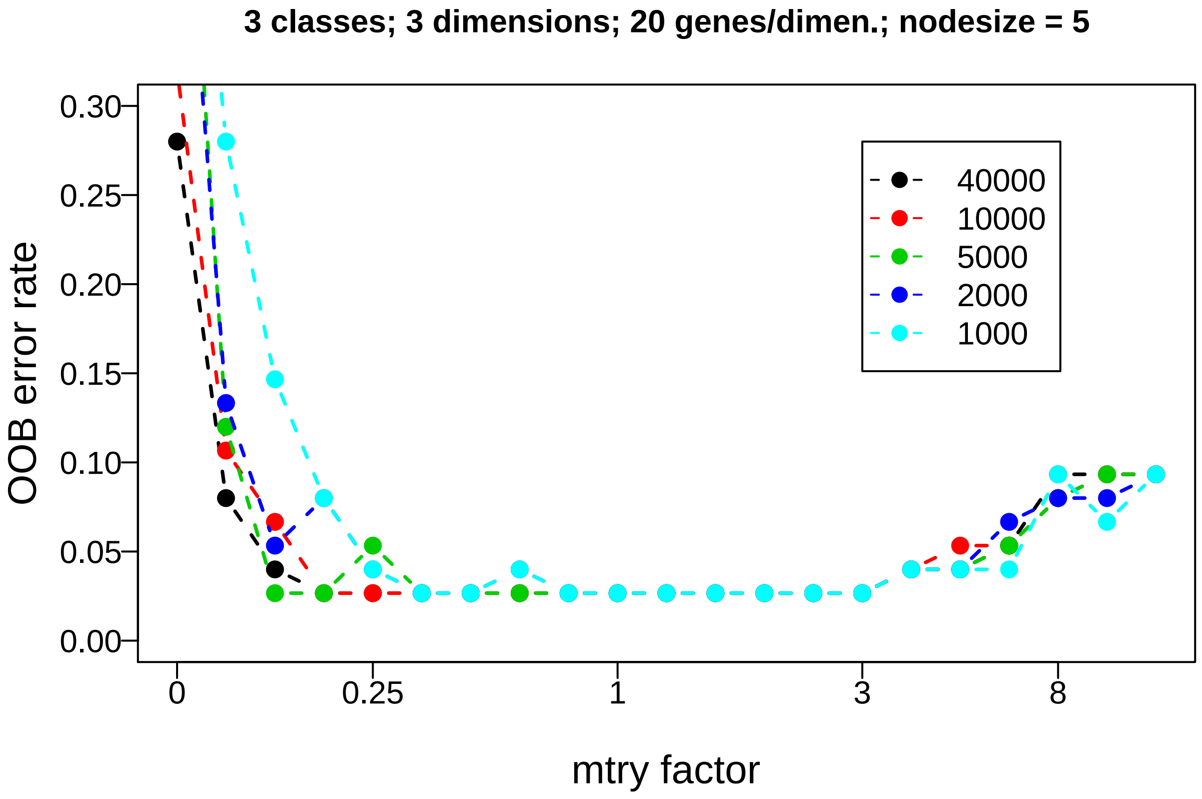
3 classes; 3 dimensions; 20 genes/dimen.; nodesize = 5
OOB error rate
0.00
0.05
0.10
0.15
0.20
0.25
0.30
0
0.25
1
3
8
mtry factor
40000
10000
5000
2000
1000

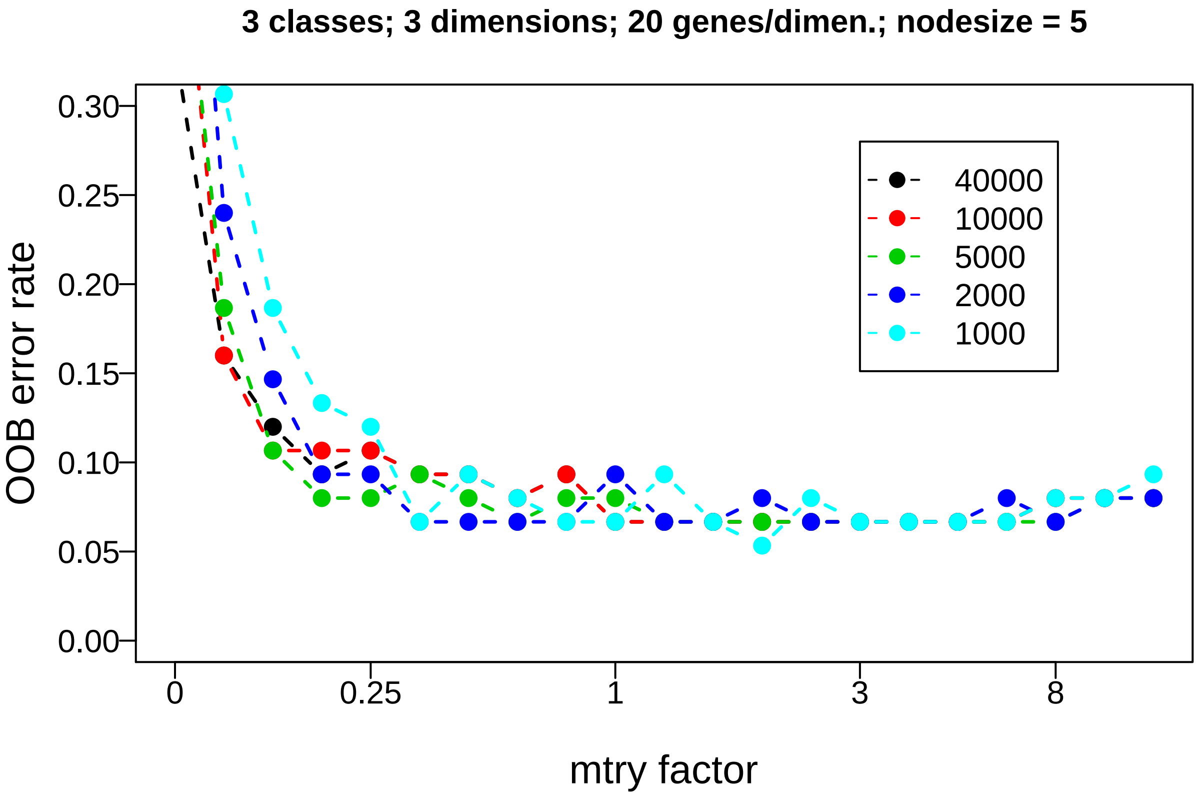
3 classes; 3 dimensions; 20 genes/dimen.; nodesize = 5
OOB error rate
0.00
0.05
0.10
0.15
0.20
0.25
0.30
0
0.25
1
3
8
mtry factor
40000
10000
5000
2000
1000

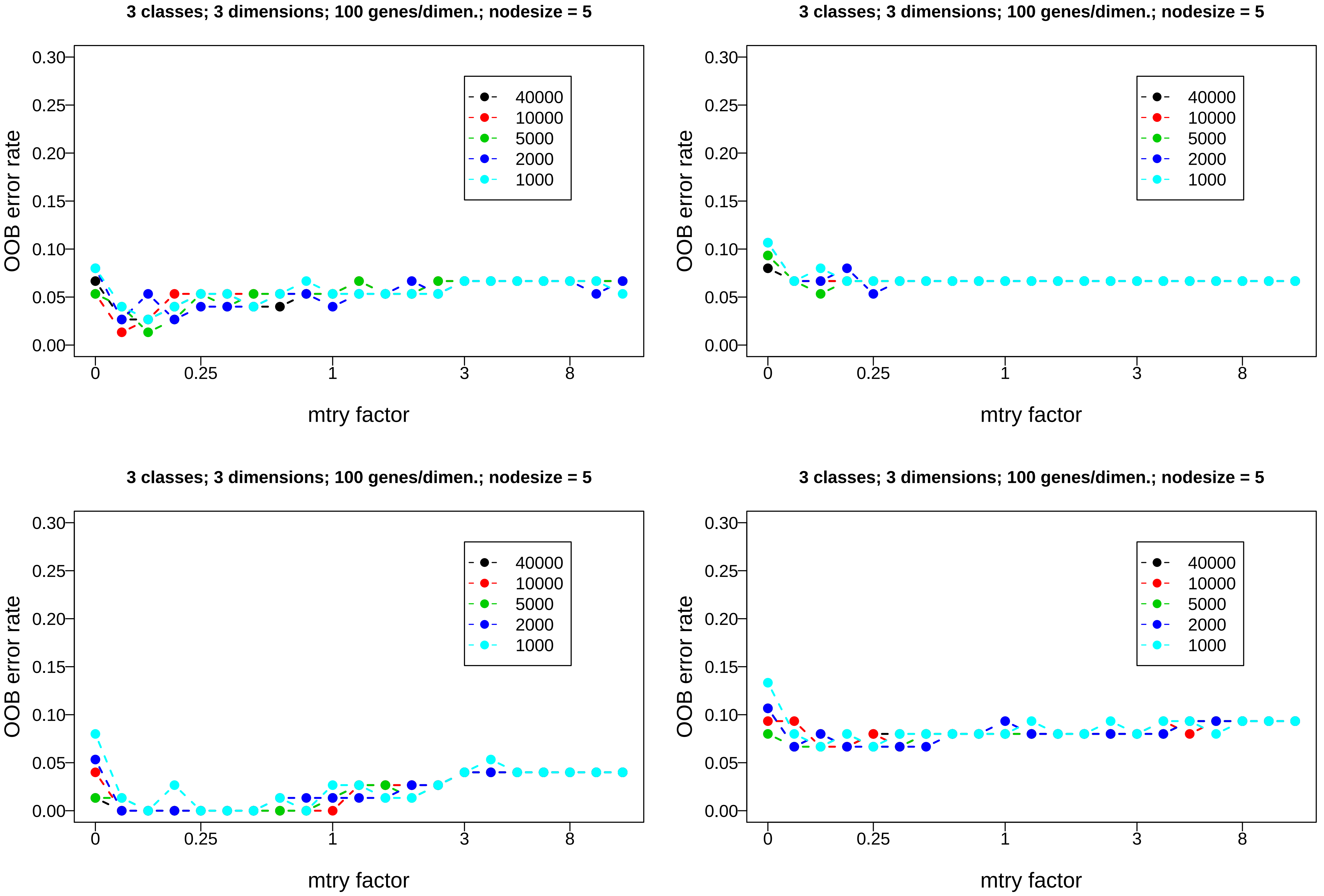
3 classes; 3 dimensions; 100 genes/dimen.; nodesize = 5
OOB error rate
0.00
0.05
0.10
0.15
0.20
0.25
0.30
mtry factor
0
0.25
1
3
8
40000
10000
5000
2000
1000
3 classes; 3 dimensions; 100 genes/dimen.; nodesize = 5
OOB error rate
mtry factor
3 classes; 3 dimensions; 100 genes/dimen.; nodesize = 5
OOB error rate
mtry factor
3 classes; 3 dimensions; 100 genes/dimen.; nodesize = 5
OOB error rate
mtry factor

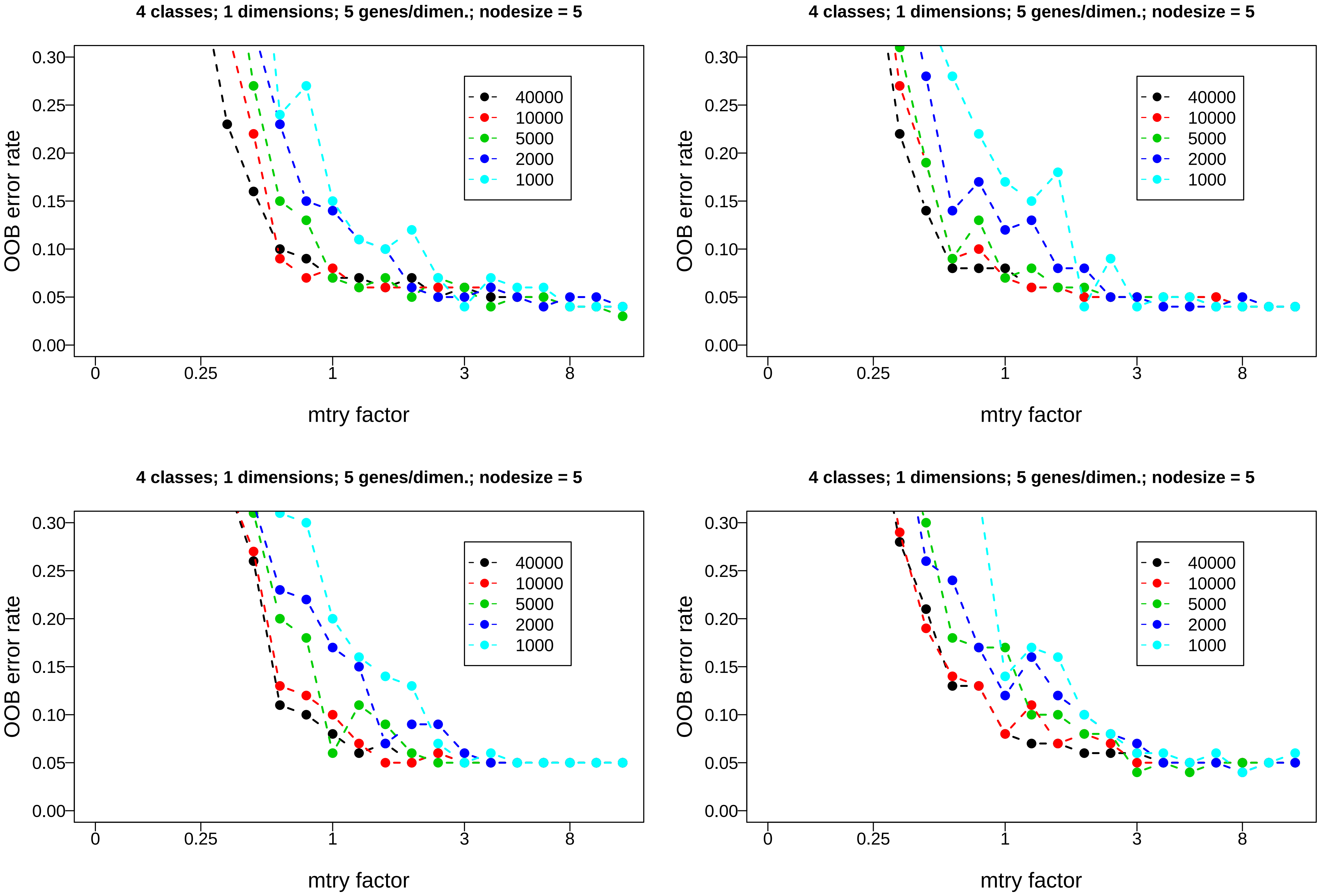
4 classes; 1 dimensions; 5 genes/dimen.; nodesize = 5
OOB error rate
0.00
0.05
0.10
0.15
0.20
0.25
0.30
0
0.25
1
3
8
mtry factor
40000
10000
5000
2000
1000
4 classes; 1 dimensions; 5 genes/dimen.; nodesize = 5
OOB error rate
mtry factor
40000
10000
5000
2000
1000
4 classes; 1 dimensions; 5 genes/dimen.; nodesize = 5
OOB error rate
mtry factor
40000
10000
5000
2000
1000
4 classes; 1 dimensions; 5 genes/dimen.; nodesize = 5
OOB error rate
mtry factor
40000
10000
5000
2000
1000

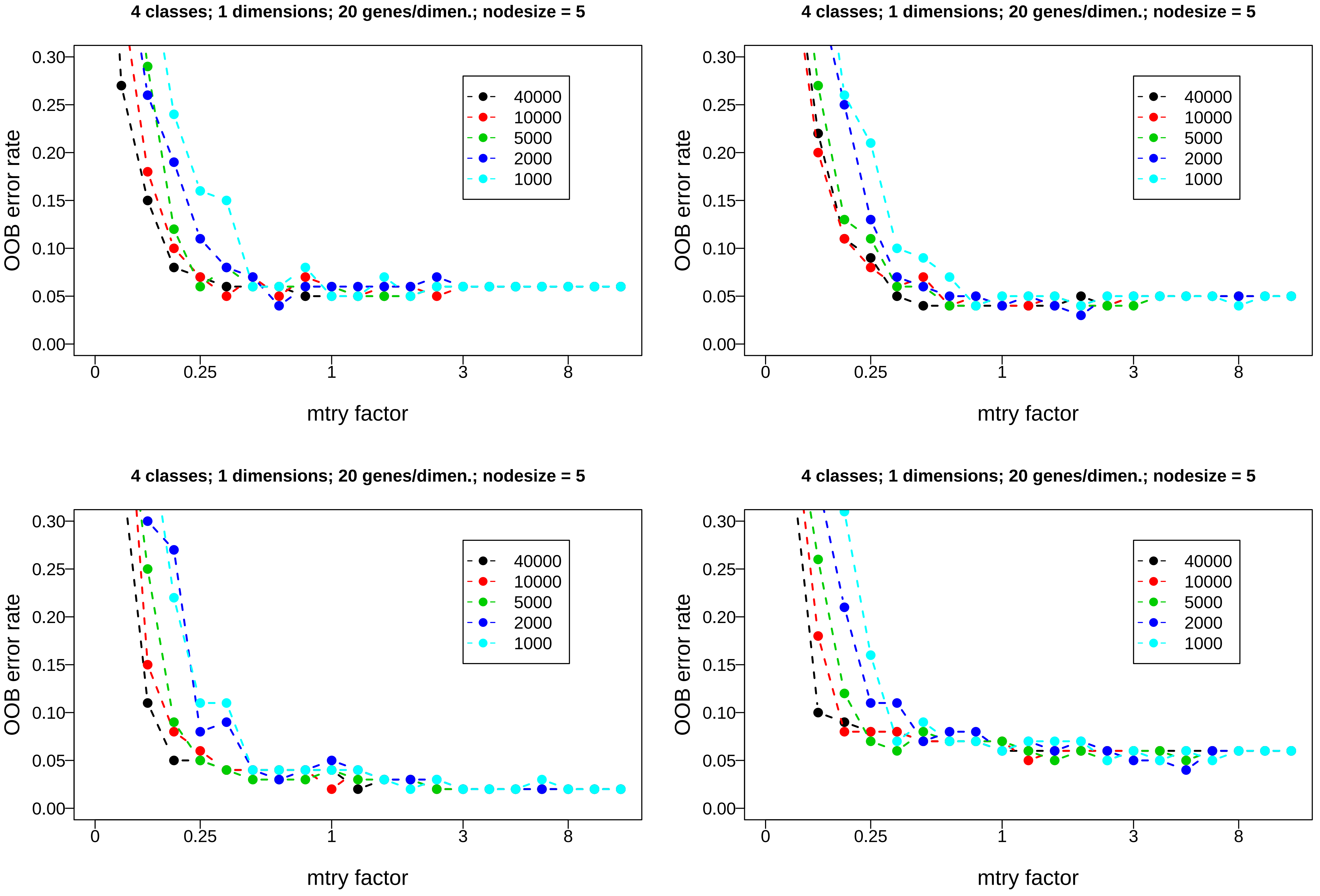
4 classes; 1 dimensions; 20 genes/dimen.; nodesize = 5
OOB error rate
0.00
0.05
0.10
0.15
0.20
0.25
0.30
mtry factor
0
0.25
1
3
8
40000
10000
5000
2000
1000
4 classes; 1 dimensions; 20 genes/dimen.; nodesize = 5
OOB error rate
0.00
0.05
0.10
0.15
0.20
0.25
0.30
mtry factor
0
0.25
1
3
8
40000
10000
5000
2000
1000
4 classes; 1 dimensions; 20 genes/dimen.; nodesize = 5
OOB error rate
0.00
0.05
0.10
0.15
0.20
0.25
0.30
mtry factor
0
0.25
1
3
8
40000
10000
5000
2000
1000
4 classes; 1 dimensions; 20 genes/dimen.; nodesize = 5
OOB error rate
0.00
0.05
0.10
0.15
0.20
0.25
0.30
mtry factor
0
0.25
1
3
8
40000
10000
5000
2000
1000

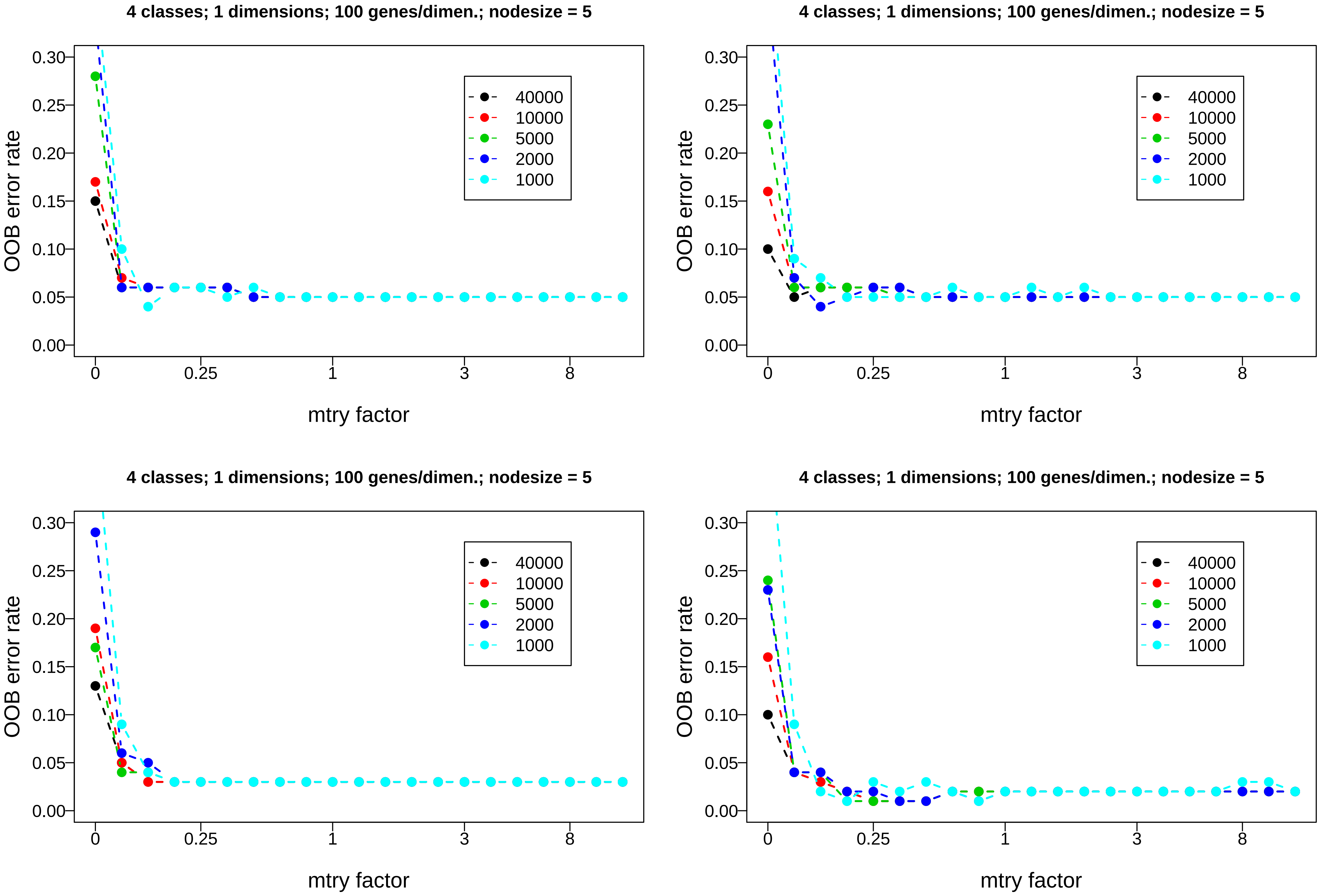

4 classes; 1 dimensions; 100 genes/dimen.; nodesize = 5
OOB error rate
mtry factor
40000
10000
5000
2000
1000
0.00
0.05
0.10
0.15
0.20
0.25
0.30
0
0.25
1
3
8

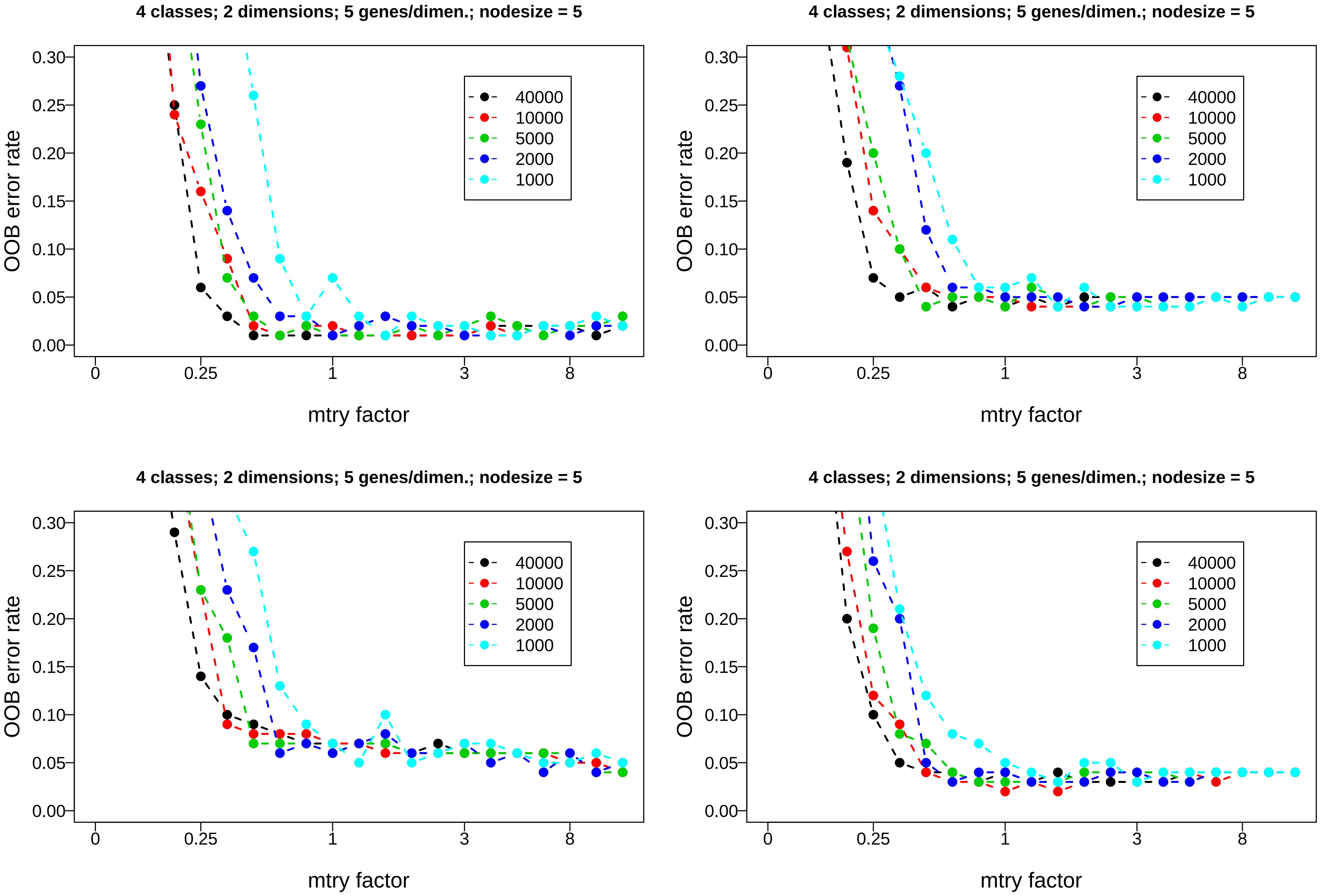
4 classes; 2 dimensions; 5 genes/dimen.; nodesize = 5
OOB error rate
0.30
0.25
0.20
0.15
0.10
0.05
0.00
0
0.25
1
3
8
mtry factor
40000
10000
5000
2000
1000
4 classes; 2 dimensions; 5 genes/dimen.; nodesize = 5
OOB error rate
mtry factor
40000
10000
5000
2000
1000
4 classes; 2 dimensions; 5 genes/dimen.; nodesize = 5
OOB error rate
mtry factor
40000
10000
5000
2000
1000
4 classes; 2 dimensions; 5 genes/dimen.; nodesize = 5
OOB error rate
mtry factor
40000
10000
5000
2000
1000

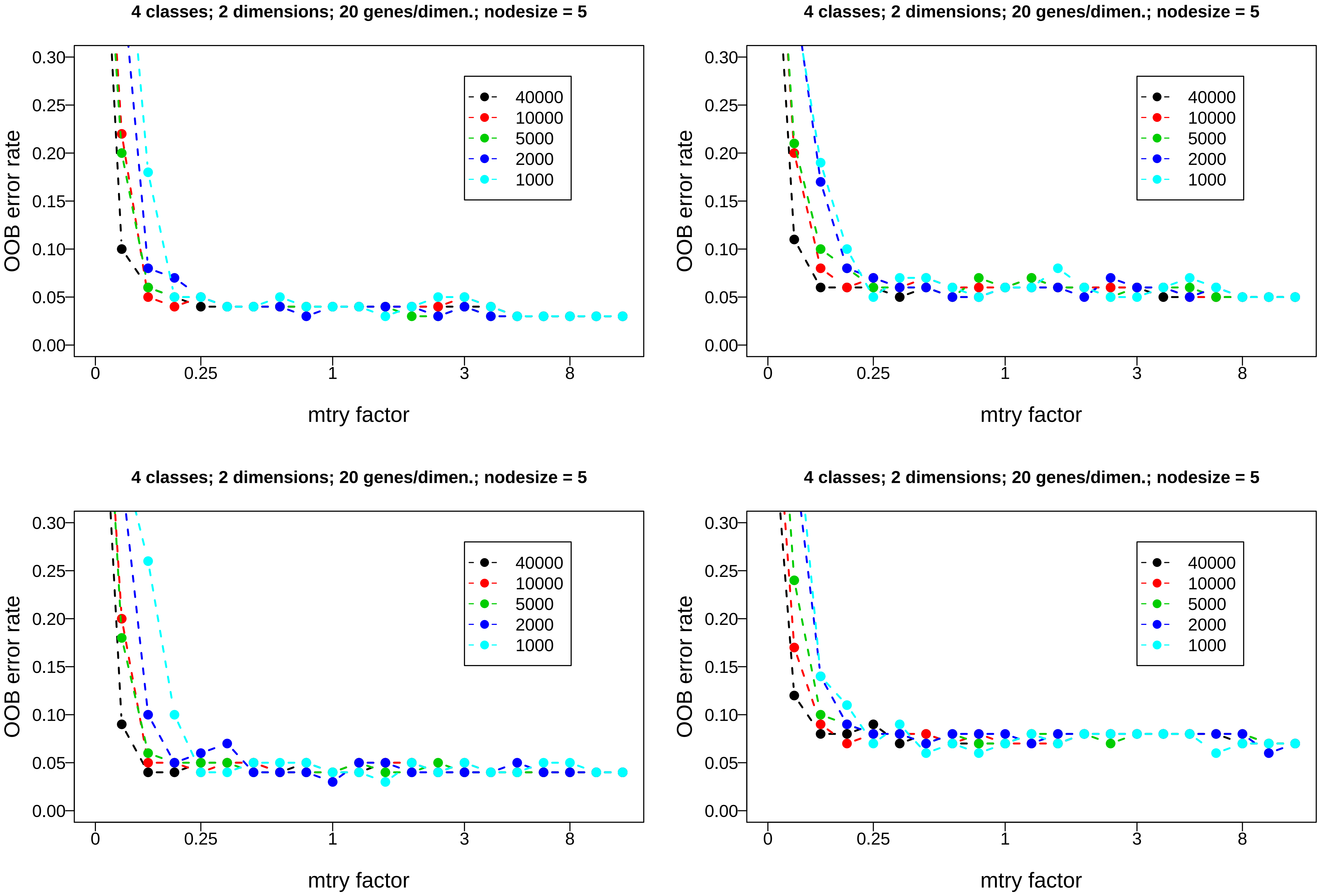

4 classes; 2 dimensions; 20 genes/dimen.; nodesize = 5
OOB error rate
mtry factor
0.00
0.05
0.10
0.15
0.20
0.25
0.30
0
0.25
1
3
8
40000
10000
5000
2000
1000

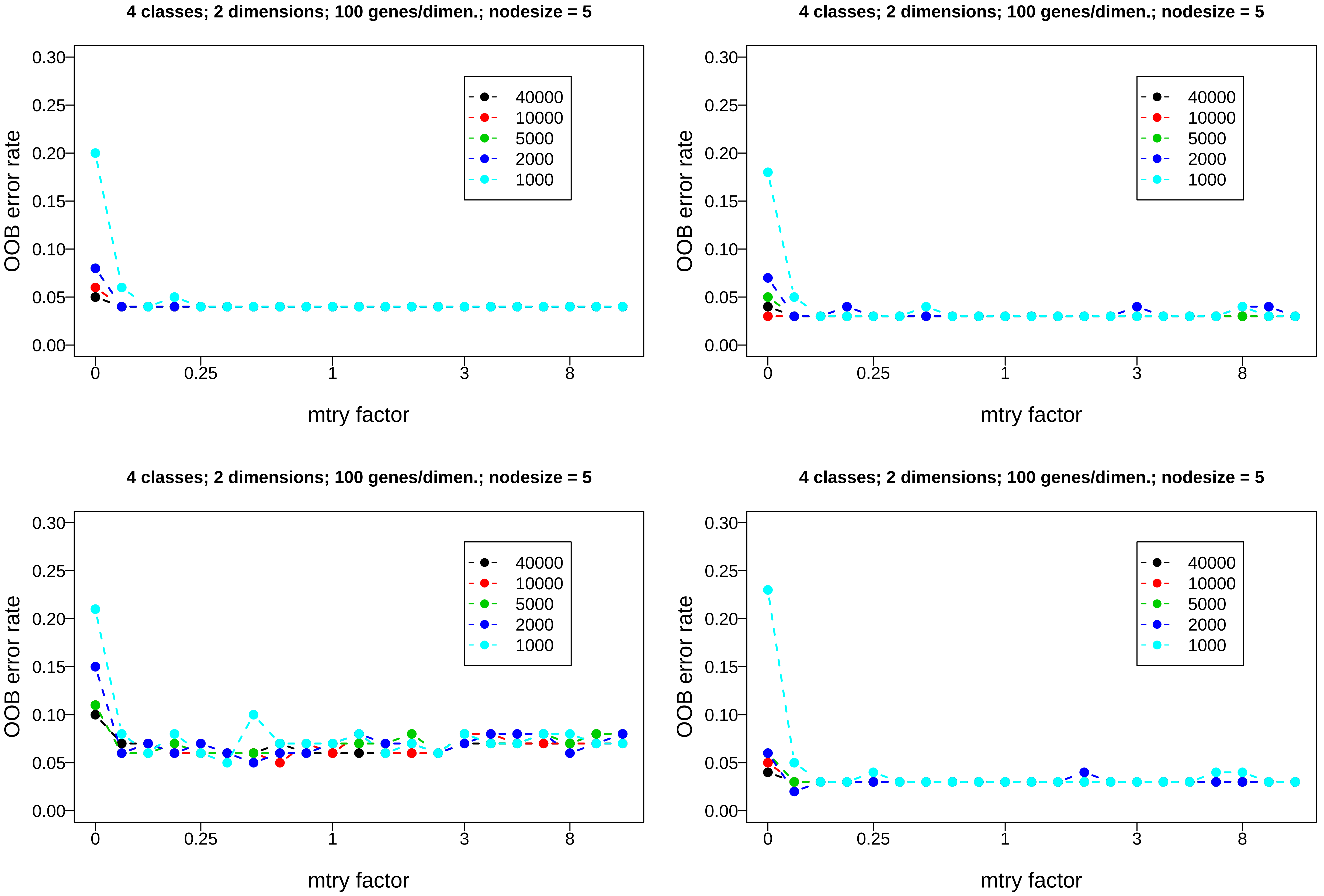
4 classes; 2 dimensions; 100 genes/dimen.; nodesize = 5
OOB error rate
mtry factor
0.00
0.05
0.10
0.15
0.20
0.25
0.30
0
0.25
1
3
8
40000
10000
5000
2000
1000
4 classes; 2 dimensions; 100 genes/dimen.; nodesize = 5
OOB error rate
mtry factor
4 classes; 2 dimensions; 100 genes/dimen.; nodesize = 5
OOB error rate
mtry factor
4 classes; 2 dimensions; 100 genes/dimen.; nodesize = 5
OOB error rate
mtry factor

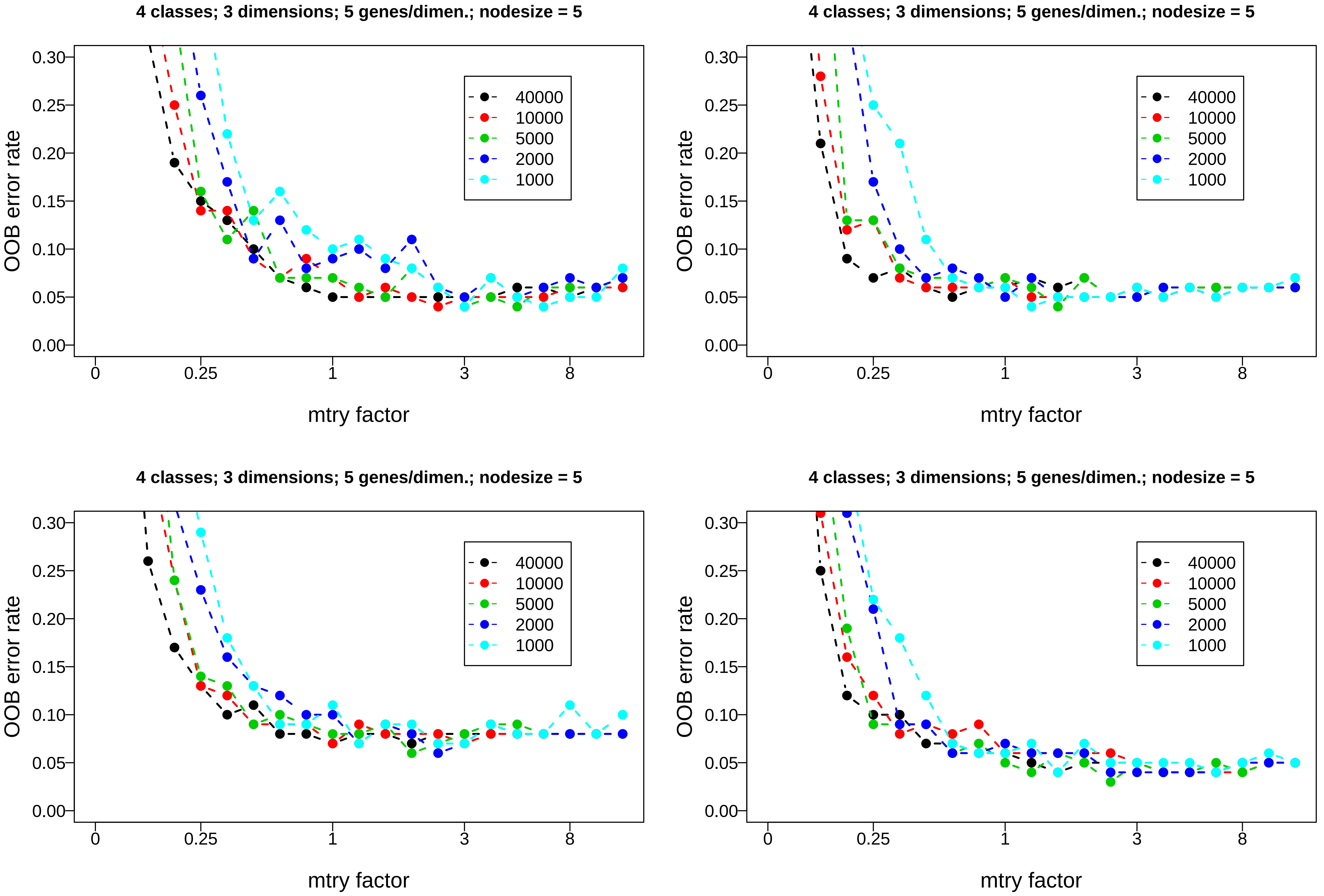
4 classes; 3 dimensions; 5 genes/dimen.; nodesize = 5
OOB error rate
mtry factor
40000
10000
5000
2000
1000
4 classes; 3 dimensions; 5 genes/dimen.; nodesize = 5
OOB error rate
mtry factor
4 classes; 3 dimensions; 5 genes/dimen.; nodesize = 5
OOB error rate
mtry factor
4 classes; 3 dimensions; 5 genes/dimen.; nodesize = 5
OOB error rate
mtry factor

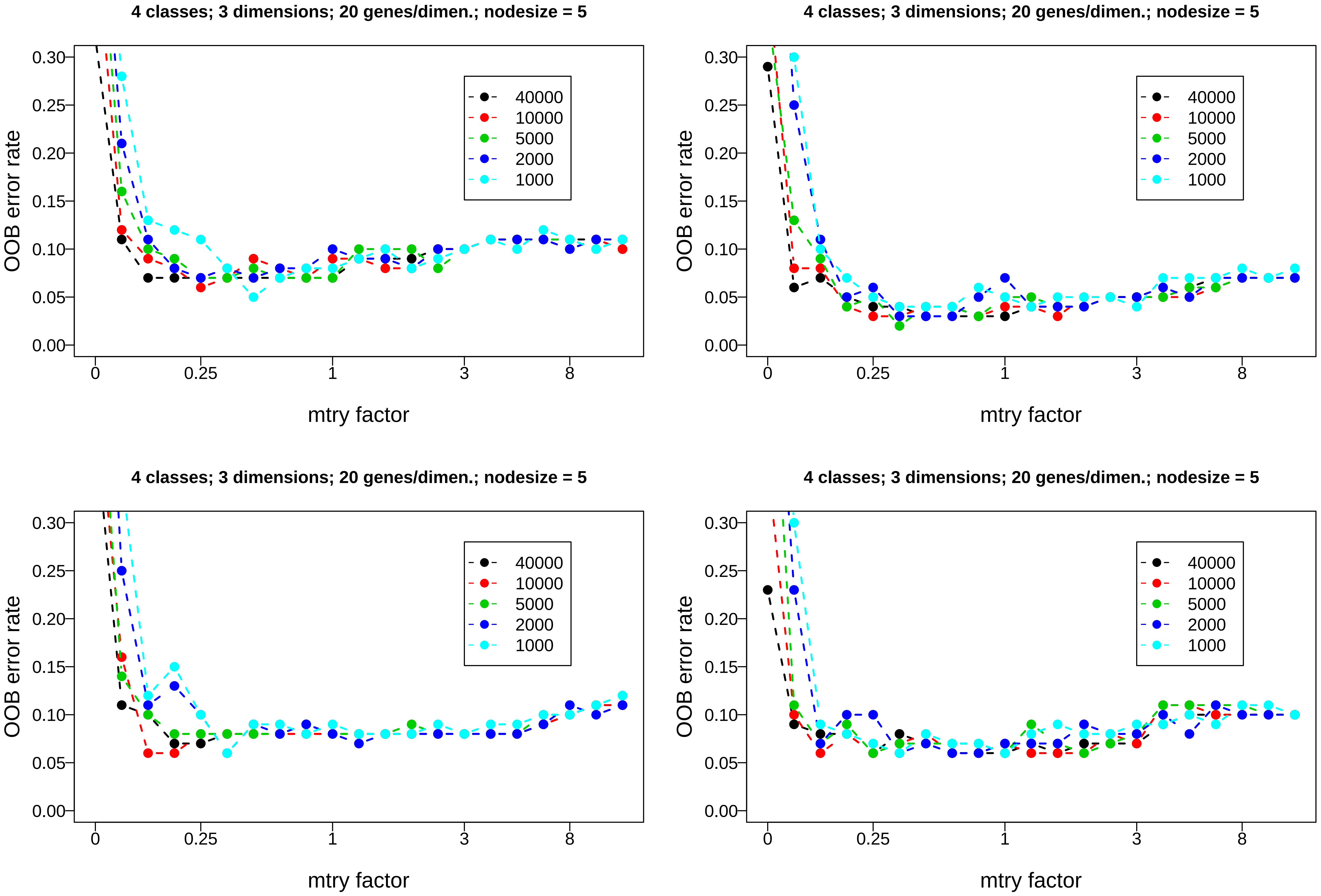
4 classes; 3 dimensions; 20 genes/dimen.; nodesize = 5
OOB error rate
mtry factor
0.00
0.05
0.10
0.15
0.20
0.25
0.30
0
0.25
1
3
8
40000
10000
5000
2000
1000
4 classes; 3 dimensions; 20 genes/dimen.; nodesize = 5
OOB error rate
mtry factor
40000
10000
5000
2000
1000
4 classes; 3 dimensions; 20 genes/dimen.; nodesize = 5
OOB error rate
mtry factor
40000
10000
5000
2000
1000
4 classes; 3 dimensions; 20 genes/dimen.; nodesize = 5
OOB error rate
mtry factor
40000
10000
5000
2000
1000

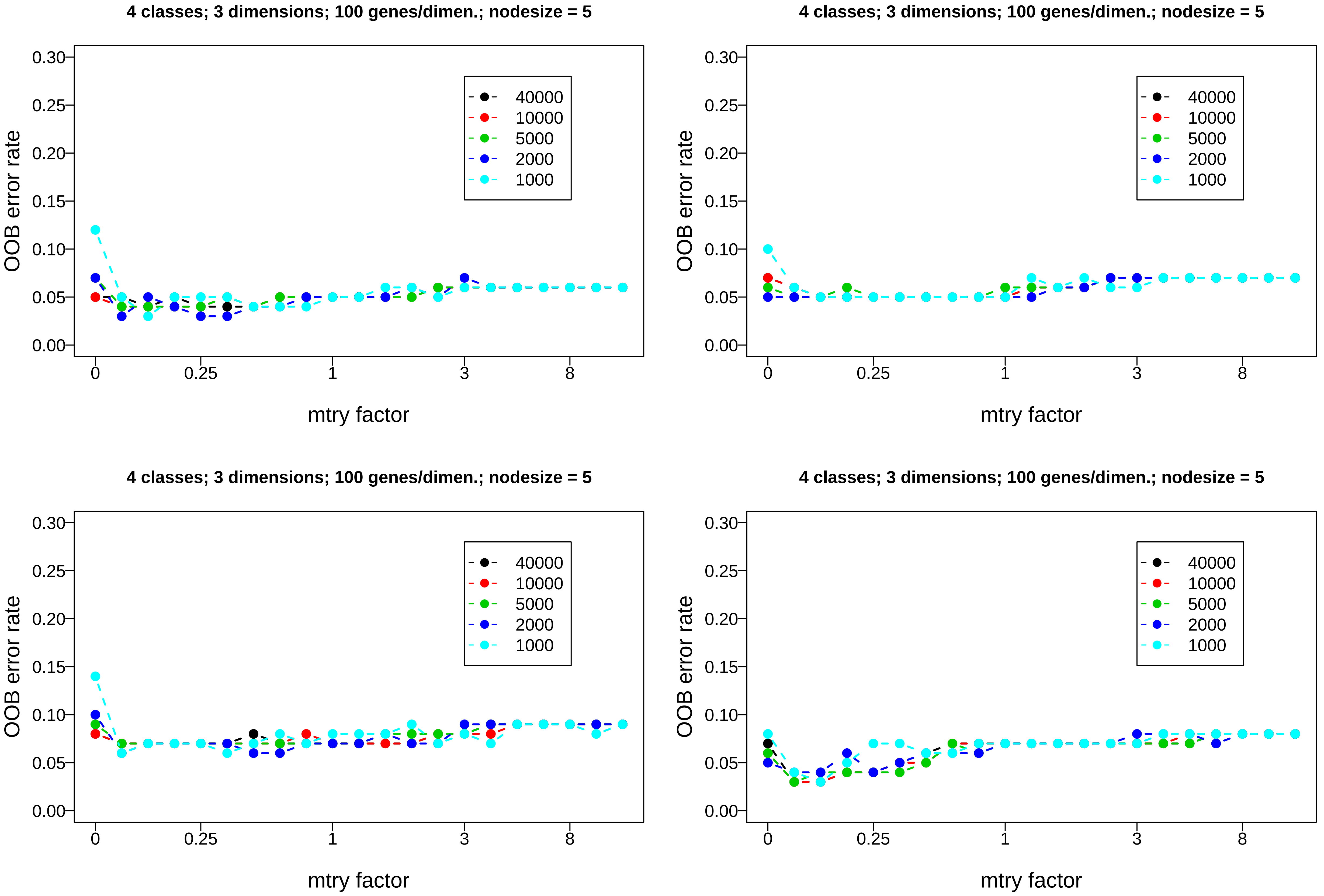

4 classes; 3 dimensions; 100 genes/dimen.; nodesize = 5
OOB error rate
0.00
0.05
0.10
0.15
0.20
0.25
0.30
mtry factor
0
0.25
1
3
8
40000
10000
5000
2000
1000
4 classes; 3 dimensions; 100 genes/dimen.; nodesize = 5
OOB error rate
mtry factor
40000
10000
5000
2000
1000
4 classes; 3 dimensions; 100 genes/dimen.; nodesize = 5
OOB error rate
mtry factor
40000
10000
5000
2000
1000
4 classes; 3 dimensions; 100 genes/dimen.; nodesize = 5
OOB error rate
mtry factor
40000
10000
5000
2000
1000

II: Simulated data sets:

No signal: 4000 noise random variables

(shown first with nodesize = 1, then nodesize = 5)

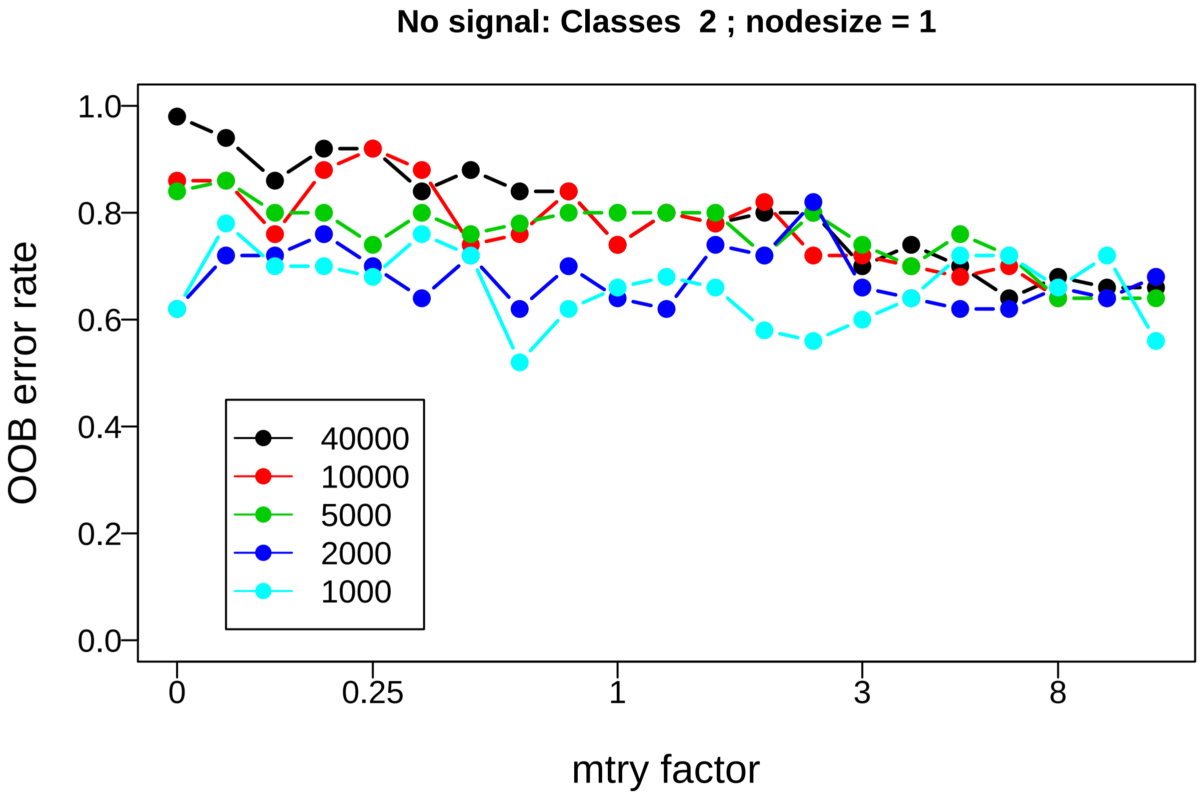
No signal: Classes 2 ; nodesize = 1
OOB error rate
mtry factor
1.0
0.8
0.6
0.4
0.2
0.0
0
0.25
1
3
8
40000
10000
5000
2000
1000

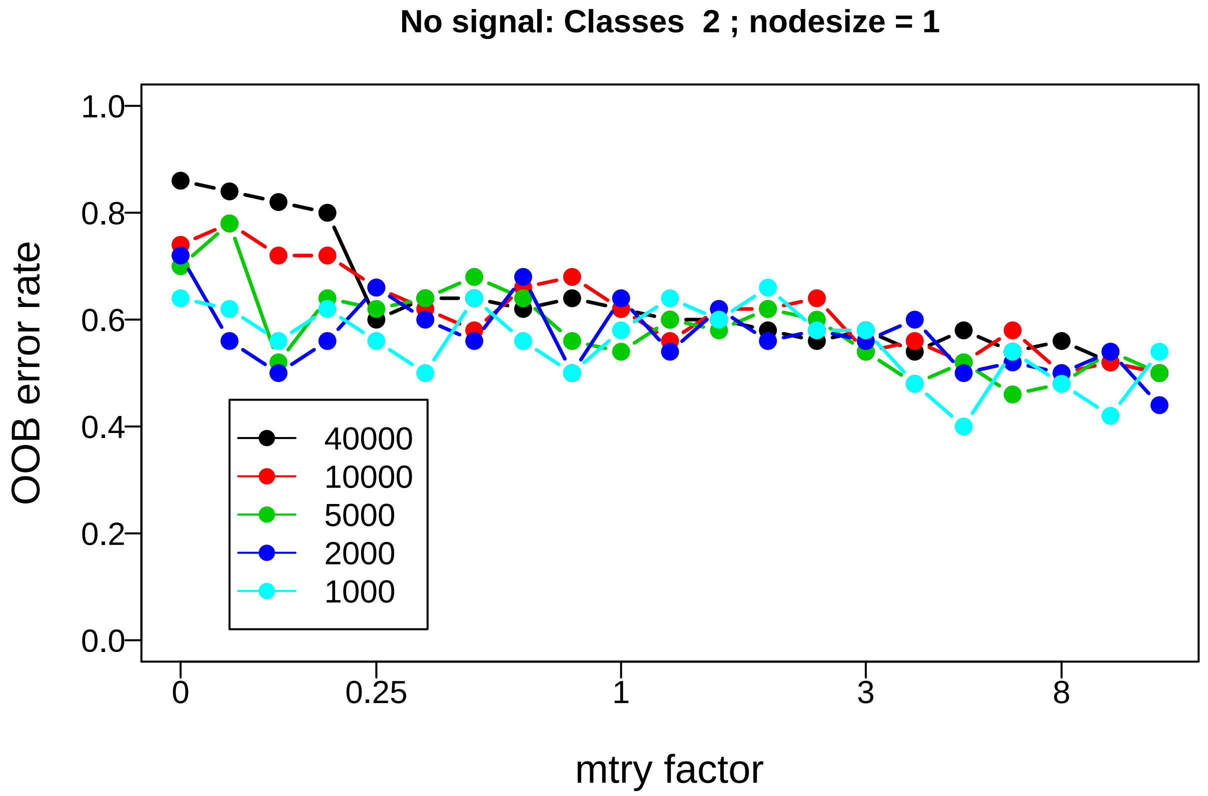
No signal: Classes 2 ; nodesize = 1
OOB error rate
mtry factor
1.0
0.8
0.6
0.4
0.2
0.0
0
0.25
1
3
8
40000
10000
5000
2000
1000

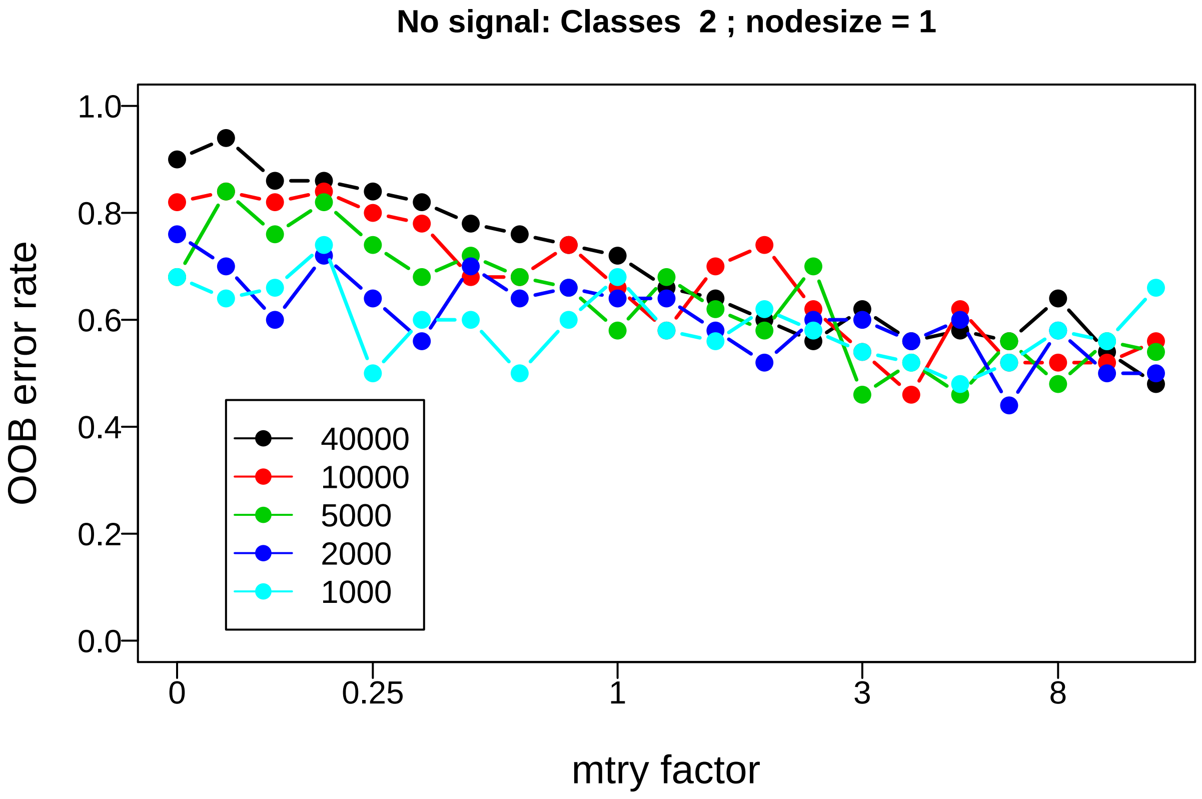
No signal: Classes 2 ; nodesize = 1
OOB error rate
mtry factor
1.0
0.8
0.6
0.4
0.2
0.0
0
0.25
1
3
8
40000
10000
5000
2000
1000

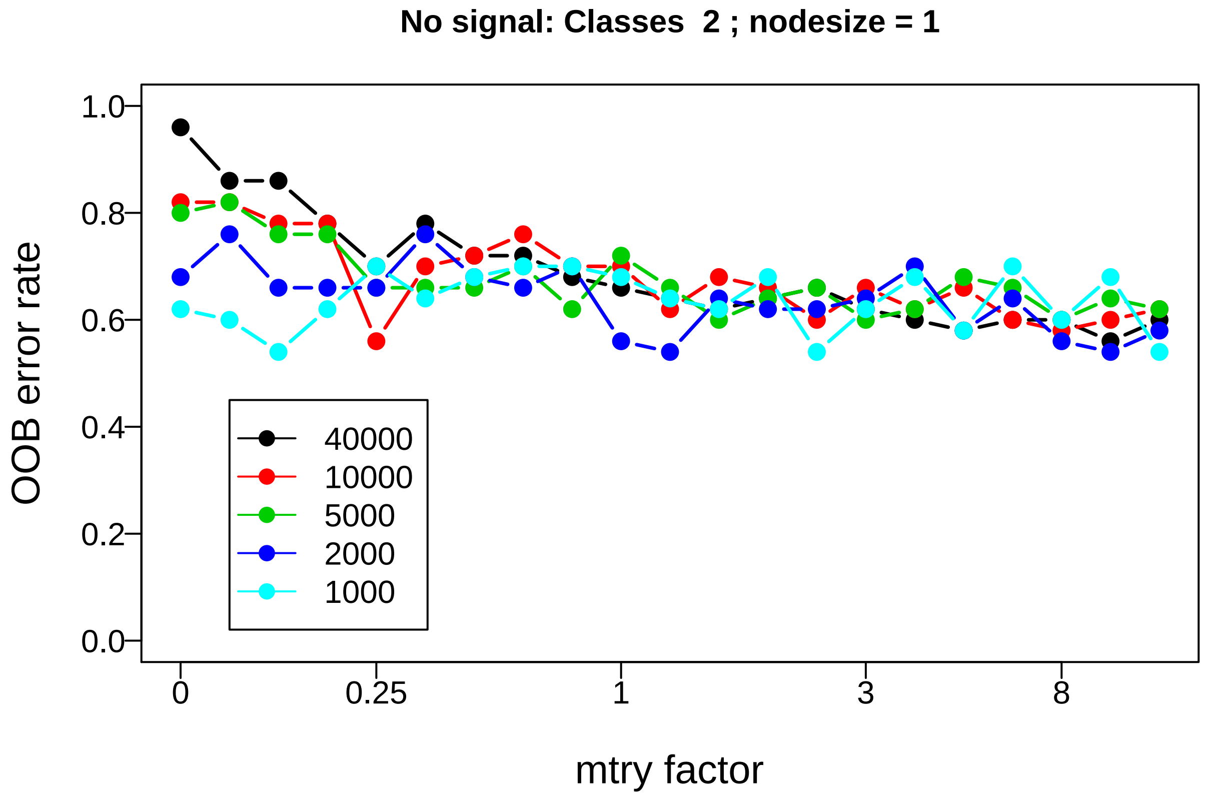
No signal: Classes 2 ; nodesize = 1
OOB error rate
mtry factor
1.0
0.8
0.6
0.4
0.2
0.0
0
0.25
1
3
8
40000
10000
5000
2000
1000

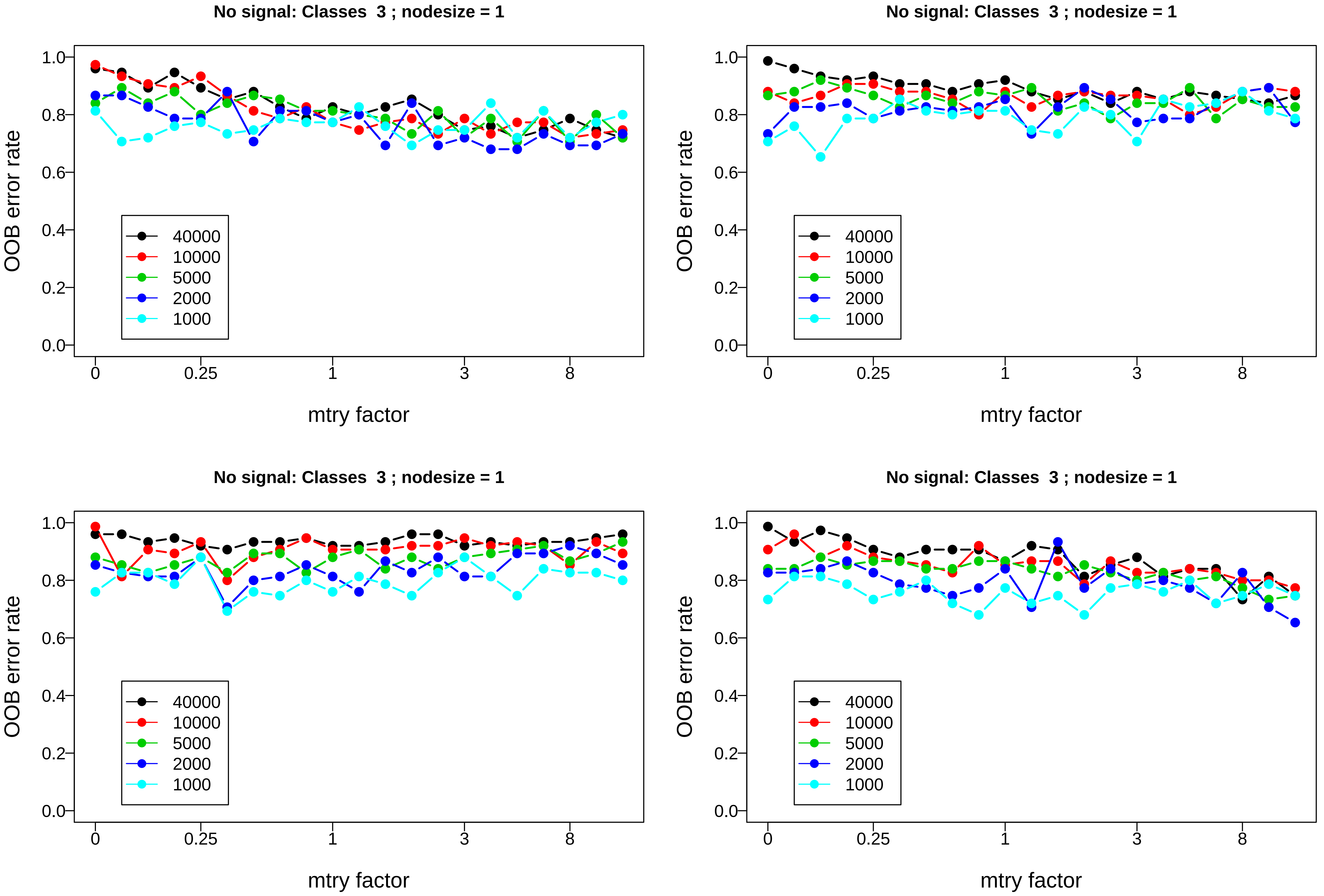
No signal: Classes 3 ; nodesize = 1
OOB error rate
mtry factor
40000
10000
5000
2000
1000
0
0.25
1
3
8
0.0
0.2
0.4
0.6
0.8
1.0

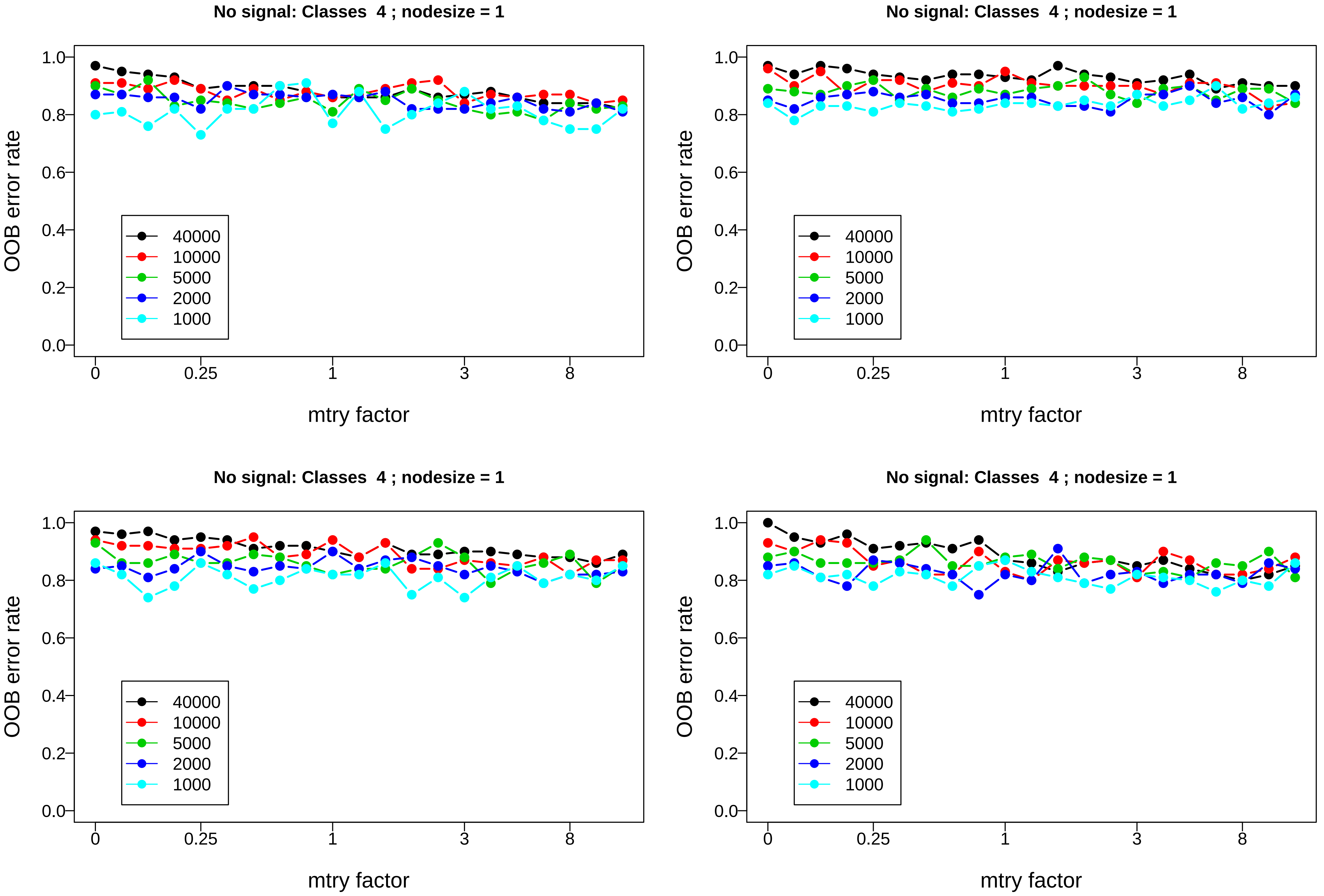

No signal: Classes 4 ; nodesize = 1
OOB error rate
mtry factor
0
0.25
1
3
8
0.0
0.2
0.4
0.6
0.8
1.0
40000
10000
5000
2000
1000
No signal: Classes 4 ; nodesize = 1
No signal: Classes 4 ; nodesize = 1
No signal: Classes 4 ; nodesize = 1

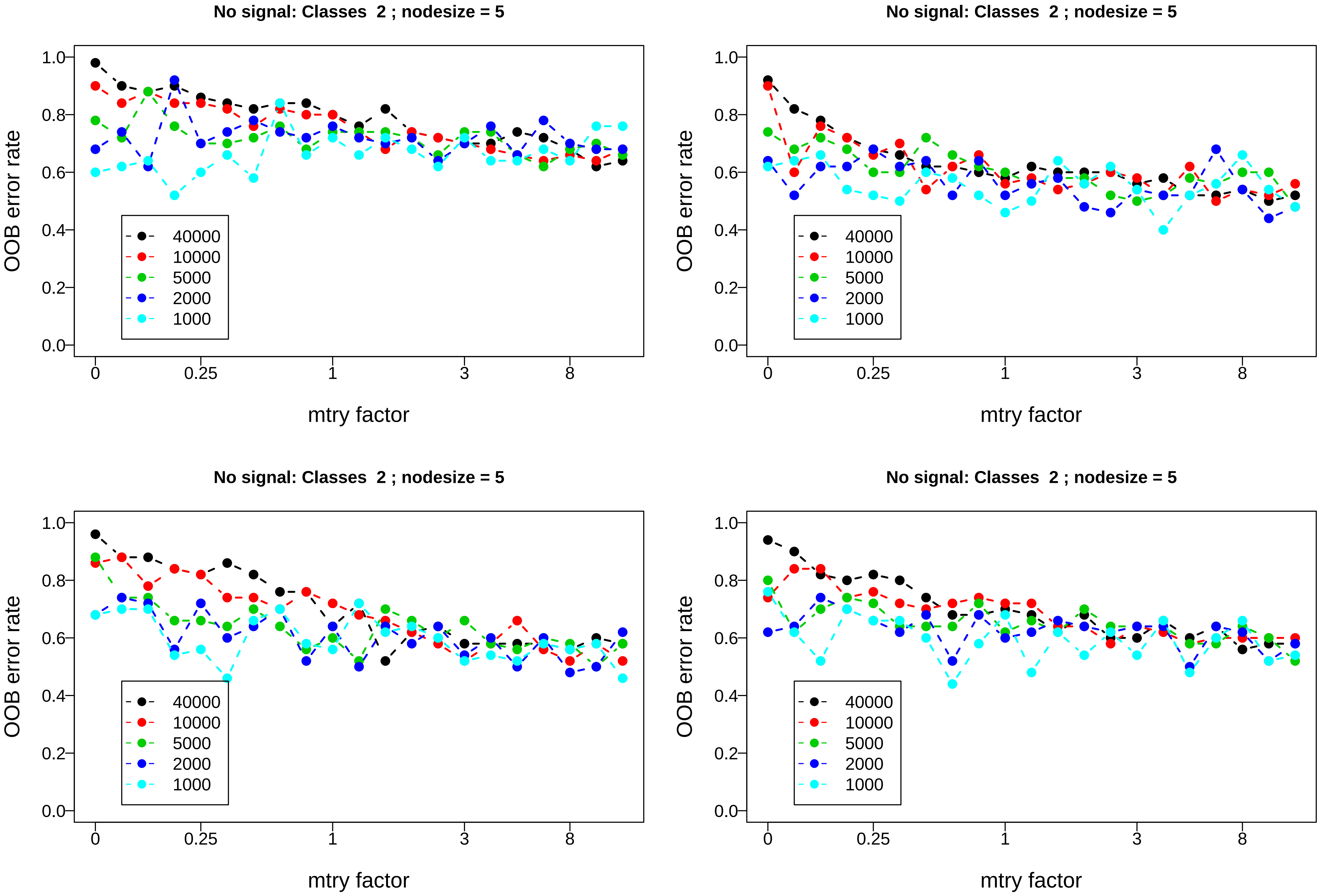

No signal: Classes 2 ; nodesize = 5
OOB error rate
mtry factor
0
0.25
1
3
8
0.0
0.2
0.4
0.6
0.8
1.0
40000
10000
5000
2000
1000

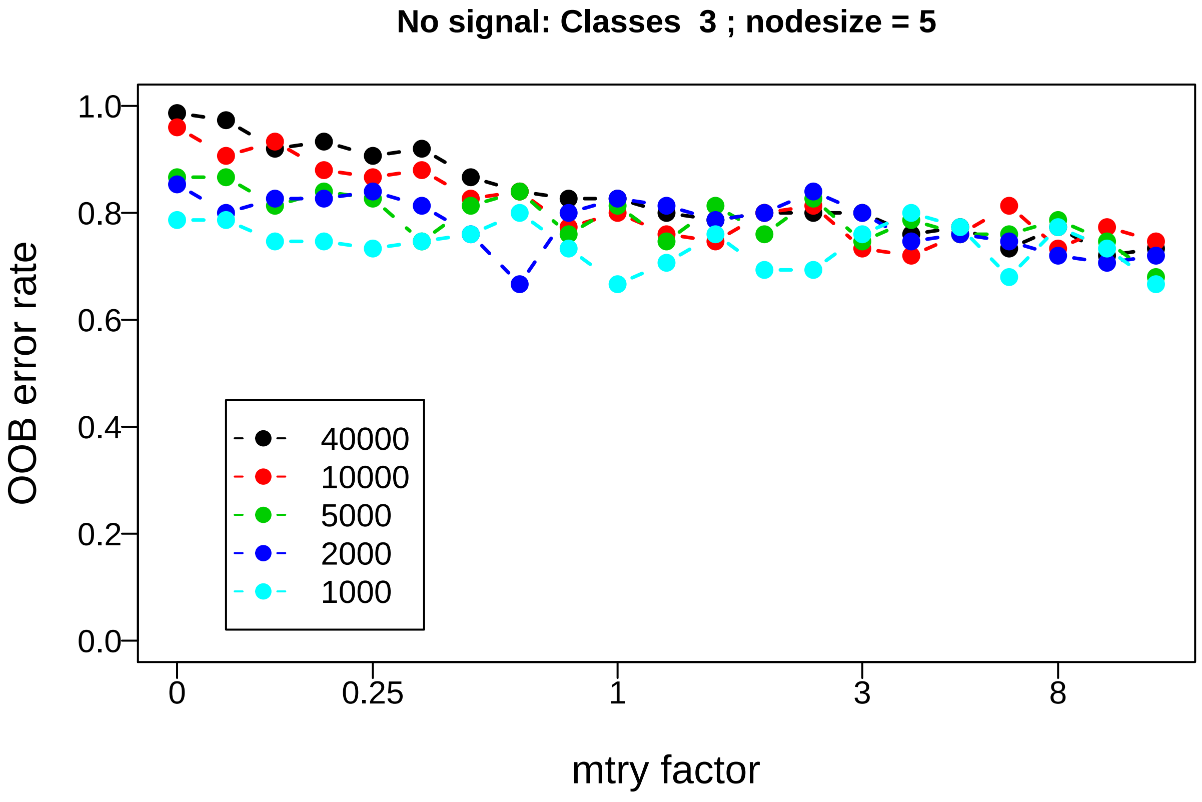
No signal: Classes 3 ; nodesize = 5
OOB error rate
mtry factor
0
0.25
1
3
8
0.0
0.2
0.4
0.6
0.8
1.0
40000
10000
5000
2000
1000
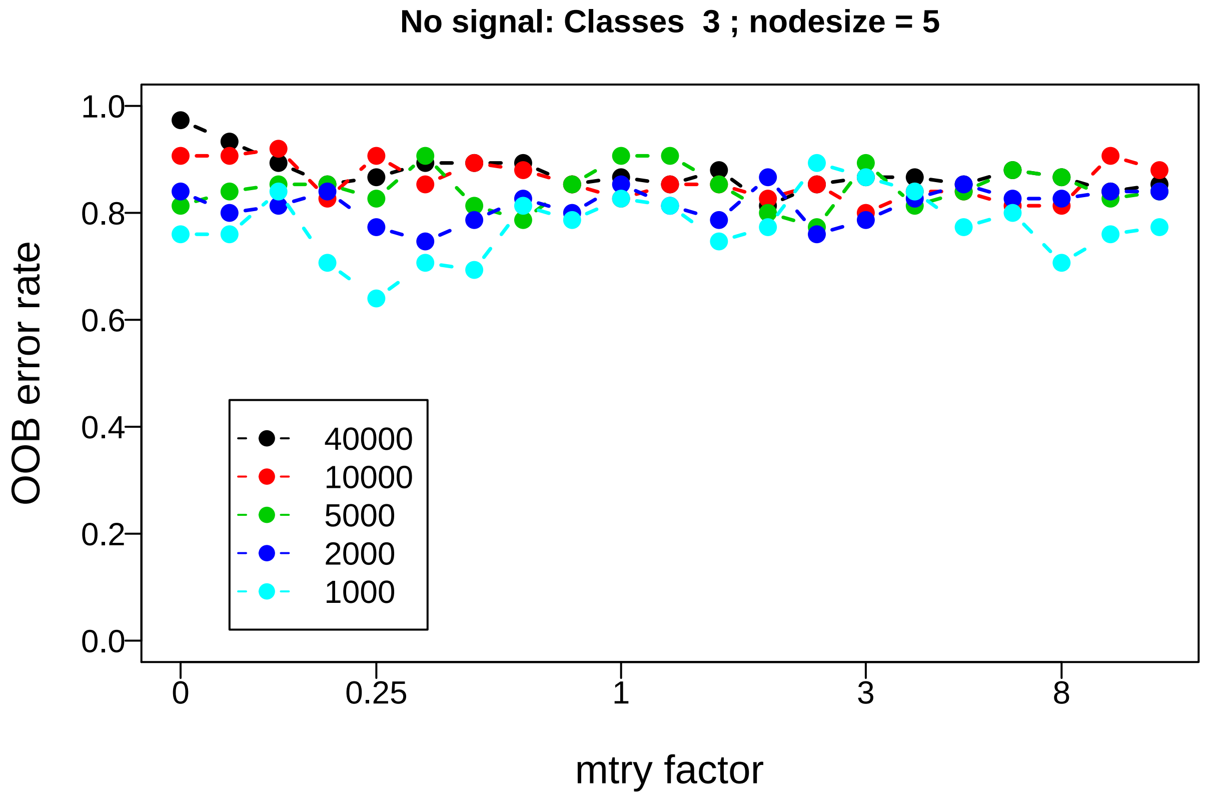
No signal: Classes 3 ; nodesize = 5
OOB error rate
mtry factor
0
0.25
1
3
8
0.0
0.2
0.4
0.6
0.8
1.0
40000
10000
5000
2000
1000
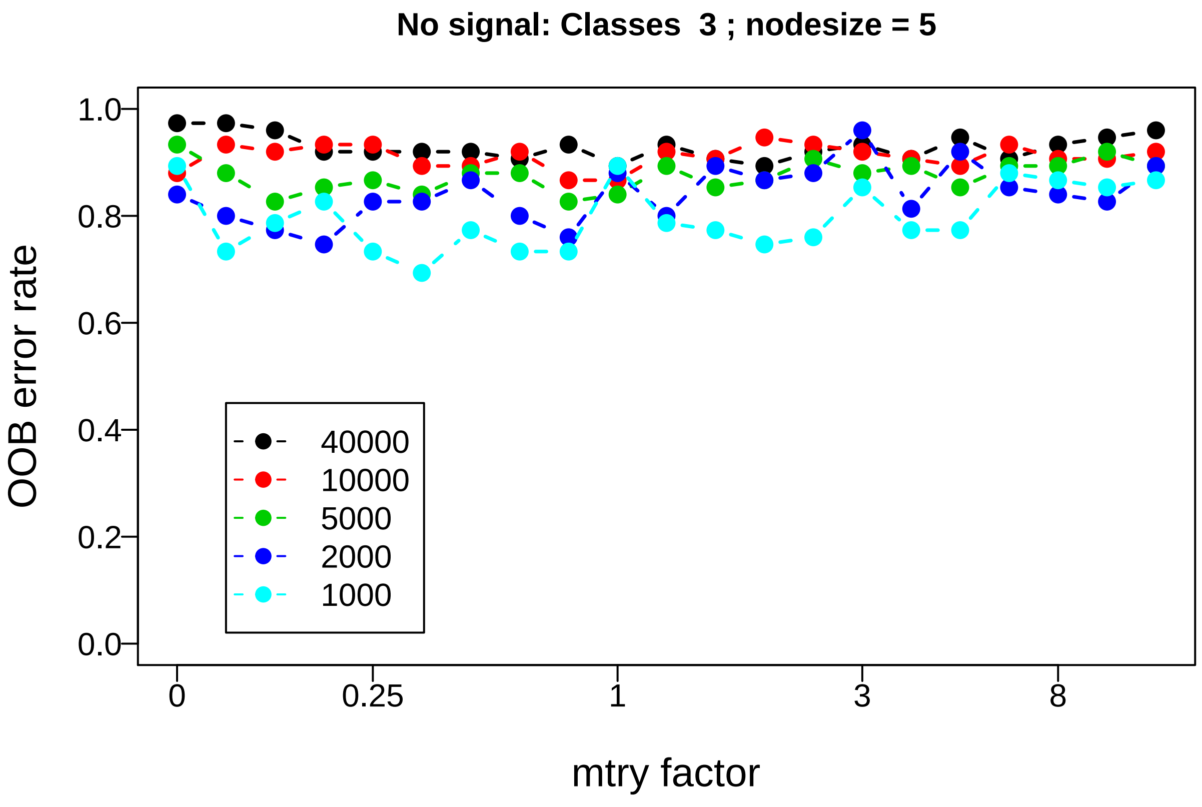
No signal: Classes 3 ; nodesize = 5
OOB error rate
mtry factor
0
0.25
1
3
8
0.0
0.2
0.4
0.6
0.8
1.0
40000
10000
5000
2000
1000
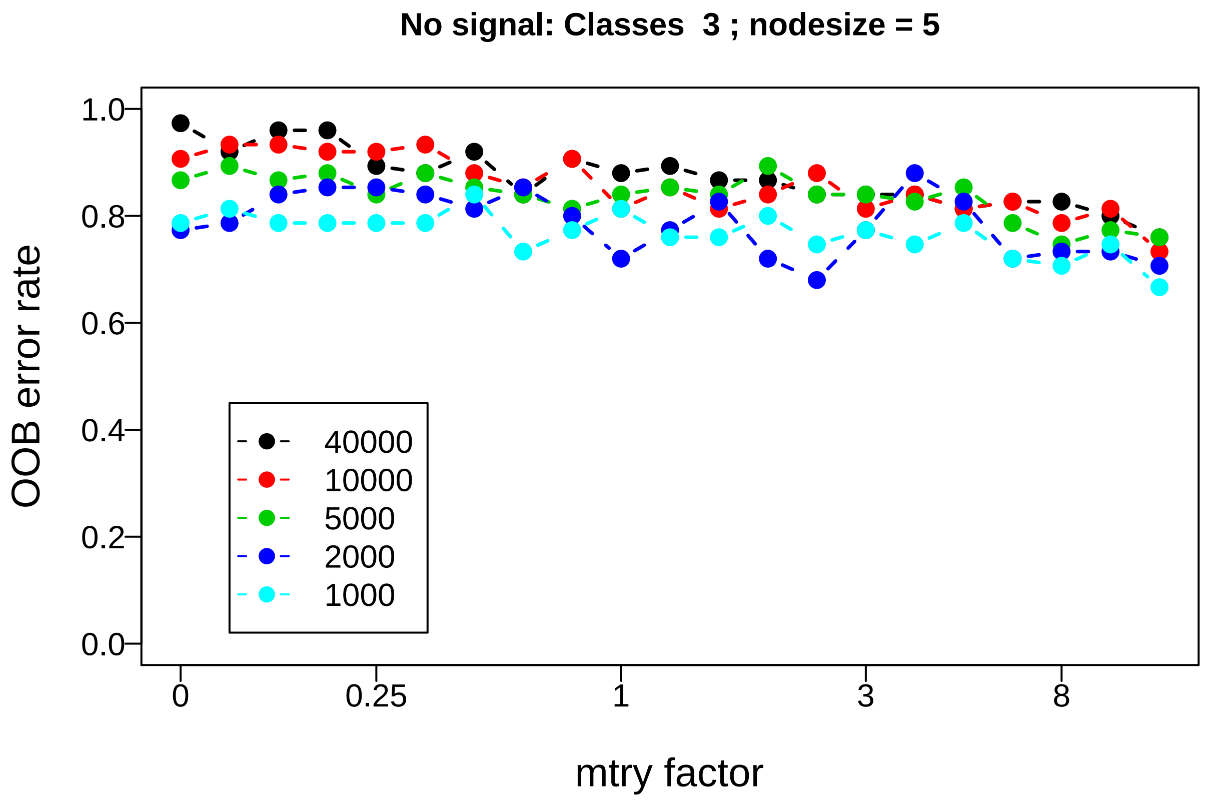
No signal: Classes 3 ; nodesize = 5
OOB error rate
mtry factor
0
0.25
1
3
8
0.0
0.2
0.4
0.6
0.8
1.0
40000
10000
5000
2000
1000

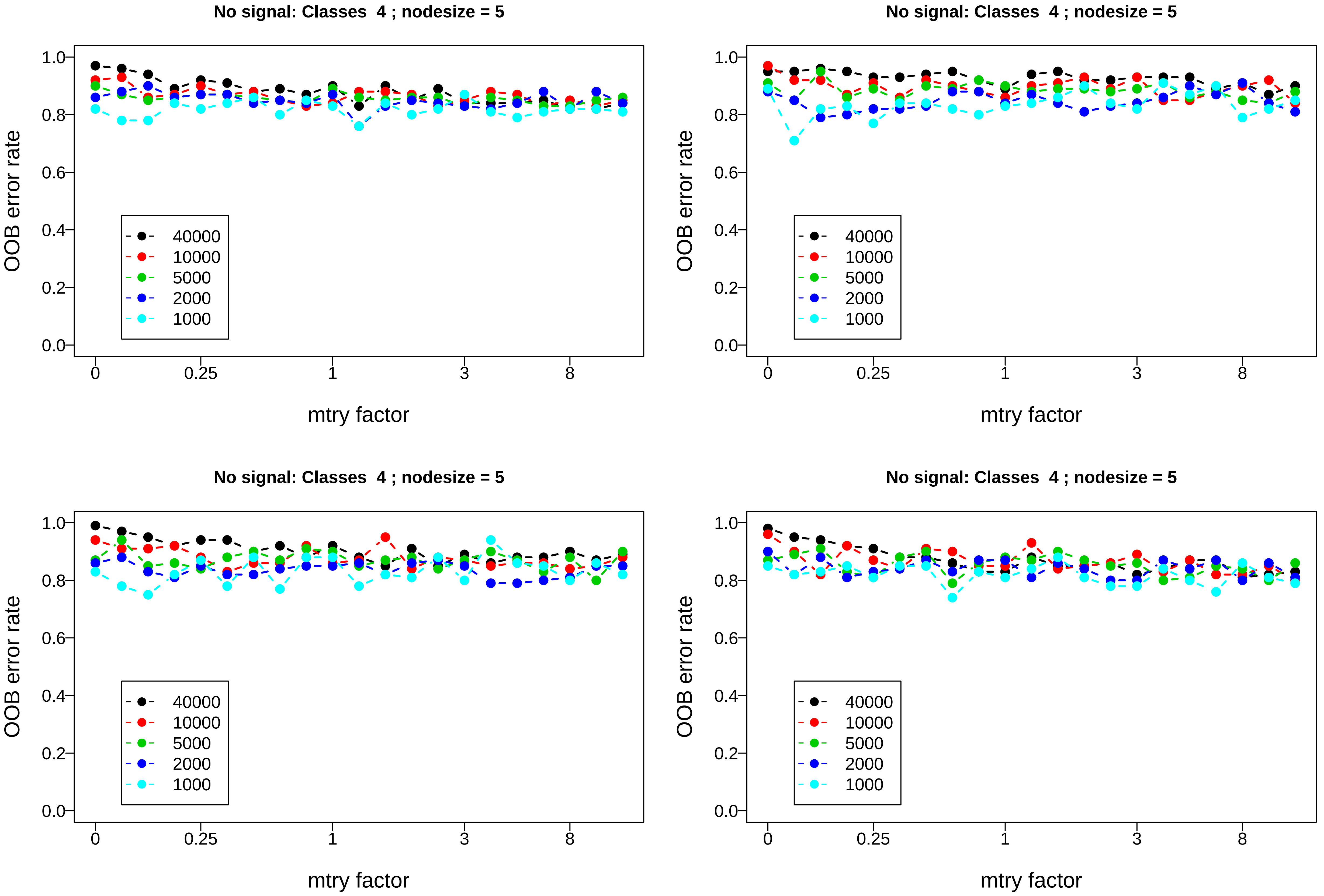
No signal: Classes 4 ; nodesize = 5
OOB error rate
mtry factor
0.0
0.2
0.4
0.6
0.8
1.0
0
0.25
1
3
8
40000
10000
5000
2000
1000
No signal: Classes 4 ; nodesize = 5
OOB error rate
mtry factor
No signal: Classes 4 ; nodesize = 5
OOB error rate
mtry factor
No signal: Classes 4 ; nodesize = 5
OOB error rate
mtry factor

III: Real microarray data sets:

(left: nodesize = 1, right: nodesize = 5)

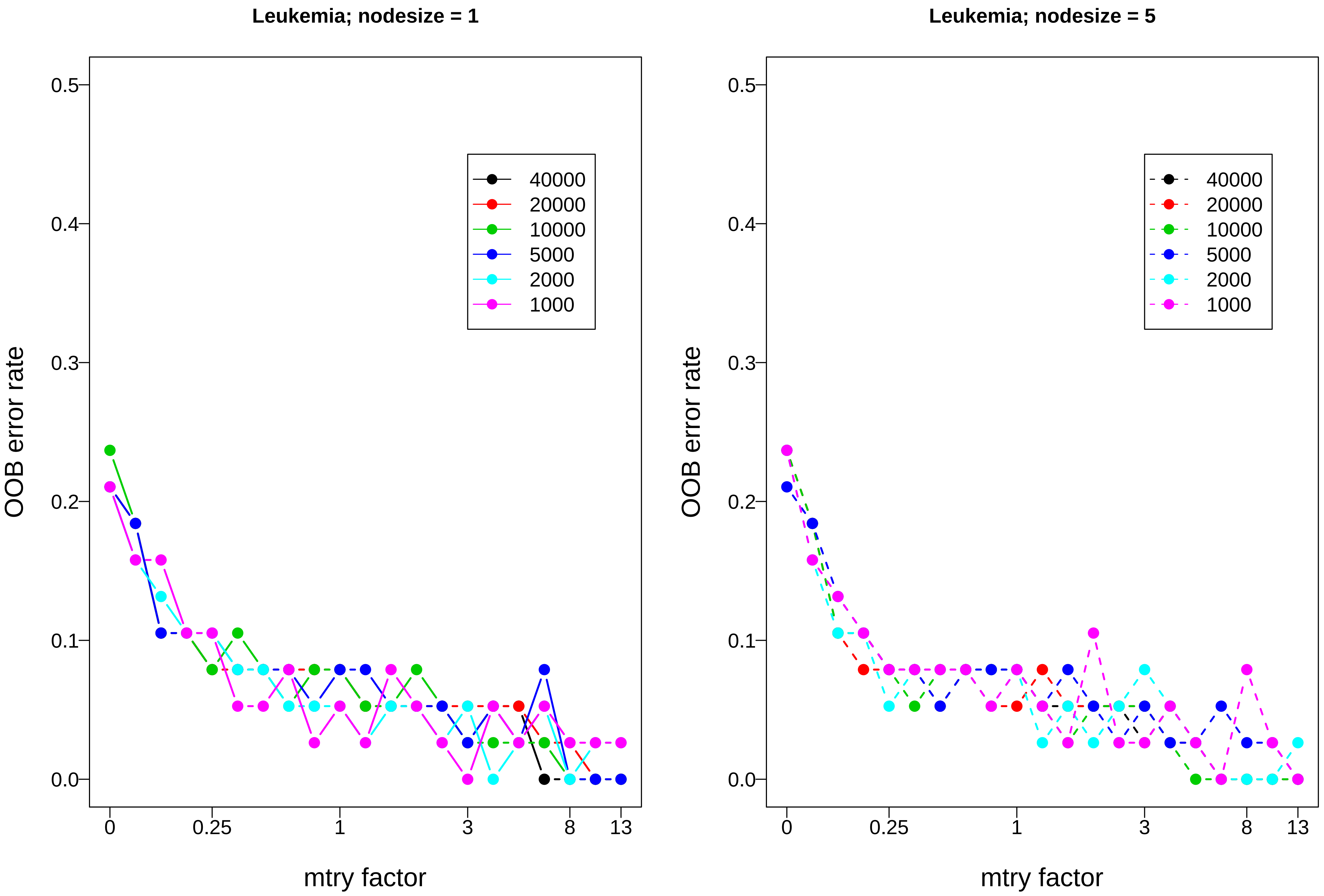

Leukemia; nodesize = 1
Leukemia; nodesize = 5
OOB error rate
mtry factor
0.0
0.1
0.2
0.3
0.4
0.5
0
0.25
1
3
8
13
40000
20000
10000
5000
2000
1000

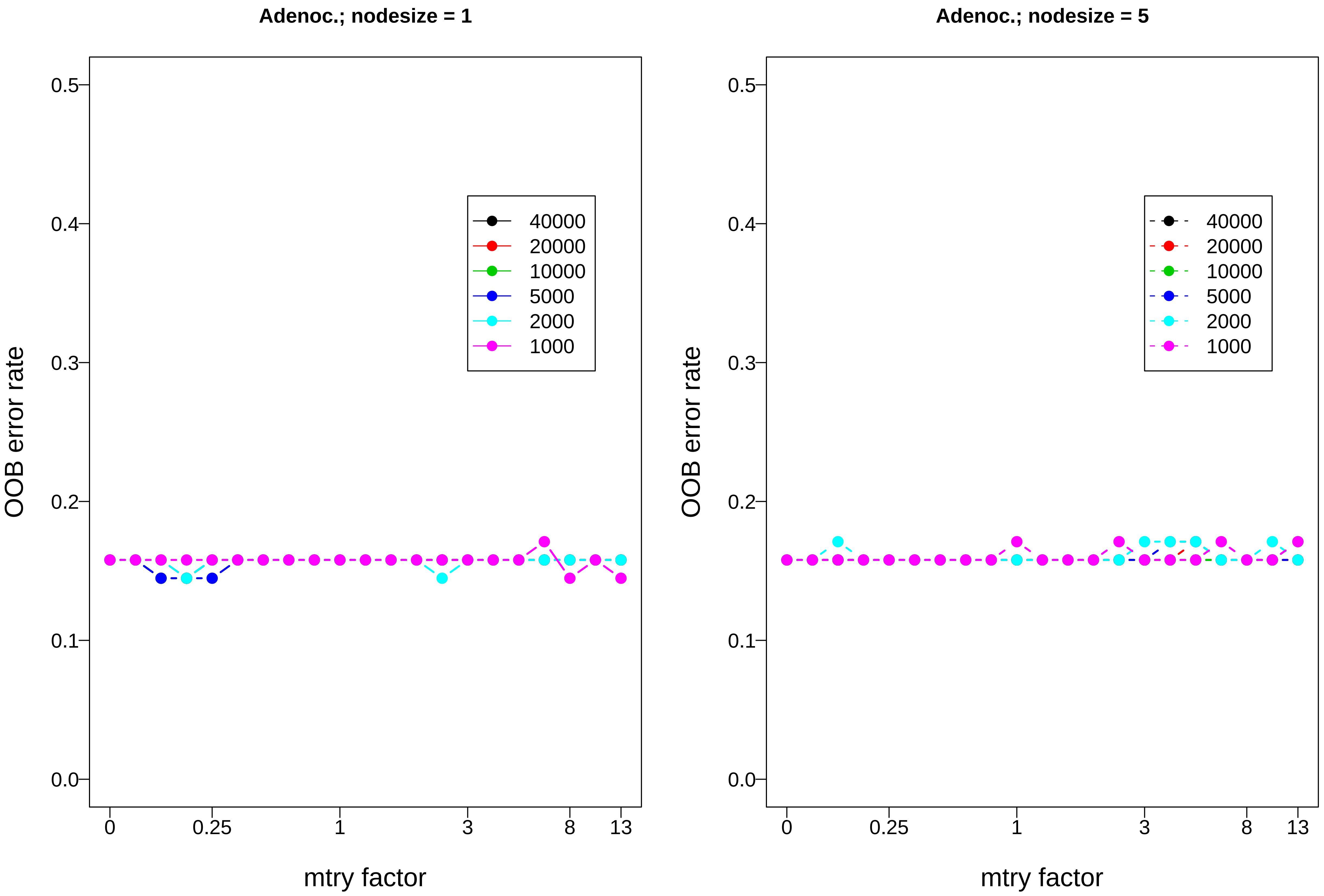
Adenoc.; nodesize = 1
Adenoc.; nodesize = 5
OOB error rate
0.0
0.1
0.2
0.3
0.4
0.5
mtry factor
0
0.25
1
3
8
13
40000
20000
10000
5000
2000
1000

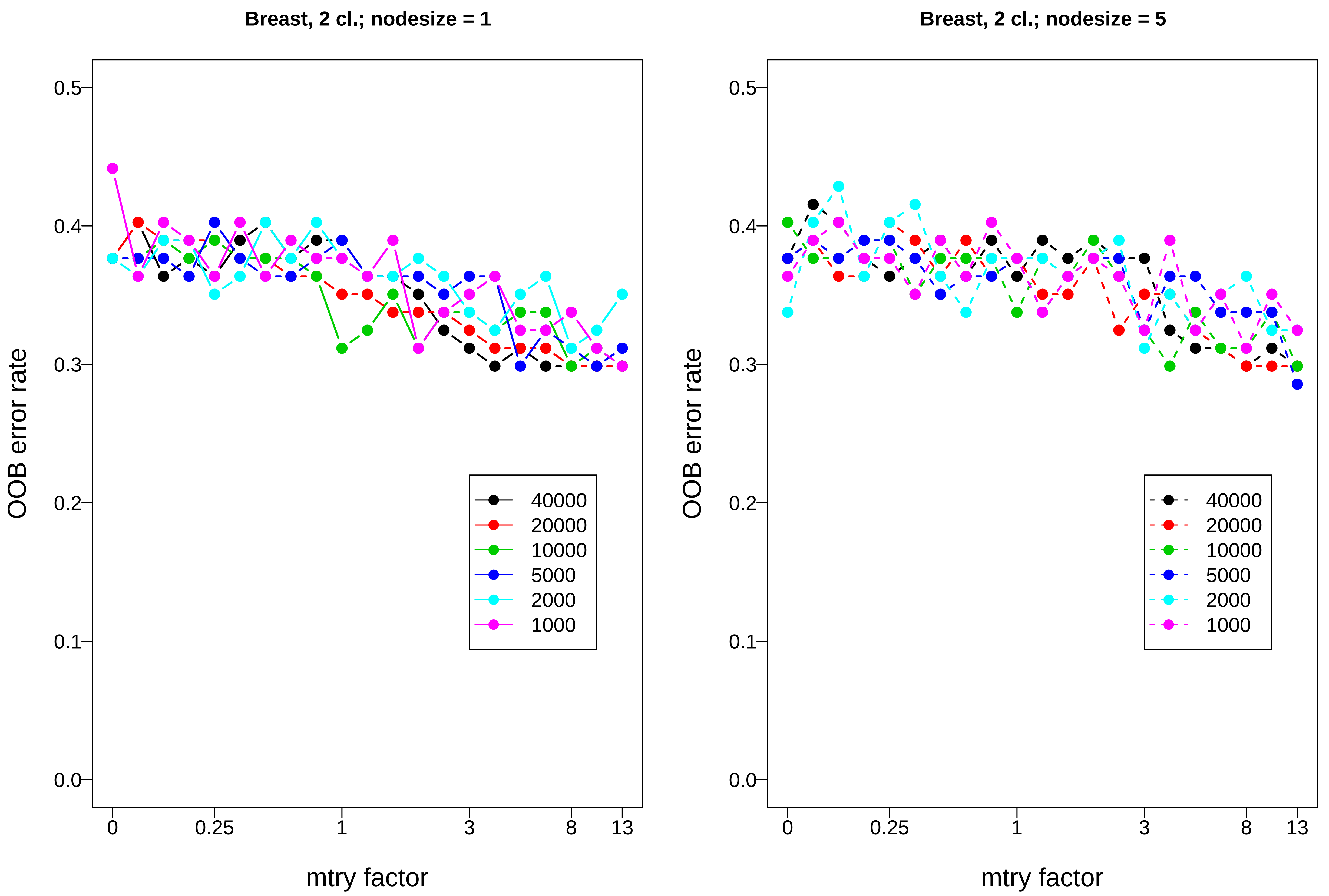

Breast, 2 cl.; nodesize = 1
Breast, 2 cl.; nodesize = 5
OOB error rate
0.0
0.1
0.2
0.3
0.4
0.5
mtry factor
0
0.25
1
3
8
13
40000
20000
10000
5000
2000
1000

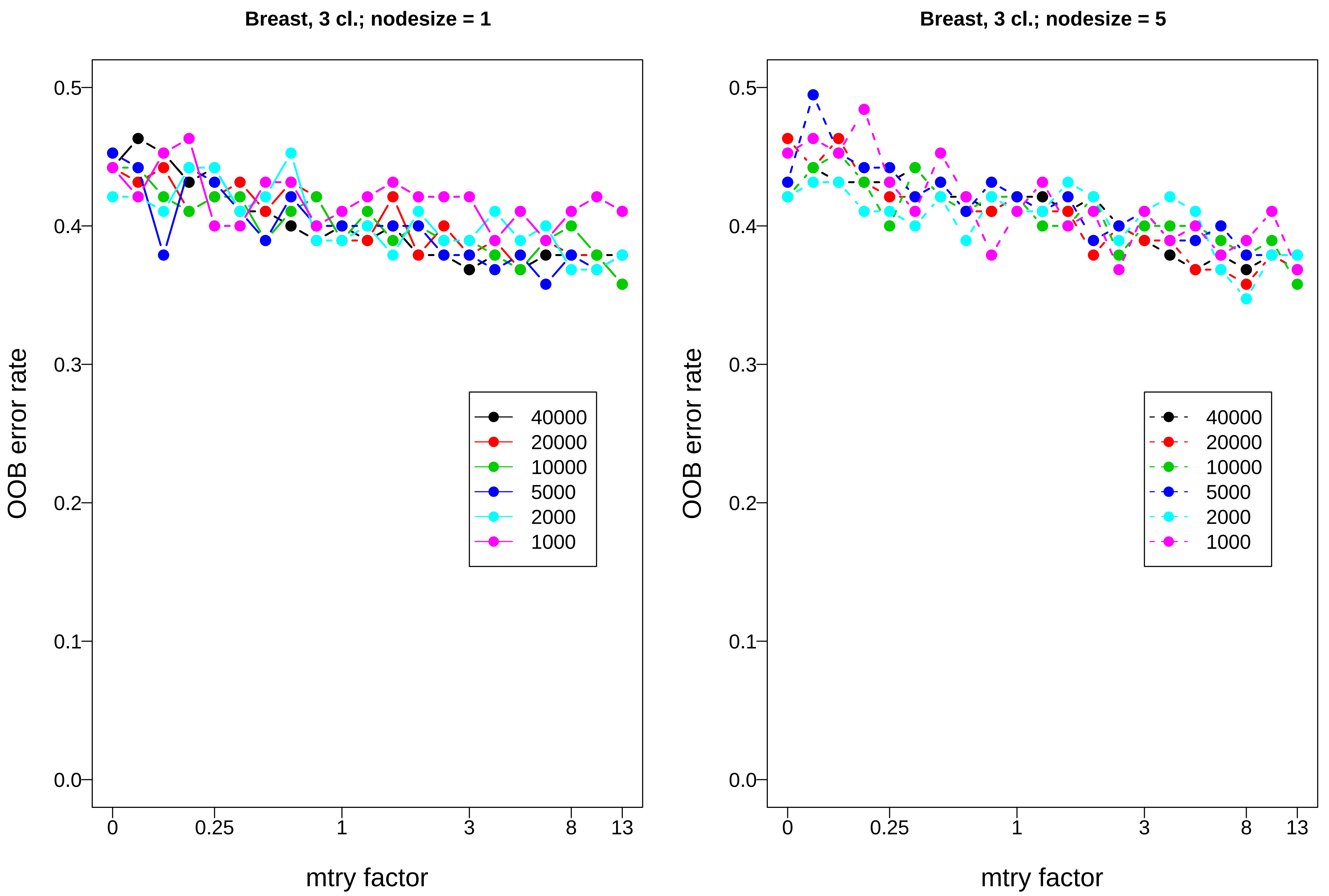
Breast, 3 cl.; nodesize = 1
OOB error rate
mtry factor
0.0
0.1
0.2
0.3
0.4
0.5
0
0.25
1
3
8
13
40000
20000
10000
5000
2000
1000
Breast, 3 cl.; nodesize = 5
OOB error rate
mtry factor
0.0
0.1
0.2
0.3
0.4
0.5
0
0.25
1
3
8
13
40000
20000
10000
5000
2000
1000

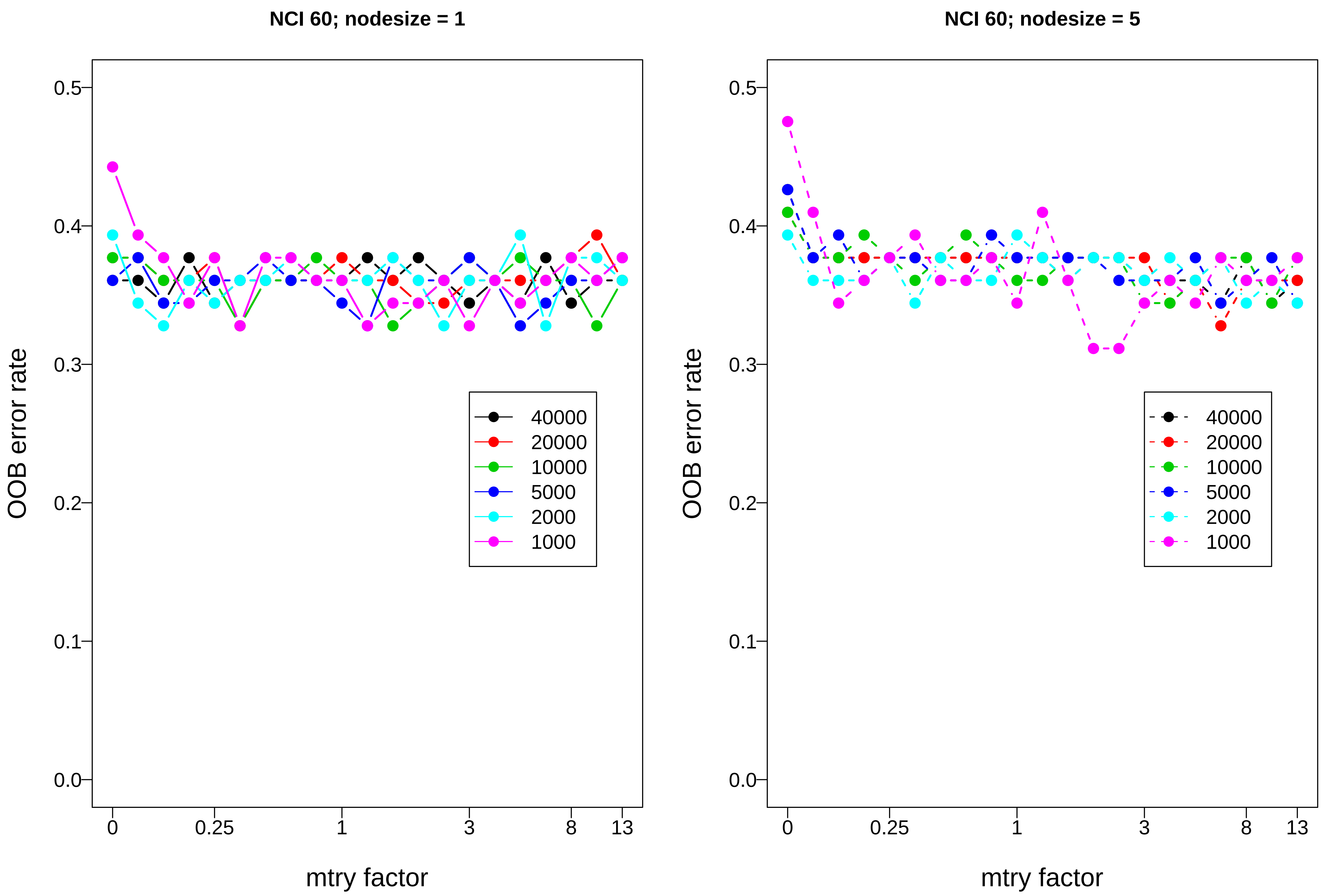
NCI 60; nodesize = 1
NCI 60; nodesize = 5
OOB error rate
mtry factor
0.0
0.1
0.2
0.3
0.4
0.5
0
0.25
1
3
8
13
40000
20000
10000
5000
2000
1000

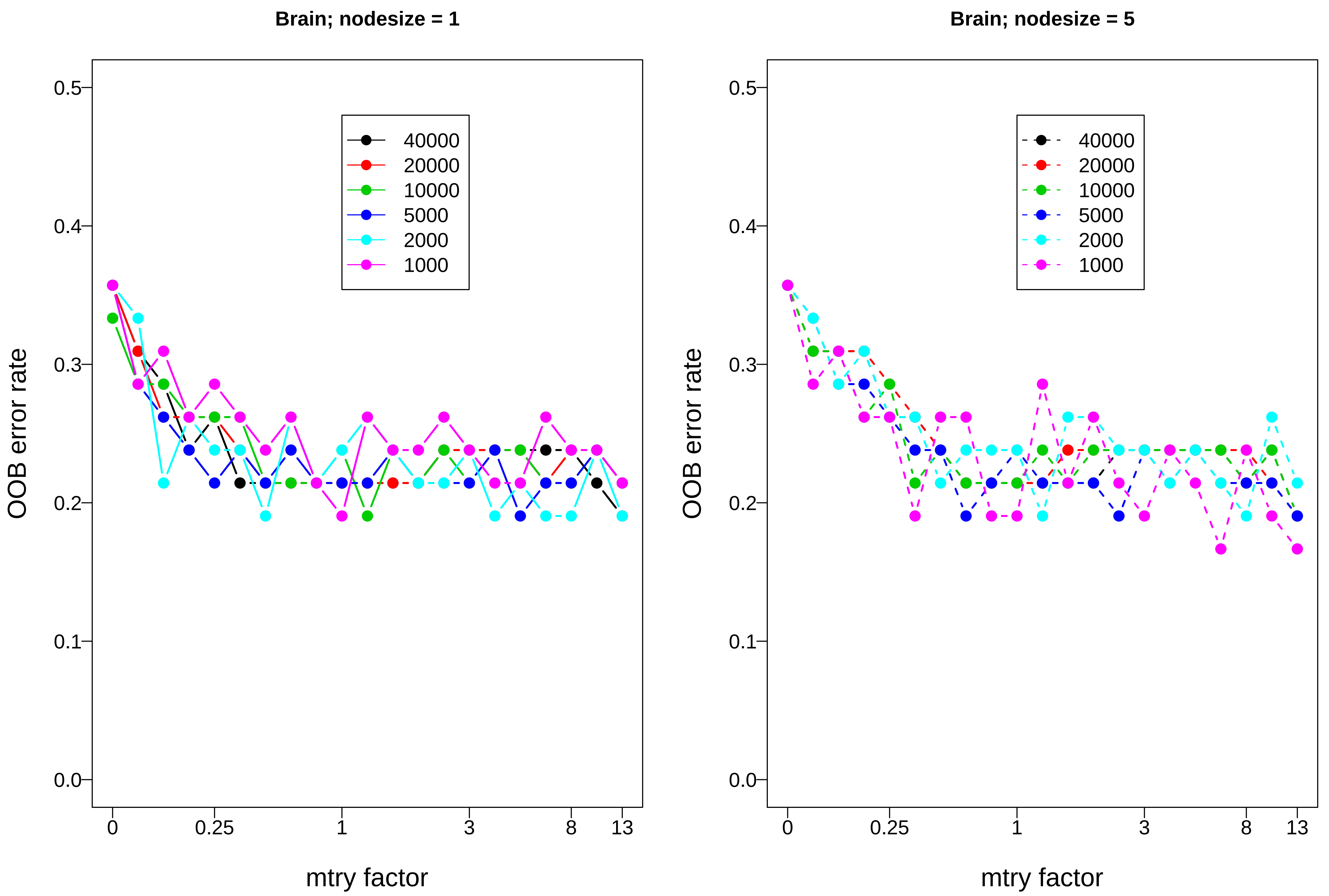

Brain; nodesize = 1
Brain; nodesize = 5
OOB error rate
mtry factor
0.0
0.1
0.2
0.3
0.4
0.5
0
0.25
1
3
8
13
40000
20000
10000
5000
2000
1000

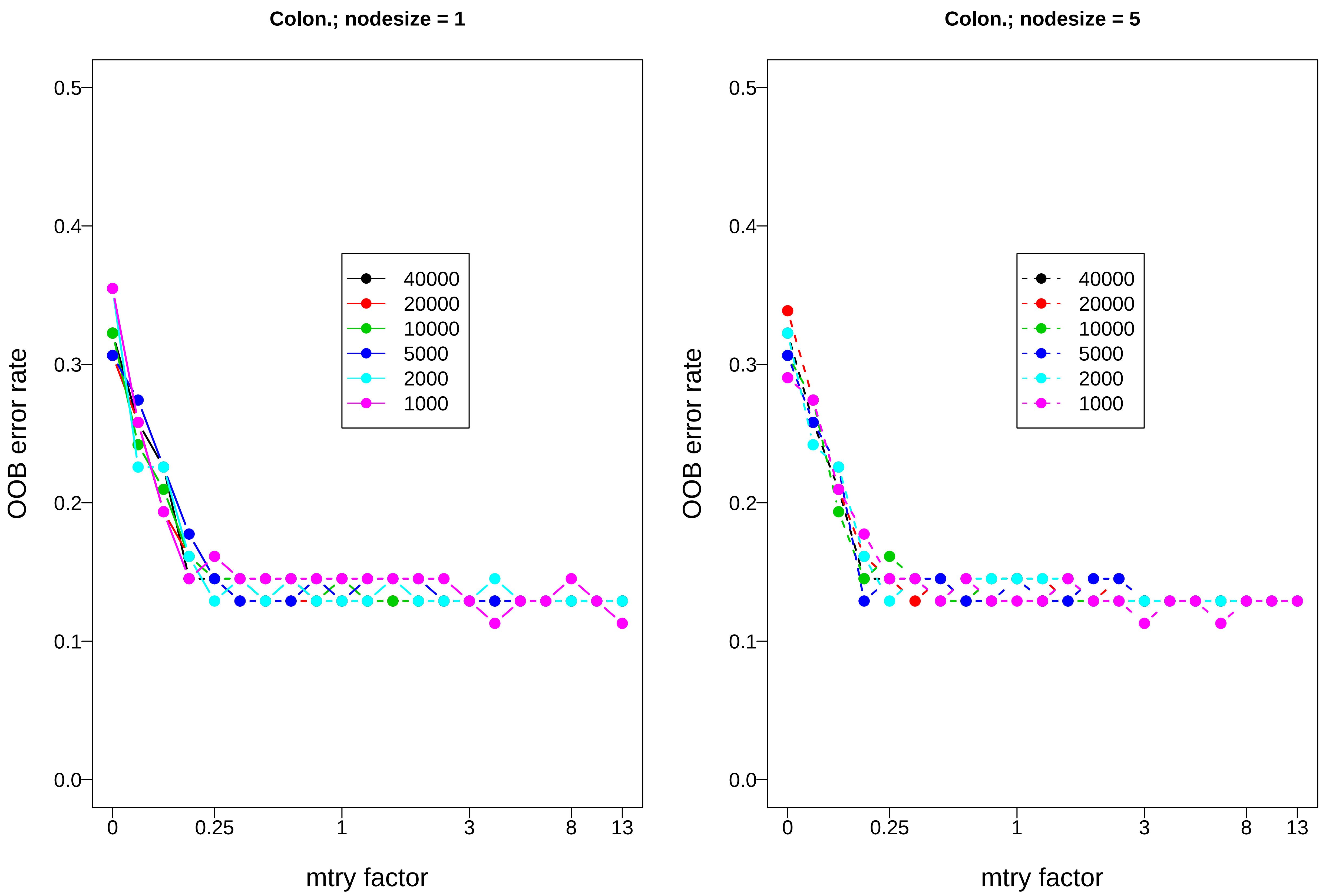

Colon.; nodesize = 1
Colon.; nodesize = 5
OOB error rate
mtry factor
0.0
0.1
0.2
0.3
0.4
0.5
0
0.25
1
3
8
13
40000
20000
10000
5000
2000
1000

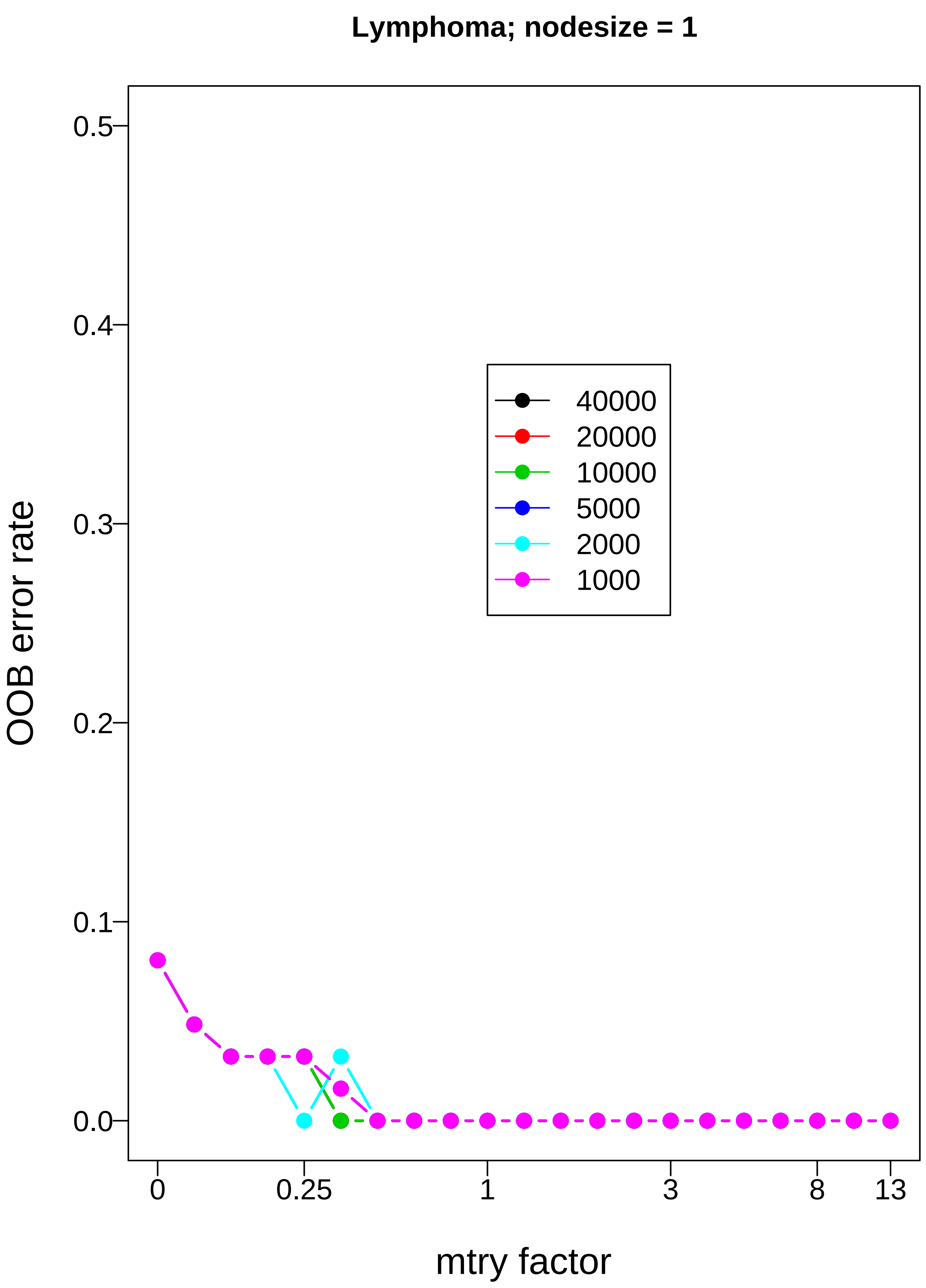

Lymphoma; nodesize = 1
OOB error rate
0.5
0.4
0.3
0.2
0.1
0.0
40000
20000
10000
5000
2000
1000
0
0.25
1
3
8
13
mtry factor

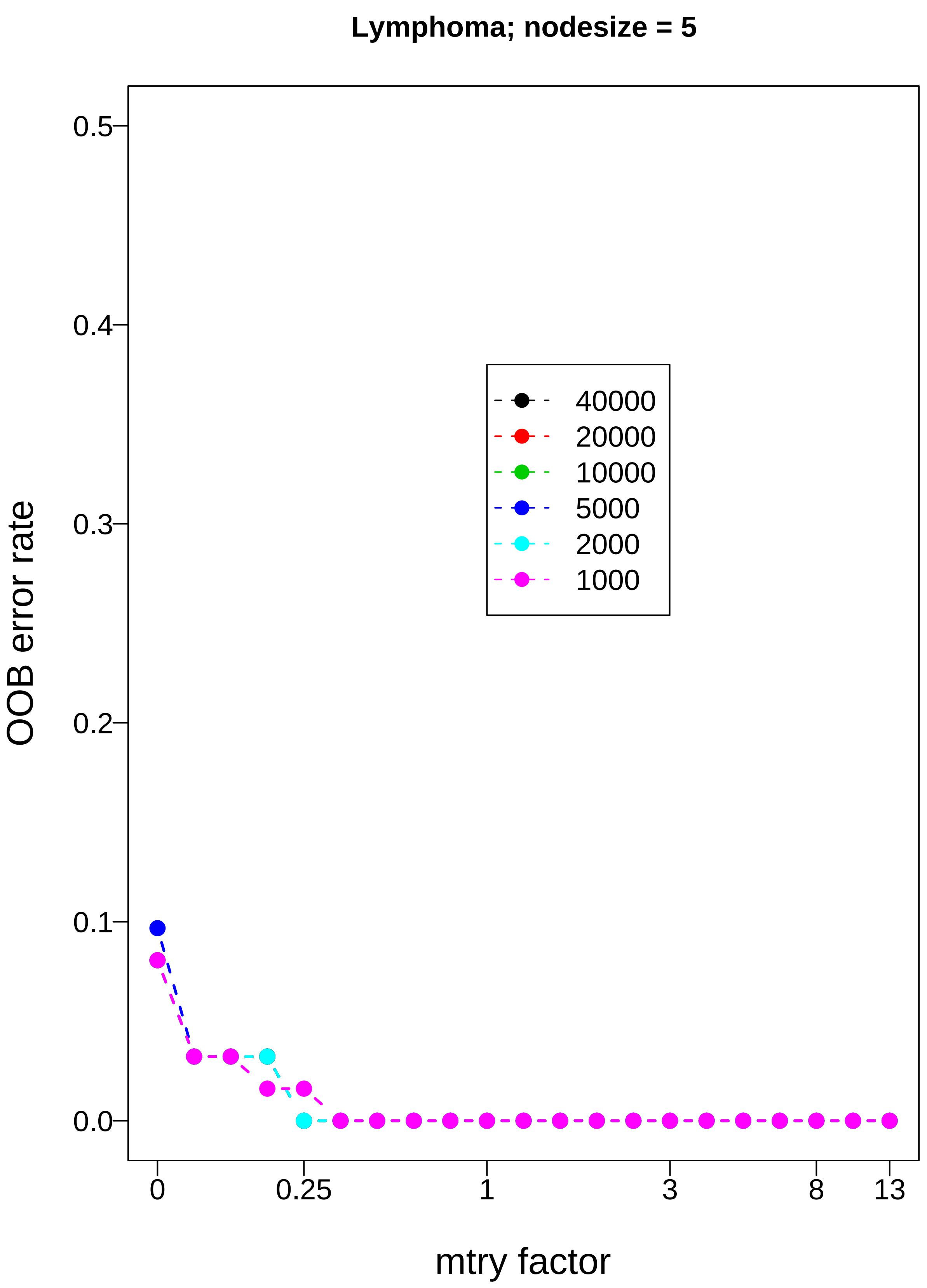

Lymphoma; nodesize = 5
OOB error rate
0.5
0.4
0.3
0.2
0.1
0.0
40000
20000
10000
5000
2000
1000
0
0.25
1
3
8
13
mtry factor

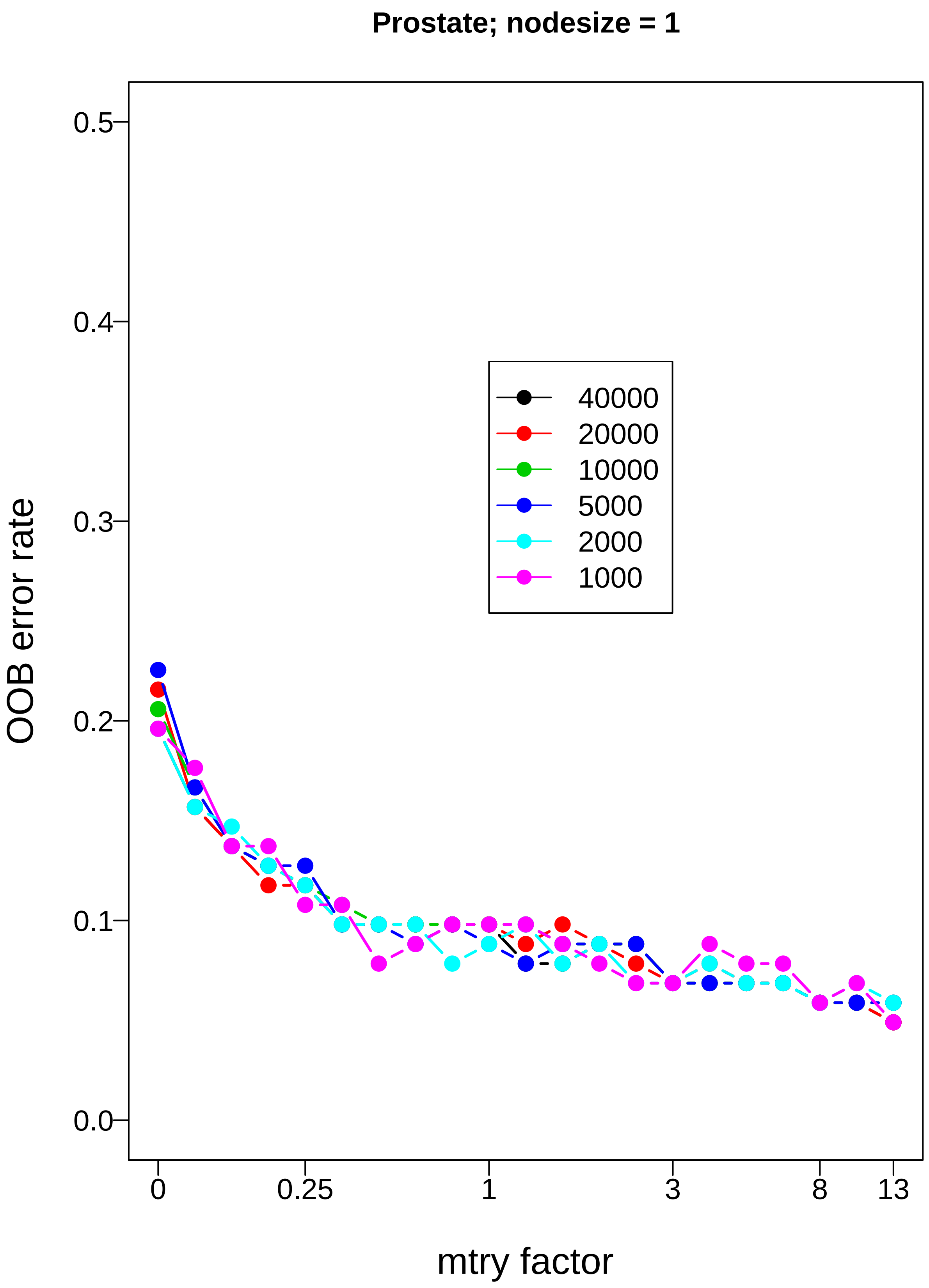
Prostate; nodesize = 1
OOB error rate
mtry factor
0.0
0.1
0.2
0.3
0.4
0.5
0
0.25
1
3
8
13
40000
20000
10000
5000
2000
1000

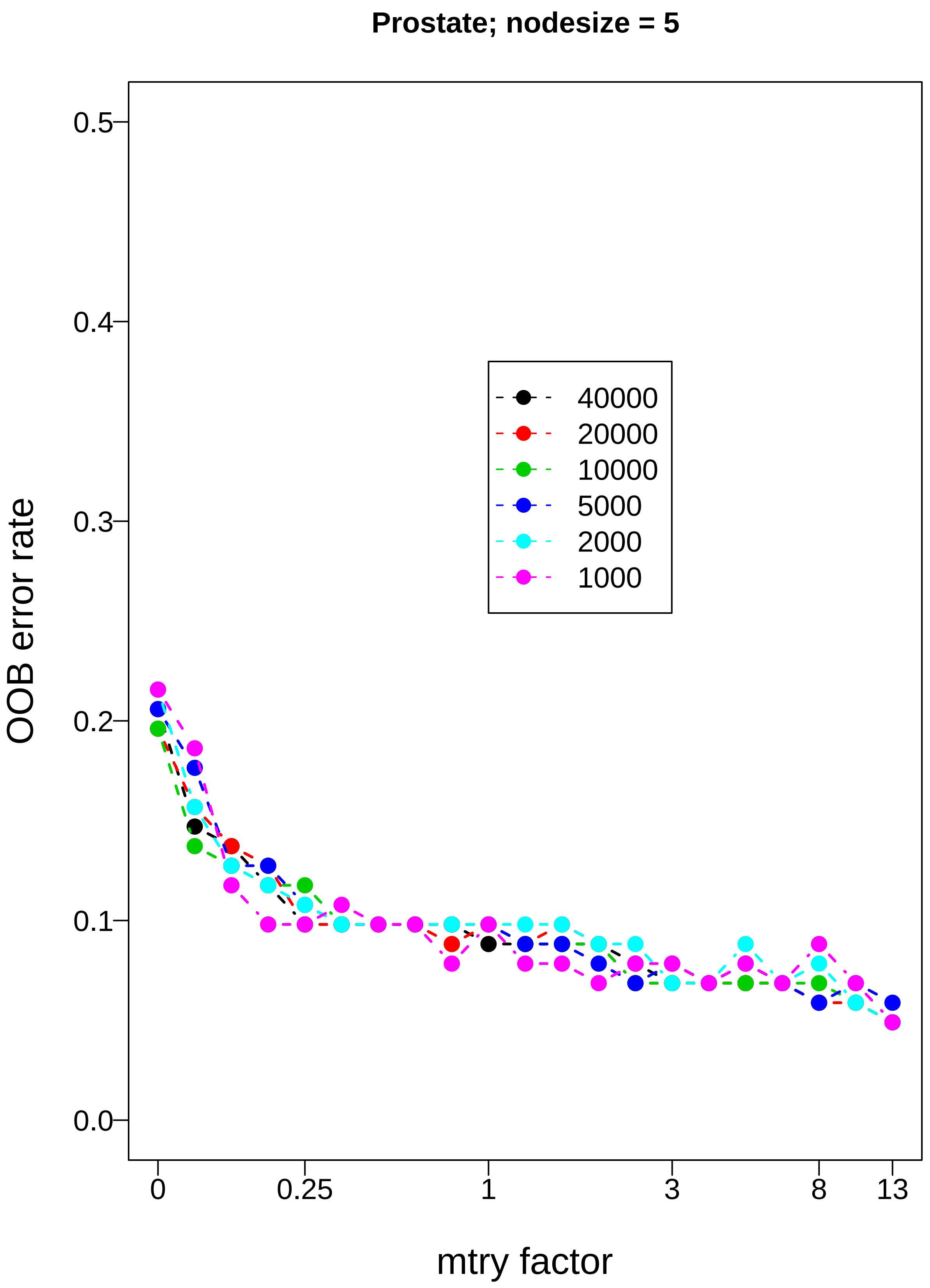
Prostate; nodesize = 5
OOB error rate
mtry factor
0.0
0.1
0.2
0.3
0.4
0.5
0
0.25
1
3
8
13
40000
20000
10000
5000
2000
1000

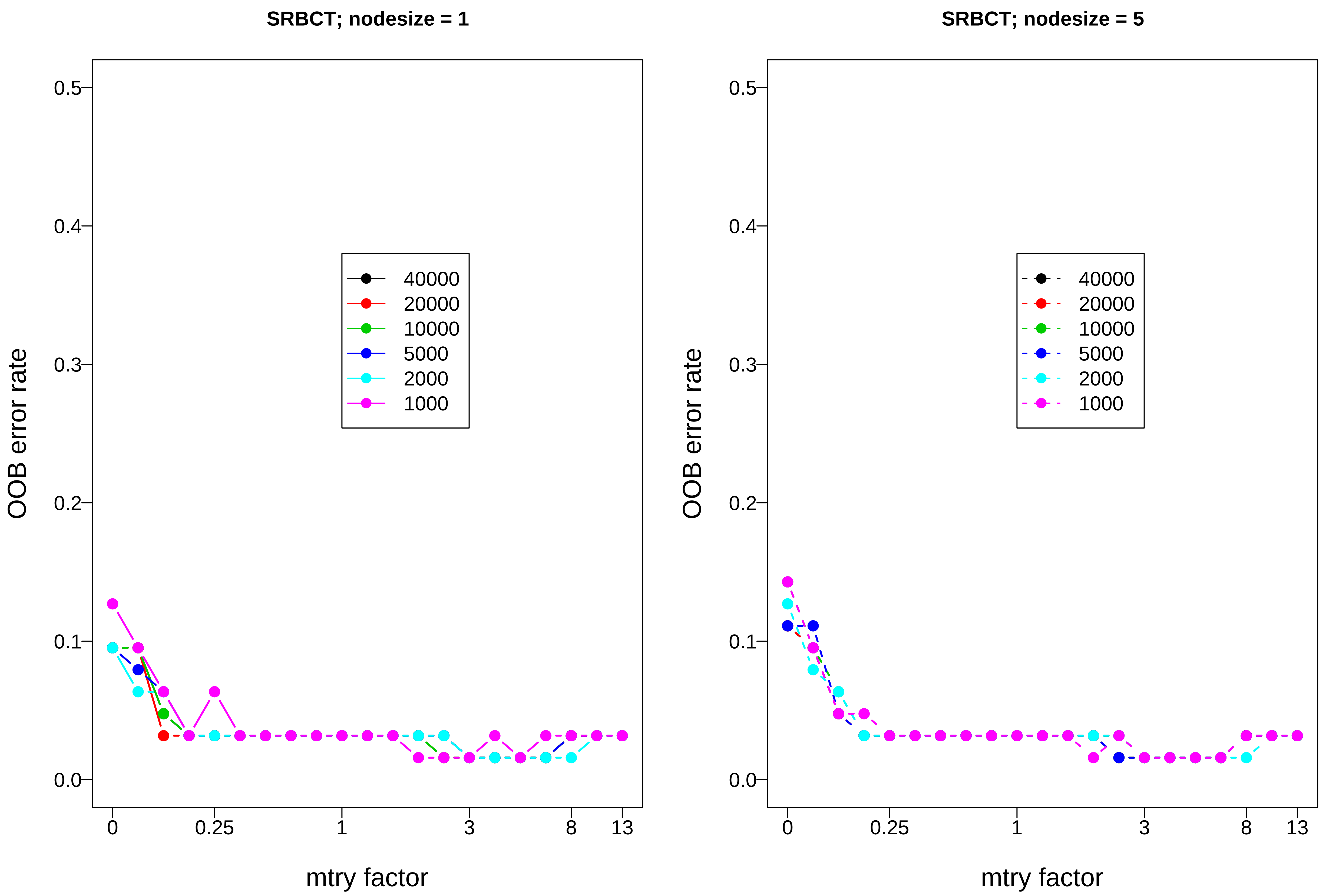

SRBCT; nodesize = 1
SRBCT; nodesize = 5
OOB error rate
0.0
0.1
0.2
0.3
0.4
0.5
mtry factor
0
0.25
1
3
8
13
40000
20000
10000
5000
2000
1000
